# Stability and Change in Gender Identity and Sexual Orientation Across Childhood and Adolescence

**DOI:** 10.1111/mono.12479

**Published:** 2025-07-15

**Authors:** Benjamin E. deMayo, Natalie M. Gallagher, Rachel A. Leshin, Kristina R. Olson

**Affiliations:** ^1^ Department of Psychology Princeton University

## Abstract

As increasing numbers of transgender, gender diverse, and queer youths come out to their friends, families and communities, their rights to express their identities in public life have become the subject of intense media scrutiny and political debate. But for all the attention transgender, gender diverse, and queer youth have received from politicians, journalists, and public intellectuals, basic science research on how these youth actually experience their identities over time remains scarce. In this monograph, we contribute to the emerging knowledge base on this topic by presenting a detailed quantitative description of gender identity and sexual orientation in a sample of over 900 North American transgender, gender diverse, and cisgender youths in the Trans Youth Project (*M*_age_ *= *8.1 years at first visit; *M*_age_ = 14.3 at latest visit; 99% living in the United States, 1% in Canada; 69% non‐Hispanic white; 73% household income >$75,000). Youths are in one of three groups: (1) a group of early identifying transgender youths, who were supported by their parents in a social gender transition (changing their name, pronouns, hairstyle, and clothing) by age 12 (*M*_age at transition_ = 6.5; *N* = 317); (2) a group of their siblings, who were cisgender at the beginning of their participation in the study (*N* = 218); and (3) a group of cisgender youths who were age‐ and gender‐matched to, but not family members of, the early identifying transgender youths (*N* = 377). Data on the youths' identities have been collected from the youths themselves and their parents between 2013 and 2024. We had two primary research goals. First, we described stability or change in youths' gender identity (Chapter 4) and sexual orientation (Chapter 6). We asked whether transgender youths' rates of change were or were not different from those of cisgender youths. Second, we examined whether measures of gender development earlier in development were related to youths' later gender identity (Chapter 5) or sexual orientation (Chapter 6) trajectories into adolescence. Stability in gender identity was by far the most common pathway for youths in all three groups, with over 80% of youths showing stability throughout their participation in the study. We saw similarity between the three groups of youths, such that the early identifying transgender youths were no more or less likely to show gender change than their siblings or youths in the unrelated comparison sample. Nevertheless, 11.9% of youths who started as cisgender were not so at their most recent report—a much higher proportion than would be predicted based on assumptions held in classic developmental psychology research about gender since the 1950s. When gender change did occur in all three groups, it overwhelmingly involved change to (and, to a lesser extent, from) a nonbinary gender identity. Results were similar regardless of whether youth‐ or parent‐report data were considered, and we found no evidence that youths were more or less likely to change at particular ages. We observed some evidence that more gender nonconformity in childhood (e.g., more femininity in childhood among children living as boys) was related to later gender change, but results were somewhat inconsistent across measures and gender identities. Youths showed diverse sexual orientations, with 60% of binary transgender and 33% of cisgender adolescents expressing queer (i.e., not straight) romantic or sexual interest. A high percentage of youths overall (37%) indicated interest in both boys and girls—a pattern particularly common among nonbinary youths. Finally, more than a third of youths have shown change in their sexual orientation, and childhood gender nonconformity was associated with whether currently binary transgender or cisgender teenagers most recently reported a queer identity. Our results accord with recent evidence indicating that today's youth are defying assumptions about gender and sexual orientation from decades of developmental research, considering gender and sexual orientation to be relatively flexible social identities rather than ones that are fixed, and view gender as having more than two categories. Early identifying transgender children's sense of their own gender was no more or less stable than cisgender children's, suggesting that children who are supported in their transgender identities tend to show developmental patterns that mirror their cisgender peers. Finally, in Chapter 7, we discuss how our findings exemplify and respond to this unique historical moment, the ways in which our findings do and do not align with past work about gender‐ nonconforming children, and how future research can continue to make strides toward better understanding a wider swath of gender development trajectories.

## I Societal and Empirical Contexts for Studying Gender and Sexual Orientation Across Development

Transgender and gender‐diverse youth represent an increasing proportion of young people in the United States, Canada, and Western Europe. According to data from the Center for Disease Control's (CDC) Youth Risk Behavior Survey [YBRS (Suarez et al., 2024)], the prevalence of transgender identity among youth aged 13–17 in the United States was estimated to be 3.3%, with an additional 2.2% questioning if they were transgender. A 2022 Pew Research Center poll suggested that 2.0% of 18–29‐year‐olds in the United States identified as transgender and 3% as nonbinary (Brown, 2022); less than a decade before, in 2016, national estimates suggested that 0.7% of 18–24‐year‐olds were transgender (Flores et al., 2016). Similarly, CDC data indicated that self‐identification as transgender among people aged 18‐24 increased almost fivefold from 0.6% in 2014 to 2.8% in 2022 (Twenge et al., 2024), and a 2024 Gallup survey indicated that 4.1% of adults 18–27 were transgender (Jones, 2025; Miller & Paris, 2025). These data and others from Western Europe and Canada (McKechnie et al., 2023; StatCan, 2022; van der Loos et al., 2023) indicate that increasing numbers of young people identify with a gender that differs from the one they were assumed to have at birth, and that today's children, adolescents, and young adults are increasingly likely to recognize gender identities that are not on either end of a binary spectrum. (For a list of key terms used throughout this chapter, see Table 1.)

**TABLE 1:** Glossary of Key Terms

Term	Definition
Assigned sex	The sex declared at or before a child's birth, typically on the basis of external genitalia or sex chromosomes
Gender identity	A person's sense of their own gender as, for example, a boy, girl, or nonbinary person
Transgender	Describes a person who identifies as a gender other than the one assumed on the basis of their assigned sex; can include nonbinary identities but is used in this manuscript to refer to binary transgender boys/men and girls/women
Gender diverse	Commonly used as an umbrella term referring to people who identify with a gender other than their binary, assigned sex and/or show consistent gender nonconformity; in Chapters III‐VII, we use “gender diverse” to refer to identities other than (transgender or cisgender) boy or girl, such as nonbinary and genderqueer
Nonbinary	A person identifying as neither a boy nor a girl, as both a boy and a girl, or as a combination of genders
Cisgender	Describes a person whose gender identity aligns with their assigned sex
Gender nonconforming	Describes traits, identities, behaviors, and preferences that run counter to cultural stereotypes expected of children of a certain assigned sex
Queer	Umbrella term describing people who are not heterosexual or cisgender; in this monograph, we use it as a sexual orientation descriptor for people who are lesbian, gay, bisexual, pansexual, or any sexual orientation that is not straight
Gender dysphoria	The experience of distress associated with an incongruence between one's assigned sex and one's gender identity, or the psychiatric diagnosis corresponding to this distress
Gender affirming medical care	Medical treatments intended to help bring one's body into alignment with one's gender identity; can include (for example) puberty blockers, hormones, or surgery
LGBTQ+	Umbrella term describing people who are not heterosexual or cisgender, including people who are lesbian, gay, bisexual, pansexual, transgender, nonbinary, queer, intersex, or asexual

*Note*: Some definitions adapted from deMayo et al. (2022).

As with gender diverse and transgender identities, the number of young people who report queer sexual orientation (e.g., lesbian, gay, bisexual, pansexual, etc.) has increased steadily over the past decade. In particular, Gen‐Z Americans (those born roughly between 1997 and 2012) show the highest levels of queer identification, with recent estimates ranging from 10% to 25% (Conron et al., 2020, Jones, 2024; Mpofu et al., 2023). A 2024 Gallup poll found that while 3.0% of Baby Boomers (born between 1946 and 1964) and 5.1% of Generation X (born between 1965 and 1980) identified as LGBTQ+ , 23.1% of Gen‐Z did (Jones, 2025). In addition, many more Gen‐Z Americans identify as bisexual—15.3% in another Gallup poll from the year prior—than identify as lesbian (3.0%) or gay (2.6%); in contrast, Baby Boomers have more members who identify as lesbian (0.7%) or gay (0.9%) than bisexual [0.6% (Jones, 2024)]. These findings suggest greater acknowledgement among young people today of sexual orientation identities that are not on either end of a binary spectrum—a phenomenon that in some ways parallels the significant rise in adolescents' and young adults' recognition of nonbinary gender identities (Twenge, 2023b, 2024).

These increases in LGBTQ+ identification have also meant that cisgender, straight people are becoming more familiar with gender diverse and queer people. While only 8% of people in 2008 indicated that they knew a transgender person (GLAAD, 2015), that number was more than 50% for 18–29‐year‐olds in a 2021 Pew Research Center poll (Minkin & Brown, 2021). The same poll also showed that while only 11% of 65+ year olds and 18% of 50–64‐year‐olds reported knowing someone who used nonbinary pronouns in 2021, 46% of 18–29‐year‐olds did. These experiences go hand in hand with changing views about gender more broadly: One poll estimated that 51% of Gen‐Z endorsed the idea that gender could be something other than male or female in 2021, up from 39% in 2019 (Twenge, 2023b).

**Figure 1:**
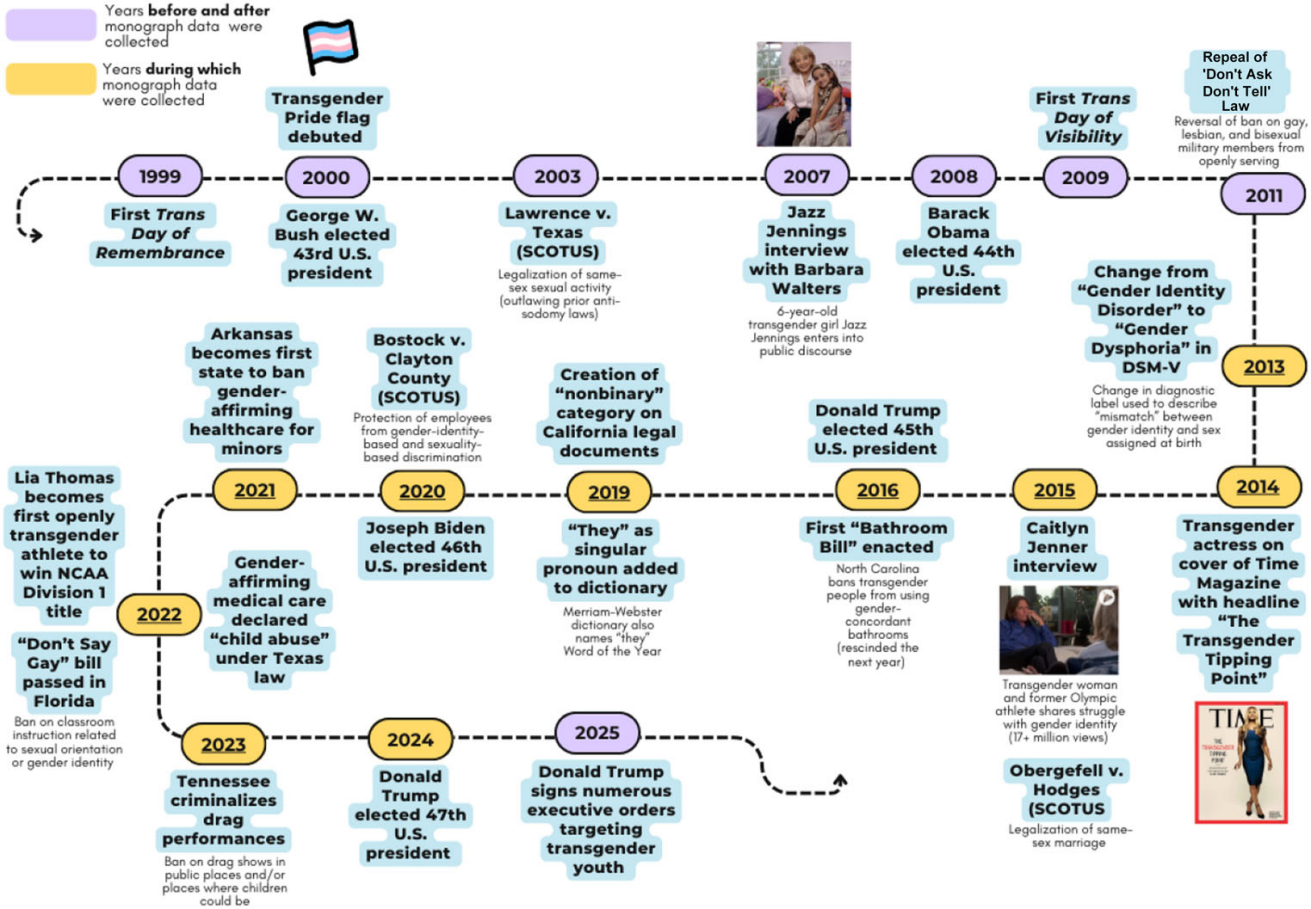
Key legal, historical, cultural, and linguistic LGBTQ milestones.

These changes in identity, first‐hand experience with transgender and gender‐ diverse people, and conceptions of gender have occurred within a rapidly changing broader social and political context (see Figure 1 for a broad overview of social, political, and linguistic changes over the last two‐and‐a‐half decades in the domain of LGBTQ+ rights and visibility). In the late 1990s, television programs in the United States began prominently featuring queer main characters, including the lesbian title character in Ellen DeGeneres's show *Ellen* and the gay lawyer Will Truman in the hit sitcom *Will and Grace*. Since then, it has become increasingly common for mainstream entertainment to feature queer characters, and many major celebrities have since come out as gay, lesbian, bisexual, and queer in the domains of music (e.g., Ricky Martin, Frank Ocean, Halsey, and Lil Nas X), sports (e.g., Megan Rapinoe, Jason Collins, Brittney Griner, and Sue Bird), comedy (e.g., Tig Notaro, Wanda Sykes, Bowen Yang, and Joel Kim Booster), and acting (e.g., Jodie Foster, Sarah Paulson, and Neil Patrick Harris). More recently, transgender and nonbinary celebrities have become increasingly visible, including singers Sam Smith, Janelle Monae, Kim Petras, and Demi Lovato; Olympic athletes Nikki Hiltz and Timothy LeDuc; comedians Mae Martin, Hannah Gadsby, and Patti Harrison; and actors Elliot Page, Laverne Cox, and Hunter Schafer.

As demographics and media portrayals have been changing, attitudes of the general public have also shifted. In 1996, only 27% of Americans thought that same‐sex marriage was valid, but by 2023, 71% did (Gallup, 2024). Similarly, while around 70% of Americans thought same‐sex sexual relations were wrong in the 1970s through early‐1990s, by the 2020s, fewer than 30% of Americans felt that way (NORC, 2022). And by 2022, 64% of Americans thought that transgender people should be protected from discrimination in jobs, housing, and public areas (Blazina & Baronavski, 2022). Meanwhile, public support for the rights of LGBTQ+ people remains high in Canada (Gubbala et al., 2023; Ipsos, 2018), where same‐sex marriage has been legal since 2005.

Since 2020, however, a clear backlash has emerged, particularly against transgender Americans. The right‐wing Republican Party, under the authority of President Donald Trump, has made anti‐transgender policy a central tenet of its political platform, targeting what it identifies as “woke gender ideology.” Prior to Trump's victory in the 2024 U.S. presidential election, the number of proposed anti‐transgender legislative bills—which concerned topics spanning bathroom access, restrictions on drag performances, and bans on medical care for transgender and gender‐diverse minors—grew quickly from fewer than 25 bills in 2019 to 600 in 2023 (Trans Legislation Tracker, 2024). In mid‐October 2024, less than a month before the election, 41% of all ads run by Donald Trump's presidential campaign had an anti‐trans focus; in total, the campaigns of Trump and other Republican candidates spent more than $200 million on anti‐transgender ads (Parks, 2024; PBS, 2024). Within a week of taking office, Trump signed several executive orders effectively outlawing any recognition of transgender or gender‐diverse identities under federal law and threatening to withhold federal funding from educational institutions that acknowledge gender diversity in any capacity; additionally, Trump has issued executive orders aimed at eliminating gender‐affirming medical care for minors and banning transgender youth from participating in athletic activities in all 50 states (White House, 2025a, 2025b, 2025c, 2025d).

Against this backdrop, several flashpoints in current discourse about gender‐ diverse youth have arisen. One controversy concerns the fact that in the past 5–10 years, clinicians have reported an especially steep increase in the number of adolescents seeking gender affirming care after childhoods in which they did not appear gender nonconforming—at least to their parents (Bazelon, 2022). While studies from decades ago have suggested that some people come out as transgender and/or first feel they might be transgender at or after puberty (Burns et al., 1990; Doorn et al., 1994; Nieder et al., 2011; van Kesteren et al., 1996), the increasingly large number of these youth—and the fact that the majority of them were assigned female at birth (past research had suggested more transgender people were assigned male at birth)—has stoked concern from clinicians and public media figures (Gentleman, 2022; Littman, 2018; Sapir et al., 2024; Shrier, 2020; Terhune et al., 2022; Wadman, 2018). The societal visibility of this group of gender‐ diverse teens has emerged in tandem with a public‐facing debate about how to approach their care, and whether it should differ from the care provided to youth who were clearly gender nonconforming or who socially transitioned in childhood (Edwards‐Leeper & Anderson, 2021; Ghorayshi, 2022). Coverage of these controversies in popular media has in turn been cited by politicians to promote legislation forbidding medical care for all transgender or gender‐diverse minors (GLAAD, 2023).

A particularly influential part of this discussion is the stories of self‐identified detransitioners—individuals who identified as transgender at one point in development and did not later. Some of these individuals received gender affirming medical care that they later regretted and have traveled around the United States speaking at legislative sessions about their experiences (Hennessy‐Fiske, 2023). As a result, the idea that transgender or gender‐diverse youth may not necessarily identify as transgender later in development has been covered heavily in popular media and prompts questions about the stability or malleability of childhood and adolescent identity—even though detransitioners themselves vary in their feelings about their individual gender trajectories (Pullen Sansfaçon et al., 2024).

All of these developments have made it so that transgender and gender‐diverse youth—and to some extent, queer youth more generally—occupy an outsized portion of the societal limelight and are consistently the topic of incendiary discourse in 2025 as we are writing this monograph. Their rights to participate in public life are constantly in question and the validity of their identities has become a common subject of debate in the political sphere. Yet, despite all this attention, basic science research on how these youth's identities develop over time has remained scarce. In this turbulent environment, developmental scientists can contribute to the public's emerging understanding of these youth's experiences, while also addressing broader questions about stability and change in gender and sexuality that have been discussed for decades—but which have taken on new resonance in an era in which many more youth are expressing flexibility and fluidity in these domains.

### I.1 Overview of Current Empirical Aims

In this monograph, we provide a detailed quantitative description of gender identity and sexual orientation across childhood and adolescence in a sample of North American youths—some transgender and gender diverse, some cisgender, some queer, some straight—with the aim of contributing to our field's and the public's emerging understanding of the various developmental trajectories youth show with respect to gender and sexual orientation. These youths are participants in the Trans Youth Project, a large‐scale longitudinal study of gender development and wellbeing that began in 2013. Although our monograph focuses on both gender identity and sexual orientation—and youths in our sample show variation on both of these dimensions—we recruited our sample on the basis of the youths' gender identities.

Our central group of interest at the start of the study was the *Recruited as Transgender* group, who underwent social transitions (i.e., changing names, pronouns, hairstyle, and clothing to align with one's gender, rather than one's assigned sex) by age 12 and before they joined this study. We recruited two comparison groups, each matched on different characteristics (further description of, and justification for, the three *recruitment groups* is provided in Chapter II). First, we included the cisgender *siblings* of the *Recruited as Transgender* group (henceforth, *Recruited as Siblings* group). This group was a helpful comparison given that their family characteristics and knowledge of gender diversity were matched to the *Recruited as Transgender* group. Second, we recruited an unrelated *Recruited as Cisgender* group, a sample of community‐recruited youths matched in age and gender to those in the *Recruited as Transgender* group. Because participants in these two comparison groups were coming of age in the same societal context as youths in the *Recruited as Transgender* group, their inclusion helped us to interpret if any findings in the *Recruited as Transgender* group should be construed as generational effects (i.e., characteristic of growing up in this particular historical moment) versus effects specific to being transgender (or living with a someone who is transgender) in childhood. On average, youths were 8.1 years old at their first visit in the study and have been followed for 6.2 years.

We have two main empirical aims, which we address after providing readers with an overview of the sample (Chapter II) and some of the measures (Chapter III) used in this monograph. First, we describe the youths' most recent identities—both gender (Chapter IV) and sexual orientation (Chapter VI)—and stability or change that they may have experienced between the beginning of their participation in the study and their most recent identity reports. Second, we probe whether measures of gendered behavior (e.g., gendered preferences for certain peers, toys, or clothing) earlier in development are related to gender identity, gender identity change, and sexual orientation later in development (Chapters V and VI). Overviews of the remaining chapters are found at the end of this chapter.

In sum, we hope to provide a detailed portrait of how the youths in our sample experience their gender identities and sexual orientations over time. Our broader goal is to contribute to the general public's understanding of transgender, gender diverse, and queer youth while also providing empirical evidence that bears on classic theories and assumptions about gender development that psychologists have held since the inception of our field.

### I.2 Empirical Context for the Current Study

While the number of youths coming out as transgender, gender diverse, and queer—as well as the broader political and social backdrop around them—is rapidly changing, researchers have been interested in questions about gender and sexuality change for decades, yielding a host of notable findings that have guided research in the field to date. We highlight this empirical background below, along with how the current work can contribute to the field's knowledge in these areas.

#### I.2.1 Stability and Change in Gender Across Childhood and Adolescence

##### I.2.1.1 Key Past Work

Over the past 75 years, a key feature of scholarship on gender identity and its stability or change across childhood and adolescence has been the way different subfields of psychology developed theories and empirical descriptions of gender largely in isolation from one another. Since at least the 1950s, research in developmental psychology has generated a normative description of gender development in childhood and adolescence that assumed that one's gender identity is concordant with their assigned sex at birth and that gender is an invariant trait across the lifespan once it emerges early in childhood. This research has demonstrated that children show awareness of gender and can discriminate between faces of different genders within the first year of life (Fagot & Leinbach, 1993; Leinbach & Fagot, 1993; Quinn et al., 2002). Awareness of one's own identity and reliable self‐categorization (along with the labeling of other people's gender) typically emerge between ages 2 and 3, with some variability (Etaugh et al., 1989; Fagot, 1985; Fagot et al., 1986; Leinbach & Fagot, 1986; S. K. Thompson, 1975; Weinraub et al., 1984). After children have gained the ability to label their own gender, the assumption in this literature has generally been that gender is static for the rest of the child's life; in fact, psychologists from the cognitive‐developmental tradition have long considered it to be a milestone in and of itself when children become completely certain of the unchangeability of their own gender (Kohlberg, 1966).

Research in developmental psychology has explored stability and change in other aspects of gender development such as stereotyping (Halim & Ruble, 2010; Signorella et al., 1993; Trautner et al., 2005), self‐segregation into gendered groups (Maccoby & Jacklin, 1987; Martin & Fabes, 2001; Serbin et al., 1993), and gendered preferences (Golombok et al., 2008; Halim et al., 2013; Maccoby & Jacklin, 1987) but has generally not investigated stability and change in self‐categorization until very recently (e.g., Katz‐Wise et al., 2024). In fact, although some work has investigated longitudinal stability and change on various continuous dimensions of gender identity [such as one's feeling that they are typical of their gender category, or their sense of contentedness with being a certain gender (Yunger et al., 2004)], researchers have explicitly stated that youth's knowledge that they are or are not members of a particular gender category will not show any individual differences after age 7 (Tobin et al., 2010, p. 608). In other words, past work has operated under the assumption—true in most people, but not everyone—that people do not question or change their gender category membership after middle childhood.

While developmental psychology has typically focused on youth who we might today describe as cisgender, this work did sometimes examine variability in gender development (even if this has not been the dominant theme in the literature). As an example, some studies have focused on the gender development of tomboys or girls who show some stereotypically masculine characteristics (Ahlqvist et al., 2013; Bailey et al., 2002; Green et al., 1982; Halim et al., 2011; Martin & Dinella, 2012; Plumb & Cowan, 1984). This research has often shown that a tomboy identity is both common among girls—with some studies indicating that up to 50% of girls adopt the tomboy label—as well as flexible, with studies suggesting that tomboys often show interest in both stereotypically masculine and feminine activities and interests. These findings are significant because they refute the notion that it is exceedingly rare for children to engage with stereotypically “cross‐gender” interests.

A separate but related literature has emphasized that even in childhood, people's conception of their own gender identity (i.e., whether they are a boy, girl, or something else) and gender typicality (i.e., how they see themselves relative to the stereotypical boy or girl) is more complex than simply labeling oneself a boy or girl. As a result, researchers have measured gender in childhood and adolescence as a multidimensional or continuous construct for decades (Bem, 1974, 1981; Egan & Perry, 2001; Martin et al., 2017; Spence, 1993; Spence & Helmreich, 1979), showing that children and adolescents can and do show individual differences in their level of identification with binary gender categories, with important consequences for real‐world outcomes like self‐worth and psychological adjustment (Carver et al., 2003; Corby et al., 2007; Yunger et al., 2004). Some prominent theorists advocated that being high in masculinity *and* femininity was not only possible, but beneficial for children and adults (Bem, 1981; Bem & Lewis, 1975).

In this way, a subset of past research in developmental psychology has acknowledged and explored the inherent variability in gender development, foreshadowing the approach that we take in this monograph. Nonetheless, the majority of literature in gender development has focused on characterizing the modal (i.e., most common) developmental pathway: that of being a cisgender child. Furthermore, even in cases when research has concerned itself with documenting non‐modal development, it has generally not included specific descriptions of children who show the most “extreme” forms of gender nonconformity (for example, children assigned male at birth who regularly assert that they are or desire to be girls), even if children who fit this description may have been participants in some past research.

This does not mean, however, that research on more marked forms of gender nonconformity in childhood and adolescence was nonexistent during the same time period. In fact, research documenting the trajectories of children who showed gender nonconformity has been conducted since at least the 1950s, mostly in the fields of clinical psychology, sexology, and child psychiatry, and typically within medical centers associated with major research institutions or universities. This research tradition began with small‐scale clinical case studies of (primarily) children assigned male at birth who showed stereotypically feminine behavior, such as playing dress‐up with their female relatives' clothes or sometimes asserting that they wanted to be girls (Bakwin, 1960; Friend et al., 1954; Green & Money, 1960, 1961; Stoller, 1968; Zuger, 1966; Zuger & Taylor, 1969). These case studies evolved into larger, more systematic research programs undertaken at several major medical clinics in North America and Western Europe beginning in the late 1960s and continuing through today (Green, 1974, 1987; Singh et al., 2021; Steensma et al., 2013a; Wallien & Cohen‐Kettenis, 2008; Zucker et al., 1985, 2012; Zucker & Bradley, 1995) which included both assigned‐male and (relatively fewer) assigned‐female children who showed pronounced gender‐nonconforming behavior, a desire to be or an insistence that they were a different gender, and/or gender dysphoria.

Research from this tradition generated a detailed description of children with gender‐nonconforming characteristics and is sometimes invoked in popular media discussions about long‐term outcomes for transgender and other gender‐diverse youth (Paul, 2024; Vilain & Bailey, 2015). Findings from these studies suggested that most children who expressed notable levels of gender‐nonconforming behavior did not go on to identify as transgender as adolescents or young adults later in development, though they did tend to be gay or bisexual. In other words, one consequential upshot of these studies was that children who express interest in being a member of a different gender group will generally outgrow (or “desist” from) these feelings later in life and that continuing to identify (or wanting to identify) as a different gender is rare once these youth reach adolescence or young adulthood.

However, it is difficult to apply this finding from past work with clinical samples to the gender development of children and adolescents—both gender diverse and cisgender—in the present day. With few exceptions, the former body of work has operated under the assumption that any fluctuation in a child's gender identity from the one associated with their sex at birth (e.g., a child assigned male at birth insisting that they are or want to be a girl) is a pathological condition that, with the right treatment, should go away (J. E. Bates et al., 1975; Bradley & Zucker, 1990; Coates & Person, 1985; Rekers et al., 1977; Stoller, 1967; Zucker, 2005). Many of the clinics and investigators conducting this research specifically offered treatments aimed at changing a child's gender identity and/or behavior (Bakwin, 1968; Green, 1987; Rekers et al., 1976; Zucker & Bradley, 1995) or expressed skepticism and caution about allowing children to change genders before puberty (Steensma & Cohen‐Kettenis, 2011). Reflecting this framing of persistent childhood gender nonconformity as a psychologically distressing phenomenon that should be stopped rather than affirmed, the diagnosis of Gender Identity Disorder (GID) was added to the *Diagnostic and Statistical Manual of Mental Disorders* [DSM‐III (American Psychiatric Association, 1980)] in 1980 and was later rebranded as Gender Dysphoria (GD) in 2013 (Zucker, 2005, 2014).

The idea that most gender dysphoric children develop into cisgender adolescents or adults may or may not apply to present‐day youth whose parents and clinicians are relatively more accepting of transgender identities. Many medical professionals and clinicians in recent years have adopted an affirmative approach in which gender diversity is not framed as pathology but rather as normal variation within a spectrum of gender trajectories (Keo‐Meier & Ehrensaft, 2018; Kuper et al., 2019; Rider et al., 2020), and therefore support gender‐diverse youth through social and/or medical transitions when the youth and their families also endorse those paths.

It is also difficult to determine whether children in the clinical samples of past work were qualitatively different from or similar to the transgender children who are coming out in higher numbers in the present day (Temple Newhook et al., 2018), which has led to debate and confusion (Tannehill, 2017; Winters, 2019; Winters et al., 2018; Zucker, 2018). For example, Olson (2016) pointed out that few children in the older clinical samples actually insisted that they *were* another gender (Zucker et al., 1999; Zucker & Bradley, 1995), whereas most of the youth socially transitioning today are identifying with a gender that differs from their assigned sex. Contemporary research has suggested that the children who are socially transitioning in the last decade or two may be a subset of gender‐nonconforming youth who show particularly strong gender nonconformity in childhood (Rae et al., 2019). The societal context in which transgender youth are growing up today has also changed drastically since the 1970s—early 2010s, when most past work with clinical samples was conducted: the general public is much more aware and accepting of transgender youth today (despite persistent anti‐trans sentiment, including from politicians). Relatedly, some transgender youths—and many youths in our sample—have access to medical treatments that would not have been available for many youths prior to 2010, meaning they have had the opportunity to present comfortably in their gender identities without it being obvious to others that they are transgender (even if these medical treatments are rapidly being banned or legally challenged in the United States and other Western countries).

In sum, mainstream developmental psychology has tended to characterize modal patterns of gender development while dedicating relatively less scholarly attention to other developmental trajectories—such as those in which a child's gender changes over time or diverges from their assigned sex. At the same time, most (though not all) extant work on gender‐nonconforming youth has framed high levels of gender nonconformity as a clinical disorder and consequently has focused significant attention on whether or not the so‐called disorder would continue into adolescence. The result is that the field of developmental science lacks a broader description of stability and change in gender identity across childhood and adolescence that does not implicitly or explicitly consider pronounced gender nonconformity a rare, pathological aberration from typical development. Additionally, because of its assumptions that gender‐conforming children will maintain cisgender identities over time, past work has not followed groups of cisgender youth and tracked *their* identities over time to describe how stable or variable (assumed‐to‐be) cisgender children's identities are.

##### I.2.1.2 Current Contribution

We offer the first detailed longitudinal description of stability and change in gender identity in a large cohort of youths who socially transitioned as children, who—for reasons described above—may not show the same developmental trajectories as other cohorts of gender‐nonconforming children in past research who were recontacted as adolescents or adults. We also compare the trajectories of children *Recruited as Transgender* to those of their siblings and a group of unrelated cisgender youths, who themselves offer the opportunity to answer interesting questions about how broad swaths of youth experience stability and change in their gender identity in a sociocultural context that is rapidly changing. Though we could not have known it in 2013 when we began this research, this cohort of youth—and members of their generation—are perhaps the most diverse in terms of gender of any generation in recent U.S. and Canadian history. Their inclusion in this study provides a unique scientific opportunity to examine their gender identities as these youths develop in a time of notable societal shift.

#### I.2.2 Sexual Orientation Across Childhood and Adolescence

##### I.2.2.1 Key Past Work

Since at least the 1970s, a large research literature has characterized the development of sexual orientation across the lifespan, including during childhood and adolescence. Sexual orientation is a trait that includes several distinguishable constructs including sexual attraction, self‐identification or self‐labeling, romantic orientation, and sexual behavior (Mustanski et al., 2014; Savin‐Williams, 2006). Unlike the historical scholarship on gender identity, the literature on sexual orientation has devoted a sizable emphasis to non‐modal trajectories and change and stability over time.

This literature has shown that once youths experience their first romantic or sexual attraction, which tends to occur around ages 9–12 (Herdt & McClintock, 2000; Maguen et al., 2002; Rosario et al., 1996; Savin‐Williams, 1995), it is not uncommon for people's sexual orientation to change over time; one review found that more than 20% of late adolescent, young adult, and middle‐aged sexual minority participants reported fluctuation in their sexual orientation in several large longitudinal studies (Diamond, 2016; Dickson et al., 2003, 2013; Katz‐Wise et al., 2023a, 2023b; Katz‐Wise & Todd, 2022; Ott et al., 2011; Savin‐Williams et al., 2012; Savin‐Williams & Ream, 2007). Other studies have shown that participants who identify as straight at the end of a longitudinal period did not necessarily think of themselves as such at all points in their life prior (Diamond, 2003; Sandfort, 2005), suggesting that change over time may not be exclusive to people who ultimately “settle” on queer identity.

One dominant focus of past work has been on gender differences in sexual orientation. Studies in the United States have often shown that women are more likely than men to report attraction to both men and women (Chandra et al., 2013; Dickson et al., 2003, 2013; Mosher et al., 2005; Savin‐Williams et al., 2012) and that women tend to report more change over time in sexual attractions than men, particularly toward a nonexclusive sexual orientation like bisexuality (Dickson et al., 2003; Mock & Eibach, 2012; Ott et al., 2011; Savin‐Williams et al., 2012; Stewart et al., 2019). However, not all studies have not found this effect (e.g., Katz‐Wise, 2015; Rosario et al., 2006).

While the relation between gender and sexual orientation in cisgender men and women has been well‐documented, this relation is less clear in transgender and other gender‐diverse people. Since the 1950s, researchers, physicians, and psychoanalysts have put forth various typologies of transgender identity, sometimes arguing that different “types” of transgender people could be distinguished by their sexual and romantic interests in adolescence or adulthood (Lawrence, 2010). One controversial theory posited that assigned‐male youth who show consistent gender nonconformity early in childhood constitute a distinct type of transgender woman who will be attracted solely to men when they grow up (Bailey, 2003; Blanchard, 1988). Though research on contemporary transgender youth's sexual orientations is still emerging, evidence from the past 10 years suggests that sexual and romantic interests among transgender and gender‐diverse youth (including transgender girls) may be significantly more varied than this theory predicts. Several studies of transgender and gender‐diverse youth from large school surveys (e.g., Gower et al., 2022; Szoko et al., 2023), gender clinics (Boskey & Ganor, 2022), and community samples collected online or through community organizations (Bosse & Chiodo, 2016; Price‐Feeney et al., 2020; Salk et al., 2020) have reported that well over half of surveyed transgender and gender‐diverse youth identify as queer. Some recent work from other gender clinics has reported that while the majority of transgender youth in their samples were straight, a substantial minority (17%–23%) reported queer sexual or romantic attraction, and even more were questioning or unsure (Bungener et al., 2017; Olson et al., 2015). This recent work raises the possibility that many transgender adolescents and adults are queer—perhaps at a much higher rate than cisgender people. It also leaves open the question of how stable sexual orientation is in transgender and gender‐diverse adolescents, since longitudinal work on their sexual orientations is scarce except for one study suggesting that sexual orientation change may be common in this group (Katz‐Wise et al., 2024).

##### I.2.2.2 Current Contribution

A major contribution of this monograph is that we build on the emerging, but still relatively small, literature about sexual orientation in transgender and gender‐ diverse youth by providing the first report of sexual orientation in the Trans Youth Project cohort. In doing so, we also report one of the first longitudinal descriptions of sexual orientation in transgender and gender‐diverse youth across multiple developmental time points. Furthermore, we describe stability and change in sexual orientation in both transgender and cisgender youth—a topic that has mostly been studied in cisgender adolescents (e.g., Katz‐Wise et al., 2023a, 2023b) and young adults.

#### I.2.3 Childhood Associates of Later Gender Identity or Sexual Orientation

##### I.2.3.1 Key Past Work

Past work has examined whether childhood measures of gender development (e.g., how stereotypically masculine or feminine a child's preferences for clothing or play activities are, or how strongly they identify as a member of a particular gender group) relate to their gender identity or sexual orientation later. While childhood associates of sexual orientation have received more empirical attention than childhood associates of later gender identity (likely because gender change has traditionally been regarded as such a rare outcome), some research with youth in gender clinics (e.g., Wallien & Cohen‐Kettenis, 2008; Steensma et al., 2013a; Drummond et al., 2008; Singh et al., 2021) has asked whether stronger gender nonconformity in childhood was related to continued gender dysphoria in adolescence and young adulthood. Examples of gender‐nonconforming behaviors that researchers assessed in childhood included wanting to dress in clothes stereotypically associated with the other binary gender, being interested in counter‐stereotypical toys or play activities, and regularly pretending to be or stating a desire to be the other binary gender. In general, this literature found that more gender‐nonconforming children tended to continue to be gender dysphoric (i.e., “persist” in their gender dysphoria) later in development at higher rates than more gender‐conforming children. A more detailed introduction to this literature can be found in Chapter V.

A relatively larger literature on childhood predictors of later sexual orientation has generally found that gender nonconformity in childhood is related to queer sexual orientation in adolescence and adulthood. The strongest evidence for this trajectory comes from prospective studies that assessed gender nonconformity in childhood and then followed up with participants later in life; these studies have typically found that more feminine interests in boys and more masculine interests in girls are associated with being queer in adolescence and adulthood (e.g., Li et al., 2017; Wallien & Cohen‐Kettenis, 2008; Zucker & Bradley, 1995; Steensma et al., 2013b).

##### I.2.3.2 Current Contribution

Whereas past work on the relation between gender nonconformity in childhood and later gender identity has taken place in clinical settings with gender‐nonconforming youth who had not socially transitioned, we have the opportunity to examine whether patterns seen in those samples are also present in socially transitioned transgender youth. In particular, we can probe whether it is the case that transgender children who show more gender nonconformity relative to their gender (for example, transgender girls [assigned male at birth] who are relatively masculine compared to other girls) are more likely to be cisgender or adopt a different gender identity later in life. Similarly, past work has generally not explored this topic with youth who were ostensibly gender conforming in childhood; this sample also allows us to test whether, among participants who began our study as cisgender, childhood gender nonconformity is associated with later gender change from cisgender to transgender or gender diverse. Finally, we can examine whether the robust relation in past literature between gender nonconformity and queer sexual orientation holds when transgender youth are included—for example, is it the case that, among both cisgender and transgender girls, those who are most stereotypically masculine in childhood are more likely to be lesbian or bisexual in adolescence?

### I.3 Overview of the Following Chapters

This monograph is organized such that Chapters II and III are primarily methodological chapters, whereas Chapters IV—VI focus primarily on reporting empirical results. Our main empirical goals in the monograph overall are to: (1) characterize stability and change in gender identity and sexual orientation among youths in the Trans Youth Project, which we do in Chapters IV and VI; and (2) examine the relation between childhood indicators of gender development and later gender or sexual orientation outcomes in adolescence or young adulthood, which we do in Chapters V and VI. Our general approach to answering these questions was to assess gender identity and sexual orientation throughout development from childhood to adolescence or early adulthood. Particularly as youth matured, we tended to assess these constructs with multiple measures to adapt as language for these constructs changed and to allow participants to fully express their identities. However, to address our central questions about stability and change, we distilled these measures into a single assessment of each youth's gender and sexual orientation at each visit, processes we describe in Chapters III (gender) and VI (sexual orientation). Below, we provide an overview of each following chapter.

#### I.3.1 Chapter II: Trans Youth Project Participants and General Design

Chapter II familiarizes readers with the Trans Youth Project, providing an in‐depth description of the *recruitment groups* in the study. We describe participant retention and attrition, detailing how long participants have been involved in the study and what percentage of them are still active. We also provide more detailed context about youths in the *Recruited as Transgender* group, describe what kinds of gender‐nonconforming behavior they showed early in childhood, and detail when in development they reached certain milestones in transition (e.g., first signs of gender nonconformity, first social transition, puberty blocker/hormone use if applicable).

#### I.3.2 Chapter III: Assessing Youth‐ and Parent‐Report Gender at Each Visit

Chapter III introduces the measures of gender identity that have been used at different points throughout the study and the framework we used to derive a single assessment of a youth's gender each time they or their parent participated. In cases in which our team manually coded youth or parent responses to determine a participant's gender or sexual orientation at a particular point in time, we describe that coding process. We also report how well various indices of the same construct (e.g., parent‐report and adolescent self‐report of the adolescent's gender, two different youth‐report measures that both assess gender identity, etc.) cohere statistically.

#### I.3.3 Chapter IV: Stability and Change in Gender Identity Over Time

Chapter IV describes stability and change in gender identity and the current gender identities of our participants. First, we examine how often participants' gender identities have changed during the course of the study. We ask if this kind of change varies by *recruitment group* (*Recruited as Transgender*, *Cisgender*, or *Siblings*) or reporter (youth or parent), and to the extent that youths show change, if there are particular times in development when it was especially likely. We then report on youths' current (i.e., most recently reported) gender identities: What identities are they (and their parents) reporting? Did members of some participant groups show more or less stability between the beginning of the study and its current point?

#### I.3.4 Chapter V: Associations Between Childhood Gender Development and Current Gender Identity

In Chapter V, we test whether childhood gender development is related to later gender identity. We specifically ask if there is an association between gendered characteristics (including gender‐typed preferences, early self‐report of gender identity on a continuous scale, and parents' ratings of their child's gender nonconformity) earlier in development and gender identity later in adolescence or adulthood.

#### I.3.5 Chapter VI: Development, Stability, and Change in Sexual Orientation Across Childhood and Adolescence

In Chapter VI, we report on youths' sexual orientations. We detail patterns of romantic and sexual attraction to boys, girls, or nonbinary people, whether youth identify as asexual or aromantic, and whether they are questioning or unsure of their identity. We discuss stability and change over time in youths' sexual orientations, probing whether there are group differences (i.e., between youths in the *Recruited as Transgender*, *Recruited as Cisgender*, and *Recruited as Siblings* groups) in levels of stability and change in sexual orientation, and test whether gender nonconformity in childhood is related to sexual orientation later.

#### I.3.6 Chapter VII: Key Findings, Strengths, Limitations, Future Directions, and Implications

Chapter VII provides readers with a general synthesis of the main findings presented in prior chapters, with an emphasis on how these findings do or do not align with what is already known about gender identity and sexual orientation across development. We also discuss how our results may be affected by, and bear on, sociocultural shifts relating to LGBTQ+ identities among today's youth (i.e., the rapid increase in visibility of transgender and especially nonbinary gender identities). We highlight strengths and limitations of our work with a focus on our sample's diversity and generalizability, retention and attrition, and best practices for open and rigorous science. Finally, we discuss ongoing work that our research team plans to continue with this cohort of youths, as well as topics relating to gender diversity and sexual orientation across development that need more research.

## II Trans Youth Project Participants and General Design

### II.1 Chapter Highlights

- In this chapter, we discuss what the Trans Youth Project is. What is its purpose? What is the history of the project, and how did it evolve over time? Who are the participants in this research—both the youths and the parents? For these topics, see **Overview of Study History and Scope** and **Participants**.
- We describe the format of the longitudinal design that we used, including the format of data collection (e.g., online surveys, face‐to‐face visits, etc.), how frequently families participated in the research, and attrition. For this topic, see **General Design and Methodology.**
- We end the chapter with an in‐depth description of the early experiences of the transgender youths in our sample, explaining what their parents noticed about their children at early ages, when the children initially socially transitioned, and where they are in the process of medical transition (if the family has chosen to pursue gender affirming medical care). For this topic, see **Additional Detail on the** ***Recruited as Transgender*** **Group**.

### II.2 Overview of Study History and Scope

The Trans Youth Project (TYP) began in 2013 at the University of Washington in Seattle, Washington, USA. The study's goal since its inception has been to longitudinally examine gender development and wellbeing in transgender and gender nonconforming children, as well as to compare these children's development to their counterparts who were cisgender in childhood, with the stated goal of following youths for 20 years. In the first year of the study, participant recruitment proceeded at a relatively slow pace, and only approximately 30 transgender children and their families were recruited into the study. By 2016, word about the study had spread among families of transgender youths, and this number grew to over 300. While there were a few qualitative studies of transgender adolescents or parents of transgender children that predate or came out around the same time as the TYP (e.g., Grossman & D'Augelli, 2006; Guss et al., 2015; Kuvalanka et al., 2014; Meadow, 2011), TYP was the first large‐scale quantitative study of transgender children who had socially transitioned.

Our main empirical goal in this monograph is to characterize stability and change in gender identity and sexual orientation among the youths in our sample. The data we draw on to do so come from youths' and parents' longitudinal reports on measures of gender identity, gender‐typed preferences (e.g., for stereotypically masculine or feminine clothing or toys), and sexual orientation. However, participants have reported on many other topics that we do not discuss in this monograph, including mental health and wellbeing, parent and community support, experiences with medical transition (for youths to whom this applies), dating, peer victimization and bullying, participation in sports, gender stereotyping and essentialism, involvement in activism, and reactions to anti‐transgender legislation and political actions, among other topics.

### II.3 Participants

#### II.3.1 Recruitment Groups

At the project's inception, its focal group was a *Recruited as Transgender* group of children who had, with the support of their parents, undertaken social transitions by age 12—in so doing, changing their public identity (i.e., often hairstyle, clothing, first name, pronouns) to align with the binary gender other than their assigned sex at birth. Key questions about these youths' long‐term gender development trajectories and wellbeing necessitated the recruitment of two comparison groups. The first was a *Recruited as Siblings* group consisting of cisgender siblings of youths in the *Recruited as Transgender* group; this group was primarily recruited to match the *Recruited as Transgender* group on home environment characteristics and exposure to discourse about gender diversity. The second comparison group was a *Recruited as Cisgender* group consisting of community‐recruited cisgender youths who were matched in age and gender (not assigned sex) to youths in the *Recruited as Transgender* group. Youths in the *Recruited as Cisgender* group were mostly recruited from the Seattle, Washington metropolitan area. The *Recruited as Cisgender* group was recruited for several reasons: (1) to mirror past work on gender‐diverse youths, which sometimes included comparison groups of “gender‐typical” children and adolescents (e.g., Green, 1987); (2) to assess the extent to which developmental trajectories observed in transgender children and their siblings were also present in youths who presumably had less exposure to gender diversity on a daily basis; and (3) to be more closely age‐ and gender‐matched to the *Recruited as Transgender* group than the *Recruited as Siblings* group was. Because we were broadly interested in the trajectories of any youths who had undergone social transition prior to adolescence, we recruited participants in the *Recruited as Transgender* group from a relatively wide age range between 3 and 12 years; this criterion was consequently applied to the two comparison groups (with occasional exceptions discussed in the sections that follow). Below, we provide more details on the three *recruitment groups*.

##### II.3.1.1 Recruited as Transgender

The *Recruited as Transgender* group (*N* = 317) consists of youths who, with the support of their parents, had undergone a binary social gender transition by age 12. This means that at their first visit in the study, youths were either living as transgender boys (boys who had been assigned female at birth) or transgender girls (girls who had been assigned male at birth). To qualify for inclusion, youths in this group had to have undergone a “complete (social) transition” (Steensma et al., 2013a); most often, this involved a change in hairstyle, clothing, and first names. However, because none of these changes uniquely maps onto being transgender (e.g., someone can be a boy with long hair or have a gender‐neutral name), we used information about the child's current gender pronouns—provided by parents and, in cases when children were old enough, confirmed by children—as the final determination of a child's qualification into this group. That is, children in the *Recruited as Transgender* group had to use the binary pronouns associated with the “opposite” sex (i.e., he/him for assigned females; she/her for assigned males) in all or nearly all situations—for example, at their home(s), at school, and with relatives. An occasional exception was allowed if, for example, a relative with whom the child seldom or never interacts still used the child's original pronouns. If youths used different pronouns across different situations (e.g., he/him pronouns at school, she/her pronouns at home) or were in the process of social transition, they were deemed ineligible for inclusion into the study [these youths were included in a separate longitudinal project (e.g., Durwood et al., 2023; Rae et al., 2019)].

##### II.3.1.2 Consideration of “Nonbinary” Category

As we write this monograph (and present findings from it) in 2025, our research team is often asked about our (lack of) inclusion of nonbinary youths in the *Recruited as Transgender* group. During our in‐person visits with families in 2013–2017, we met very few youths (if any) who were using they/them pronouns consistently across contexts; moreover, the term “social transition” was, at the time, primarily used by researchers and families to refer to binary transitions (Steensma et al., 2011). We did occasionally meet families with youths who, for example, felt like a mix of a boy and a girl, but their parents did not tend to describe them as having socially transitioned; thus, they did not meet our study's inclusion criteria, so we did not include them in the TYP sample. Given this context, we refer to youths in the *Recruited as Transgender recruitment group* as having been transgender girls or transgender boys, even though we understand that today, with other terms available, it is possible that some of these youths might have opted to describe themselves with other terms such as nonbinary. Importantly, if the research team had any indication that the child did not live as a boy or girl in everyday life—for example, if the child lived as a mix of a boy and girl or avoided the use of all pronouns—they were not included in the *Recruited as Transgender* group and instead were included as part of a long‐term study of (other) gender nonconforming youth. As we discuss more in subsequent chapters of the monograph, some children did, at an early visit with the research team, select gender identity terms other than boy or girl (including in the *Recruited as Cisgender* and *Recruited as Siblings* groups), and we conduct some analyses based on those initial responses (though the overall patterns nearly always look the same regardless of analysis approach). It is undoubtedly the case that more young people (including those in the study and those not in the study) use the term nonbinary and/or use they/them pronouns than did when we began the study (Twenge, 2024).

##### II.3.1.3 Recruited as Siblings

The *Recruited as Siblings* group (*N* = 218) consists of siblings of youths in the *Recruited as Transgender* group, all of whom were cisgender at the beginning of their participation in the TYP. Inclusion of the *Recruited as Transgender* group's siblings allows us to infer that differences between participant outcomes among youths in the two groups are likely not due to being raised in different household contexts, local communities, or political environments. Additionally, the *Recruited as Siblings* group is a useful comparison to the *Recruited as Cisgender* group (an unrelated comparison group of initially cisgender youths, described in the next section) because it provides information on whether there are systematic differences in long‐term outcomes between children who grow up in a family that is actively supporting a transgender child and those who do not. Conversely, the *Recruited as Siblings* group contains some limitations. Youths in this group are not age‐ or gender‐matched to youths in the *Recruited as Transgender* group, complicating some direct comparisons between them. Additionally, it is difficult to know how much their gender trajectories are actually impacted by receiving frequent exposure to gender diversity by living with their transgender sibling (and, thus, how we should think about the generalizability of their results).

Each youth participant in the *Recruited as Transgender* group has at most one sibling in the *Recruited as Siblings* group in this monograph. Like youths in the *Recruited as Transgender* group, youths in this group were between the ages of 3 and 12 at their first visit (except for one participant whose first participation was at 14); sometimes, participants in this group were younger than 3 when their (older) transgender sibling began participating in the study, and in these cases, the younger sibling sometimes “aged in” to the study and began participating after they turned 3 (if the transgender participant did not already have another sibling participating in the study). Conversely, if participants in the *Recruited as Transgender* group had siblings who were older than 12 at the child's first visit, we did not include them as age 12 was the upper‐bound for first enrollment. As a result of the asymmetric inclusion process, youths in the *Recruited as Siblings* group are, on average, younger than their siblings in the *Recruited as Transgender* group by 6.5 months.

##### II.3.1.4 Recruited as Cisgender

The *Recruited as Cisgender* group (*N* = 377) consists of youths who were living as cisgender as of their first participation (e.g., if these children were assigned male at birth, they were living as boys, and if they were assigned female at birth, they were living as girls). Youths in this group are not related to youths in the *Recruited as Transgender* group, but each participant was recruited to match the age (within 4 months at time of testing) and gender (not assigned sex) of a specific youth participant in the *Recruited as Transgender* group. While running the study, it became clear that youths in the *Recruited as Cisgender* sample were more likely to drop out or miss follow‐up contact attempts than youths in the *Recruited as Transgender* group, among which only one family formally dropped out in the first 6 years. For this reason, when youths in the *Recruited as Cisgender* group dropped out or if it became too difficult to reach them for a follow‐up visit, we recruited another cisgender youth (age‐ and gender‐matched to the *Recruited as Transgender* participant) to replace them. This approach meant that we could keep the number of families actively participating roughly equal between the *Recruited as Transgender* and *Recruited as Cisgender* groups. In this way, some participants (*N* = 316; due to an error we were missing one initial match) can be conceptualized as “original” matched comparison participants to the 317 *Recruited as Transgender* youths, while a smaller subset (*N* = 61) can be conceptualized as replacements for the original matched comparison group and/or their initial replacements. The latter sometimes joined the study at later ages (i.e., at 13 years old or above). In the remainder of this monograph, we report results for all youths in the *Recruited as Cisgender* group together (e.g., those in the original matched comparison group or replacements). The Supporting Information for this chapter includes information about demographics, study participation, and final gender and sexual orientation outcomes when examining only the original participants; the key take‐away is that results show the same patterns as are reported in the main text when restricting to the original sample.

#### II.3.2 Demographics of Participating Families

Below, we describe the racial‐ethnic, socioeconomic, ideological, and geographic characteristics of participating families (see Table 2 for full demographics). Although there is variation within and across the three participant groups, parents in our sample are disproportionately highly educated, upper‐income, and politically liberal. The youth are disproportionately White, non‐Hispanic, or multiracial. As of initial recruitment, our sample included youths from more than 40 U.S. states and 2 Canadian provinces, although the majority lived in states that were politically liberal and that, at the time of this writing, had not passed many anti‐LGBTQ+ laws.

**TABLE 2:** Demographic Information of Youths and Parents

	Recruited as Transgender	Recruited as Siblings	Recruited as Cisgender
Youth assigned sex at birth: *N* (%)			
Male	208 (66%)	125 (57%)	128 (34%)
Female	109 (34%)	93 (43%)	249 (66%)
Youth age as of latest visit (years): *M* (*SD*, range)	15.1 (*SD* = 3.04, *range:* 4–21)	14.0 (*SD* = 3.88, *range:* 5–22)	13.8 (*SD* = 3.60, *range:* 4–22)
Youth race‐ethnicity (parent‐reported): *N* (%)			
White, non‐Hispanic	219 (69%)	151 (69%)	255 (68%)
White, Hispanic	25 (8%)	19 (9%)	20 (5%)
Multiracial, non‐Hispanic	41 (13%)	24 (11%)	70 (19%)
Asian, non‐Hispanic	9 (3%)	4 (2%)	13 (3%)
Black/African, non‐Hispanic	5 (2%)	4 (2%)	4 (1%)
Other	14 (4%)	8 (4%)	7 (2%)
Not reported	4 (1%)	8 (4%)	8 (2%)
Primary parent race‐ethnicity: *N* (%)			
White, non‐Hispanic	258 (81%)	178 (82%)	274 (73%)
White, Hispanic	13 (4%)	7 (3%)	6 (2%)
Multiracial, non‐Hispanic	14 (4%)	10 (5%)	25 (7%)
Asian, non‐Hispanic	5 (2%)	4 (2%)	26 (7%)
Other	7 (2%)	4 (2%)	7 (2%)
Not reported	20 (6%)	15 (7%)	39 (10%)
Household annual income: *N* (%)			
Less than $25,000	11 (3%)	9 (4%)	6 (2%)
$25,001–$50,000	33 (10%)	21 (10%)	17 (5%)
$50,001–$75,000	64 (20%)	39 (18%)	43 (11%)
$75,001–$125,000	98 (31%)	72 (33%)	118 (31%)
More than $150,000	111 (35%)	77 (35%)	192 (51%)
Not Reported	0 (0%)	0 (0%)	1 (0%)
Household educational attainment: *N* (%)			
High school diploma	8 (3%)	6 (3%)	8 (2%)
Some college/Associate's degree	43 (14%)	26 (12%)	32 (8%)
College/Bachelor's degree	92 (29%)	60 (28%)	167 (44%)
Advanced degree (MA, MD, PhD, etc.)	165 (52%)	119 (55%)	156 (41%)
Other	5 (2%)	4 (2%)	2 (1%)
Not reported	4 (1%)	3 (1%)	12 (3%)
Household political ideology (1–7 scale): *N* (%)			
Liberal (1–2)	274 (86%)	184 (84%)	239 (63%)
Moderate (3–5)	40 (13%)	32 (15%)	122 (32%)
Conservative (6–7)	2 (1%)	2 (1%)	12 (3%)
Other	1 (0%)	0 (0%)	2 (1%)
Not reported	0 (0%)	0 (0%)	2 (1%)
Geographic location (at recruitment): *N* (%)			
US: Northeast	37 (12%)	22 (10%)	13 (3%)
US: Midwest	65 (21%)	50 (23%)	0 (0%)
US: South	57 (18%)	39 (18%)	0 (0%)
US: West	152 (48%)	102 (47%)	364 (97%)
Canada	6 (2%)	5 (2%)	0 (0%)
Primary parent gender: *N* (%)			
Man	25 (8%)	21 (10%)	34 (9%)
Woman	276 (87%)	187 (86%)	329 (87%)
Nonbinary or other	16 (5%)	10 (5%)	10 (3%)
Not reported	0 (0%)	0 (0%)	4 (1%)

*Note*: To protect participant privacy (and minimize the possibility of identifiability), we do not report demographics when fewer than 10 participants share it; these participants are reflected in the “Other” category. Household educational attainment, political ideology, and income are based on the family's earliest report of each.

We note that, by the last 2 years of recruitment (2015–2017), sign‐ups for participation outpaced our ability to test families. We therefore prioritized recruitment of families who had been underrepresented in our sample up to that point (e.g., those from lower‐socioeconomic‐status backgrounds, those with children of color, those from states that were not represented in the early sample) to expand our sample's diversity. As a result, not all families who initially signed up to participate were able to do so. Recruitment of new families ended once resources became limited and the team decided to utilize limited funds to prioritize follow‐ups rather than new recruitment.

### II.4 General Design and Methodology

#### II.4.1 Study Recruitment

Recruitment of participants in the *Recruited as Transgender* group began in the summer of 2013 and continued until December 2017, with recruitment rates increasing across this interval—only 47 participants (15%) were recruited in 2013–2014, while 270 participants (85%) were recruited in 2015–2017. Because participants in the *Recruited as Cisgender* group were sometimes replaced, and/*or Recruited as Siblings* participants joined the study later, these groups had new recruits through 2023. Transgender youths were recruited in many ways, including in‐person and online support groups for families with transgender children and camps or conferences for transgender youths. Additionally, some families in this group found our website themselves and signed up or heard about the study from the media or others in their network. Siblings of transgender youths were recruited using the same mechanisms as transgender youths (and were often—but not always—recruited into the study at the same time).

Youths in the *Recruited as Cisgender* group were primarily recruited through a participant pool at the University of Washington; a minority were recruited from a participant database at Princeton University. This recruitment strategy meant that youths in the *Recruited as Cisgender* group were primarily living in Washington State (with a small number living in New Jersey) when they joined the study, though some have since moved and continue to participate. In recruiting families, parents of children in all three groups were told that the project was a longitudinal study involving transgender youths prior to agreeing to participate. We knew that this information could influence the willingness of parents—particularly, those in the *Recruited as Cisgender* group—to participate. However, we made this decision both (a) for full transparency and (b) in the hopes of better matching the two sets of families—those in the *Recruited as Cisgender* and *Recruited as Transgender* groups—on variables that might otherwise bias one sample and not the other (e.g., parents' political orientation).

#### II.4.2 Study Administration

For all youths in the *Recruited as Transgender* group, participants' first visits were in‐person with the research team. This is also true for almost all youths in the *Recruited as Siblings* and *Recruited as Cisgender* groups, though these youths could also be added to the study via Zoom participation or an online survey (3% participated for the first time in this manner). For youths in the *Recruited as Transgender* and *Recruited as Siblings* groups, in‐person visits often took place at the family's home, in a private space within a public facility (e.g., a rented room at a public library, restaurant, or college), or in a private space at or near a camp, conference, or support group meeting for families of transgender youths. Visits with families in the *Recruited as Cisgender* group were mostly conducted in a developmental psychology research lab at the University of Washington. Meeting families in‐person for the first visit was intentional for the purpose of rapport‐building, given the potentially sensitive nature of the work, and because remote participation in the absence of a parent (which we wanted to provide to children for their own confidentiality) was not possible at the youngest end of the age spectrum. Until early 2020, subsequent sessions with youths 12 years and under (and their parents) were conducted in‐person in the locations previously described; after the onset of the COVID‐19 pandemic in 2020, all study sessions with youths 12 years and under were conducted via Zoom (who by then were older, and had more experience using online teleconferencing). Youths 13 years and older (beginning in 2019) and later 12 years and over (beginning in 2021) completed studies remotely via an unmoderated online survey. Youth assent (verbal for youths 3–8; written and verbal for youths 9–11 years or exclusively verbal for youths 9–11, depending on the institution; written for youths 12 years and above) and parental consent were obtained before youth participation. Youths and parents were both told that they could skip any questions or stop at any time, and all participants (youths and parents) received a toy, cash, and/or gift card (valued at $5–$25 per gift) for participating. The exact amounts and form of payment varied by participant age, length of the study, and year of participation. Youths who had reached 18 in the United States or 16 in Canada could participate without parent consent.

#### II.4.3 Parent Participation

All youths had at least one parent also participate in the study, and some youths had multiple parents who participated. Participation from more than one parent per youth was more common in the *Recruited as Transgender* and *Recruited as Siblings* groups compared to the *Recruited as Cisgender* group because we were often, for example, in the youth's home or at an event that the whole family attended, often on a weekend (as opposed to in a developmental psychology lab, where appointments often occurred on weekdays when one parent was usually working). To keep parent reporting similar across groups, and because in all groups some families had only one parent, we focus on reports from a single parent per child participant (i.e., the “primary parent”; henceforth, *parent*). The primary parent was defined as the parent who provided responses the greatest number of times over the course of the child's participation in the study so far. While our main analyses throughout the monograph include only the primary parent's report, on rare occasions, we include (and clearly label that we are including) data from other parents (e.g., when describing inter‐rater reliability for qualitative coding of write‐in responses from parents, we include data from both primary and secondary parents; we do so because responses from both were coded together). In general, results are extremely similar when using reports from primary or secondary parents, whose reports of their children's gender identities were in agreement in 98% of cases in which both parents participated. On extremely rare occasions, youths had more than two parents participate in the study (i.e., a stepparent in addition to two divorced parents); results from these tertiary and quaternary parents are not reported in the monograph.

#### II.4.4 Longitudinal Structure, Participation Frequency, and Attrition

Face‐to‐face visits (in person or on Zoom, primarily for youth 12 and under) occurred approximately every 1–3 years. The exact timing varied because, for the first 6 years of the study, the research team had to travel to meet with families. The team might, for example, travel to the Northeast for a week, driving to each family's home; however, if a given family was away that week, they would miss an appointment and not participate until the next time a team member was in the area (e.g., for a conference or personal travel). Once the youths are 12 or older, they and their parents are asked each year to participate in an annual online survey.

Occasionally, parent surveys were only given to a subset of participants (e.g., parents of youths in the *Recruited as Transgender* group, or parents whose children we had not seen in more than a year). Additionally, parents sometimes participated more frequently than once a year because parent‐only surveys were distributed to parents in 2015, 2017, 2018, and 2020; these might have been filled out close in time to a child's face‐to‐face visit. Since the beginning of TYP, a goal has been to follow the youths for 20 years (pending continued funding, feasibility, and continued participant interest). Summary statistics regarding participation frequency, duration of time in the study, and age at initial and most recent visits are presented in Table 3.

**TABLE 3:** Characteristics Regarding Recruitment and Participation of Each Group

		Recruited as Transgender	Recruited as Siblings	Recruited as Cisgender
Total *N* (Youths)		317	218	377
Recruitment Method		Conferences, Camps, Gender Clinics, Word of Mouth, Online Recruitment Through Lab Website	Conferences, Camps, Gender Clinics, Word of Mouth, Online Recruitment Through Lab Website	Participant Databases at the University of Washington and Princeton University
Number of visits in study: M (SD, range)		5.88 (*SD* = 1.84, *range:* 1–9)	4.64 (*SD* = 1.89, *range:* 1–9)	4.62 (*SD* = 2.02, *range:* 1–9)
Youth age at first visit (years): M (SD, range)		8.0 (*SD* = 2.36, *range:* 3–12)	7.8 (*SD* = 2.55, *range:* 3–14)	8.4 (*SD* = 2.37, *range:* 3–14)
Year of first participation	2013–2017: *N* (%)	317 (100.0%)	201 (92.2%)	319 (84.6%)
	2018–2023: *N* (%)	0 (0.0%)	17 (7.8%)	58 (15.4%)
Average time (years) between initial and most recent visit: *M* (SD, range)		7.03 (*SD* = 1.92, *range:* 0–10.25)	6.20 (*SD* = 2.42, *range:* 0–10.33)	5.42 (*SD* = 2.74, *range:* 0–9.92)
Participants with a study visit since 2020: *N* (%)		300 (95%)	200 (92%)	311 (82%)
Official attrition^a^	Youth opted out of study: *N* (%)	4 (1.3%)	2 (0.9%)	16 (4.2%)
	Youth deceased: *N* (%)	0 (0.0%)	2 (0.9%)	0 (0.0%)
	Youth lost to contact: *N* (%)	2 (0.6%)	2 (0.9%)	0 (0.0%)

*Note*: We define participation (first, most recent, and since 2020) with respect to visits at which either a youth reported about themself or a parent reported about that youth. In the table, a youth is considered opted out if the youth and their parent(s) contacted the research team to tell us that they are no longer participating. If a youth has opted out while a parent has remained in the study, we continue to learn about these youths from their parent and the youth is therefore not considered opted out in the above table (this applies to 5 *Recruited as Transgender* youths, 2 *Recruited as Cisgender* youths, and 3 *Recruited as Siblings* youths). Of the two deceased siblings, one died by suicide and one did not. The four youths lost to contact (indicating that the research team has no way to contact these families and has stopped trying) come from two families: one family in which both participating parents have passed away, and one family for which we have no active contact information.

^a^ Participants in the “Official attrition” row are participants whose involvement with the study has officially ended because they explicitly opted out, the youth is deceased, or the research team has no way of contacting the youth's parents. Sometimes, *Recruited as Cisgender* youths became hard to reach or schedule and were replaced, as detailed in the main text; however, these cases are not counted as “Official attrition” in the table.

Retention in the study has generally been high, with 89% of participants (and 95% of all *Recruited as Transgender* participants) having contributed a study visit from either the youth or a parent between January 2020 and February 2024—a period of several years after their initial participation. However, we have experienced (slightly) higher rates of attrition in the *Recruited as Cisgender* and *Recruited as Siblings* groups relative to the *Recruited as Transgender* group. The most consequential result of differential attrition rates among the three groups is that youths in the *Recruited as Cisgender* group (and, to a lesser extent, the *Recruited as Siblings* groups) have participated fewer times and over a shorter timespan than youths in the *Recruited as Transgender* group (see Table 3). When answering questions about how likely youths in the three groups are to show change (see Chapter IV), we attempt to mitigate the effect of this differential attrition by statistically controlling for the amount of time a youth has been in the study. However, this does not fully address all of the complications introduced by differential attrition rates; in Chapter VII, we more thoroughly discuss the robustness of various results from the monograph in light of the uncertainty introduced by attrition. We do note that the number of participants who are officially no longer in the study (see “Official attrition”, Table 3)—that is, they explicitly opted out, or the youth is deceased, or the research team has no way of contacting the youth's parents—is exceedingly low; these figures are represented in the bottom row of Table 3 and are much lower than the number of youths who are still officially enrolled in the study (and whom we still attempt to contact) but who stopped responding to our correspondence years ago.

For the most part, measures administered to youths and parents at any given point in time have been the same for participants in all three *recruitment groups*. There are exceptions to this: for example, in face‐to‐face visits, parents of transgender children were asked to describe when in development certain transgender‐specific milestones (e.g., age of social and medical transition, etc.) occurred. Additionally, in online surveys since 2015, certain questions are displayed to youths and parents only in cases when the youth is identified as transgender or gender diverse at that visit (e.g., questions about medical transition or identity‐specific bullying), meaning that youths in the *Recruited as Transgender* group are more likely to receive and respond to these questions. However, most of the core identity measures we report on in this manuscript were overwhelmingly asked of youths and parents in all three groups.

#### II.4.5 Consideration of “Recruitment Gender”

Throughout this monograph, we refer to, and organize some of our results by, *recruitment gender*, which refers to the gender that a child was living as in their everyday life at the time of their first visit in the study. For youths in the *Recruited as Transgender* group, this was determined by parents' (and in cases of older youths, children's) reports about pronoun use; for youths in the *Recruited as Cisgender* and *Recruited as Siblings* groups, this always aligned with the binary gender associated with the child's assigned sex. We refer to this categorization as “*recruitment gender*” to indicate that it is based on the researchers' assessment of the youth's gender at recruitment (as described above) and not based on youths' responses on any of the measures reported as dependent variables in this monograph; more information on alternative approaches for defining youths' starting‐point gender identities is provided in Chapter IV (subsection titled “Technical Details”). All youths in the study were recruited as boys or girls (see subsection title *Consideration of “Nonbinary” Category* in *Participants*, above, for discussion of why youths were not recruited as nonbinary).

### II.5 Additional Detail on the *Recruited as Transgender* Group

**Figure 2:**
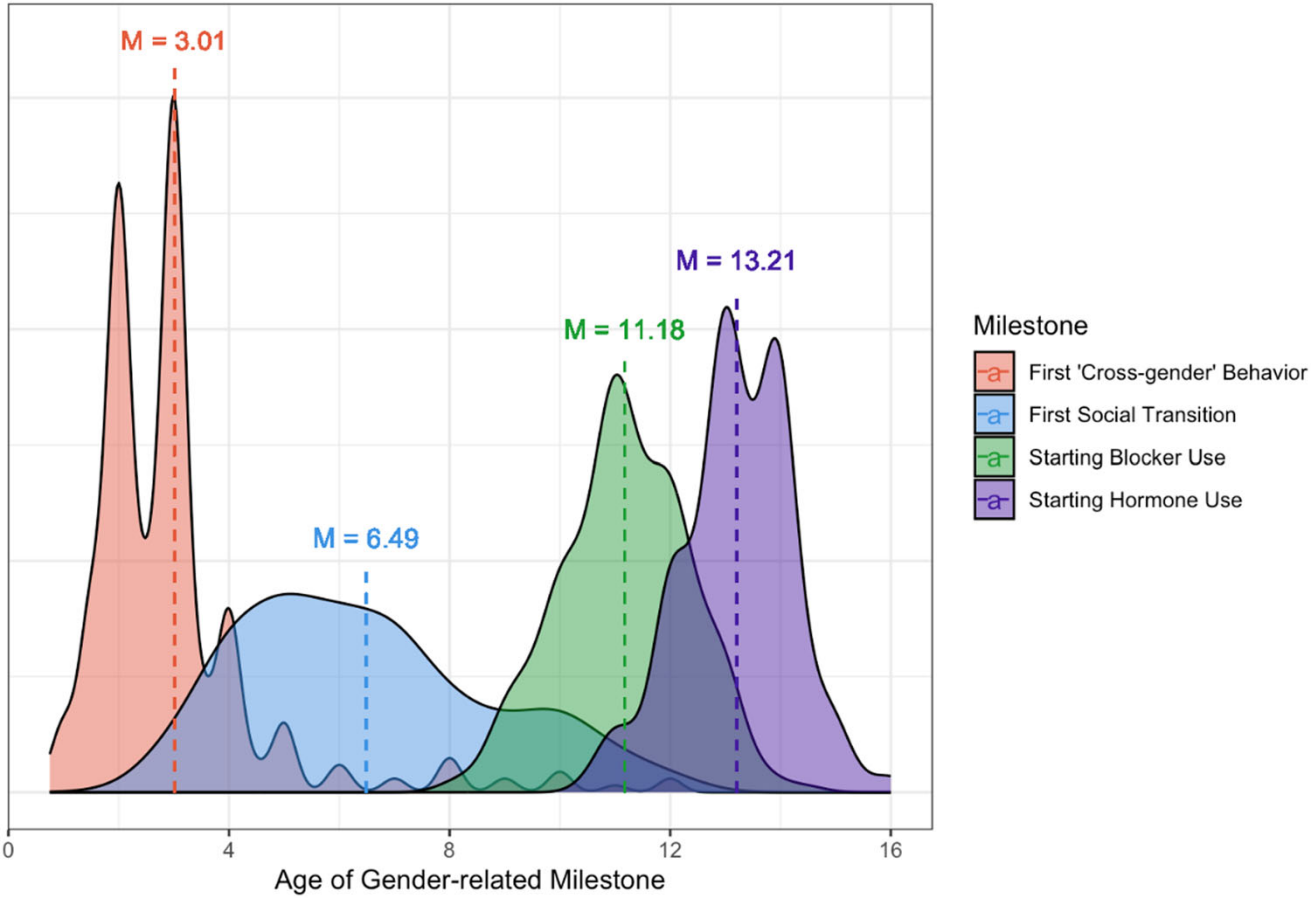
Relevant gender‐related milestones among *Recruited as Transgender* youths. *Note*: “Blocker use” refers to gonadotropin‐releasing hormone (GnRH) agonists, medications that suppress the physiological changes associated with endogenous puberty (e.g., breast growth in assigned females, voice deepening in assigned males). “Hormone use” refers to hormonal treatments that induce physical changes associated with exogenous puberty (e.g., testosterone causing voice‐deepening and facial hair growth in transgender boys, estrogens causing breast development and body fat redistribution in transgender girls). Distributions of First “Cross‐gender” Behavior and First Social Transition include all 317 youths in the *Recruited as Transgender* group, whereas distributions for Starting Blocker Use and Starting Hormone Use do not, because some youths will never access these treatments, and others will (likely) begin to access them after the time period reported in this monograph.

In the final section of this chapter, we provide additional information on youths in the *Recruited as Transgender* group to help situate our description of them in later chapters in the context of their lived experiences. First, we provide descriptive data on several relevant milestones, including when parents first noticed behaviors “opposite” to those associated with children's sex assigned at birth, when children socially transitioned, and when (or if) children received gender affirming medical care (see Figure 2 for a summary). We additionally characterize the immediate social environments of youths in this participant group by providing youths' and parents' assessment of social support from others.

#### II.5.1 Early Reports of “Cross‐Gender” Behavior

Often, parents of youths in this group reported that they had noticed what some might consider “cross‐gender” behavior early in their child's life. That is, children engaged in behaviors that were counter‐stereotypic given their child's assigned sex. Examples include behaviors like an assigned male child placing a belt over a t‐shirt as a “dress” while wearing their mother's high heels, or an assigned female child getting in the boys' line when their preschool class was organizing for a trip to the bathroom. Although nearly all children exhibit *some* “cross‐gender” behaviors, the degree or frequency of these behaviors among children in this *recruitment group* was often notable. Beginning at the first visit, we asked parents to report the *age* of the earliest signs of “cross‐gender” behavior they observed and then to describe *which* “cross‐gender” behaviors their children engaged in. In general, parents reported observing “cross‐gender” behavior in the years before their children made a social transition: the mean age at which parents recalled first observing “cross‐gender” behavior was 3 years (*SD* = 1.69, range: 9 months–12 years)—roughly 3.5 years before the average age at which children transitioned. Nine percent transitioned 7 or more years later, 28% transitioned 5 or more years later, 61% transitioned 3 or more years later, and 94% transitioned 1 or more years later.

To gain an understanding of the nature of the children's “cross‐gender” behavior, we coded the responses that parents provided when asked to describe their children's earliest “cross‐gender” behavior. We inspected the qualitative data and then coded each response for several of the themes we noted: (1) toys/interests, (2) play/activity, (3) media, (4) self‐portrayals or pretend play, (5) physical appearance, (6) temperament, (7) social affiliations, (8) gender identity/names, and (9) color. In the majority of cases (86%), parents' responses described *affirmative* behaviors (i.e., what the child *did* do); in all other cases (14%), parents' responses described *negated* behaviors (e.g., “refused to wear any clothes he considered 'girl‐y',” “no interest in cars, trains, vehicles”). Two coders independently read each response and coded it for the presence or absence of each of the themes listed above (responses received a “1” for each theme referenced and a “0” for each theme not referenced; themes were not mutually exclusive, so a given response could receive a “1” for multiple themes). Percentage agreement for each theme was high (agreement: 92%–98%). As shown in Table 4, the large majority (86%) of “cross‐gender” behavior descriptions included a reference to physical appearance (e.g., “dress‐up in stereotypical girl costumes), and about half (49%) mentioned children's toys or interests (e.g., “playing with trucks”); all other themes were present in less than a quarter of parents' responses.

**TABLE 4:** Themes Present in Parents' Reports of Recruited as Transgender Youths' Early “Cross‐gender” Behavior

Theme	Example of Response Containing This Theme	% Responses
Physical appearance	“dress‐up in stereotypical girl costumes”, “getting boy‐style haircuts”	86
Toys or interests	“playing with trucks”, “wanted to play with Barbie dolls”	49
Gender identity or name	“at 3, she said she was a girl”, “he kept changing his name to a masculine name”	21
Colors	“drawn towards pink”, “avoidance of pink”	18
Self‐portrayals or pretend play	“being the daddy during play”, “she would draw herself as female”	17
Social affiliations	“female friend preference”, “always playing almost exclusively with boys”	16
Play or activities	“football”, “only playing house”	15
Media or public personas	“desire to read only stories with female protagonists”, “being into superheroes”	12
Temperament	“more feminine demeanor”, “he showed the boys that he can be as strong as them”	6

One important note: While it was not systematically recorded by the research team, quite frequently parents answering this question would point out that they did not perceive particular behaviors as “only for boys” or “only for girls” (to the contrary, parents and youths in this study overwhelmingly reject prescriptive gender stereotypes; deMayo, Kahn‐Samuelson, et al., 2022). Instead, many parents were clear that the behaviors described in response to this question were ones that were *stereotypically* or *culturally* associated with one gender or the other. Furthermore, many parents spontaneously asserted that they had not thought of these behaviors, at the time they occurred, as an indication of the child's gender identity; many instead assumed their child was simply gender nonconforming or, in some cases, thought their child might grow up to be gay or lesbian. Many parents reported not even knowing that a child could be transgender at the time their child was first exhibiting these behaviors.

#### II.5.2 Youths' Social Transitions

The age at which children socially transitioned for the first time spans a wide range, from as young as 2 years to as old as 12 years. However, children's first social transitions tended to be clustered around early‐to‐middle childhood: the mean age of first transition in our sample is 6.49 years (*SD* = 2.26 years) and the modal age at which children first transitioned is 5 years (often at the start of kindergarten). Separately, a subset of parents (84%) responded to a question asking when their child first *told* them that they did not identify as the gender the parent had assumed (i.e., the gender associated with the child's sex assigned at birth). The age at which parents reported learning that their child identified with a gender other than that associated with their assigned sex ranged from 1 and 13; in the majority of cases (71%), parents said they learned this before their children turned 6. We suspect the two parents indicating learning about their child's identification when the youth was 13 were the result of reporting or memory errors, as these youth had socially transitioned at age 12 and these reports occurred at later visits.

#### II.5.3 Youths' Medical Transitions

Puberty blockers suppress the physiological changes brought on by endogenous puberty, while gender‐affirming hormones cause the development of secondary sex characteristics associated with the other binary gender (e.g., facial hair growth in transgender boys, breast development in transgender girls). The majority of youths in the *Recruited as Transgender* group started puberty blockers (86%) and/or gender‐affirming hormones (68%) at some point during the study. Within the *Recruited as Transgender* group, 16% started blockers and continue to use them without having begun gender‐affirming hormones, and 66% started both blockers and gender‐affirming hormones and continue to use gender‐affirming hormones (roughly one‐third of these youths have stopped using blockers). The remaining youths who medically transitioned started blockers and then stopped (1%), started blockers and gender‐affirming hormones and stopped both (1%), started blockers and gender‐affirming hormones but stopped the latter (1%), or never started blockers but started hormones (1%). The mean age at which youths in our sample began blockers was 11.18 years (*SD* = 1.15, *range*: 8–14.5 years), and the mean age at which they began taking gender‐affirming hormones was 13.21 years (*SD* = .99, *range*: 10.5–16 years). More details about this sample's experiences with gender affirming medical care are reported in Olson et al. (2024).

#### II.5.4 Youths' Social Support

A notable aspect of life for youths in this group is the high level of social support for their gender identity and/or expression. Of youths who provided data on social support measures (74% responded to this measure between 2021 and 2023), the mean level of support reported at youths' most recent visit—on a scale of 1 (not accepting at all) to 7 (fully accepting)—was 6.48 (*SD* = .69, *range*: 3.11–7.00). This value reflects the average level of social support from parents, others living at home, extended family not living at home, closest friends, other friends, peers/acquaintances, teachers, co‐workers, and other unrelated adults (e.g., coaches, religious leaders) when youths last answered this question (*α* = .92; youths only reported support from people who know their transgender or gender‐diverse identity). Parents were also asked to report on the extent to which *they* and those around them—the same groups that children were asked about—were accepting of their child's current gender identity and expression (on the same 1–7 scale). At the last visit on which parents provided this information (in 90% of cases, between 2021 and 2023), the mean level of support parents reported across domains (*α* = .83) was 6.69 (*SD* = .44, *range*: 4.33–7.00). For youths' and parents' social support ratings separated by domain (e.g., parents, teachers), see Table 5. Altogether, these data suggest that the youths in this sample overall experience very high levels of social support: both youths and parents provided estimates well above the midpoint of the scale (although parents' estimates were higher than youths'; *t*(234) = −4.13, *p* < .001). The current results are important to understand within the context of these highly supportive environments, as research suggests that many young transgender youths do not have much social support and many face high levels of social rejection, including from their parents (e.g., Eisenberg et al., 2017).

**TABLE 5:** Perceived Social Support of Recruited as Transgender Youths (Youths' and Parents' Most Recent Report)

Social Support Domain	Youth‐Reported	Parent‐Reported
Parents	*M* = 6.87, *SD* = .54, *range:* 3–7 (*n* = 235)	*M* = 6.99, *SD* = .08, *range:* 6–7 (*n* = 317)
Others living in your home	*M* = 6.85, *SD* = .55, *range:* 3–7 (*n* = 207)	*M* = 6.96, *SD* = .23, *range:* 5–7 (*n* = 246)
Extended family not living at home	*M* = 6.01, *SD* = 1.36, *range:* 2–7 (*n* = 227)	*M* = 6.18, *SD* = 1.22, *range:* 1–7 (*n* = 258)
Closest friends	*M* = 6.83, *SD* = .56, *range:* 4–7 (*n* = 196)	*M* = 6.95, *SD* = .30, *range:* 4–7 (*n* = 220)
Other friends	*M* = 6.28, *SD* = 1.13, *range:* 2–7 (*n* = 152)	*M* = 6.69, *SD* = .78, *range:* 1–7 (*n* = 180)
Peers/acquaintances	*M* = 5.62, *SD* = 1.53, *range:* 1–7 (*n* = 134)	*M* = 6.15, *SD* = 1.22, *range:* 1–7 (*n* = 149)
Teachers	*M* = 6.36, *SD* = 1.09, *range:* 2–7 (*n* = 131)	*M* = 6.57, *SD* = .95, *range:* 1–7 (*n* = 214)
Coworkers	*M* = 6.22, *SD* = 1.52, *range:* 1–7 (*n* = 59)	*M* = 6.57, *SD* = 1.02, *range:* 1–7 (*n* = 95)
Other unrelated adults	*M* = 6.18, *SD* = 1.40, *range:* 1–7 (*n* = 107)	*M* = 6.46, *SD* = 1.02, *range:* 2–7 (*n* = 128)

*Note*: Social support is on a scale from 1 (lowest support) to 7 (highest support).

### II.6 Summary

The Trans Youth Project, which began in 2013 and is ongoing, has followed a cohort of socially transitioned transgender children (*Recruited as Transgender* group), their siblings (*Recruited as Siblings* group), and an age‐ and gender‐matched group of community‐recruited cisgender children (*Recruited as Cisgender* group). Participants have, as of February 2024, been studied for approximately 6 years, and data from youths and their parents have been collected in online surveys, in‐person visits in participants' homes or in a university lab, and Zoom interviews with experimenters. The transgender youths in our sample generally experience very high levels of social support from their families; they initially socially transitioned at age 6.5, and most have accessed puberty blockers and gender‐affirming hormones in adolescence.

## III Assessing Youth‐ and Parent‐Reported Gender at Each Visit

### III.1 Chapter Highlights

- Youths and parents in the Trans Youth Project have reported about the youths' gender identities on a heterogeneous set of measures since 2013; this chapter provides an overview of the two‐step process we used to take these highly variable data and synthesize them so that we could derive a single description of each youth's gender at each time point in which they participated—which we then use to assess overall stability and change in the coming chapters. For these topics, see **Deriving Visit‐Level Codes from Responses to Individual Measures**.
- How do we know that these single descriptions of the youths' gender identities were accurate or face valid? We provide evidence in this chapter suggesting that they are; see **Evidence of Validity of Five‐Category Coding Process**.
- We briefly discuss how much parents and youths agreed or disagreed in their contemporaneous reports of the youth's gender; for this topic, see **Overall Agreement Between Youth‐ and Parent‐Reported Gender‐at‐Visit**.

### III.2 Introduction

In this monograph, we are interested in characterizing stability and change in youths' gender identities and sexual orientations in the 11 years (2013–2024) that our research team has been running the Trans Youth Project. In this time, societal notions of gender have changed (see Chapter I for a broad overview of societal changes regarding gender and sexual orientation that have occurred in the past several decades) and youths in the study have grown from children into adolescents and young adults. As concepts of gender have become more nuanced, we have had to shift our assessment methods. Within a single session, we often asked about participants' gender with multiple heterogeneous measures (e.g., continuous and categorical, forced choice and select‐all‐that‐apply; open‐ and closed‐ended, etc.), some using older terms (to allow for some continuity in our measures) and some using more updated terms (to better match how participants were describing themselves in the moment). However, to address the central questions of this monograph, which have to do with characterizing participants' trajectories of identity over time, we had to convert this heterogeneous set of answers into a common metric for assessing stability and change.

In this chapter, we describe how we converted youths' and parents' responses on a diverse array of identity measures from over a decade of research into a determination of each youth's gender identity at each visit in which either they or their parent participated. We begin by providing an overview of our approach to categorizing each youth's gender at each visit based on their responses on the various identity measures. Then, we provide evidence regarding the validity of our categorizations of participants' responses to individual measures and our coding of youths' gender at each visit. Finally, we briefly describe agreement between youth‐ and parent‐report when they participated at the same time point.

### III.3 Deriving Visit‐Level Codes from Responses to Individual Measures

One of our central questions in this monograph concerns stability and change in gender identity. To address this question, we need to know youths' gender identities (according to them and/or their parents) at each visit in which they or their parents participated. Here, we describe how we derived a single gender‐at‐visit categorization for each youth, at each visit, according to each respondent (i.e., the youth themselves or their parents).

In the majority of study visits, each participant (i.e., the youth and/or any parents that participated) was asked to describe the youth's gender identity on at least one measure; in many visits, participants received several gender identity measures. These measures included (1) continuous identity scores on a 0–100 scale (see “*Continuum* Measure” in “Evidence of Validity of Visit‐Level Codes,” later this chapter), (2) discrete categorical measures, and (3) open‐ended measures. We generally relied on the categorical and open‐ended measures to derive determinations of youths' gender identities at each visit (according to each respondent); we only considered continuous scores (along with the youths' assigned sex and *recruitment gender*, see Chapter II for definitions) in rare cases when attempting to resolve ambiguous responses on categorical or open‐ended measures. Youth‐report and parent‐report categorical and open‐ended measures are shown in Tables 6 and 7, respectively.

**TABLE 6:** Summary of youth self‐report measures

Measure	Wording/Description	Purpose in this Monograph	Responses Per Participant	Age Range Across All Visits (Years)	Range of Years Measure was Asked	Youths Who Never Gave a Response to Measure (*N*)	Youths Who Gave a Response to Measure on 1 Visit (*N*)	Youths Who Gave a Response to Measure on 2 or More Visits (*N*)
*Continuum* from “boy” to “girl”	“Some people feel they are a boy, some people feel they are a girl, and some people feel they are somewhere in between a boy and a girl. Indicate the place you think best shows how you feel on the inside.” (response provided on a line with labels “boy” and “girl” at either end)	Provided convergent validity with categorical measures; used to predict later identity outcomes	Mean = 2.5, range = [0,6]	Mean = 12.6, range = [3, 22]	[2016, 2024]	100	169	643
*Measure 1*: Three‐item multiple choice^a^	“Are you a boy, a girl, or something else?” [if “something else” was selected, options of “both”, “neither”, “it changes”, or “I don't know” were offered]	Used to determine gender at visit	Mean = 2.5, range = [0,6]	Mean = 12.3, range = [3, 22]	[2016, 2024]	81	173	658
*Measure 2*: Six‐option multiple choice^b^	“What gender do you identify as?” [options: boy, girl, nonbinary, gender fluid, agender, I prefer a different term (please describe)]	Used to determine gender at visit	Mean = 0.1, range = [0,1]	Mean = 14.5, range = [13, 18]	[2019, 2020]	801	111	0
*Measure 3*: Seven‐option multiple choice with focus on modality^b^	“Which of these terms do you most prefer to use to describe your gender identity?” [options: transgender, gender nonconforming, nonbinary, cisgender, gender fluid, agender, I prefer a different term (please describe)]	Used to determine gender at visit	Mean = 0.1, range = [0,1]	Mean = 14.5, range = [13, 18]	[2019, 2020]	812	100	0
*Measure 4*: Yes‐or‐no questions^c^	Asked to answer “yes”, “no”, “skip”, or “I don't know” to the following: “Are you a boy?”; “Are you a girl?”; “Are you nonbinary?”	Used to determine gender at visit	Mean = 1.5, range = [0,3]	Mean = 14.6, range = [5, 22]	[2021, 2024]	229	217	466
*Measure 5*: Select‐all‐that‐apply question	“Which of these terms describe your gender identity? (select all that apply)" [options: agender, boy/man, cisgender, girl/woman, gender nonconforming, genderfluid, nonbinary, transgender, I prefer a different term (please describe)]	Used to determine gender at visit	Mean = 1.3, range = [0,3]	Mean = 15.3, range = [12, 22]	[2021, 2024]	316	215	381
*Measure 6*: Open‐ended description of gender	“In your own words, how would you describe your gender identity?”	Used to determine gender at visit	Mean = 1.2, range = [0,3]	Mean = 15.3, range = [12, 22]	[2021, 2023]	337	224	351

^a^ An alternative version of Measure 1 was used prior to 2017 which is not included in this monograph given evidence that it was too imprecise and confusing for children; see Supporting Information for details.

^b^ Measures 2 and 3 were only administered in one annual online survey (in 2019) and only given to youths in the *Recruited as Transgender* and *Recruited as Cisgender* groups (*Recruited as Siblings* group was not recruited that year).

^c^ Use of this measure in the TYP was first reported in Wittlin et al. (2024).

**TABLE 7:** Summary of Parent‐Report Gender Measures

Measure	Wording/Description	Purpose in Study	Responses From Parent Per Youth Participant	Youth Age Range Across All Visits (Years)	Range of Years Measure was Asked	Youths Whose Parent Never Responded to Measure (*N*)	Youths Whose Parent Responded to Measure on 1 Visit (*N*)	Youths Whose Parent Responded to Measure on 2 or More Visits (*N*)
*Measure 7*: Three‐item multiple choice	“What is your child's gender (i.e., how your child identifies even if it is not your child's biological sex)”? [options: boy, girl, other]	Used to determine gender at visit	Mean = 2.6, range = [0,7]	Mean = 9.4, range = [3, 18]	[2013, 2024]	5	183	724
*Measure 8*: Six‐option multiple choice	“What gender does your child identify as?” [options: boy, girl, nonbinary, gender fluid, agender, I prefer a different term (please describe)]	Used to determine gender at visit	Mean = 1.6, range = [0,4]	Mean = 15.4, range = [11, 22]	[2019, 2023]	243	208	461
*Measure 9*: Seven‐option multiple choice with focus on modality	“Which of these terms do you most prefer to use to describe your child's gender identity?” [options: transgender, gender nonconforming, nonbinary, cisgender, gender fluid, agender, I prefer a different term (please describe)]	Used to determine gender at visit	Mean = 1.6, range = [0,4]	Mean = 15.4, range = [11, 22]	[2019, 2023]	245	214	453
*Measure 10* a: Select‐all‐that‐apply with 12 options	“Please mark all of the following descriptions that you would use to describe your child. Feel free to mark multiple items.” [options: both genders, neither gender, cisgender boy, cisgender girl, female‐to‐male transgender, male‐to‐female transgender, bisexual, gay male, lesbian, gender‐nonconforming natal male, gender‐nonconforming natal female, other]	Used to determine gender at visit	Mean = 0.2, range = [0,1]	Mean = 8.5, range = [3, 14]	[2015, 2016]	734	178	0
*Measure 11* b: Open‐ended	“How would you best describe your child's gender? Some example terms we've heard are: princess/pink boy, genderqueer, boy who likes girl stuff, tomboy, etc. Pick your favorite term to describe your child and explain if that's helpful.”	Used to determine gender at visit	Mean = 0.4, range = [0,1]	Mean = 9.3, range = [3, 15]	[2017, 2017]	577	335	0

*Note*: This table reflects measurement information based on the primary parent answering the relevant measure; information about other parents is not represented.

^a^ Measure 10 was only administered to parents once (in a 2015 survey) and was not asked of parents of youths in the *Recruited as Cisgender* group.

^b^ Measure 11 was only administered to parents once; it was only asked to parents of youths in the *Recruited as Cisgender* or *Recruited as Siblings* youths, or *Recruited as Transgender* youths if the parent indicated their child was cisgender at the time of completing the survey.

#### III.3.1 Deriving a Five‐Category Gender‐at‐Visit Code for Each Respondent

Our goal was to derive a single gender‐at‐visit code for each respondent at each visit based on whatever responses they provided to Measures 1–6 (youths, see Table 6) or 7–11 (parents, see Table 7); detailed information about participant responses to these measures appears in the Supporting Information for this chapter. While discretizing youths' identities in this way is in tension with a desire to let participants self‐describe their identity—and therefore may lose some of their nuance—this was necessary to derive a quantitative estimate of stability and change in gender identity over time. We adopted an initial five‐category coding scheme: boy, girl, gender diverse (e.g., nonbinary, agender), boy‐expansive (a mix of boy identification and gender‐diverse identification in the same response, e.g., “nonbinary boy”), and girl‐expansive (a mix of girl identification and gender‐diverse identification in the same response, e.g., “demi‐girl”).

**Figure 3:**
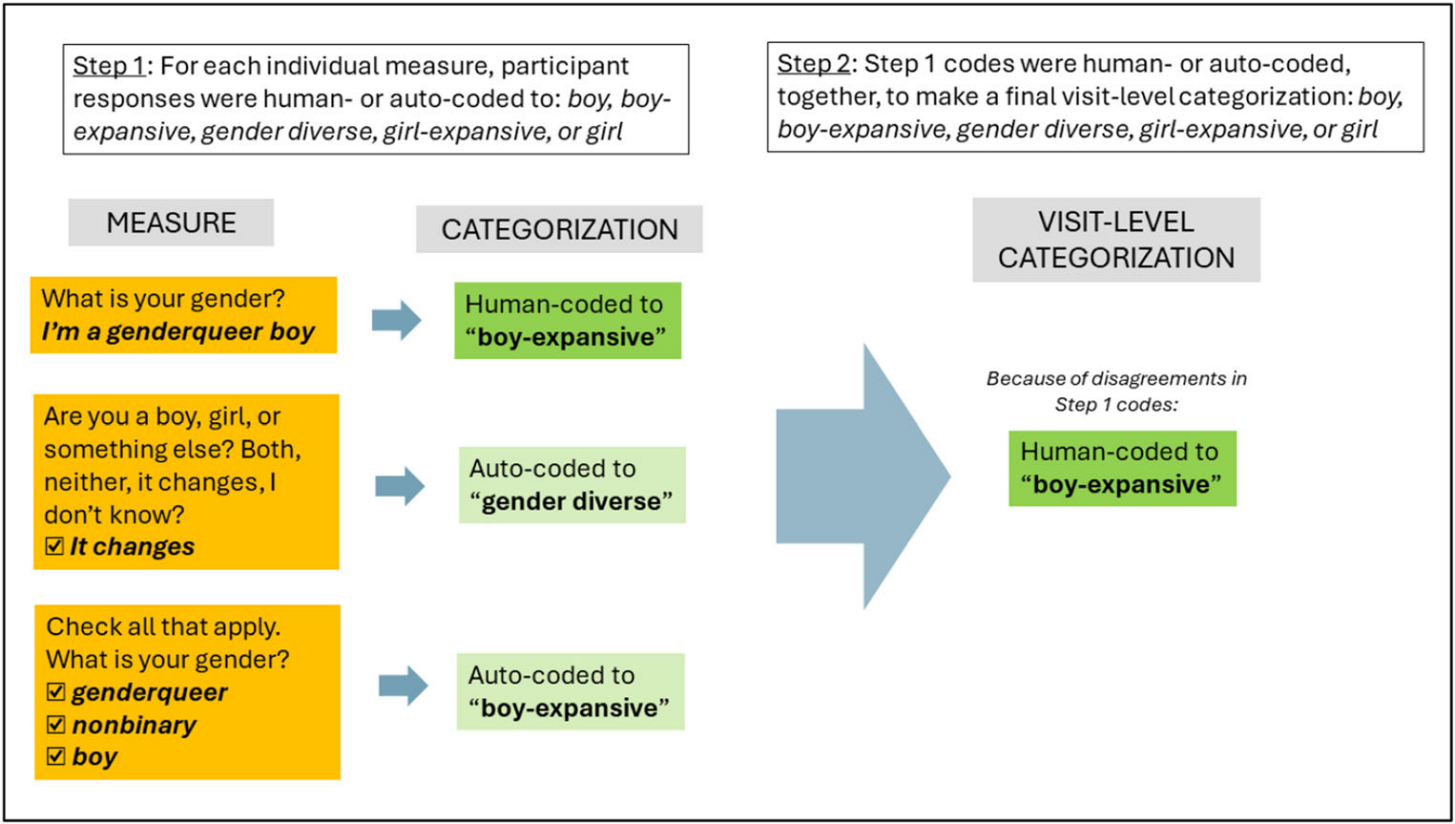
Example of Step 1 and Step 2 gender‐at‐visit coding.

There were two main steps involved in turning youths' and parents' responses on individual measures into visit‐level determinations of the youth's gender identity in this 5‐category scheme (see Figure 3 for an example of this two‐step process). In Step 1, we coded each response to each individual measure into one of the aforementioned five categories; if a participant's response on a measure was not sufficiently informative to reasonably infer which category in the 5‐category scheme would be most appropriate, the response was deemed “uncodable.” Next, in Step 2, we aggregated all the responses that a participant had given across gender measures in a particular visit to a single code in the 5‐category scheme. Both steps sometimes involved qualitative review of participant responses by two human coders, a process we describe below.

#### III.3.2 Qualitative Coding Procedure

All qualitative coding of gender identity information was handled by the same two coders, who were both in their early‐to‐mid‐20s (i.e., born around the year 2000); one is a genderqueer person from South Asia, the other is a white North American woman.

##### III.3.2.1 Qualitative Coding in Step 1

In Step 1 (coding participants' responses on individual measures into the five‐category coding scheme), qualitative coding was sometimes necessary to determine what code in the five‐category scheme should be assigned to a participant's response on a single measure. Most commonly, qualitative coding was used in Step 1 when a measure gave respondents an opportunity to write in their own term describing their or their child's gender, as was the case in youth‐report Measures 2, 3, 5, and 6, and all parent measures (7–11). For Measure 10, parents' responses were examined by human coders even in some cases in which parents did not write in their own term, because parents sometimes selected a combination of answer choices that were not straightforward enough to be coded using pre‐programmed categorization rules (e.g., a parent selected “Gender‐Nonconforming Natal Male,” “Both Genders,” “Neither Gender,” and “Gay Male” to describe their child).

Coders reviewed responses independently and convened after examining no more than 250 responses at a time to compare codes; any disagreements were resolved through discussion. They did not have access to youths' assigned sex except in cases when a response was only interpretable with respect to it (e.g., a respondent wrote in “binary transgender” or “cisgender”). Example responses for each of the five categories are shown in Table 8, and more details about how 5‐category determinations were made for each measure (including interrater reliability) are shown in Tables 9 (youth‐report) and 10 (parent‐report).

**TABLE 8:** Examples of Codes in the 5‐Category Scheme

Categorization	Example Items
Boy	“Boy”, “cisgender man”, “I am a boy that uses he/him pronouns”
Boy‐expansive	“Nonbinary boy”, “gay transmasculine individual”, “demi‐boy”
Gender diverse	“Non binary”, “gender‐fluid”, “gender creative”
Girl‐expansive	“Trans feminine mtf nonbinary”, “kinda girl kinda nonbinary”, “demi‐girl”
Girl	“Girl”, “female”, “trans girl”
Uncodable	“Confusing”, “I don't know”

**TABLE 9:** Descriptions of How Raw Responses on Youth Self‐Report Measures Were Recoded Into 5‐Category Scheme

Measure	Response Options	Response Coded as:					Responses Requiring Human coding (*N*)	Inter‐Rater Reliability (*κ*)
		Boy	Boy‐Expansive	Gender Diverse	Girl‐Expansive	Girl		
*Measure 1*	Select one of: boy, girl, I don't know, Both, Neither, or It changes	Selecting “boy”	NA	Selecting “I don't know”, “Both [boy and girl]”, “Neither” [boy nor girl]”, or “It changes”	NA	Selecting “girl”	NA	NA
*Measure 2*	Select one of: boy, girl, nonbinary, gender fluid, agender, I prefer a different term	Selecting “boy”, or self‐reporting a term recoded as boy	Self‐reporting a term recoded as boy‐expansive	Selecting “nonbinary”, "gender fluid”, or “agender”, or self‐reporting a term recoded as gender diverse	Self‐reporting a term recoded as girl‐expansive	Selecting “girl”, or self‐reporting a term recoded as girl	3	Perfect agreement
*Measure 3*	Select one of: transgender, gender nonconforming, nonbinary, cisgender, gender fluid, agender, I prefer a different term	Self‐reporting a term recoded as boy	Self‐reporting a term recoded as boy‐expansive	Selecting “gender fluid”, “nonbinary”, “agender”, or “gender nonconforming” or self‐reporting a term recoded as gender diverse	Self‐reporting a term recoded as girl‐expansive	Self‐reporting a term recoded as girl	7	Perfect agreement
*Measure 4*	Select “yes” or “no” to “Are you a”: Boy, Girl, Nonbinary	Selecting “Yes” on “Boy” and selecting “No” on (or skipping) “Nonbinary” and “Girl”	Selecting “Yes” on “Boy” and “Nonbinary” and selecting “No” on (or skipping) “Girl”	Selecting “Yes” on “Nonbinary” and selecting “No” (or skipping) both binary genders; Selecting “Yes” on “Nonbinary” and selecting “Yes” on both binary genders; Selecting “Yes” on both binary genders.	Selecting “Yes” on “Girl” and “Nonbinary” and selecting “No” on (or skipping) “Boy”	Selecting “Yes” on “Girl” and selecting “No” on (or skipping) “Nonbinary” and “Girl”	NA	NA
*Measure 5* a	Select all that apply from: agender, boy/man, cisgender, girl/woman, gender nonconforming, genderfluid, nonbinary, transgender, I prefer a different term	Selecting “Boy/Man” and none of the gender‐ diverse options^b^, or self‐reporting a term recoded as boy	Selecting “Boy/Man” and at least one of the gender‐ diverse options^b^, or self‐reporting a term recoded as boy‐expansive	Selecting “agender”, “gender nonconforming”, “genderfluid”, or “nonbinary” and neither of the binary gender options; Self‐reporting a term recoded as gender diverse	Selecting “Girl/Woman” and at least one of the gender‐ diverse options^b^, or self‐reporting a term recoded as girl‐expansive	Selecting “Girl/Woman” and none of the gender‐ diverse options^b^, or self‐reporting a term recoded as boy	45	0.69
*Measure 6*	Open‐ended description	All responses were reviewed by human coders					1078	0.93

^a^ Coding decisions were made without regard to whether youths selected “cisgender” or “transgender”, which we did not use to make classification decisions.

^b^ Gender‐diverse options were agender, gender nonconforming, gender fluid, or nonbinary.

**TABLE 10:** Descriptions of How Raw Responses on Parent‐Report Measures Were Recoded Into 5‐Category Scheme

Measure	Response Options	Response Coded as:					Responses Requiring Human Coding (*N*)	Inter‐rater Reliability (*κ*)
		Boy	Boy‐Expansive	Gender Diverse	Girl‐Expansive	Girl		
*Measure 7*	Select One of: Boy, Girl, or Other	Selecting “Boy” or Self‐reporting a Term Recoded as Boy	Self‐Reporting a Term Recoded as Boy	Selecting “Other” and Self‐Reporting a Term Recoded as Boy	Self‐Reporting a Term Recoded as Girl‐Expansive	Selecting “Girl” or Self‐Reporting a Term Recoded as Girl	49	0.78
*Measure 8*	Select one of: boy, girl, nonbinary, gender fluid, agender, I prefer a different term	Selecting “boy”, or self‐reporting a term recoded as boy	Self‐reporting a term recoded as boy‐expansive	Selecting “nonbinary”, gender fluid”, or “agender”, or self‐reporting a term recoded as gender diverse	Self‐reporting a term recoded as girl‐expansive	Selecting “girl”, or self‐reporting a term recoded as girl	35	0.96
*Measure 9*	Select one of: transgender, gender nonconforming, nonbinary, cisgender, gender fluid, agender, I prefer a different term	Self‐reporting a term recoded as boy	Self‐reporting a term recoded as boy‐expansive	Selecting “gender fluid”, “nonbinary”, “agender”, or “gender nonconforming” or self‐reporting a term recoded as gender diverse	Self‐reporting a term recoded as girl‐expansive	Self‐reporting a term recoded as girl	35	0.83
*Measure 10* a	Select all that apply among: both genders, neither gender, cisgender boy, cisgender girl, female‐to‐male transgender, male‐to‐female transgender, bisexual, gay male, lesbian, gender‐nonconforming natal male, gender‐nonconforming natal female, other	Selecting “female‐to‐male transgender” or “cisgender boy”, or self‐reporting a term recoded as boy	Self‐reporting a term recoded as boy‐expansive, or selecting a combination of terms deemed by human coders as indicative of boy‐expansive identity	Self‐reporting a term recoded as gender diverse, or selecting a combination of terms deemed by human coders as indicative of gender‐diverse identity	Self‐reporting a term recoded as girl‐expansive, or selecting a combination of terms deemed by human coders as indicative of girl‐expansive identity	Selecting “male‐to‐female transgender” or “cisgender girl”, or self‐reporting a term recoded as girl	24	0.76
*Measure 11*	Open‐ended description	All responses were reviewed by human coders					336	0.96

^a^ For Measure 10, not all responses required review from human coders; responses that were relatively simple (e.g., consisted only of “cisgender boy” or “male‐to‐female transgender”) were coded automatically, and only more complex, ambiguous, or open‐ended responses were reviewed manually.

##### III.3.2.2 Qualitative Coding in Step 2

In Step 2, we took all of the (five‐category) codes for each individual measure a respondent had provided at a visit and combined them into a single gender‐at‐visit code. Most often, all of a respondent's answers within a single visit received the same five‐category code; in these cases, this five‐category code was deemed the respondent's gender‐at‐visit code. However, respondents sometimes provided answers that received discrepant five‐category codes within the same visit (11.8% and 3.6% of youth‐ and parent‐report visits, respectively, in which respondents answered two or more gender identity measures). In these cases, their whole set of responses from the visit was examined by human coders. We also gave coders access to youths' *recruitment gender* (i.e., whether they were recruited into the study as a boy or a girl; see Chapter II for details), assigned sex, and their score on a continuous measure of gender identity (see *Continuum* from “boy” to “girl”, Table 6). Coders were given this additional information to help disambiguate complex cases in which youths gave seemingly conflicting or uninformative answers (e.g., if a respondent described their gender identity with a description such as “Very typical of people of my gender”, additional information was helpful in determining what they meant). Overall, inter‐rater reliability was good for Step 2 qualitative coding (*κ* = 0.76 and 0.96 for youth‐report and parent‐report, respectively).

#### III.3.3 Visits Without Interpretable Responses

Additionally, there were cases in which a participant (usually a youth, but occasionally a parent) participated in a study visit but did not provide enough information for the research team to use the participant's responses to arrive at a gender‐at‐visit code. This was most frequently the case with youth self‐report visits prior to 2017, when the only youth self‐report measure administered to children was an alternative, superseded version of Measure 1 which offered 6 gender options to children to choose from all at once (“Are you a boy, a girl, both, neither, it changes, or you don't know?”). Anecdotal experience among members of the research team who administered this measure to children indicated that offering so many response options to children as young as three was confusing and overwhelming, and led to responding that appeared random in all three *recruitment groups*; thus, due to construct validity and comprehensibility concerns, the research team decided it should not be used when determining the youth's gender‐at‐visit code (see Supporting Information for statistical analysis supporting the observation that this measure was too confusing for children). As a result, many youths in the study did not have a codable self‐report of their own gender on their first visit in the study, particularly youths who began participating prior to 2017. Missing data also occurred, for example, if a participant skipped all gender report questions, gave responses that were all deemed uncodable, or gave conflicting responses that coders could not reconcile in Step 2 qualitative coding (e.g., saying they were “only a boy” on one measure and “only a girl” on another measure).

#### III.3.4 Condensing Five‐Category Codes into Three‐Category Codes

A few measures did not include answer choices that could be coded as boy‐expansive or girl‐expansive (e.g., Measure 1 in Table 6 earlier this chapter). If a youth or parent only received this type of measure on a particular visit, then they could only have been assigned a visit‐level code of boy, girl, or gender diverse for that visit (and not one of the “expansive” categories). To make this type of visit comparable to a visit in which the participant could express an expansive identity (e.g., by writing in “nonbinary boy” as an open‐ended answer), we also converted the five‐category visit‐level codes into three‐category codes. There were two options for this recoding. In the main text, we report results from analyses in which the girl‐expansive and boy‐expansive categories are recoded as girl and boy, respectively; in the Supporting Information, we report analytic results obtained if expansive categories are recoded as gender diverse. In general, results are quite similar across these two approaches as only 3.4% of gender‐at‐visit summary codes fell into the expansive categories.

### III.4 Evidence of Validity of Five‐Category Coding Process

Before addressing our central questions in Chapters IV and V concerning stability of gender over time using the visit‐level codes, we first report here on the validity of our five‐category coding system. Here, we discuss two pieces of information suggesting that these codes are face valid: (1) coherence between a respondent's answers when they received more than one open‐ended or discrete measure in a single visit; and (2) convergent validity of 5‐category codes with concurrent scores on a continuous measure of gender identity (*Continuum* measure, described briefly in Table 6 and explained in more detail in the Supporting Information for this chapter).

**Visit‐Level Coherence Among Categorical Measures: Youth‐Report.** When youths gave reports on several measures in a single visit, their responses on those measures usually yielded the same code in the 5‐category coding scheme. In total, there were 1423 visits in which youths gave at least two codable responses to separate gender‐relevant questions (meaning that, in theory, it was possible for the youth to have a within‐visit discrepancy); in 1256 of these cases (88.2%), the youths' multiple answers at that visit were assigned the same gender code, suggesting a high level of convergence between measures. Visits with conflicting codes (*N* = 167) were qualitatively coded as described above (Step 2 coding); interrater reliability was good and disagreements were resolved through discussion (*κ* = 0.76).

**Visit‐Level Coherence Among Measures: Parent Report.** When parents gave reports on several measures in a single visit, their responses on those measures usually yielded the same code in the 5‐category coding scheme. In total, there were 1042 visits in which parents gave at least two codable responses to separate gender‐relevant questions (meaning that, in theory, it was possible to have a within‐visit discrepancy); in 1004 of these cases (96.4%), the parents' multiple answers at that visit were assigned the same gender code, suggesting a high level of between‐measure convergence. For those visits in which parents gave responses that were assigned conflicting codes (*N* = 38), human coders examined all the parent's responses to arrive at a single determination for their report of their child's gender at that visit; interrater reliability was high and resolved through discussion (*κ* = 0.96). For information regarding convergence between parent reports when two parents simultaneously reported about the same youth, see the Supporting Information for this chapter.

**Coherence Between Youth‐Report Identity and** ***Continuum*** **Scores.** To further assess the validity of our 5‐category codes for youth‐report data, we examined the relation between those codes and youths' contemporaneous scores on a continuous identity measure (henceforth referred to as *Continuum*). On *Continuum*, youths were asked to indicate their identity on a scale from 0 (feeling totally like a boy) to 100 (feeling totally like a girl), with 50 representing “a mix of both”; see Supporting Information for this chapter for more details on *Continuum*'s administration to participants, scoring, and results. For our purposes in this chapter, we are interested in whether *Continuum* scores align with codes assigned to youths' responses on individual measures (Step 1 coding) and gender‐at‐visit codes (Step 2 coding).

**Figure 4:**
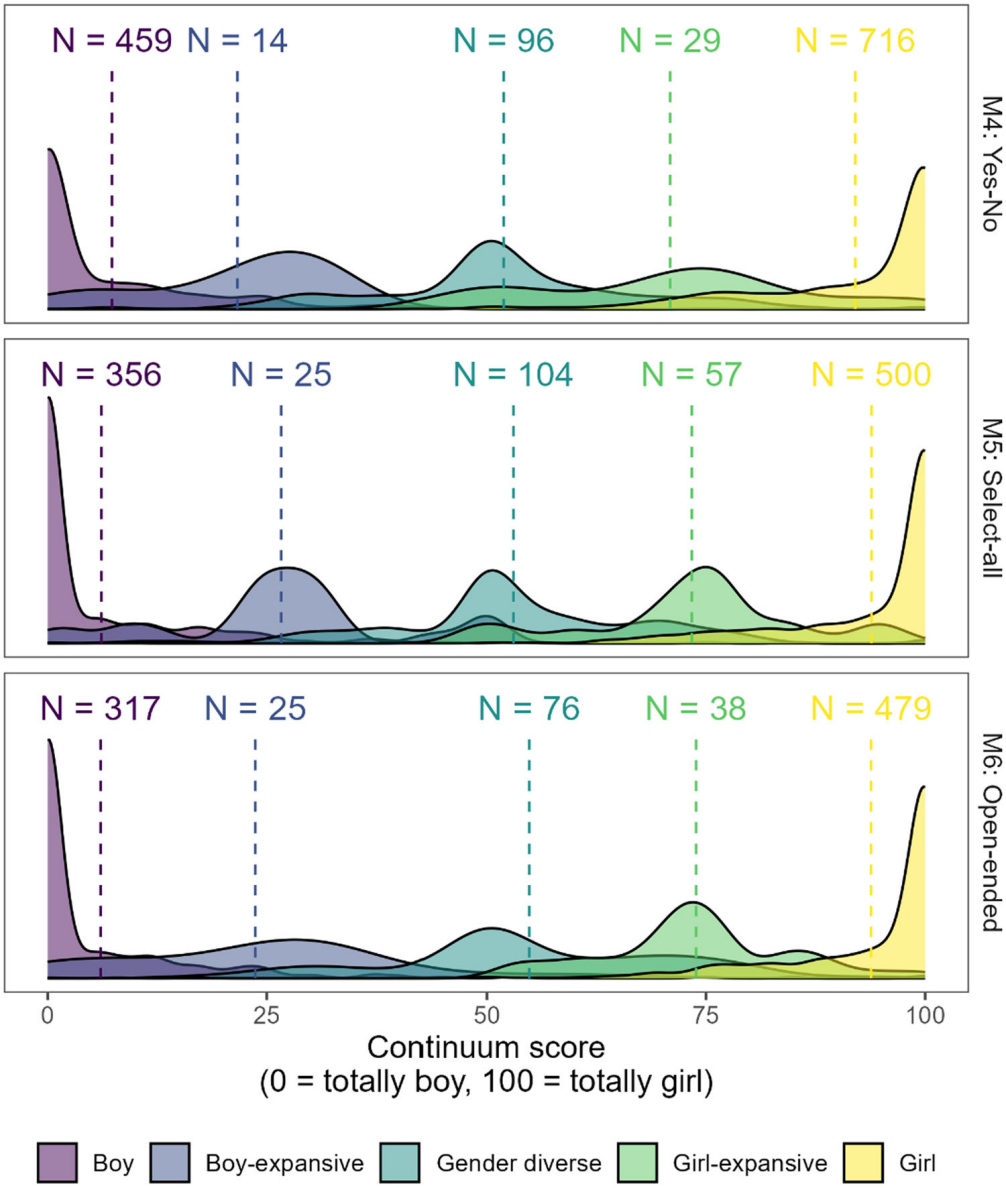
Density curves of youth‐report *Continuum* scores in all three recruitment groups, broken down by Step 1 coding on measures 4, 5, and 6. Dashed lines represent means. *Note*: All three *recruitment groups* are included in every panel of this figure due to small *N*'s in some categories.

**Figure 5:**
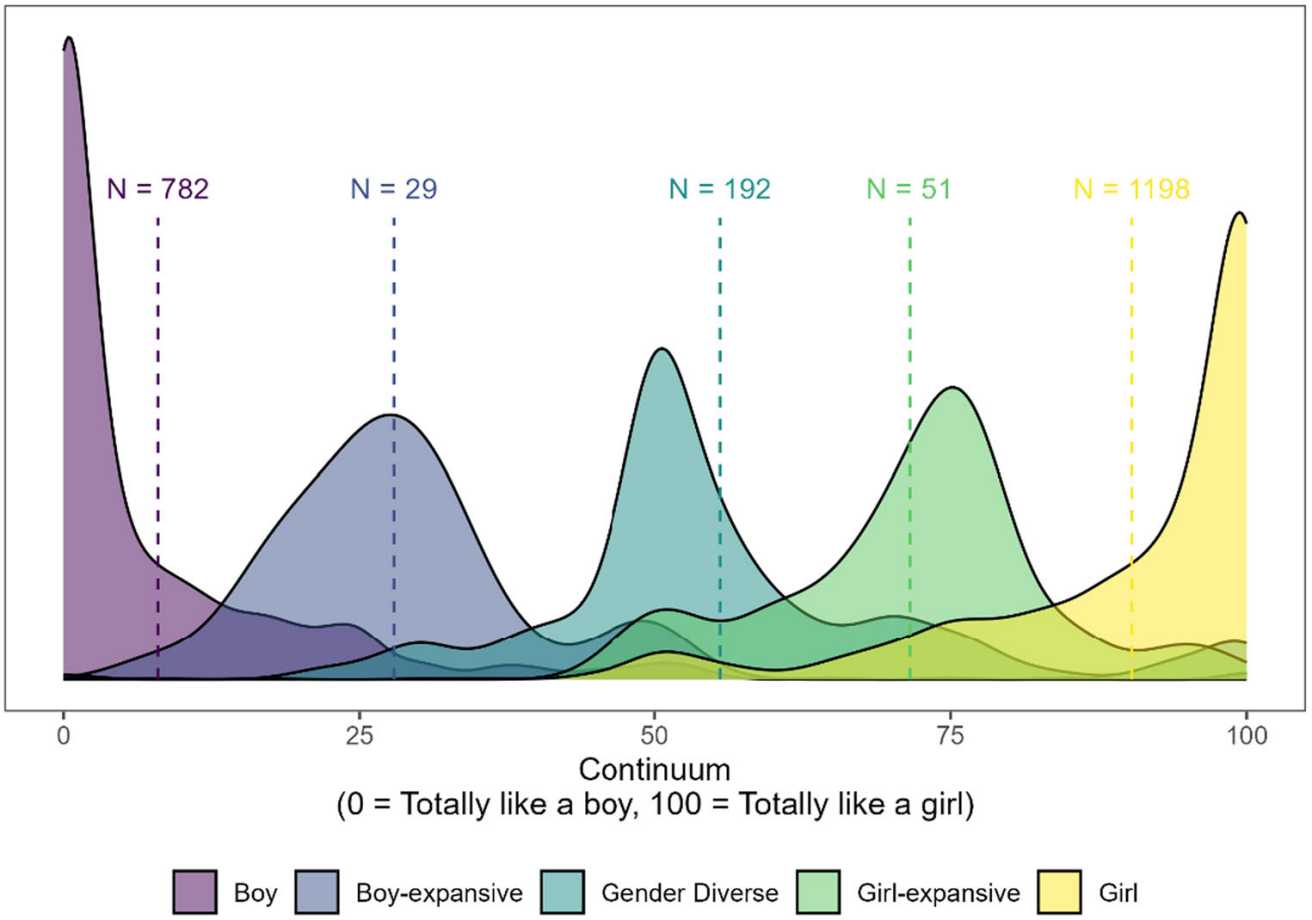
Density curves of youth‐report *Continuum* scores in all three recruitment groups, broken down by Step 2 gender‐at‐visit coding. Dashed lines represent means. *Note*: In 7.4% of visits represented in this figure, human coders had access to *Continuum* scores when deciding visit‐level gender codes; all three *recruitment groups* are included together in this figure due to small *N*'s in some categories.

First, we assessed whether codes assigned to individual measures in Step 1 coding (prior to being synthesized in Step 2 coding into a visit‐level gender‐at‐visit code for a youth) showed the expected pattern of associated *Continuum* scores. Density curves in Figure 4 depict the clear relation between a youth's *Continuum* score and the code they received in the 5‐category scheme for Measures 4, 5, and 6 (selected for visualization because they allowed for a respondent to be coded into any of the five categories, and had sufficient response *N*'s to be analyzed in this way). The correspondence between *Continuum* scores and Step 2 overall gender‐at‐visit codes shows a similar pattern (Figure 5), though we note that in 7.4% of visits depicted in Figure 5, human coders decided on the gender‐at‐visit code while having access to the youth's *Continuum* score—a choice made to help disambiguate rare cases in which a participant's responses on discrete or open‐ended measures within a visit were truly incommensurate or uninterpretable. In sum, these visualizations demonstrate a robust coherence between codes on the 5‐category scheme and scores on *Continuum*.

### III.5 Overall Agreement Between Youth‐ and Parent‐Report Gender‐at‐Visit

**Figure 6:**
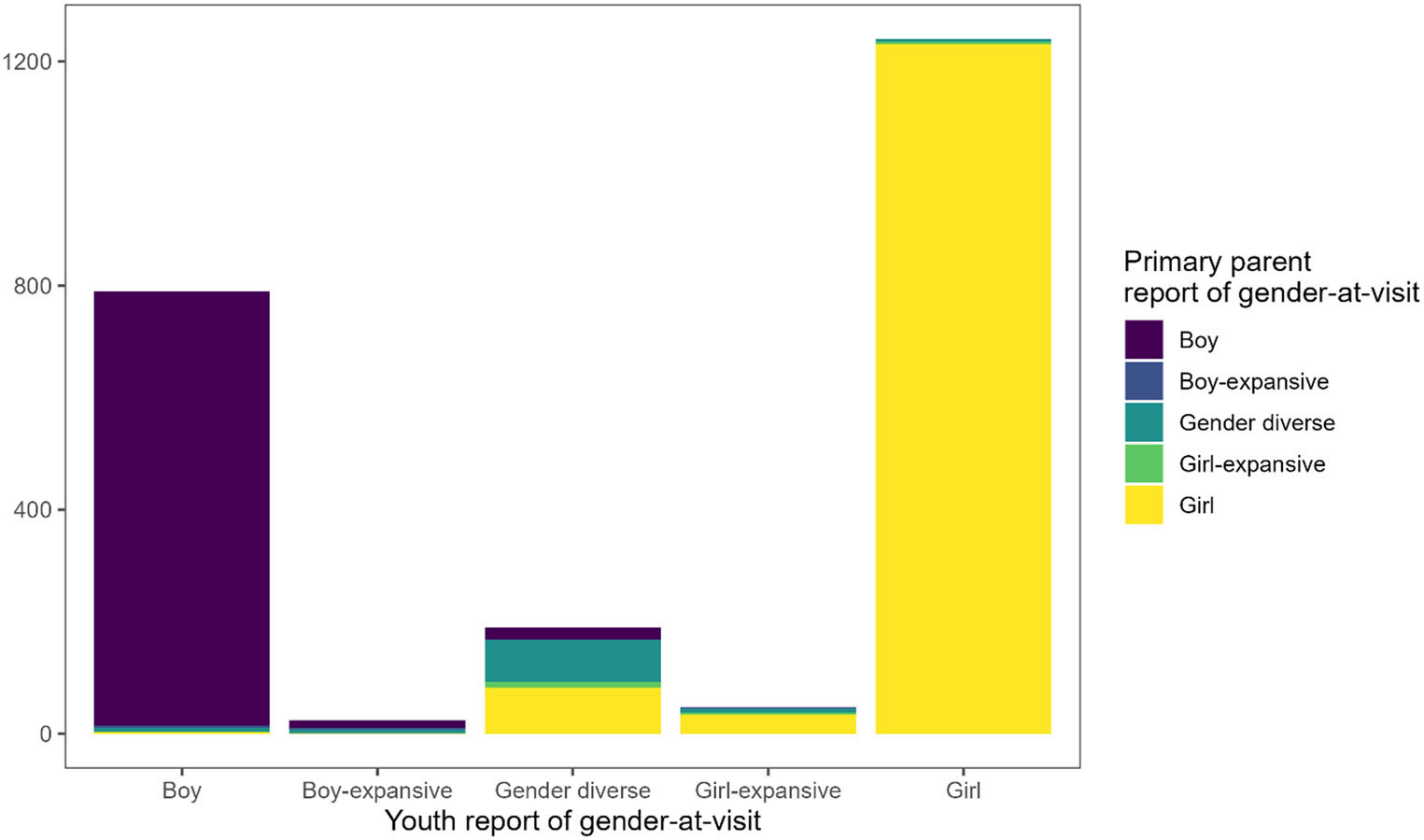
Between youth‐ and parent‐reported gender‐at‐visit codes. *Note*: Youths with multiple visits at which both parent and youth provided relevant answers are included multiple times in this figure.

Among all visits in which both youths and parents gave categorical reports of the youth's gender (*N* = 2292 visits), the gender‐at‐visit code assigned to the youth's report was the same as that assigned to the parent's report 91.1% of the time. Figure 6 illustrates the overall agreement between youths' and parents' gender‐at‐visit codes. Disagreement between youths and parents was exceedingly rare when youths reported binary boy or girl identities (the vast majority of visits), but relatively more common when youths reported a boy‐expansive, girl‐expansive, or gender‐diverse identity. In general, parents tended to provide binary labels more often than youths, and youths tended to provide expansive or gender‐diverse labels more often than parents. More information about youth‐parent coherence with respect to youths' most recently reported identities appears in Chapter IV.

### III.6 Summary

Information about youths' gender identities in the TYP takes the form of responses to highly heterogeneous measures from youths and parents over the course of more than a decade. This chapter described how we synthesized these data to create a code for each youths' gender at each visit (according to each respondent—youth or parent—who participated). Youths could be coded as boys, girls, gender diverse (e.g., nonbinary, agender, genderqueer, etc.), boy‐expansive (a combination of boy and gender‐diverse identification), or girl‐expansive (a combination of girl and gender‐diverse identification). These gender‐at‐visit codes showed good correspondence with youths' contemporaneous reports of identity on a continuous scale from 0 to 100. When both a youth and their parent participated at the same visit, they were overwhelmingly likely to report the same gender identity for the youth (>90% of the time).

## IV Stability and Change in Gender Identity Over Time

### IV.1 Chapter Highlights

- We discuss how many youths in the Trans Youth Project have reported gender stability and change and test whether this differs among youths in the three *recruitment groups* (*Recruited as Transgender*, *Recruited as Cisgender*, and *Recruited as Siblings*); for this topic, see **Results for RQ1: How Much Stability and Change Do Youths and Parents Report?**.
- We then focus on youths' current identities (i.e., the ones they reported at their most recent visit in the TYP) and whether they are or are not the same as youths' *recruitment genders* (i.e., their gender at the beginning of the study). We examine if rates of concordance between *recruitment gender* and current gender differ among the three *recruitment groups*. For this topic, see **Results for RQ2: What are Youths' Current Identities, and How Do They Align with Recruitment Gender?**.
- On both of the above questions, we compare whether youths and parents systematically differed; see subsections ***Comparing Youth‐ and Parent‐Reported Stability and Change*** and ***Comparing Youths' Current Identities by Reporter (Youth vs. Parent)***. We also explore if the first gender change is more likely to occur at particular points in development (see subsection ***When in Development Does Change Occur?***).
- Analytic choices are explained in‐depth in ***Technical Details***.

### IV.2 Introduction

How much does a person's sense of their gender identity change (or not) during childhood or adolescence? Psychologists, clinicians, and researchers have explored some version of this question for decades, but almost always have done so in a specific subset of youths: those whose persistent gender nonconformity in childhood concerned their parents (or other adults) enough to seek clinical advice (e.g., Green, 1987; Zucker & Bradley, 1995). In this chapter, we ask this question about two other groups of youths who, for the most part, have not been studied in past research on stability and change in gender identity over time: (1) socially transitioned transgender youths, who changed their names, pronouns, clothing, and/or hairstyle to live as the other binary gender than their assigned sex and (2) youths who were cisgender in childhood and (at least to the knowledge of their parents) did not show significant indications of gender‐diverse identities as children. Two societal shifts within the past 15–20 years have allowed for an empirical investigation of these groups where doing so before may not have been possible.

The first shift is that a growing cohort of gender‐nonconforming children have been socially transitioning to live in line with their gender identities early in life. Social transitions in childhood have occurred in the United States for at least a century (Gill‐Peterson, 2018), but appear to have been infrequent until the 2000s. Only now are there large enough numbers of adolescents and young adults who socially transitioned in childhood to study these questions quantitatively. As this group has become increasingly visible, researchers, clinicians, and parents have begun asking about what their identities will look like in the long term. Do youths who live as transgender early in childhood later live as cisgender—or undergo what is sometimes called “retransition” or “detransition” (Green, 2017; Olson et al., 2022; Steensma et al., 2011)?

Second, recent polls and research studies suggest that more and more young people are identifying as transgender and nonbinary by adolescence—some of whom may not have shown persistent gender nonconformity or gender dysphoria as children. Prior to the 2010s, showing such a trajectory was considered to be rare—so much so that it was not on the radar of most gender development researchers (in contrast to the idea that sexual orientation could fluctuate across childhood and adolescence, which has been recognized and investigated since at least the 1980s). Recent population‐level data illustrate the growth of this increase in transgender and nonbinary identification: the 2023 Youth Risk Behavior Survey, a representative sample of U.S. high school students (roughly ages 14–18), found that 3.3% identified as transgender and another 2.2% were questioning whether they might be (Suarez et al., 2024). Other research has suggested that young people are particularly likely to identify as transgender or nonbinary relative to people in older generations: a 2022 poll from Pew Research Center found that while 5.1% of 18–29‐year‐olds identified as transgender or nonbinary, only 1.6% of 30–49‐year‐olds and 0.3% of 50+ year‐olds did (Brown, 2022). These data suggest that what may have been thought of as an exceedingly uncommon trajectory in past work may not be so rare anymore—thereby giving researchers a better opportunity to document these trajectories in the present day. Below, we review existing research on gender stability and change through childhood and adolescence; we then highlight how the samples we report on in this monograph extend this literature to a wider swath of youths (see “Work in the Current Chapter”).

### IV.3 Identity Stability and Change in Gender‐Nonconforming Youth

Clinical psychologists and psychiatrists have long been interested in whether gender‐diverse children will show stability or change in their gender identity (Bakwin, 1968; Davenport, 1986; Drummond et al., 2008; Green, 1974, 1987; Kosky, 1987; Lebovitz, 1972; Money & Russo, 1979; Zucker & Bradley, 1995; Zuger, 1966). Classic studies in this area reported on the longitudinal outcomes of children whose parents were concerned about their gender (e.g., because their male child was perceived to be too feminine and/or consistently wished to be a girl) and brought them to gender clinics for assessment and/or treatment. Most of the youths in these studies later outgrew these concerns and “desisted” from their earlier gender dysphoria. For example, studies from the two largest clinics publishing data on this topic found that between 12% and 39% of youths who could be reached for follow‐up persisted in showing gender dysphoria as adolescents or adults, while the rest came to identify as what we might call cisgender today (Zucker & Bradley, 1995; Green, 1987; Wallien & Cohen‐Kettenis, 2008; Steensma et al., 2013a; Drummond et al., 2008; Singh et al., 2021). Interpreting these data is complicated by uncertainty about whether the youths in those studies all had “cross‐sex” identification in childhood or not [i.e., did the assigned males in these studies identify as girls, or rather as boys who had feminine interests? (Olson, 2016; Temple Newhook et al., 2018)]. It thus remains unclear whether this same pattern—in which only a minority of gender‐nonconforming children persist in gender dysphoria and/or identify as transgender as adolescents or adults—will apply to a sample of youths who unambiguously identified as transgender (i.e., a member of a gender category that does not align with their assigned sex) in childhood and who actually lived in line with that gender identity early in life (i.e., socially transitioned).

There is some reason to suspect that socially transitioned youth might identify as transgender as adolescents or young adults at higher rates than gender‐ nonconforming children in past clinical samples. Rae et al. (2019) found that in a group of gender‐nonconforming youths, those who went on to socially transition showed stronger “cross‐gender” identities and gender‐typed preferences (e.g., for toys, peers, and clothing) before they transitioned than those gender‐ nonconforming youths who did not ultimately socially transition. Furthermore, the aforementioned clinic‐based studies have generally found that those youths who showed stronger “cross‐gender” preferences and identities (i.e., reported stronger identification in childhood with the other binary gender from their assigned sex) in childhood were most likely to identify as transgender (or continue to show gender dysphoria) in adolescence and adulthood (Wallien & Cohen‐Kettenis, 2008; Steensma et al., 2013a; Drummond et al., 2008; Singh et al., 2021; Zucker & Bradley, 1995; see Chapter V for more on these findings). These findings suggest that those gender‐nonconforming youths who socially transitioned in childhood might be especially likely to later live as transgender adolescents or adults.

While some recent work has reported on longitudinal outcomes in transgender teenagers (Katz‐Wise et al., 2024), the only published work that speaks to identity stability or change in youth who socially transitioned as children is a prior Trans Youth Project publication with the *Recruited as Transgender* cohort we report on in this monograph. That study showed that among the 317 youths in the *Recruited as Transgender* group, 94% were still identifying as binary transgender, 3.5% were identifying as nonbinary, and 2.5% were identifying as cisgender an average of 5 years after their initial social transition, when they were an average of 12 years of age (Olson et al., 2022). One limitation of that study was that the categorization of youths' identities was made using parents' reports about the pronouns youths were using in everyday life, rather than youths' or parents' reports of their actual identification. Furthermore, it has been suggested that the period of time when gender‐diverse youths are the most likely to retransition to live as cisgender is between ages 10 and 13 (Steensma et al., 2011), indicating that outcomes after that time, like those we report here, would be more appropriate to compare to past clinic‐based reports.

### IV.4 Work in the Current Chapter

This chapter expands on the aforementioned previous Trans Youth Project publication (Olson et al., 2022) in several ways. First, we examine youths' and parents' reports of the youth's identity over time using direct reports of identity, rather than only using parents' reports of pronouns as a proxy for gender identity. Second, while the previous report utilized data through 2020, this chapter is more up‐to‐date and provides information over a longer timespan as it includes data through February 2024.

Finally, and perhaps most uniquely, the current report also addresses these questions in a sample of youths who were all cisgender upon recruitment and did not show notable gender nonconformity at the start of the study (*Recruited as Cisgender* and *Recruited as Siblings* groups). Of course, as with all groups of youths, the youths in these groups varied in terms of how gender conforming they were at the start of the study (e.g., some of the girls were stereotypically “girly” while others were tomboys), but the key point is that they were not specifically recruited because of notable gender nonconformity, in contrast to the *Recruited as Transgender* group and longitudinal samples in past research. Youths in the *Recruited as Cisgender* and *Recruited as Siblings* groups are particularly important to study given broader demographic and societal shifts that suggest that more youth in this generation are identifying as transgender or nonbinary. We suspect that these youths in our study have significantly more latitude and opportunity to show fluidity in their gender over time than previous generations given the cohort effect that seems to be occurring at the moment (Suarez et al., 2024; Twenge, 2024; Brown, 2022) and because these youth are in families that are generally more tolerant of gender diversity (as indicated by political orientation and willingness to participate in the current study on gender diversity). Any shifts or changes in their identities during the course of the study can also help to contextualize findings within the *Recruited as Transgender* group. For example, if we observe notable numbers of *Recruited as Transgender* youths identifying as nonbinary, we can ask if this trajectory reflects something unique about youths who socially transitioned earlier in childhood, or whether this trajectory is also common in youths who did not socially transition in childhood—and thus may reflect a broader cultural or societal change.

#### IV.4.1 Research Questions

In this chapter, we focus on two primary research questions, each with their own set of follow‐up questions.

##### IV.4.1.1 Research Question 1

First, how stable or not are youths' gender identities? This question considers change that may have happened at any point in the study, in any direction; thus, for example, a youth whose *recruitment gender* was boy, and who then reported a nonbinary identity before transitioning back to being a boy at their latest report, would count as having shown change in analyses dealing with this first question. As a follow‐up to RQ1, we consider whether stability and change in gender identity varies by initial *recruitment group* (*Recruited as Transgender*, *Recruited as Cisgender*, or *Recruited as Siblings*) or reporter (youth or parent). We additionally probe whether, when we do see change, it tends to occur at particular times in development.

##### IV.4.1.2 Research Question 2

Second, what are youths' current gender identities? This question, while similar to the first question above, is separate: whereas the first question is concerned with whether youths showed any gender change at all across all their visits in the TYP, this second question only considers what youths and parents said at their most recent report of gender. As a follow‐up to RQ2, we examine whether *recruitment groups* vary in their reports of current gender identity relative to *recruitment gender*—in other words, we ask if youths in some *recruitment groups* are more or less likely to currently have the gender with which they started the study. We also discuss whether youths or parents differ in how often reports of current gender do or do not accord with *recruitment gender*.

#### IV.4.2 Technical Details

##### IV.4.2.1 Defining Change Relative to *Recruitment Gender*

In this chapter's longitudinal analyses, we could determine a child's “starting” gender in one of two ways: by *recruitment gender*, defined as the gender that the child was using in everyday life at the time of their first participation in the study (e.g., a child was a girl if she used she/her pronouns and was treated by others as a girl; see Chapter II for details), or by *earliest self‐described gender*, defined as their earliest codable self‐report of gender (see Chapter III for details on how responses were deemed codable or not). For 90% of participants, *recruitment gender* and *earliest self‐described gender* were the same. However, there are practical differences between the two approaches. A youth often did not provide a codable report of gender at their first visit, meaning that in order to use their *earliest self‐described gender* as their “starting” point, we either needed to ignore data before the *earliest self‐described gender* was provided (reducing total usable data), or we had to assume that a youth's report at (for example) the second visit (if the first was uncodable) would have applied at the first visit, presuming more stability than may be warranted.

For this reason, in the main text, we define stability and change relative to *recruitment gender* throughout. However, for all formal analyses in this chapter that examine youths' self‐reported stability and change, we include a parallel analysis using *earliest self‐described gender* as the youth's “starting point” in the Supporting Information for this chapter. We do not include parallel versions of formal analyses examining parent‐report data in which we define gender change relative to *earliest self‐described gender*; the reason is that parents' earliest descriptions of their children's gender were virtually always the same as the youth's *recruitment gender* (in fact, researchers decided on the youth's *recruitment gender* based in part off of parents' testimony), so the two approaches are essentially redundant when considering parent‐report data. The advantage of the *earliest self‐described* approach is that it is a more child‐centered analysis, given that *recruitment gender* was influenced by parental input (parents told researchers what pronouns the youth was using), and *earliest self‐described* gender was not. Almost all results in this chapter show the same patterns regardless of the way we code gender at recruitment.

##### IV.4.2.2 Operationalization of Gender Change and Statistical Modeling Strategy

In this chapter's analyses, we are interested in whether a youth has shown stability or change in their gender identity during study participation. In Research Question 1, we are interested in whether a youth has shown any change at all, whereas in Research Question 2, we are only concerned with the youth's current gender and whether it has changed from their *recruitment gender* (without considering what may have happened in between). In both operationalizations of change, we use multi‐level logistic regression to predict change by other variables (e.g., *recruitment group*) while accounting for the nested structure of the data (i.e., having multiple participants per family). In cases when categorical predictors have more than two levels, we follow up on these with Wald χ^2^ tests to assess the overall relationship between individual predictors and the dependent measure (Beitler & Landis, 1985; Luke, 2017). In answering these questions, we also consider that participants who have been in the study for longer periods of time have had more of an opportunity to show change. For that reason, we include *time elapsed* since the beginning of the youth's participation in the study as an additional predictor and report when *time elapsed* is and is not associated with change. Throughout this monograph, we do not run statistical tests if the underlying data contain 10 or fewer participants in any given cell; this issue did not affect any of our intended tests in Chapter IV, but it does come up in Chapters V and VI.

##### IV.4.2.3 Stability and Change on Individual Measures

This chapter focuses on stability and change in categorical identity between visits. As such, we are operating on the overall gender‐at‐visit categorizations described in Chapter III, rather than being interested in whether youths gave the same response on any particular measure from visit to visit. However, summaries of stability and change on particular measures are reported in the Supporting Information for this chapter.

##### IV.4.2.4 “Expansive” Categories

In Chapter III, we described how youths who expressed an “expansive” identity (i.e., a mix of “boy” or “girl” and nonbinary identification) were recoded in two ways for compatibility across visits. In this chapter, we report results with the “boy‐expansive” and “girl‐expansive” categories coded as “boy” and “girl”, respectively; in the Supporting Information, we show parallel results if these categories are coded as gender diverse instead. Results between the two approaches are almost always the same; see Supporting Information for minor discrepancies.

##### IV.4.2.5 Referring to “Current Gender”

One final note about terminology: we sometimes refer to a youth's “current” gender identity. What we mean is the gender identity the youth reported at their most recent visit before February 2024. We use this term for ease of communication (rather than specifying “gender identity at the latest wave included in the current report”), with an understanding that the larger longitudinal study is still ongoing at the time of our writing this manuscript.

### IV.5 Results for RQ1: How Much Stability and Change Do Youths and Parents Report?

#### IV.5.1 Stability and Change According to Youth Report

##### IV.5.1.1 Overall Stability and Change

Table 11 shows what percentage of youths in each *recruitment group* (a) have given a codable report of their gender at a follow‐up visit; and (b) of those who have, what percentage have shown change. Across all groups, the most common pattern was stability: of those with a codable report at a follow‐up visit, 83.1% reported stability and 16.9% reported change. Detailed visualizations of youth‐reported stability and change for each participant are shown in Figures S3–S5 in the Supporting Information for this chapter.

**TABLE 11:** Proportion of Youths Who Have Shown Gender Change According to Youth Report

Recruitment Group	Total *N*	*N* (% of Total)		*N* (% of Youths With Codable Follow‐up)			Mean Age at First Visit (Years)	Mean Age At Most Recent Codable Report (Years)
		Missing Codable Follow‐up	Has Codable Follow‐up	Stable	Has Shown One Change	Has Shown More Than One Change		
Recruited as Transgender	317	34 (10.7%)	283 (89.3%)	231 (81.6%)	29 (10.2%)	23 (8.1%)	8.0	15.3
Recruited as Cisgender	377	92 (24.4%)	285 (75.6%)	245 (86.0%)	21 (7.4%)	19 (6.7%)	8.4	15.2
Recruited as Siblings	218	41 (18.8%)	177 (81.2%)	143 (80.8%)	24 (13.6%)	10 (5.6%)	7.8	14.4

##### IV.5.1.2 Stability and Change by Group

To determine whether there were differences in levels of stability and change among the three *recruitment groups*, we fit a mixed‐effects logistic regression model predicting whether a youth has shown stability from three predictors: (1) a fixed‐effect categorical predictor of *recruitment group*, with three levels (*Recruited as Transgender*, *Recruited as Cisgender*, and *Recruited as Siblings*); (2) a fixed‐effect continuous predictor of *time elapsed* between the research team's first occasion collecting data about the youth and the youth's most recent codable report of gender (in years, mean‐centered); and (3) a random intercept for each family to account for nonindependence introduced by sibling pairs in the sample. A Wald χ^2^ test on this model found that *recruitment group* was not a significant predictor of gender stability, Wald χ^2^(2) = 2.37, *p* = .305, indicating that there was no significant difference in stability and change between the three *recruitment groups*. However, *time elapsed* was a significant predictor of stability such that participants who had been in the study for a longer time showed slightly less stability in gender identity than those who had been in the study for a shorter length of time, Wald χ^2^(1) = 5.09, *p* = .024. This time effect would generally be expected since the longer one spends in the study, the more opportunity they have to show gender change.

#### IV.5.2 Stability and Change According to Parent Report

##### IV.5.2.1 Overall Stability and Change

Similar to what we found in youth reporters, the most common pattern in parent‐reporters was stability. Among parents across all groups with a codable follow‐up report of their child's gender, 10.7% reported change and 89.3% reported stability in their child's gender identity. Table 12 shows the percentage of youths in each *recruitment group*, according to parent‐report, who have and have not shown gender change. Detailed visualizations of parent‐reported stability and change for each participant are shown in Figures S6–S8 in the Supporting Information for this chapter.

**TABLE 12:** Proportion of Youths Who Have Shown Gender Change According to Parent Report

Recruitment Group	Total *N*	*N* (% of Total)		*N* (% of Youths With Codable Follow‐up)			Mean Age at First Visit (Years)	Mean Age at Most Recent Codable Report (Years)
		Missing Codable Follow‐up	Has Codable Follow‐up	Stable	Has Shown One Change	Has Shown More Than One Change		
Recruited as Transgender	317	12 (3.8%)	305 (96.2%)	270 (88.5%)	23 (7.5%)	12 (3.9%)	8.0	15.1
Recruited as cisgender	377	30 (8.0%)	347 (92.0%)	318 (91.6%)	18 (5.2%)	11 (3.2%)	8.3	14.1
Recruited as Siblings	218	10 (4.6%)	208 (95.4%)	180 (86.5%)	20 (9.6%)	8 (3.8%)	7.8	14.1

##### IV.5.2.2 Stability and Change by Group

To determine if there were group differences in levels of stability and change as assessed by parents, we fit a logistic mixed‐effects model predicting whether a youth (according to their primary parent) has shown stability relative to their *recruitment gender*, with the same three predictors as in the analysis of youth‐reported change: (1) a categorical fixed‐effect of *recruitment group* with three levels; (2) a continuous fixed‐effect of *time elapsed* between the beginning the youth's participation in the study and the primary parent's most recent codable report of the youth's gender (mean‐centered, in years); and (3) a random intercept for each family to account for nonindependence introduce by siblings pairs. A Wald χ^2^ test on this model found that *recruitment group* was not a significant predictor of stability and change in gender identity, Wald χ^2^(2) = 5.50, *p* = .064. However, *time elapsed* was significantly related to stability and change in gender identity such that more time spent in the study was associated with slightly higher levels of gender change, Wald χ^2^(1) = 3.92, *p* = .048.

#### IV.5.3 Comparing Youth‐ and Parent‐Reported Stability and Change

Tables 11 and 12 suggested that parents were slightly less likely to say their children's gender had changed than the youths themselves were. To test the comparison of youths and parents directly, we selected the subset of youths for whom we have codable follow‐up reports from both the youth and their parent (*N* = 744) and ran a McNemar's test on the contingency table of youths' and parents' reports of whether the youth had remained stable in their gender identity (coded as 1) or had reported change at some point in the study (coded as 0). Parents were less likely to say that their children's genders had changed than the youths themselves were (McNemar's χ^2^(1) = 25.35, *p* < .001). In the discussion, we address why we might be seeing higher rates of change in youth‐reported data relative to reports from parents.

#### IV.5.4 When in Development Does Change Occur?

To investigate whether changes in gender identity—and specifically a person's first change in identity from their *recruitment gender*—tend to occur at particular ages, we calculated the percentage of follow‐up visits at each age in which participants reported change from their *recruitment gender* for the first time. These percentages are shown in Tables 13 (youth report) and 14 (parent‐report). Note that once a given participant had a first change, their later data were removed from these calculations because they could not, by definition, show a first change after that point. These tables indicate that changes occurred throughout development in all three participant groups and that no particular developmental stage was associated with observing dramatically higher levels of first change.

**TABLE 13:** Percent of Visits at Different Ages in Which Youths Self‐Reported First Gender Change

Age (Years)	% of Visits in Age Range Reporting First Gender Change (*N*)			
	Recruited as Transgender	Recruited as Cisgender	Recruited as Siblings	Total
6–8	11.0% (8)	5.3% (3)	8.0% (4)	8.3% (15)
9–11	10.9% (21)	3.6% (6)	12.7% (8)	8.3% (35)
12–14	5.6% (13)	7.1% (18)	9.1% (11)	6.9% (42)
15–17	4.2% (8)	7.1% (12)	7.8% (6)	6.0% (26)
18+	1.9% (1)	1.9% (1)	8.0% (2)	3.1% (4)

*Note*: Figures are only shown for cells in which there were 10 or more total follow‐up visits on which youths could have reported first‐gender change; as a result, ages 3–5 are not shown.

**TABLE 14:** Percent of Visits at Different Ages in Which Parents Reported First Change in Their Child's Gender

Age (Years)	% of Visits in Age Range Reporting First Gender Change (*N*)			
	Recruited as Transgender	Recruited as Cisgender	Recruited as Siblings	Total
3–5	7.1% (2)	0.0% (0)	3.2% (1)	3.1% (3)
6–8	2.9% (4)	0.0% (0)	1.5% (2)	1.3% (6)
9–11	2.6% (8)	1.4% (5)	3.0% (5)	2.2% (18)
12–14	3.5% (13)	4.1% (16)	4.8% (9)	4.0% (38)
15–17	2.9% (7)	2.5% (5)	8.5% (11)	4.0% (23)
18+	1.2% (1)	4.1% (3)	0.0% (0)	2.1% (4)

### IV.6 Results for RQ2: What are Youths' Current Identities and How Do They Align with the Youths' *Recruitment Genders*?

#### IV.6.1 Current Youth‐Reported Identities with Respect to Recruitment Gender

**Figure 7:**
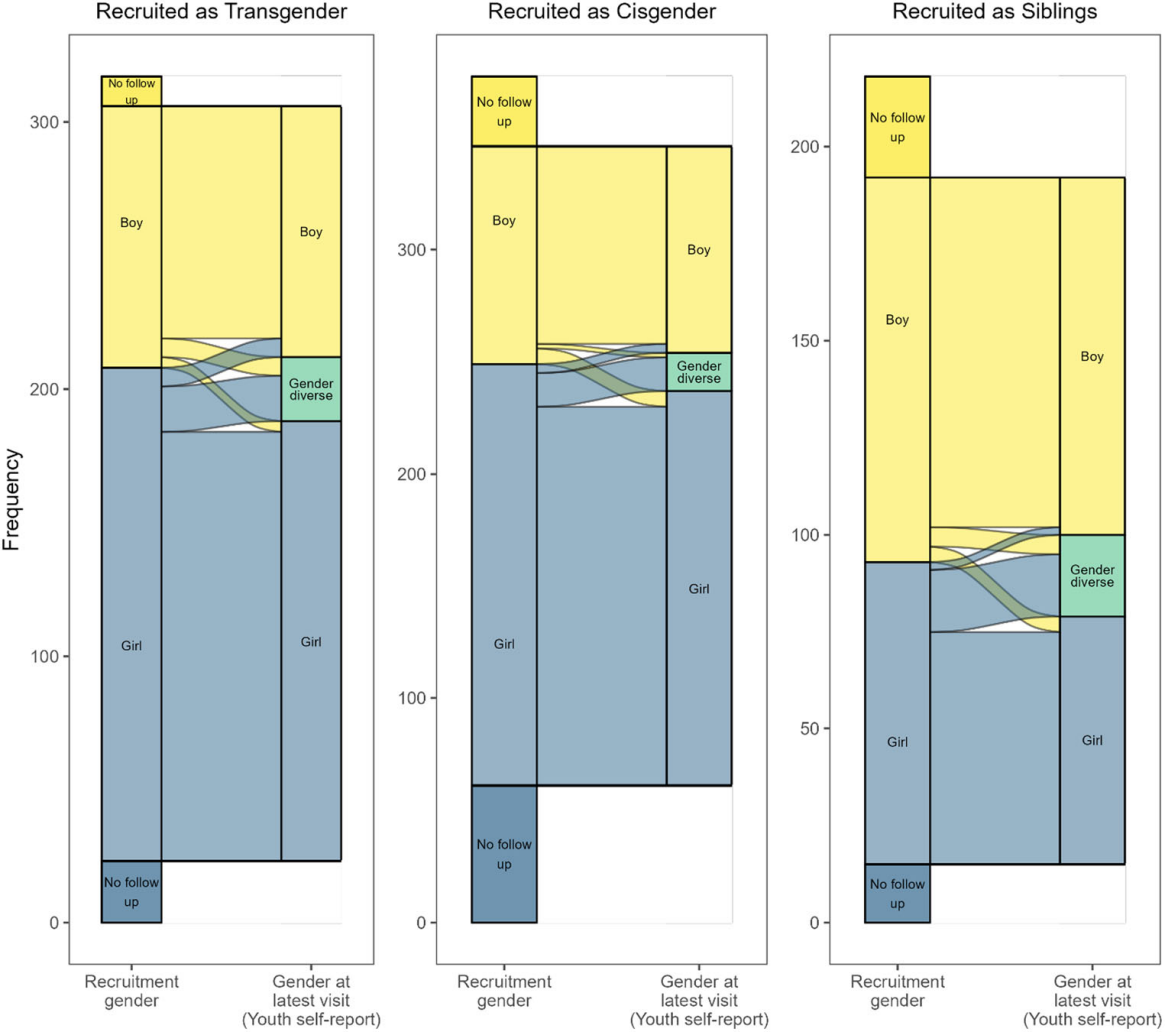
Relation between *recruitment gender* and youths' current gender identities, per youth report.

**Figure 8:**
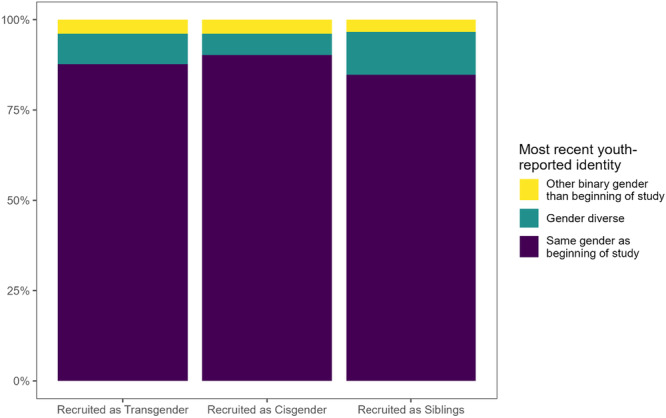
Most recent gender identity according to youth report, among youth with at least one codable follow‐up report.

Table 15 shows, and Figures 7 and 8 depict, current identities according to the youths themselves, among the 745 youths (81.7% of the total sample) who have given a codable report of their gender at a follow‐up visit. Of these, the vast majority 655 (87.9%) are currently identifying as their *recruitment gender*. Among youths who are currently a different gender than their *recruitment gender*, the majority are gender diverse, indicating that changing to a gender‐diverse identity is more common in this sample than undergoing a binary transition (relative to one's *recruitment gender*).

**TABLE 15:** Current Identities, Per Youth Report

Recruitment Group	Recruitment Gender	Total *N*	*N* with Codable Follow‐up (% of Total)	*N* (% of Youths With Codable Follow‐ups)			Mean Age at First Visit (Years)	Mean Age at Most Recent Codable Report (Years)
				Boy	Gender Diverse	Girl		
Recruited as Transgender	Boy	109	98 (89.9%)	87 (88.8%)	7 (7.1%)	4 (4.1%)	8.6	15.6
	Girl	208	185 (88.9%)	7 (3.8%)	17 (9.2%)	161 (87.0%)	7.6	14.5
Recruited as Cisgender	Boy	128	97 (75.8%)	88 (90.7%)	2 (2.1%)	7 (7.2%)	9.0	15.1
	Girl	249	188 (75.5%)	4 (2.1%)	15 (8.0%)	169 (89.9%)	8.2	14.5
Recruited as Siblings	Boy	125	99 (79.2%)	90 (90.9%)	5 (5.1%)	4 (4.0%)	7.5	13.7
	Girl	93	78 (83.9%)	2 (2.6%)	16 (20.5%)	60 (76.9%)	7.7	13.8

##### IV.6.1.1 Current Gender Identity Relative to *Recruitment Gender*, by *Recruitment Group*

To determine if *recruitment groups* differed in current identity relative to *recruitment gender* (i.e., whether their identity was the same or different), we fit a logistic mixed‐effects model predicting whether a youth's current identity matched their *recruitment gender* from a categorical fixed effect of *recruitment group*, a continuous fixed effect of *time elapsed* between the beginning of a youth's participation in the Trans Youth Project and their most recent report, and a random intercept for each family. A Wald χ^2^ test on this model found that neither *recruitment group,* Wald χ^2^(2) = 3.96, *p* = .138, nor *time elapsed*, Wald χ^2^(1) = 1.25, *p* = .263, were significant predictors of whether a youth's *recruitment gender* and current gender matched.

#### IV.6.2 Current Parent‐Reported Identities with Respect to Recruitment Gender

**Figure 9:**
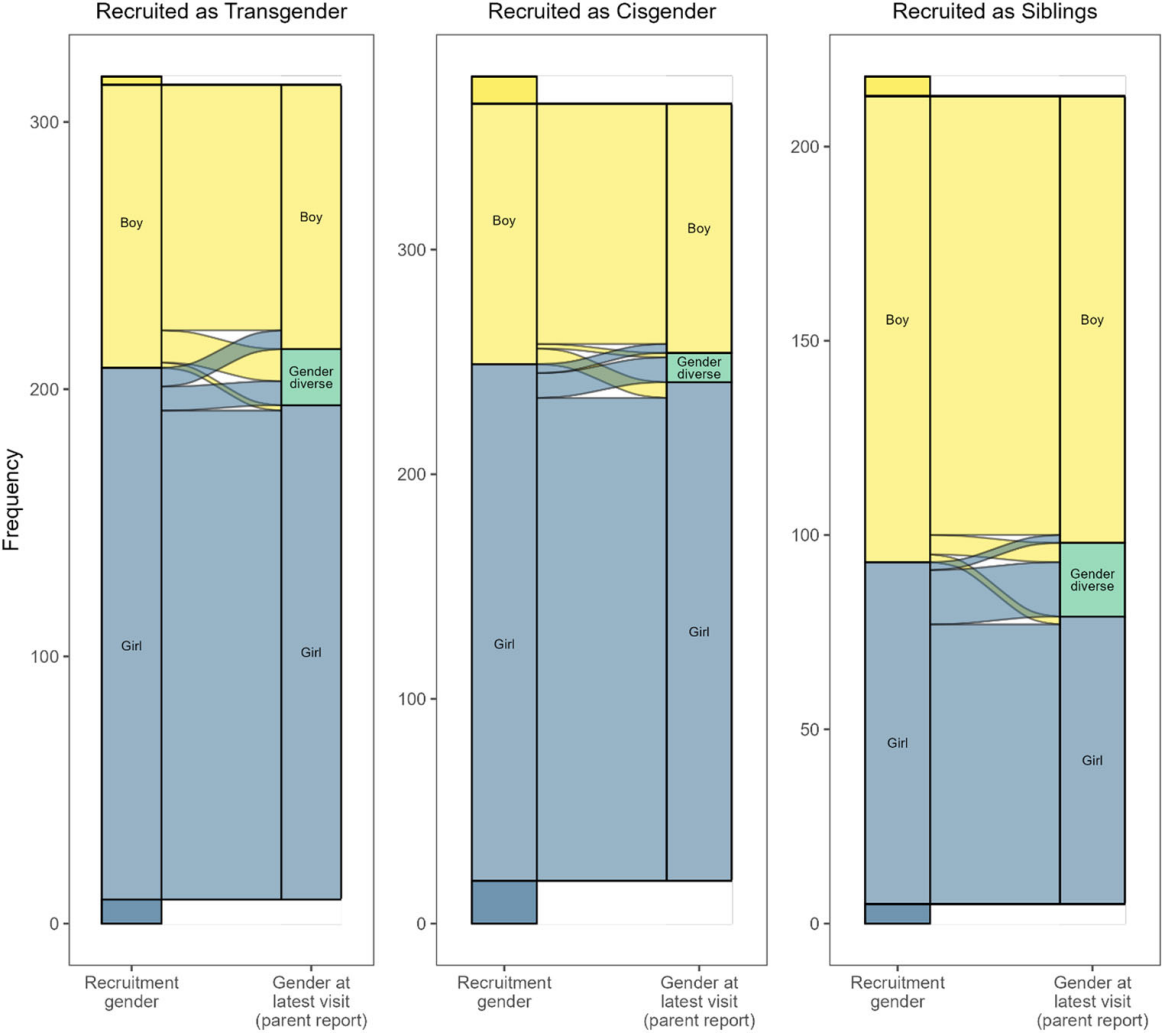
Relation between *recruitment gender* and youth's current gender identity, per parent‐report. *Note*: Darker yellow and blue overhanging portions on the top and bottom of the left side of each panel represent youths whose parents did not provide a codable report of the youth's gender on a follow‐up visit.

**Figure 10:**
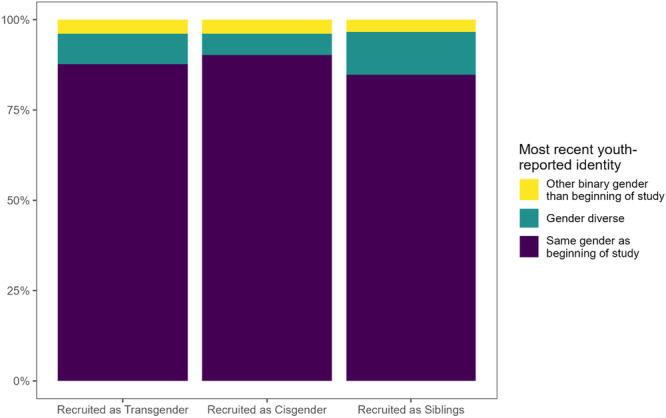
Most recent gender identity according to parent‐report, among youths with at least one codable follow‐up report from parents.

Table 16 shows, and Figures 9 and 10 depict, current youth identities according to parents among the 859 youths (94.3% of the total sample) whose parents have given a codable report of their child's gender at a follow‐up visit. Mirroring youths' most recent self‐reports of their gender identities, the vast majority of parents (91.0%) reported that their child identified as their *recruitment gender*, and among those who are currently a different gender, the majority are gender diverse.

**TABLE 16:** Current Youth Identity, Per Parent‐Report

Recruitment Group	Recruitment Gender	Total *N*	*N* With Codable Follow‐up (% of Total)	*N* (% of Youths With Codable Follow‐ups)			Mean Age at First Visit (Years)	Mean Age at Most Recent Codable Report (Years)
				Boy	Gender Diverse	Girl		
Recruited as Transgender	Boy	109	106 (97.2%)	92 (86.8%)	12 (11.3%)	2 (1.9%)	8.6	15.6
	Girl	208	199 (95.7%)	7 (3.5%)	9 (4.5%)	183 (92.0%)	7.7	14.8
Recruited as Cisgender	Boy	128	117 (91.4%)	108 (92.3%)	2 (1.7%)	7 (6.0%)	8.9	14.8
	Girl	249	230 (92.4%)	4 (1.7%)	11 (4.8%)	215 (93.5%)	8.0	13.8
Recruited as Siblings	Boy	125	120 (96.0%)	113 (94.2%)	5 (4.2%)	2 (1.7%)	7.7	14.2
	Girl	93	88 (94.6%)	2 (2.3%)	14 (15.9%)	72 (81.8%)	7.9	14

##### IV.6.2.1 Current Gender Identity Relative to *Recruitment Gender*, by *Recruitment Group*

To examine potential differences between *recruitment groups*, we fit a logistic mixed‐effects model predicting whether parent‐report of current gender matched the youth's *recruitment gender* from the following predictors: a categorical fixed effect of *recruitment group*, a continuous fixed effect of *time elapsed* between the beginning of a youth's participation in the Trans Youth Project and their parent's most recent report, and a random intercept for each family. Neither *recruitment group,* Wald χ^2^(2) = 2.87, *p* = .238, nor *time elapsed*, Wald χ^2^(1) = 3.08, *p* = .079, were significant predictors of whether a youth identified as their *recruitment gender* most recently.

#### IV.6.3 Comparing Youths' Current Identities by Reporter (Youth vs. Parent)

Did youths and parents systematically differ in their reports of the youth's identity at the youth's most recent visit? Of the 745 youths who have ever given a codable self‐report of their gender at a follow‐up visit, 691 (92.8%) also had a parent give a codable report of the youth's gender at the same (most recent) timepoint. Among this subgroup with overlapping participation, Table 17 shows contingency tables of youths' and parents' reports of the youth's identities in *Recruited as Transgender* group (*N* = 257), *Recruited as Cisgender* group (*N* = 277), and *Recruited as Siblings* group (*N* = 156). By far, the modal pattern in all three groups was that youths and parents agreed on the youth's gender identity.

**TABLE 17:** Contingency Tables of Youth‐ and Parent‐Reported Current Youth Identity

	Youth Report	Parent‐Report			% Agreement
		Boy	Gender Diverse	Girl	
Recruited as Transgender	Boy	80	5	0	94.6%
	Gender Diverse	0	11	8	
	Girl	0	1	152	
Recruited as Cisgender	Boy	90	1	0	96.4%
	Gender Diverse	1	9	7	
	Girl	0	1	169	
Recruited as Siblings	Boy	81	1	0	94.2%
	Gender Diverse	2	10	3	
	Girl	1	2	56	

*Note*: Only includes youths who had codable parent‐ and youth‐reports of gender at most recent visit.

### IV.7 Discussion

This chapter investigated (1) levels of stability and change in youths' gender identities and (2) the youths' current gender identities. Analyses revealed evidence of several core phenomena. First, stability in gender identity was the most common trajectory in all three *recruitment groups* (*Recruited as Transgender*, *Cisgender*, and *Siblings*). Most youths (83.1% according to youths themselves, 89.3% according to their parents) have not shown evidence of fluctuation in their categorical gender identity since the beginning of their participation, and as a result, most (87.9% according to youths, 91.0% according to their parents) currently identify as having the same gender they entered the study with.

The high levels of stability and continued identification as binary transgender in the *Recruited as Transgender* group (87.6% are currently binary transgender according to youth report) are notable given past work with clinical samples which found that only a minority of children who show gender‐nonconforming behavior and/or identity in childhood went on to identify as transgender by adolescence or adulthood (e.g., Singh et al., 2021; Drummond et al., 2008; Wallien & Cohen‐Kettenis, 2008; Green, 1987). It is difficult to know precisely what drives the apparent discrepancy between our sample and those of past work, but many factors that others have described could contribute (e.g., Olson et al., 2022; Olson, 2016; Ashley, 2022; Temple Newhook et al., 2018). In particular, it may be that the subset of youth who identify as transgender in childhood—presumably, a small subset of those who behave in gender‐nonconforming ways—have a different trajectory than those who do not. This would be consistent with findings from Rae et al. (2019), who observed that youths who go on to socially transition tend to show stronger gender nonconformity than those who do not even before they transition. It also accords with findings in the clinical literature that children with stronger gender nonconformity tend to show more gender dysphoria in adolescence and adulthood (Singh et al., 2021; Steensma et al., 2013a; Wallien & Cohen‐Kettenis, 2008). In other words, those gender‐nonconforming youth who assert a strong gender identity that contrasts with their sex at birth (i.e., a male child who identifies as a girl) are especially likely to live as transgender later.

There are other notable differences between the current *Recruited as Transgender* cohort and the samples in past studies; any of these differences may explain differences in outcomes. Of course, the youths in the *Recruited as Transgender* group in this study (and few of those in past studies) had socially transitioned. Furthermore, the *Recruited as Transgender* youths in this study generally had families who, at least by the time of enrollment in the study, supported their identities. In contrast, families in past clinic‐based work often approached those clinics because of concerns about their child's gender nonconformity, and youths in this work sometimes received therapy with an aim of changing their gender identities or gendered behavior (e.g., Green, 1987; Zucker & Bradley, 1995). The broader cultures of North America and Europe have shifted over time such that while transgender people continue to be subject to discrimination today, they experience generally higher rates of acceptance than they did when many of these studies occurred (Greenberg et al., 2019; Twenge, 2023a). Any of these differences, or a combination of them, might help explain why the results of the current study and past clinic‐based studies diverge.

Our results regarding the current gender identities of our *Recruited as Transgender* group also differ slightly from percentages reported in Olson et al. (2022)—a report of the identities of the same youths approximately 3–4 years earlier. While both reports suggest few youths in the *Recruited as Transgender* group (2%–4%) are living as cisgender, the current report finds notably higher rates of identification as gender diverse (and especially, nonbinary). This discrepancy may be in part due to the fact that we include data from youths themselves rather than just parents. As we saw here, there was a general trend such that youths were more likely to report gender‐diverse identities than parents. In addition, the Olson et al. (2022) report assessed gender based on the child's pronoun use as reported by parents, whereas this monograph uses more nuanced and a greater number of gender identity measures. Finally, several years have passed since the previous paper's analyses (that paper utilized data through 2020; current analyses ran through February 2024), allowing additional time for youths to experience gender change—a possibility made more plausible given that nonbinary identification among U.S. youths has increased in the past several years (Twenge, 2024).

Another primary finding was that levels of stability and change were roughly equivalent across all three *recruitment groups*. That is, the proportion who reported an identity other than their *recruitment gender* over the course of the study (so far) was approximately 15%–20% in all three groups according to youth self‐report and did not differ significantly among the groups. According to parent‐report, the proportion showing change was between 8% and 13%, but again we did not find significant differences among the groups.

Particularly among the *Recruited as Cisgender* and *Recruited as Siblings* groups, rates of reported change and eventual identification with gender‐diverse identities may seem high: we found that 9.8% of *Recruited as Cisgender* and 15.3% of *Recruited as Siblings* youths self‐reported a current gender identity other than their *recruitment gender*. However, our findings are relatively aligned with emerging evidence suggesting that youth are increasingly reporting gender‐diverse identities and thinking about gender more flexibly relative to earlier generations (Twenge, 2023a, 2023b, 2024; Brown, 2022; Trevor Project, 2021). For example, survey data from October 2018 from 5000 secondary students in a school district in the Northeastern United States found that 9.8% of youth surveyed indicated “incongruence between gender identity and sex assigned at birth” (Kidd et al., 2021), and approximately 5%–10% of 11th grade (16–17 years old) public school students surveyed in the 2022 Minnesota Student Survey indicated a gender diverse or transgender identity (Minnesota Department of Education, 2022). Furthermore, a recent paper that also recruited 18–22‐year‐old youth in the greater Seattle area, where most of our *Recruited as Cisgender* sample comes from, observed similar rates of gender‐diverse identification among young adults ages 18–22 (King et al., 2024). While it may be that the relatively high level of gender diverse and transgender identification among our *Recruited as Cisgender* and *Siblings* groups is related to their families' demographic profiles (predominantly white, middle‐to‐high SES, politically liberal), as we discuss in Chapter 7, there is little empirical evidence of disproportionate transgender identification by these demographic variables.

Regardless, these findings tell us that the rates of change we see—and, particularly, the rates of gender‐diverse identities—in our *Recruited as Transgender* sample likely reflect the broader socio‐cultural moment, rather than anything unique to the experiences of children who socially transition early. Similarly, the rate of retransition to live as cisgender among youths in the *Recruited as Transgender* group (3.9% according to youths, 3.0% according to parents) was remarkably parallel to the rates of transitioning to live as binary transgender among the *Recruited as Cisgender* group (3.9% according to youths, 3.2% according to parents).

While parents and youths generally reported the same overall trends, we did observe a significant difference by reporter on the question of whether youths' identities had ever changed during the study. Specifically, youth reported slightly more change than parents. There are several possible explanations for this pattern. It may be the case that some youths experience gender change without telling their parents or that youth generally think about their gender in less binary ways than their parents. Some recent work also suggests that young people—both transgender and cisgender—think more flexibly about gender than cisgender mature adults (Gallagher et al., 2025) and that younger people tend to see gender as existing on a spectrum more often than older people do (Geiger & Graf, 2019; Mintel, 2020; Puckett et al., 2022). In this study, we found it is not uncommon for youths to use a gender‐diverse label while their parents would use a label of “boy” or “girl” (Table 17; see also Figure 6, Chapter III). If this finding replicates in other samples, it is an intriguing avenue for continuing research on children's and adolescents' gender cognition.

A less interesting explanation for this discrepancy is that youths generally answered more measures about their gender identity at each visit than parents did, meaning that they had more opportunities to express nuanced or gender‐diverse identities which would have counted as gender change in the present analysis. A related issue is that we generally expect measures from young children to contain more noise than those from adults. For example, a 5‐year‐old may not be fully listening to a question and may give a seemingly “random” answer, which might cause us to inaccurately conclude that a youth's gender had changed even if it had not. It is challenging to rule for one of these alternatives over the others, and both possibilities are likely playing some role in this divergence.

### IV.8 Summary

We have presented evidence for several core observations in this chapter. First, among early‐transitioning transgender children as well as their counterparts who were cisgender in childhood, stability in gender identity was by far the modal trajectory, according to youth and parent‐report, most youths' current identities are the same as those they held at the beginning of the study. Second, to the extent that youths' identities did change, gender change was no more or less likely in any of the *recruitment groups* compared to the others. In other words, socially transitioned transgender youths were no more or less likely to show gender change throughout childhood and adolescence than youths who are cisgender in childhood. Youths appear to report slightly more change in their gender than their parents report about them, though all major patterns were replicated across data from youths and parents. In general, the most common changes of identity were to gender‐diverse identities. In all groups, approximately 15% of youths identified as gender diverse (e.g., nonbinary, agender, etc.) at the most recent assessment.

Overall, our findings point to the general conclusion that early‐identifying transgender children who are supported in a childhood social transition show similarly stable developmental trajectories as youth who were cisgender in childhood—a finding that contrasts with past work with clinical samples of gender‐ nonconforming youth, most of whom were not transgender or gender dysphoric in adolescence or young adulthood. We also found that gender change is not rare among youth who were cisgender and gender‐conforming in childhood. Nonetheless, these findings raise another question that we address in Chapter 5: Does the degree to which children show gender nonconformity in childhood predict which children ultimately report gender identity change?

## V Associations Between Childhood Gender Development and Current Gender Identity

### V.1 Chapter Highlights

- In this chapter, we test whether, among participants living as boys initially, those with more stereotypically feminine preferences, behaviors, and identity were more likely to identify as nonbinary or girls at their most recent Trans Youth Project visit. Similarly, we ask whether, among children who were initially identified as girls, those who were stereotypically masculine were more likely to recently identify as nonbinary or boys at their most recent Trans Youth Project visit.
- Technical details regarding how we tested these questions (e.g., statistical modeling choices, participant inclusion, etc.) can be found in **Technical Details** (under subheading ***Work in this Chapter***).

### V.2 Introduction

Is the degree of a child's gender nonconformity at all related to their gender identity outcome years later in adolescence or young adulthood? In this chapter, we address this question by examining if there is any relationship between childhood assessments of gender development (e.g., a child's self‐report identity on a continuous scale, or their gendered preferences for toys, clothing, or peers) and later gender identity. This question has generally not been asked in children assumed to be cisgender or without a history of significant gender nonconformity, presumably because the field of developmental psychology has typically conceptualized gender identity as a stable trait. Even if researchers had considered that some cisgender youth would later identify as transgender (or today's equivalent of genderqueer or nonbinary), they likely assumed such an outcome was so rare that aiming to predict it would be too difficult.

In contrast, researchers in clinical psychology and psychiatry have, on several occasions, asked if children who showed notable gender nonconformity later grow up to be transgender, show gender dysphoria, or otherwise have a non‐cisgender identity (e.g., Green, 1987; Zucker & Bradley, 1995). The largest and best‐documented studies in this literature come from two medical clinics that have treated gender‐nonconforming youth with psychotherapy and/or gender‐affirming medical care over much of the last half‐century: one that started in Utrecht, Netherlands, and then moved to Amsterdam (henceforth referred to as *Dutch clinic*), and another in Toronto, Canada (henceforth referred to as *Canadian clinic*; Drummond et al., 2008; Singh et al., 2021; Zucker & Bradley, 1995; Wallien & Cohen‐Kettenis, 2008; Steensma et al., 2013a). Participants were youths who were referred to these clinics for concerns related to their gender and/or the distress they were experiencing as a result of it and were clinically assessed between approximately 1980 and 2015. The Canadian clinic was shut down in 2015, while the Dutch clinic continues to see new patients. However, most reports of longer‐term follow‐up focus on youths whose intake occurred prior to 2010.

Upon intake in these clinics, youths and their parents were typically asked to complete a battery of standardized measures about the child's gender identity and expression (along with measures of mental health and wellbeing). One such measure was the Gender Identity Questionnaire for Children (*GIQ*; Johnson et al., 2004), a measure completed (despite the name) by parents that assesses a range of constructs including children's gender‐typed preferences for play activities, toys, and playmates, and children's feelings that they are or wish to be members of the other sex or have different sexual anatomy; full text of the measure can be found in Johnson et al. (2004). Several other youth‐report measures were used in the Dutch and Canadian clinics as well (Table 18).

**TABLE 18:** Summary of Longitudinal Literature with Clinically Referred Children and Adolescents.

Citation	Clinic	Participants	Gender Identity at Follow‐up	Predictors of Gender Identity at Follow‐up
Wallien & Cohen‐Kettenis, 2008	Dutch	77^a^ gender‐referred participants (59 assigned males, 18 assigned females), age 8.4 years on average at intake, age 18.9 years at follow‐up	27% of youths were gender dysphoric (“persisted”) at follow‐up	At intake, youths who ended up “persisting” were more likely to show stronger self‐reported “cross‐gender” identification on the Gender Identity Interview for Children (GIIC; Zucker et al., 1993); stronger parent‐reported “cross‐gender” behavior, preferences, and identification on the Gender Identity Questionnaire for Children (GIQ, labeled *Parent Questionnaire* in this chapter; Johnson et al., 2004); and were more likely to have fulfilled diagnostic criteria for Gender Identity Disorder.
Steensma et al., 2013a	Dutch	127^b^ gender‐referred participants (79 assigned males, 48 assigned females), age 9.2 years on average at intake, age 16.1 years on average at follow‐up	37% of youths were gender dysphoric (“persisted”) at follow‐up	At intake, youths who ended up “persisting”: were more likely to show stronger self‐reported “cross‐gender” identification (GIIC); had stronger parent‐reported “cross‐gender” behavior, preferences, and identification (*Parent Questionnaire*); “Behaved” and “wished” to be the opposite sex more often as children according to parents as indicated on the Child Behavior Checklist (CBCL; Achenbach, 1999; Verhulst et al., 1996); and were more likely to have fulfilled diagnostic criteria for Gender Identity Disorder.
Drummond et al., 2008	Canadian	25^c^ gender‐referred assigned‐female participants, age 8.8 on average at intake, age 23.2 on average at follow‐up	12% of participants were gender dysphoric (“persisted”) at follow‐up	Participants who ended up “persisting” were more likely to recall (at follow‐up) stronger “cross‐gender” behavior from childhood than those who were not gender dysphoric at follow‐up, based on the Recalled Childhood Gender Identity/Gender Role Questionnaire (Zucker et al., 2006).
Singh et al., 2021	Canadian	139 d gender‐referred assigned‐male participants, age 7.49 years on average at intake, age 20.58 years on average at follow‐up	12% of participants were gender dysphoric (“persisted”) at follow‐up	Researchers computed a composite of several measures assessed at intake: Draw‐a‐Person (Zucker et al., 1983); free play (Zucker et al., 1982); child‐reported “cross‐gender” identification (GIIC); cross‐sex peer preference; cross‐sex toy preference; and parent‐reported “cross‐gender” behavior preferences, and identification (*Parent Questionnaire*). Higher scores (indicating more “cross‐gender” behavior, preferences, and identity) on the composite predicted “persisting” gender dysphoria at follow‐up.

^a^ Includes *N* = 23 subjects who could not be reached for follow‐up.

^b^ Includes *N* = 28 subjects who could not be reached for follow‐up.

^c^ Does not include *N* = 12 subjects who could not be reached for follow‐up, declined to participate, were not available to participate, or whose parents declined to give researchers contact information for their children.

^d^ Does not include *N* = 19 subjects who could not be reached for follow‐up, *N* = 6 subjects who declined to participate, and *N* = 162 subjects whom the researchers did not attempt to contact due to resource constraints.

Information about participants (assigned sex and age at intake and follow‐up) as well as the key takeaway concerning childhood predictors of later identity from the Dutch and Canadian clinics are summarized in Table 18. While there are several other older studies that have reported rates of adolescent or adult transgender identities (or “gender identity disorder” as it was termed at the time) among gender‐ nonconforming male children, these studies typically provided no information about childhood assessments and had samples too small (*N*'s < 30; typically <10) to formally assess statistical relations between childhood and adolescence (e.g., Bakwin, 1968; Davenport, 1986; Kosky, 1987; Lebovitz, 1972; Money & Russo, 1979; Zuger, 1966). For this reason, we focus on the larger and better‐documented studies that occurred in Canada and the Netherlands.

Each of the studies described in Table 18 asked whether any of the previously described gender development measures from childhood predicted whether the participant was gender dysphoric at follow‐up. The first three studies in Table 18 reported that children who generally expressed more desire to be members of the opposite sex (or conviction that they were members of the opposite sex) and children with stronger “cross‐gender” preferences, behaviors, and identity, were more likely to present gender dysphoria at follow‐up. One study also found that participants who were gender dysphoric at follow‐up were also more likely to retroactively recall stronger gender‐nonconforming behavior in childhood (Drummond et al., 2008).

In addition to the work reviewed above, earlier work from our research team has examined whether childhood preferences and identity are associated with later gender transition (Rae et al., 2019). Participants in this work were *not* participants in the Trans Youth Project (and thus are not reported in this monograph); rather, they were gender‐nonconforming youths who were recruited around a similar time as the *Recruited as Transgender* group, but who had *not* undergone a social transition at the time of recruitment (unlike youths in the current *Recruited as Transgender* group, all of whom had already socially transitioned when the study began). Rae et al. (2019) found that those who went on to socially transition (i.e., went on to live as the “other” gender) showed stronger stereotypically “cross‐gender” identity and preferences for toys, clothing, and peers than those who did not before the transition actually occurred (Rae et al., 2019). This work suggests that even before a social transition, there may be meaningful variability among gender‐nonconforming children, and that those who go on to live or identify as binary transgender might be those with especially strong “cross‐gender” (or what, before transition, appear to be “cross‐gender”) preferences and identities.

#### V.2.1 Work in this Chapter

In this chapter, we are interested in asking if boys who are more feminine in childhood are more likely (than boys who are more masculine) to later live as girls or nonbinary people—and if more masculine girls are more likely than feminine girls to live as boys or nonbinary people later in life. To test these questions, we take three measures from childhood—children's continuous measure of gender identity (*Continuum*; Question 1), a composite measure of their toy, clothing, and peer preferences (*Preference*; Question 2), and a parent‐report measure of their gender identity and expression [Gender Identity Questionnaire (Johnson et al., 2004), henceforth referred to as *Parent Questionnaire*; Question 3]—and assess whether they are related to later identity outcomes. Table 19 provides an overview of these three measures; more detailed information about their administration and scoring appears in the Supporting Information for Chapters III (*Continuum*) and V (*Preference* and *Parent Questionnaire*).

**TABLE 19:** Overview of Gender Development Measures Used in this Chapter

Measure	Description
*Continuum*	Youth‐report identity measure on a scale of 0 (feeling totally like a boy)—100 (feeling totally like a girl)
*Preference*	Composite of three separate measures of gender‐typed preferences:
	1. Peer preference (6 trials, 2 alternative forced choice between a boy and a girl) 2. Toy preference (4 trials, 5 alternative forced choice, choosing among 5 toys ranging from stereotypically masculine to feminine) 3. Clothing preference (4 trials, 5 alternative forced choice, choosing among 5 outfits ranging from stereotypically masculine to feminine)
*Parent Questionnaire* (originally Gender Identity Questionnaire; Johnson et al., 2004)	Sixteen‐item measure asking parents to report on their child's gender typicality (e.g., in terms of playing with same‐ and other‐sex dolls, imitating same‐ or other‐sex characters on TV, etc.) and the extent to which the child likes being their assigned sex or desires to be another gender.

We chose to examine these three childhood measures because they correspond most closely with childhood gender development measures used as predictors of later identity in prior literature: the *Parent Questionnaire* has been used in past work in the Dutch and Canadian clinics, and these clinics also included assessments of youths' gender‐typed preferences for toys, clothes, and peers (e.g., Singh et al., 2021; Wallien & Cohen‐Kettenis, 2008; Steensma et al., 2011), similar to our *Preference* measure. The *Continuum* was introduced in a past TYP publication (Gülgöz et al., 2022) and thus was not used in the Dutch and Canadian clinics; however, these clinics did use various measures that aimed to capture how strongly a youth did or did not identify with their assigned sex on a continuous scale.

In these analyses, we only include youths who identified as a girl or boy when the childhood measure was collected as there were not enough gender‐diverse youths for full analysis. If youth or parents completed these measures multiple times, we used the first time they completed the childhood measure in the analyses. Their latest measure of gender identity (i.e., current gender identity) is used as the outcome variable in these analyses. For inclusion in these analyses, current identity had to be collected at a later visit than the childhood measure (since we were not interested in predicting contemporaneous identity).

##### V.2.1.1 Technical Details

Here, we detail more in‐depth technical information about the work in this chapter for interested readers.

##### V.2.1.2 Terminology and Alternative Coding

Throughout this chapter, we use the term “current gender identity”, by which we mean the identity that a youth had at their most recent visit with the research team (the last time we received a codable identity from them before February 2024; see Chapter III for more on what counted as “codable”). In this chapter's analyses, boy‐expansive and girl‐expansive visit‐level codes are treated as boy and girl, respectively (see Chapter 3 for details of visit‐level coding); in the Supporting Information, we report parallel results if the expansive categories are treated as gender diverse instead.

##### V.2.1.3 Analytic Approach and Modeling Decisions

Our key questions in this chapter concern whether scores on a particular continuous measure from earlier in development (*Continuum*, *Preference*, or *Parent Questionnaire*) are related to youths' current gender identity. First, we asked whether youths who identified as boys in childhood—but today are girls or gender diverse—showed more stereotypically feminine early scores than did those who continue to identify as boys today. To do so, we first attempted to fit a linear mixed‐effects regression model predicting boys' early scores on each continuous dependent measure (*Continuum*, *Preference*, or *Parent Questionnaire*) from a three‐level categorical factor of *current identity* (whether the youth is currently still a boy, is now a girl, or is now gender diverse) and a random intercept for each family to account for nonindependence introduced by sibling pairing in the data set. We then ran a type‐II Wald chi‐square test to determine whether *current identity* was significantly related to early scores on the dependent measures. Next, using the same modeling strategy, we asked the parallel question for youths who were girls at their early reports; that is, we asked whether these youths' scores on the early continuous measures differed as a function of whether the youth is currently a girl, a boy, or gender diverse. In cases in which three‐level factors were significant, we ran pairwise follow‐up Tukey adjusted comparisons.

We adjusted models from the above specification in two cases. First, in some cases, there were fewer than 10 youths in one of the three levels of the *current identity* factor (e.g., there were often fewer than 10 included youths who were girls in childhood and are now boys). In these cases, to adhere to our minimal cell size rule throughout the monograph, we collapsed the *current identity* variable into a two‐level factor comparing those who are the same binary gender today as they were at the early report, and those who are not. Due to small *N*'s of youths who have changed identity during the study, this was necessary in five of the six analyses we report in this chapter. Second, when linear mixed‐effects models resulted in singular fits or did not converge, we removed the random intercept for each family. In these cases, we report *t*‐statistics (in cases in which we are comparing two groups) or *F*‐statistics (in cases in which we are comparing three groups).

**Figure 11:**
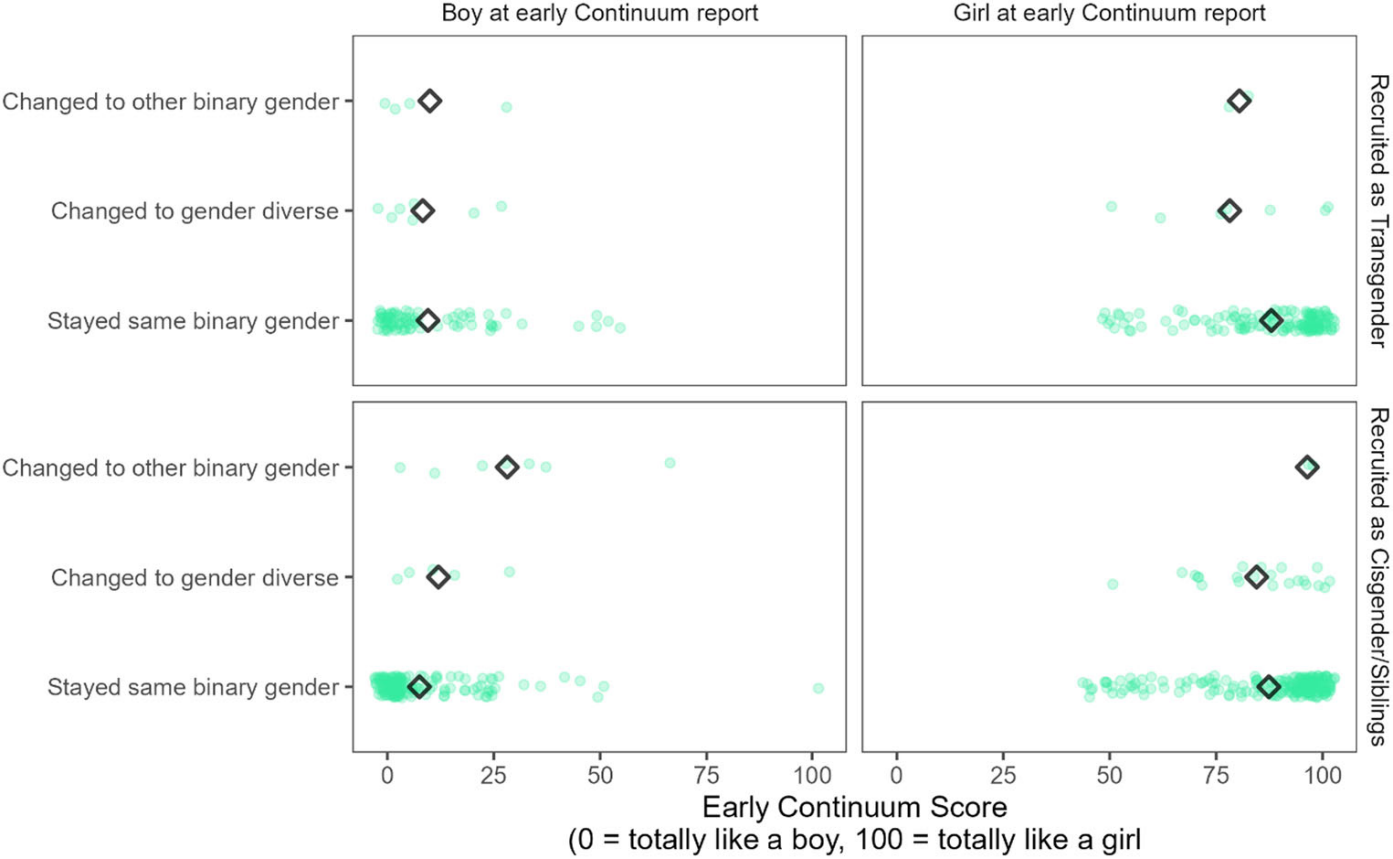
Early continuum scores by gender at time of report and recruitment modality. *Note*: Black diamonds represent means; points are jittered for visual clarity.

**Figure 12:**
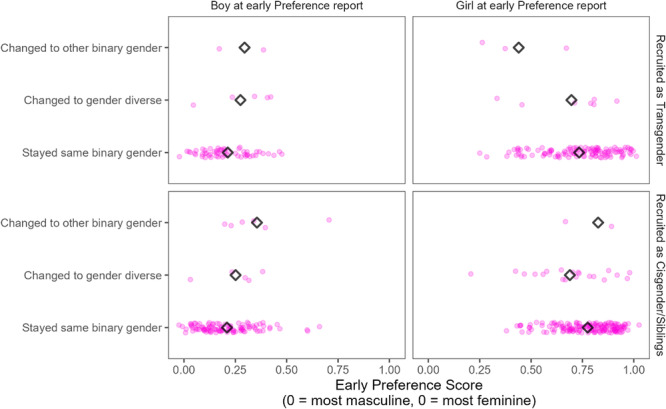
Early preference scores by gender at time of report and recruitment modality. *Note*: Black diamonds represent means; points are jittered for visual clarity.

**Figure 13:**
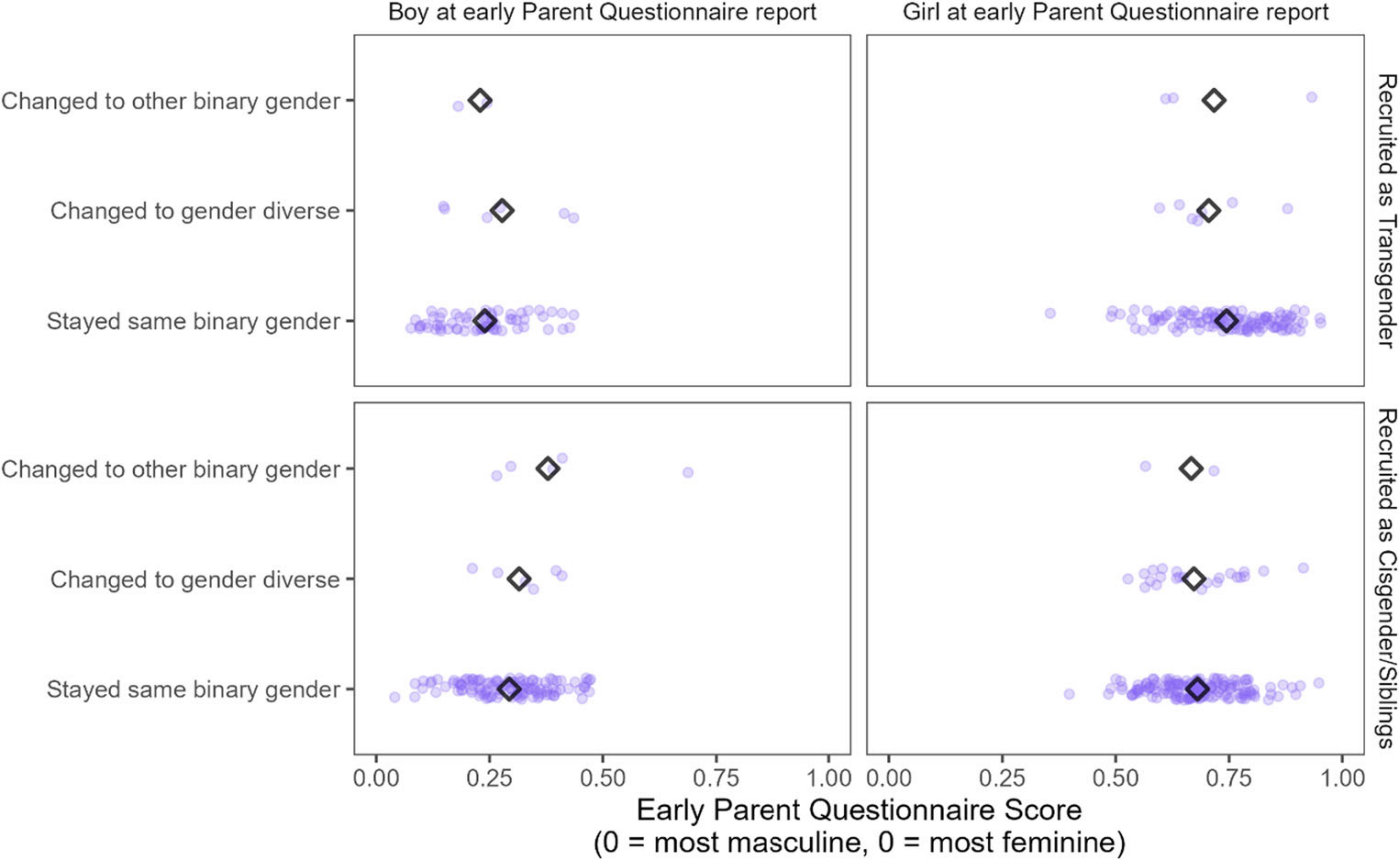
Early parent questionnaire scores by gender at time of report and recruitment modality. *Note*: Black diamonds represent means; points are jittered for visual clarity.

While we were not able to include a predictor of *modality* (i.e., test whether the relation between these continuous measures and gender change hinges on whether a youth is initially transgender or cisgender) due to small *N*'s, we visualize participant scores broken down by whether the youth is in the *Recruited as Transgender* group versus in the *Recruited as Cisgender* or *Recruited as Siblings* groups in Figures 11, 12, and 13.

### V.3 Results

#### V.3.1 Question 1: Continuum and its Relation to Current Gender Identity

Among all 912 youths in the study, 618 (67.8%) provided a *Continuum* report that could be included in this analysis. On average, this early *Continuum* report came at 10.3 years old, and their most recent categorical report of gender identity came at 14.8 years old. Figure 11 shows participant scores.

##### V.3.1.1 Boys at Early *Continuum*

Among youths who were boys at their early *Continuum* report (*N* = 249, *M*_age at early *Continuum*_ = 10.7 years, *M*_age at most recent gender report_ = 15.18), early *Continuum* scores differed significantly among youths who are currently still boys (*N* = 226, *M*_*Continuum*_ = 8.25, *SD*_*Continuum*_ = 13.12), those who are now girls (*N* = 11, *M*_*Continuum*_ = 21.56, *SD*_*Continuum*_ = 19.45), and those who are now gender diverse (*N* = 12, *M*_*Continuum*_ = 9.85, *SD*_*Continuum*_ = 11.11), Wald χ^2^(2) = 10.44, *p* = .005. Post‐hoc Tukey tests on estimated marginal means found that youths who are currently boys showed significantly more masculine scores than youths who are currently girls, *t*(246) = 3.20, *p* = .004; no other pairwise comparisons were significant (*p*'s > .09).

##### V.3.1.2 Girls at Early *Continuum*

Due to small *N*'s, for youths who were girls at their early *Continuum* report (*N* = 369, *M*_age at early *Continuum*_ = 10.0 years, *M*_age at most recent gender report_ = 14.56), we compared early *Continuum* scores between two groups of participants: those who are currently girls, and those who are not (i.e., are boys or gender diverse). We removed the random intercept for each family as it led to a singular fit. Early *Continuum* scores did not differ significantly between youths who are currently girls (*N* = 336, *M*_*Continuum*_ = 87.64, *SD*_*Continuum*_ = 15.28) and those who are currently boys or gender diverse (*N* = 33, *M*_*Continuum*_ = 83.65, *SD*_*Continuum*_ = 14.04), *t*(367) = 1.44, *p* = 0.151.

#### V.3.2 Question 2: Preference and its Relation to Current Gender Identity

Among all 912 youths in the study, 482 (52.6%) gave a response on *Preference* that could be used in the present analysis. On average, this early *Preference* report came at 8.4 years old, and their most recent categorical report of gender identity came at 13.8 years old. Figure 12 shows participant scores.

##### V.3.2.1 Boys at Early *Preference*

Among youths who were boys at their early *Preference* report (*N* = 176, *M*_age at early *Preference*_ = 8.5 years, *M*_age at most recent gender report_ = 13.9 years), we compared early *Preference* scores between those who are currently boys (*N* = 158, *M*_*Preference*_ = 0.21, *SD*_*Preference*_ = 0.13) and those who are currently girls or gender diverse (*N* = 18, *M*_*Preference*_ = 0.30, *SD*_*Preference*_ = 0.16) due to small *N*'s. Youths who are currently boys had significantly more masculine early *Preference* scores than those who are currently girls or gender diverse, *t*(173.98) = −2.68, *p* = .008.

##### V.3.2.2 Girls at Early *Preference*

For youths who were girls at their early *Preference* report (*N* = 306, *M*_age at early *Preference*_ = 8.4 years, *M*_age at most recent gender report_ = 13.7 years), we compared early *Preference* scores between those who are currently girls (*N* = 274, *M*_*Preference*_ = 0.76, *SD*_*Preference*_ = 0.16) and those who are currently boys or gender diverse (*N* = 32, *M*_*Preference*_ = 0.68, *SD*_*Preference*_ = 0.20) due to small *N*'s. Youths who are currently girls had significantly more feminine early *Preference* scores than those are currently boys or gender diverse, *t*(301.68) = 2.76, *p* = .006.

#### V.3.3 Question 3: Parent Questionnaire and its Relation to Current Gender Identity

Among all 912 youths in the study, 519 (56.9%) had a *Parent Questionnaire* report from a primary parent that could be used to predict current identity. On average, this early *Parent Questionnaire* report came at 8.7 years old, and their most recent categorical report of gender identity came at 14.2 years old. Figure 13 shows participant scores.

##### V.3.3.1 Boys at Early Parent Questionnaire

Among youths who were boys at their early *Parent Questionnaire* report (*N* = 198, *M*_age at early *Parent Questionnaire*_ = 8.9 years, *M*_age at most recent gender report_ = 14.4 years), we compared early *Parent Questionnaire* scores between those who are currently boys (*N* = 179, *M*_*Parent Questionnaire*_ = 0.27, *SD*_*Parent Questionnaire*_ = 0.09) and those who are currently girls or gender diverse (*N* = 19, *M*_*Parent Questionnaire*_ = 0.31, *SD*_*Parent Questionnaire*_ = 0.11) due to small *N*'s; the two groups did not significantly differ, *t*(191.19) = −1.62, *p* = .107.

##### V.3.3.2 Girls at Early Parent Questionnaire

For youths who were girls at their early *Parent Questionnaire* report (*N* = 321, *M*_age at early *Parent Questionnaire*_ = 8.6 years, *M*_age at most recent gender report_ = 14.0 years), we compared early *Parent Questionnaire* scores between those who are currently girls (*N* = 286, *M*_*Parent Questionnaire*_ = 0.71, *SD*_*Parent Questionnaire*_ = 0.10) and those who are currently boys or gender diverse (*N* = 35, *M*_*Parent Questionnaire*_ = 0.68, *SD*_*Parent Questionnaire*_ = 0.10) due to small *N*'s; the two groups did not significantly differ, *t*(317.14) = 1.47, *p* = .143.

### V.4 Discussion

This chapter examined whether continuous measures of gender development in childhood—namely, identity on a continuous scale (*Continuum*), gender‐typed preferences (*Preference*), and parent‐report gender typicality on a commonly used scale in past work with gender‐diverse youths (*Parent Questionnaire*)—were related to their gender identity in adolescence. Results from this chapter's analyses, which are summarized in Table 20, were mixed, but generally indicated that scores on the *Preference* measure were most reliably related to later gender outcomes, whereas *Continuum* and *Parent Questionnaire* scores showed inconsistent or null results.

**TABLE 20:** Summary of Relations Between Continuous Gender Development Measures in Childhood and Identity in Adolescence

Measure	Brief Description of Measure	Result With Boys at Early Report of Measure	Result With Girls at Early Report of Measure
*Continuum*	Youth‐reported continuous gender identity on a 0–100 scale	Current boys more masculine than current girls; neither group differed from currently gender diverse	No difference between current girls and current boys/gender diverse
*Preference*	Youth‐reported gender‐typed preferences for toys, clothing, and peers	Current boys more masculine than current girls/gender diverse	Current girls more feminine than current boys/gender diverse
*Parent Questionnaire*	Parent‐report gender typicality	No difference between current boys and current girls/gender diverse	No difference between current girls and current boys/gender diverse

Findings with the *Preference* measure were generally consistent with past results in the literature; in contrast, we found mixed evidence with *Continuum*, and no significant relation between the *Parent Questionnaire* and later gender identity. Studies of youth who were assessed in gender clinics have typically found that those who tended to show stronger “cross‐gender” identification and preferences in childhood were more likely to be transgender (or gender dysphoric) adolescents or young adults (Wallien & Cohen‐Kettenis, 2008; Steensma et al., 2013a; Drummond et al., 2008; Zucker & Bradley, 1995; Singh et al., 2021). Rae and colleagues (2019) similarly found that children who later went on to socially transition showed greater gender nonconformity before that transition than those who, 2 years later, had not gone on to transition. Together, the current and past work suggest that childhood gender nonconformity is, at the group level, related to later identity. That said, we urge caution in using any given individual child's early gender development to infer a later identity given that, as shown in Figures 11, 12, and 13, score distributions overlap between groups of youth with divergent later gender outcomes—and the effects are generally small.

A notable aspect of our work—and a key difference from past work with clinical samples—is that we included youths who, at the start of the study, were cisgender and (as far as we were made aware) did not have gender histories that had suggested to their families that they would be anything other than cisgender later. Yet within this sample, as discussed in Chapter IV, several *have* gone on to identify as nonbinary and/or transgender, allowing us to examine the relation between childhood gender nonconformity and later gender trajectories in this group of youths—in addition to those who showed significant gender nonconformity, on whom most past work on this question has focused.

Because the number of youths who have shown gender change in all three *recruitment groups* is relatively small (fewer than 20% in each group), the overall *N*'s of youths who are currently a different gender than they were when they answered each of our continuous measures is quite small. As a result, we were unable to test whether the predictive value of these childhood measures does or does not differ based on whether a particular youth is transgender or cisgender; we were also not able to test for gender differences (i.e., if there was more predictive signal for boys than girls). We hope that future work featuring larger samples of youths who go through changes in gender can address these issues given that we were unable to here.

### V.5 Summary

We found some evidence that youth‐reported continuous identity (among children who identified as boys) and youth‐reported gender‐typed preferences (among children who identified as boys and those who identified as girls) were associated with later gender change. Parent‐report gender typicality on a questionnaire used with clinical samples in past work showed no reliable relationship with later gender change. Sample size limitations inhibited our ability to test whether these relationships were or were not moderated by gender modality (i.e., whether a youth was transgender or cisgender as a child). While the relative rarity of gender change makes such trajectories difficult to predict statistically without very large samples, we observed some convergent evidence with past work demonstrating that stronger gender nonconformity in children is related to identifying as transgender in adolescence and young adulthood.

## VI Development, Stability, and Change in Sexual Orientation Across Childhood and Adolescence

### VI.1 Chapter Highlights

- We describe how youths in the Trans Youth Project self‐described their sexual orientation identities and discuss how much our sample does or does not exemplify national trends showing increasing queer identification among young people (for this topic, see **Research Question 1: Overall Rates of Sexual Orientation Identification**).
- We test whether teenage boys and girls in our sample who were more gender nonconforming in childhood (according to several measures of childhood gender development) are more likely to be queer today (for this topic, see **Research Question 2: What is the Relation Between Earliest Gender Development Measures and Current Sexual Orientation?**).
- We describe how much participants have or have not shown change in sexual orientation across the longitudinal study, whether certain trajectories of change are particularly common, and whether youths in different *recruitment groups* showed different levels of change (for this topic, see **Research Question 3: Stability and Change in Attraction Over Time**).

### VI.2 Introduction

Sexual orientation and its development across the lifespan have been topics of interest in psychology for decades. Research in this area often centers on the percentage of the population that is straight vs. queer (e.g., lesbian, gay, bisexual, pansexual, or any other identity that is not straight), how rates of identifying with these categories may or may not be changing over time, differences by sex or gender, and how stable (or not) sexual orientation is within individuals. Despite a focus on these questions in the scientific literature for some time, interest in them has risen in the past decade as several broad social changes appear to be occurring, particularly among young people.

First, the number of young people coming out as queer has substantially increased. Research from the survey analytics company Gallup indicated that well over 20% of Gen‐Z people in the United States identified as queer in 2023 and 2024, compared to 9%–14% of Millennials and 4%–5% of Generation X (Jones, 2024, 2025); other data from the Youth Risk Behavior Survey, a national survey administered by the United States federal government, showed a 10% *decrease* in identifying as heterosexual among U.S. high school students (roughly ages 14–18) between 2019 and 2021. Second, there has been an unprecedented shift (at least in modern history) toward acceptance of (at least some) queer identities among the general public in the United States, with 69% of Americans in 2024 indicating that gay marriage should be accepted, up from 44% in 2010 (Gallup, 2024). These developments have occurred contemporaneously with the emergence of an increasingly visible cohort of transgender and gender‐diverse youth whose experiences with sexual orientation have not been documented as much as those of cisgender youth (e.g., Suarez et al., 2024).

Our focus in this chapter is on sexual orientation among participants in the Trans Youth Project (TYP), an ongoing longitudinal study of transgender and cisgender youth which began in 2013. The TYP presents a singular opportunity to investigate these recent trends as it includes information about sexual orientation in a group of transgender and cisgender youths whom our research team has followed as these societal changes have occurred in the United States (where 98% of the sample lives). In that sense, it is a unique prospective study of sexual orientation over time in a group that was not recruited based on sexual orientation. We focus on three main goals in this chapter: (1) describing sexual orientation among youths in our sample and how it does or does not differ based on gender identity (i.e., boy, girl, or gender diverse) or modality (i.e., transgender or cisgender); (2) examining whether gender‐typed behavior or preferences in childhood are related to current sexual orientation; and (3) examining sexual orientation stability and change across development, including whether levels of stability and change differ by gender identity and modality.

#### VI.2.1 Historical Estimates of Prevalence of Queer Identity

Beginning with Kinsey's foundational volumes *Sexual Behavior in the Human Male* and *Sexual Behavior in the Human Female* (published in 1948 and 1953, respectively) and continuing through the 2000s, researchers have attempted to estimate how much of the population can be classified as not straight—either because of attractions, sexual behavior, or identity labels (all of which are facets of sexual orientation which may or may not align within individuals; see subsection “Conceptualization of Sexual Orientation and Terminology” later this chapter for more discussion). Estimates vary widely, in part because of heterogeneity in how sexual or romantic attractions are operationalized or assessed in different work (Schick et al., 2014). A brief review by the Kinsey Institute at Indiana University suggests that among some of the most well‐known work in this literature from 2011 or prior, estimates of non‐heterosexual behavior or identification usually land somewhere between 2% and 10% (Kinsey Institute, 2011) though some work has suggested higher rates of up to 15%–20% (Bagley & Tremblay, 1998; Gonsiorek et al., 1995).

#### VI.2.2 Fluidity in Sexual Orientation

Traditional conceptions of sexual orientation consider it to be a fixed trait once it is realized in adolescence; however, large studies often report fluctuations for as much as 20% of sexual minority populations (Savin‐Williams & Ream, 2007; Savin‐Williams et al., 2012; Ott et al., 2011; Dickson et al., 2003; Dickson et al., 2013). Women and girls, who tend to be more likely to report bisexuality than men and boys (Chandra et al., 2013; Dickson et al., 2003; Dickson et al., 2013; Mosher et al., 2005; Savin‐Williams et al., 2012), have sometimes been shown to report more fluidity in sexual orientation over time than men and boys (Dickson et al., 2003; Mock & Eibach, 2012; Ott et al., 2011; Savin‐Williams et al., 2012; Stewart et al., 2019), though some studies have not found this effect (Katz‐Wise, 2015; Rosario et al., 2006). Longitudinal work in the past two decades in the United States has suggested that sexual orientation change is not uncommon among youths—and may perhaps even be modal among transgender and gender‐diverse youths (Katz‐Wise et al., 2016; 2023a; 2023b; 2024; Stewart et al., 2019).

#### VI.2.3 Sexual Orientation in Transgender Youths

In general, sexual orientation among self‐identified transgender youths was rarely studied until about a decade ago. An older literature centered mainly in the field of sexology, however, has argued that transgender women who showed initial signs of their transgender identity very early in life—like most of the trans girls in the Trans Youth Project—tended to be exclusively interested in men (i.e., straight, by our definition), as compared to those who come out as transgender in later adolescence or adulthood who were not (Blanchard, 1988; Bailey, 2003). While this theory has been heavily criticized (e.g., Serano, 2020; Winters, 2008), most of that criticism has focused on its claims about the sexuality of transgender women who come out later in life. In the current study, we can investigate the claim concerning transgender girls who show notable femininity early in life—namely, that they will later be attracted only to men—and whether it holds in a sample of contemporary transgender girls.

Recent work suggests this may not be the case. Specifically, transgender and gender‐diverse youth in contemporary studies tend to report high rates of queer identities—often higher rates than cisgender youth, even though the latter group's rates of queer identification are also increasing (Boskey & Ganor, 2022; Bosse & Chiodo, 2016; Bungener et al., 2017; Gower et al., 2022; Olson et al., 2015; Price‐Feeney et al., 2020; Salk et al., 2020; Szoko et al., 2023). However, these recent studies showing high queer identification among transgender adolescents do not typically indicate whether these youth came out early in childhood or not; our study thus presents an opportunity to test this question directly given that our sample includes a large number of youths who came out as transgender early in life.

#### VI.2.4 Relation Between Childhood Gender‐Typed Behavior and Later Sexual Orientation

In this chapter, we also examine whether childhood gender‐typed behavior is related to later sexual orientation. Both retrospective studies (in which adults recall behavior from their childhood) and prospective studies (in which participants' behaviors are measured in childhood and identity is assessed later in life) have demonstrated that stronger “cross‐gender” behavior in childhood, such as interest in playing with toys or wearing clothes typically associated with people of the other binary gender, is associated with queer sexual orientation in adolescence or adulthood (Steensma et al., 2013b; Bailey & Zucker, 1995). In particular, a recent prospective study with over 4500 youths found that “cross‐gender” behavior (e.g., preferring to play house for boys and enjoying rough‐and‐tumble play for girls) at 3.5–4.75 years predicted sexual orientation at 15 (Li et al., 2017). Among the samples described in Chapter V from the Dutch and Canadian gender clinics, high proportions of participants who showed strong gender nonconformity as children reported identifying as, or showing behaviors and attractions consistent with being, cisgender gay or bisexual adults (e.g., Drummond et al., 2008; Singh et al., 2021; Steensma et al., 2013b; Wallien & Cohen‐Kettenis, 2008). Thus, there is evidence that males with more stereotypically feminine interests and behavior in childhood are more likely to be queer (cisgender) adolescent boys or men; likewise, females with more stereotypically masculine interests and behavior in childhood are more likely to be (cisgender) queer adolescents or adults. In this chapter, we provide another test of this question, along with a unique opportunity to ask if these patterns extend to samples that include transgender youth.

### VI.3 Current Work in this Chapter

In this Chapter, we contribute to the above literature by reporting on sexual orientation over time among participants in the Trans Youth Project. In particular, we address three major research questions:

(1) What is the current sexual orientation of participants, and does it differ by their current gender identity or current gender modality?
(2) Are childhood reports of (a) gender identity as measured on a continuous spectrum (*Continuum*), (b) gender‐typed preferences for toys, clothing, and peers (*Preference*), and (c) scores on a parent‐report measure of gender typicality (*Parent Questionnaire*) related to the youth's most recently reported sexual orientation and does this differ by their current gender identity or current gender modality?
(3) How much stability and change in sexual orientation have youths reported, and does it differ among youths who began the study as transgender vs. cisgender?

#### VI.3.1 Chapter‐specific Decisions

To answer these research questions, we made several decisions about how we would analyze data and present results, detailed below.

##### VI.3.1.1 Conceptualization of Sexual Orientation and Terminology

Several contemporary researchers (e.g., Diamond, 2016; Katz‐Wise et al., 2017; Mustanski et al., 2014; Stewart et al., 2019; Ybarra et al., 2019) have pointed out that identity labels, romantic attraction (generally thought of as desire for intimate emotional connection), and sexual attraction (generally thought of as sexual interest based largely on attraction to physical characteristics) are not always concordant within individuals, and it is often appropriate to consider them as separate constructs. However, heterogeneity in the measures used across the study period made it difficult in many cases to determine whether youths were reporting on their sexual or romantic attractions in a particular visit (or both). Furthermore, in visits where both sexual and romantic attraction toward certain gender groups were assessed in separate 1–7 scales (see Measures 7–9, Table 22, later this chapter), we found close correspondence between the two (Pearson's *r*'s > 0.83). Thus, when discussing what group of people a youth is attracted to, we do not make a distinction between sexual attraction and romantic attraction and subsume both under the label of *attraction*. When we use the term sexual orientation in the rest of the chapter, we are referring to an even broader construct of which sexual and romantic attraction to particular groups are one part (including, for example, whether someone is asexual and/or questioning, which we also assessed in this project).

At some points in this chapter, we were tasked with describing the varied and nuanced identities of our participants with categorical labels. To do so, we relied on simplified labels including *straight*, *bisexual*, and *gay/lesbian*; we refer to the union of the latter two labels as *queer*. We primarily did this in cases in which our language would potentially be verbose or confusing otherwise (e.g., rather than say, “more cisgender youths than transgender youths showed attraction only to an individual who had a different binary gender”, we opted to say “more cisgender youths than transgender youths were straight”). This means that we sometimes modified the exact term the youths used to describe themselves (e.g., a boy may have self‐identified as “queer” and indicated an exclusive interest in boys, which we would describe as “gay”). While this approach sacrificed some nuance inherent in the youths' self‐report labels, it was necessary for comparing across individuals and time.

Furthermore, the labels of *straight*, *bisexual*, and *gay/lesbian* as we use them in this chapter are agnostic to whether youths show interest in nonbinary people (i.e., a girl may be labeled as “straight” in this monograph even if she showed interest in nonbinary people along with a stated interest in boys), which is not always the case in colloquial use. We did this because we observed several cases in which youth described themselves as straight and also indicated an interest in nonbinary people—demonstrating that at least some youth's identification with being straight did not preclude attraction to nonbinary people. However, we note that this is a limitation of our methodology and an area for future investigation. In this chapter, we examine interest in nonbinary people separately (see section titled *Interest in Nonbinary People*).

Finally, because the meanings of these terms are unclear when used to describe gender‐diverse participants (i.e., a nonbinary person who self‐describes as “gay”), we restrict usage of the labels *straight*, *gay*, and *bisexual* to describing participants who are boys or girls, even though some gender‐diverse youths may use these labels for themselves in everyday life. As a result of this decision, analyses throughout the chapter that refer to *straight*, *gay*, and *bisexual* youths only include participants who were binary transgender or cisgender at the time sexual orientation was measured, though we do include several descriptive and inferential analyses about gender‐diverse youths throughout the chapter.

##### VI.3.1.2 Presentation of Results by Identity‐at‐Visit

In Chapters II‐IV, we primarily presented results broken down by youths' *recruitment group* (*Recruited as Transgender*, *Recruited as Cisgender*, and *Recruited as Siblings*) and sometimes *recruitment gender* (recruited as boy or recruited as girl). In the sections of this chapter that address Question 1 (current sexual orientation) and 2 (relation between childhood gender development and current sexual orientation), we take a different approach by presenting results broken down by youths' gender identities (i.e., boy, girl, or gender diverse) and modalities (i.e., transgender or cisgender) at the time they gave the response being reported. We do so because sexuality terms are often understood in the context of gender (e.g., “lesbian” typically denotes one's gender and sexuality) and because interpreting later gender in terms of initial *recruitment group* could be misleading (e.g., if someone began the study as a transgender girl and now lives as a cisgender boy who is interested in girls, it would be less appropriate to describe him as “lesbian”). Furthermore, we aim to situate our results within an emerging literature on gender‐diverse youths' sexualities that reports sexual orientation as a function of gender (e.g., examining how today's transgender vs. cisgender girls do or not differ in their sexual orientation). Thus, in sections pertaining to Questions 1 and 2, results tables break down responses by youths' identities at the time they gave the response, and we do not report results for youths who did not concurrently give a report of their gender. Alternate versions of tables showing youths' identities (Question 1) broken down by both *recruitment group* (*Recruited as Transgender*, *Recruited as Cisgender*, and *Recruited as Siblings*) and *recruitment gender* (recruited as boy or recruited as girl) are presented in the Supporting Information for this chapter.

Only in addressing Question 3 about stability and change in sexual orientation do we report results with respect to youths' *recruitment gender* and *recruitment group* in the main text; we do this to better mirror equivalent analyses in Chapter IV that report gender change with respect to initial/early gender. Thus, Question 3's analyses clearly indicate people's identities as “recruited as transgender”, “recruited as boys”, etc.

##### VI.3.1.3 Use of Parent Data

In prior chapters, we considered parent reports of youths' gender. In this chapter, we do not examine parent reports of youth sexual orientation for several reasons. First, our parent‐report data on youth sexual orientation were considerably sparser than our youth‐report data. Second, when we did ask parents about their youth's sexual orientations, there were often problems with measure wording (e.g., we almost always asked parents about their predictions of their child's future sexual orientation, rather than their current sexual orientation, making the comparison to youth's answers uninterpretable) and parents often told us they did not know their child's sexual orientation, creating significant missing data. For these reasons, we decided not to code parental responses about youths' sexual orientations and focus exclusively on youth reports throughout this chapter, though we have since adopted clearer measures of parent‐reported youth sexual orientation and hope to report these data in future publications.

### VI.4 Method: Assessment of Sexual Orientation

There were two types of visits in which youths reported data about their sexual orientation. The first type, which we refer to as a “face‐to‐face” visit, was an in‐person or virtual visit where the youths interacted face‐to‐face with an experimenter. These visits (*N* = 490) involved younger children—typically 9–11 (381 visits) but sometimes 12–14 years old (107 visits). At these visits, youths only answered the Childhood Sexuality (Crush—Yes/No) measure (see Table 22) that asked about their previous crushes (“Have you ever had a crush on a [boy]/[girl]?”). Based on this measure alone, youths could be categorized as being attracted to *only boys, both boys and girls*, and *only girls;* youths could also be categorized as not having shown interest in boys or girls.

The second type of visit, which we refer to as an “online survey visit”, was one of several online surveys that youths 12 and over completed without an experimenter (*N* = 1301 visits). In these surveys, youths answered a variety of measures (Teen Sexuality Measures 1–9c, Table 22), which were adjusted throughout the study to be age‐appropriate for the youth as they grew up, as well as to reflect societal shifts in commonly used terms. Youths often answered multiple measures in a single visit.

For each online survey visit, we examined the full set of responses on Teen Sexuality Measures 1–9c that a youth had provided at that visit, and subsequently used qualitative coding on this set of responses to extract information about the youth's sexual orientation on four dimensions: (1) the youths' *Target of Attraction (Among Boys and Girls)*, henceforth referred to as *Target of Attraction*; (2) whether the youth was *Asexual or Aromantic*; (3) the youths' *Interest in Nonbinary People*; and (4) whether the youth was *Questioning*. Two coders—one white genderqueer person, another queer white Hispanic man, both from the United States and in their twenties—independently reviewed all online survey responses and assigned codes to each visit on all applicable dimensions of sexual orientation. All disagreements were resolved through discussion, and inter‐rater reliability was high (see Table 21). Each of these dimensions is described in more detail later in this chapter when we present results (see subsection “Online Survey Visits with Older Youths”); Table 21 presents participant *N*'s and inter‐rater reliability.

**TABLE 21:** Summary of Four Sexual Orientation Dimensions Coded from Online Survey Visits With Teens

Sexual Orientation Dimension	Youths Who Received at Least One Code on Dimension (*N*)	% of Online Survey Visits in Which Code Could Not be Assigned	Inter‐rater Reliability (Cohen's *κ*)
*Target of Attraction*	596	4.3%	0.90
*Asexual*	585	13.1%	0.92
*Interest in Nonbinary People*	542	19.3%	0.92
*Questioning*	595	4.9%	0.86

**TABLE 22:** Summary of Measures Used to Assess Sexual Orientation

Measure	Wording/Description	Number of Responses Per Participant	Age Range Across All Visits (Years)	Range of Years Measure was Asked	Youths With 0 Responses on Measure	Youths With 1 Response on Measure	Youths With 2 or More Responses on Measure
*Childhood Sexuality Measure*: Crush—Yes/No	“Have you ever had a crush on a [boy]/[girl]?” [options for each question: “Yes”, “No”]	Mean = 0.5, range = [0,2]	Mean = 11.2, range = [7, 14]	[2015, 2024]	496	342	74
*Teen Sexuality Measure* 1: Crushes on boys and/or girls	“People sometimes have crushes. Which, if any, best describes your feelings?” [options: “I only have crushes on girls”, “I mostly have crushes on girls”, “I equally have crushes on girls and boys”, “I mostly have crushes on boys”, “I only have crushes on boys”, “Not sure”]	Mean = 0.1, range = [0,1]	Mean = 14.6, range = [13, 18]	[2019, 2020]	806	106	0
*Teen Sexuality Measure 2*: Crushes on binary and/or nonbinary people	“People sometimes have crushes. Which, if any, best describes your feelings?” [options: “I only have crushes on non‐binary people”, “I mostly have crushes on non‐binary people”, “I equally have crushes on non‐binary people and binary people”, “I mostly have crushes on binary people”, “I only have crushes on binary people”, “Not sure”]	Mean = 0, range = [0,1]	Mean = 14.4, range = [13, 18]	[2019, 2020]	874	38	0
*Teen Sexuality Measures 3a, 3b, and 3c*: Attraction to Boys, Girls, and Nonbinary People	“I am romantically interested in [boys]/[girls]/[nonbinary people]” [options for each question: “Yes”, “No”]	Mean = 0.4, range = [0,1]	Mean = 15.2, range = [12, 20]	[2021, 2022]	590	322	0
*Teen Sexuality Measure 4*: LGBQ	“Are you gay, lesbian, bisexual, pansexual, or queer?” [options: “Yes”, “No”]	Mean = 1.3, range = [0,3]	Mean = 15.3, range = [12, 22]	[2021, 2023]	310	211	391
*Teen Sexuality Measure 5*: Select‐all	“How would you describe your sexual orientation?” [options: select all that apply from “Gay”, “Lesbian”, “Bisexual”, “Straight”, “Asexual”, “Pansexual”, “I prefer a different term”]	Mean = 1.2, range = [0,3]	Mean = 15.3, range = [12, 22]	[2021, 2023]	330	214	368
*Teen Sexuality Measure 6*: Open‐ended	“How would you describe your sexuality?” [follow‐up question: “If you have any comments you would like to make about your responses above please feel free to write them here.”]	Mean = 0.8, range = [0,2]	Mean = 15.5, range = [12, 22]	[2022, 2023]	446	239	227
*Teen Sexuality Measures 7a, 7b, and 7c*: General Attraction to Boys, Girls, and Nonbinary People	“I am attracted to [boys/men]/[girls/women]/[nonbinary people]” [options: 1–7 scale, 1 = Not at all true, 7 = Very true]	Mean = 0.7, range = [0,2]	Mean = 15.6, range = [12, 22]	[2022, 2023]	457	239	216
*Teen Sexuality Measures 8a, 8b, and 8c*: Romantic Attraction to Boys, Girls, and Nonbinary People	“I am romantically attracted to [boys/men]/[girls/women]/[nonbinary people]” [options: 1–7 scale, 1 = Not at all true, 7 = Very true]	Mean = 0.7, range = [0,2]	Mean = 15.6, range = [12, 22]	[2022, 2023]	461	247	204
*Teen Sexuality Measures 9a, 9b, and 9c*: Sexual Attraction to Boys, Girls, and Nonbinary People	“I am sexually attracted to [boys/men]/[girls/women]/[nonbinary people]” [options: 1–7 scale, 1 = Not at all true, 7 = Very true]	Mean = 0.5, range = [0,2]	Mean = 16.2, range = [12, 22]	[2022, 2023]	575	263	74

Not all visits included measures on which youths could have provided a response that could be coded for all four dimensions. In other words, some visits (for example) included questions that allowed coders to assign the youth a code for the *Target of Attraction* and *Interest in Nonbinary People* dimensions, but not a code for the *Asexual or Aromantic* or *Questioning* dimensions. Additionally, on some visits, youths skipped all or almost all questions about sexual orientation; we did not consider these visits codable. Therefore, when we report results on each of these four dimensions, we do so with respect to the set of visits on which youths received a set of measures that allowed us to assign them a code on that dimension (and actually answered these measures). In other words, a youth would not be assigned a code on *Asexual* if they received no questions where they could have expressed asexuality (or lack thereof), or if they skipped all the sexual orientation measures on the survey.

### VI.5 Research Question 1: Overall Rates of Sexual Orientation Identification

We present the overall sexual orientation of our participants in two sections: first, we report identities from face‐to‐face visits with younger youths, and second, we report identities from online survey visits with older youths. We present results separately because face‐to‐face visits only included the *Childhood Sexuality Measure* (Crush—Yes/No; see Table 22), whereas online survey visits may have included numerous *Teen Sexuality* measures (Measures 1–9; see Table 22).

#### VI.5.1 Face‐to‐Face Visits with Younger Youths

In face‐to‐face visits, we measured attraction by asking youths if they had or had not had a crush on a boy and/or girl (*Childhood Sexuality Measure*; see Table 22 for full wording). If a youth provided a sexual orientation report on more than one face‐to‐face visit, we present results from their most recent report here. Full results for the 384 youths who answered this measure between ages 7 and 14 are presented in Table 23. For some of these youths, the face‐to‐face visits represented in the table were *not* the most recent occasion on which they reported their attraction because they later reported their attraction on an online survey when they were older (see next section titled “Online Survey Visits with Older Youths”).

**TABLE 23:** *Target of Attraction* as Reported on Most Recent Response to Childhood Sexuality Measure

Identity at Vsit	*N*	Mean Age (Years) at Visit	Youths Reporting a Crush on: *N* (%)			
			Only Boys	Both Boys and Girls	Only Girls	Neither Boys nor Girls
Binary trans boy	50	11.4	4 (8.0%)	8 (16.0%)	23 (46.0%)	15 (30.0%)
Binary trans girl	92	11.3	30 (32.6%)	21 (22.8%)	7 (7.6%)	34 (37.0%)
Cisgender boy	79	11.3	1 (1.3%)	3 (3.8%)	53 (67.1%)	22 (27.8%)
Cisgender girl	129	11.3	69 (53.5%)	20 (15.5%)	0 (0%)	40 (31.0%)
Gender diverse, AFAB	13	11.2	3 (23.1%)	6 (46.2%)	0 (0%)	4 (30.8%)
Gender diverse, AMAB	21	11.0	2 (9.5%)	4 (19.0%)	2 (9.5%)	13 (61.9%)

*Note*: AFAB refers to “assigned female at birth”; AMAB refers to “assigned male at birth.”

##### VI.5.1.1 Overall Rates of Expressing Straight or Queer Romantic Interest (Or No Attraction at All) in Face‐to‐Face Visits with Children

Among transgender boys and girls, 37.3% expressed *straight* attraction, 28.2% expressed *bisexual*, *gay*, or *lesbian* attraction, and 34.5% indicated that they had not yet experienced attraction to boys or girls at their most recent face‐to‐face visit. Among cisgender boys and girls, 58.7% expressed *straight* attraction, 11.5% expressed *bisexual*, *gay*, or *lesbian* attraction, and 29.8% indicated that they had not yet experienced attraction to boys or girls. Few youths were gender diverse at their most recent face‐to‐face visit, and of those who were, 20.6% reported attraction to *only boys* or *only girls*, 29.4% reported attraction to *both boys and girls*, and 50% reported that they had not yet experienced attraction to boys or girls.

##### VI.5.1.2 Does Straight vs. Queer Attraction Differ by Gender and/or Modality on Face‐to‐Face Visits with Children?

We were interested in whether girls (vs. boys) and transgender youths (vs. cisgender youths) were more likely to express *straight* (vs. *queer*) attraction. We considered this question only among boys and girls who expressed attraction to either boys or girls (or both) on a face‐to‐face visit (*N* = 239). We first fit a logistic mixed‐effects regression model predicting whether a youth indicated *straight* (vs. *queer*) attraction from fixed effect predictors of *gender* (boy vs. girl, contrast‐coded −0.5 and 0.5, respectively) and *modality* (transgender vs. cisgender, contrast‐coded −0.5 and 0.5 respectively), their interaction, and a random intercept for each family. This model yielded significant main effects of *gender*, such that girls were less likely to be *straight* than boys (*OR* = 0.35, *z* = −2.41, *p* = .016), and *modality*, such that cisgender youths were more likely to be *straight* than transgender youths (*OR* = 6.12, *z* = 3.23, *p* = .001); there was no significant interaction between *gender* and *modality* (*OR* = 0.48, *z* = −0.88, *p* = .380). We originally intended to test for group differences in expressing *gay*/*lesbian* vs. *bisexual* interest; however, given that there were fewer than 10 cisgender boys who expressed *gay* or *bisexual* interest on a face‐to‐face visit, we did not run this test in keeping with our general rule about minimum cell sizes throughout the monograph.

#### VI.5.2 Online Survey Visits with Older Youths

Here, we report results from online survey visits with older youths regarding the four dimensions of sexual orientation we coded: the youth's *Target of Attraction* among boys and girls, whether the youth was *Asexual*, whether the youth was *Interested in Nonbinary People*, and whether the youth was *Questioning*. For each dimension, if the youth has received a code on the dimension in multiple visits, we report the most recent visit.

##### VI.5.2.1 Target of Attraction (Toward Boys and Girls)

###### VI.5.2.1.1 Coding of Target of Attraction

For each online survey visit (*N* = 1301), human coders reviewed all sexual orientation‐related responses and decided on a code for the youth's *Target of Attraction* at that visit. The youth could be assigned codes indicating that their attraction was to *only boys*, *mostly boys*, *both boys and girls*, *mostly girls*, or *only girls*; participants who indicated interest in neither boys nor girls received a *no interest in boys or girls* code. For some formal analyses that follow in this chapter, there were not enough participants in some categories (in particular, showing interest in *mostly boys* or *mostly girls*) to be able to compute relevant statistics. We therefore converted the five initial categories into three: *only girls*, *both girls and boys*, or *only boys*, such that *mostly girls* and *mostly boys* were subsumed into the *both boys and girls* category. In the Supporting Information for this chapter, we report results of parallel analyses in which *mostly girls* and *mostly boys* are instead considered *only girls* and *only boys*, respectively.

###### VI.5.2.1.2 Rates of Various *Targets of Attraction* on Survey Visits with Older Youths

Among transgender boys and girls who had a relevant response (*N* = 224), 60.3% reported *queer* attraction, 33.0% indicated *straight* attraction, and 6.7% reported interest in *neither boys nor girls*. Among cisgender boys and girls with a relevant report (*N* = 327), 33.0% were *queer*, 62.7% were *straight*, and 4.3% expressed interest in *neither boys nor girls*. Among youths who were gender diverse, 20% showed interest in *neither boys nor girls*, while 71.1% showed interest in *both boys and girls* and 8.9% showed interest in *only girls*. Table 24 shows examples of written‐in responses from youths that correspond with various *Target of Attraction* codes; Table 25 shows full results of the most recent identity.

**TABLE 24:** Example Write‐In Responses for *Target of Attraction* Codes

*Target of Attraction* Code	Example Write‐in Responses
Only boys	“I would describe my sexuality as being attracted to boys”, “I like boys only”, “a cis gay male”
Mostly boys	“I'm attracted to more masculine people rather than fem people”, “Bisexual with a very large preference for men”
Both boys and girls	“pansexual”, “bisexual”
Mostly girls	“bisexual but leaning towards the lesbian side”, “mostly attracted to women but I'm open to other options”
Only girls	“I like girls”, “lesbian”
Not interested in boys or girls	“I'm not interested in other people yet”

**TABLE 25:** Target of Attraction as Indicated on Youths' Most Recent Online Survey Visits

Identity at Visit	*N*	Mean Age at Visit (years)	Youths Indicating Attraction to: *N* (%)					
			Only Boys	Mostly Boys	Both Boys and Girls	Mostly Girls	Only Girls	Neither Boys nor Girls
Binary trans boy	80	16.1	10 (12.5%)	9 (11.2%)	23 (28.8%)	8 (10.0%)	28 (35.0%)	2 (2.5%)
Binary trans girl	144	15.5	46 (31.9%)	10 (6.9%)	36 (25.0%)	13 (9.0%)	26 (18.1%)	13 (9.0%)
Cisgender boy	145	15.6	5 (3.4%)	3 (2.1%)	10 (6.9%)	9 (6.2%)	111 (76.6%)	7 (4.8%)
Cisgender girl	182	15.5	94 (51.6%)	22 (12.1%)	39 (21.4%)	7 (3.8%)	13 (7.1%)	7 (3.8%)
Gender diverse, AFAB	30	15.8	0 (0%)	0 (0%)	19 (63.3%)	2 (6.7%)	3 (10.0%)	6 (20.0%)
Gender diverse, AMAB	15	15.4	0 (0%)	0 (0%)	10 (66.7%)	1 (6.7%)	1 (6.7%)	3 (20.0%)

*Note*: AFAB refers to “assigned female at birth”; AMAB refers to “assigned male at birth.”

###### VI.5.2.1.3 Testing Differences in Target of Attraction by Gender and Modality

A series of analyses probed whether there were group differences in attraction.

###### VI.5.2.1.4 Are Boys vs. Girls, or Transgender vs. Cisgender Youths, More Likely to Report Queer Attraction?

We assessed whether there were *gender* or *modality* differences in identifying as *straight* vs. *queer*. As with the face‐to‐face visits, we tested this only among girls and boys (excluding youths who were gender diverse at their most recent visit due to small sample sizes and interpretational concerns). A logistic mixed‐effects model including fixed‐effect categorical predictors of *gender* (boy vs. girl; contrast‐coded −0.5 and 0.5, respectively), *modality* (transgender vs. cisgender; contrast‐coded −0.5 and 0.5, respectively), and the interaction of *gender* and *modality*, as well as a random intercept for each family, found that girls were less likely than boys to be *straight* (*OR* = 0.52, *z* = −3.21, *p* = .001) and that cisgender youths were more likely than transgender youths to be *straight* (*OR* = 4.13, *z* = 6.30, *p* < .001)—mirroring patterns found in the younger subset of youths. There was also a significant interaction between *gender* and *modality* (a pattern we did not find with younger youths), such that the difference between boys and girls was more pronounced for cisgender youths than it was for transgender youths (*OR* = 0.28, *z* = −3.02, *p* = .003).

###### VI.5.2.1.5 Are Some Groups of Queer Youths (e.g., boys, trans youths) More Likely to be Bisexual vs. Gay/Lesbian?

As a second question, we looked more closely at *queer* youths (*N* = 243) to determine whether there were group differences in whether queer youths were *gay* vs. *bisexual*. Our original logistic mixed‐effects model with a random intercept for each model obtained a singular fit, so we removed the random intercept and ran a simple logistic regression model predicting whether youths were gay or bisexual from predictors of *gender*, *modality*, and their interaction. This model found that none were significant predictors (all *p*'s > .196). That is, among queer youths, there were no differences by *gender* or *modality* in rates of being *bisexual* vs. interested in one's own gender exclusively.

###### VI.5.2.1.6 Do Binary Transgender, Cisgender, and Gender‐Diverse Youths Differ in Rates of Being Bisexual (Rather than Attracted to One Gender)?

As a final analysis, we considered whether there were between‐modality differences in likelihood to report single‐gender attraction (i.e., *only boys* or *only girls*) vs. *bisexual* attraction (i.e., *mostly boys*, *both boys and girls*, and *mostly girls*). A 3 (*modality*: transgender, cisgender, gender diverse) x 2 (single‐gender attraction, bisexual) χ^2^ test of independence showed that youths in different modality groups differed in their likelihood of being bisexual, χ^2^(2) = 57.23, *p* < .001; follow‐up tests found that gender‐diverse youths were more likely to be bisexual than binary transgender (χ^2^(1) = 19.64, *p* < .001) and cisgender (χ^2^(1) = 48.74, *p* < .001) youths.

###### VI.5.2.1.7 Interim Summary: Target of Attraction

We found that girls were more likely than boys, and binary transgender youths more likely than cisgender youths, to express queer attraction, though gender identity and modality were not related to which variety of queer attraction (i.e., gay or lesbian vs. bisexual) one reported. Gender‐diverse participants overwhelmingly tended to report interest in *both boys and girls* and did so to a greater extent than binary boys and girls.

##### VI.5.2.2 Other Dimensions of Sexuality: Asexuality/Aromanticism, Interest in Nonbinary People, and Questioning

In addition to classifying youths' *Target of Attraction* on each online survey visit, coders also determined whether youths (1) were asexual or aromantic, (2) showed attraction to nonbinary people, or (3) expressed that they were questioning or otherwise unsure of their sexuality (henceforth referred to as *Asexual*, *Interest in Nonbinary*, and *Questioning*, respectively). For all three of these dimensions, coders assigned a code of *Yes* or *No* based on the youths' responses on online survey visits when relevant information was available.

For each dimension, we had three key questions. First, were youths more likely to have a *Yes* on any of these codes depending on their modality (binary transgender, cisgender, or gender diverse)? To test this, we fit a logistic regression model predicting *Yes* codes with a 3‐level categorical predictor of gender modality. Second, were youths more likely to have a *Yes* on any of these codes depending on their gender identity (boy, girl, or gender diverse)? To test this, we fit a logistic regression model predicting *Yes* codes with a 3‐level categorical predictor of gender identity. Regression models including predictors of gender identity, gender modality, and their interaction (as we conducted in the *Target of Attraction* analyses) were not appropriate here because gender diverse is both an identity group and a modality group in our analyses (given that some nonbinary people see that identity as a gender identity and a gender modality). Thus, we opted to fit separate models to examine the effects (or lack thereof) of identity and modality individually.

Third and finally, were youths who reported different *Targets of Attraction* (interest in boys and/or girls, as described in the prior section) more likely to be coded as *Asexual*, *Interested in Nonbinary People*, or *Questioning*? To test this question, we fit a logistic regression model predicting whether or not a youth received *Yes* codes from a categorical predictor of *Target of Attraction*, excluding youths who reported no *Target of Attraction*.

We initially attempted to fit all of the following models in this section with random intercepts for each family to account for nonindependence introduced by sibling pairs in the data set; when these models led to singular fits or convergence issues, we removed the random intercepts. When we were able to include random intercepts, we determined the significance of categorical predictors with three or more levels (e.g., boy, girl, gender diverse) using a Wald χ^2^ test; in cases in which we were not able to do include random intercepts, we report a likelihood ratio χ^2^ statistic.

###### VI.5.2.2.1 Asexuality and Aromanticism

To receive a *Yes* code on *Asexual*, youths had to endorse or write in the labels “asexual” or “aromantic” (or a related term, e.g., “greysexual”); coders determined that never having experienced attraction (e.g., saying “I'm 12 and I haven't had a crush on anyone”) was not sufficient evidence of asexuality or aromanticism and received a *No* code. This decision was made because we typically do not consider young children who have not yet experienced attraction as “asexual”—at least in the same way—as we do individuals who express this identity later in life. Additionally, being coded as *Yes* on *Asexual or Aromantic* was not considered mutually exclusive with showing some attraction to others, because we found that many youths expressed identities such as “asexual lesbian” (which we coded as *Yes* on *Asexual* and *only girls* on *Target of Attraction*). Full results are presented in Table 26.

**TABLE 26:** Most Recent Youth Self‐Identification as *Asexual* on Online Survey Visit

Identity at Visit	Total *N* Receiving Code on *Asexual*	Mean Age (Years)	*Asexual* (% of Total)	*Target of Attraction* Among Youths Coded as *Asexual*: *N*					
				Only Boys	Mostly Boys	Both Boys and Girls	Mostly Girls	Only Girls	Not Interested in Boys or Girls
Binary trans boy	79	16.1	8 (10.1%)	1	1	3	0	1	2
Binary trans girl	139	15.5	14 (10.1%)	0	0	1	2	2	9
Cisgender boy	142	15.6	6 (4.2%)	1	0	0	0	1	4
Cisgender girl	177	15.5	11 (6.2%)	2	3	2	0	1	3
Gender diverse, AFAB	30	15.8	11 (36.7%)	0	0	6	0	1	4
Gender diverse, AMAB	15	15.4	3 (20.0%)	0	0	1	0	0	2

*Note*: Includes only youths who participated in online survey visits in which they could have been coded as *Asexual* (i.e., completed a relevant measure; *N* = 585).

###### VI.5.2.2.2 Relation Between Asexuality and Gender/Modality

Both gender modality (χ^2^(2) = 23.84, *p* < .001) and identity (χ^2^(2) = 20.06, *p* < .001) were significant predictors of asexuality. With respect to gender modality, post‐hoc pairwise Tukey tests found that gender‐diverse youths were more likely to be *Asexual* than transgender youths (*z* = 3.55, *p* = .001) and cisgender youths (*z* = −5.11, *p* < .001), but transgender and cisgender youths did not differ (*z* = −2.06, *p* = .099). With respect to gender identity, post‐hoc pairwise Tukey tests found that gender‐diverse youths were more likely to be *Asexual* than girls (*z* = 4.33, *p* < .001) and boys (*z* = −4.48, *p* < .001), but boys and girls did not differ (*z* = −0.69, *p* = .770).

###### VI.5.2.2.3 Relation Between Asexuality and Target of Attraction

**Figure 14:**
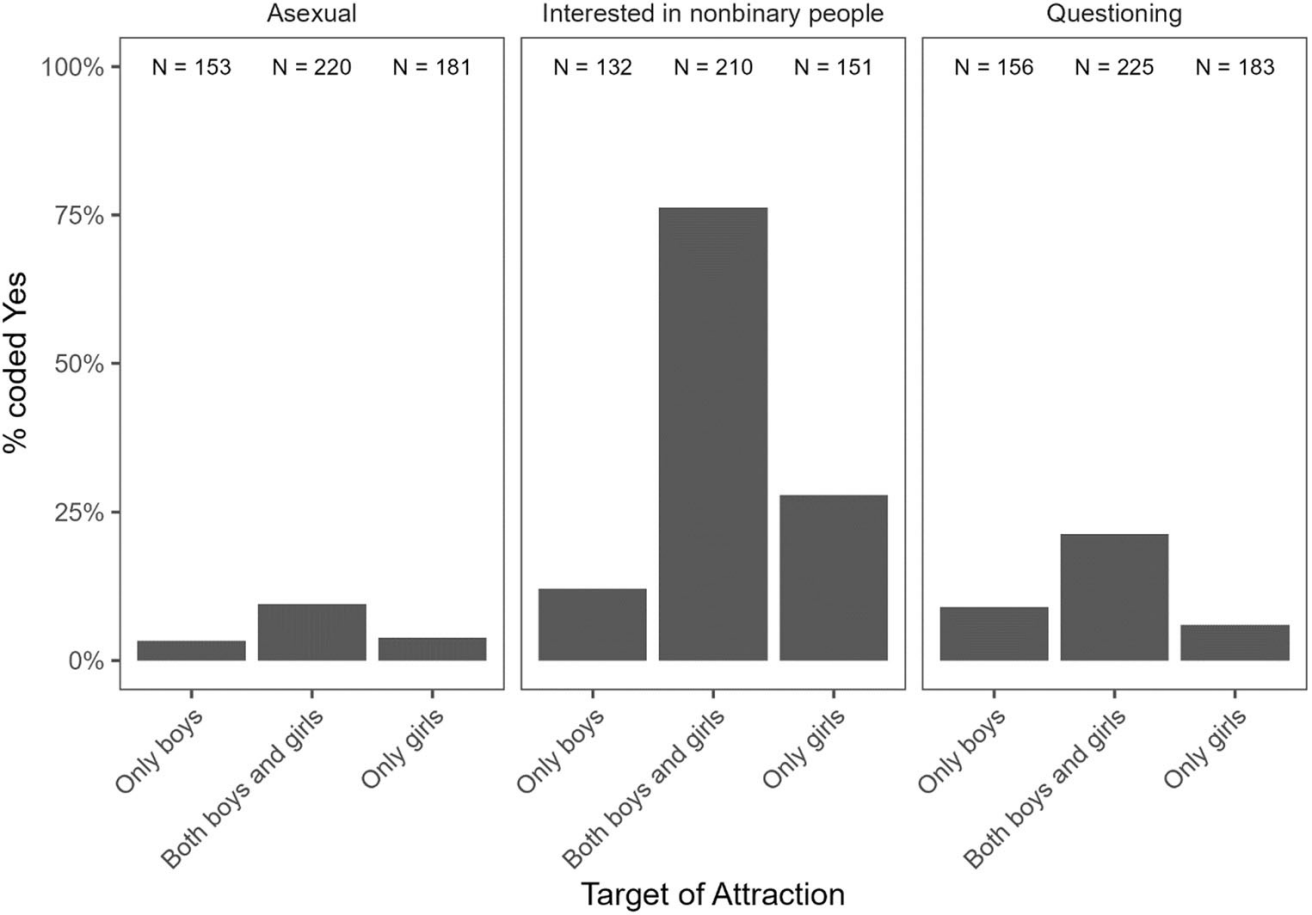
Relationship between target of romantic attraction and asexual, interest in nonbinary people, and questioning.

As shown in the leftmost panel of Figure 14, we also found that *Target of Attraction* was related to being *Asexual* (χ^2^(2) = 8.62, p = *.010*); however, no post‐hoc pairwise Tukey comparisons between *Target of Attraction* categories (*only boys*, *both boys and girls*, and *only girls*) were significant (all *p*'s > .06). The overall number of youths who were *Asexual* was small; thus, the model was likely underpowered to detect subtle differences between *Target of Attraction* categories in asexuality.

###### VI.5.2.2.4 Interest in Nonbinary People

Participants received a “Yes” code on *Interest in Nonbinary* if they expressed interest in nonbinary or genderqueer people in their responses to any of Teen Sexuality Measures 1–9c. Full results on this dimension are presented in Table 27.

**TABLE 27:** Most Recently Expressed Interest or Non‐Interest in Nonbinary People Among Youths

Identity at Visit	Mean Age at Visit (years)	*N*	*N* (% of Total)	
			*Yes* on *Interest in Nonbinary*	*No* on *Interest in Nonbinary*
Binary trans boy	16.2	76	35 (46.1%)	41 (53.9%)
Binary trans girl	15.5	126	65 (51.6%)	61 (48.4%)
Cisgender boy	15.6	132	31 (23.5%)	101 (76.5%)
Cisgender girl	15.6	167	57 (34.1%)	110 (65.9%)
Gender diverse, AFAB	15.9	27	22 (81.5%)	5 (18.5%)
Gender diverse, AMAB	15.5	13	12 (92.3%)	1 (7.7%)

###### VI.5.2.2.5 Relation Between Interest in Nonbinary People and Gender/Modality

Both gender modality, Wald χ^2^(2) = 39.23, *p* < .001, and identity, Wald χ^2^(2) = 27.50, *p* < .001, were significant predictors of interest in nonbinary people. With respect to gender modality, post‐hoc pairwise Tukey tests found that gender‐diverse youths were more likely to be interested in nonbinary people than transgender youths (*z* = 3.55, *p* = .001) and cisgender youths (*z* = −5.43, *p* < .001); transgender youths were also more likely to be interested in nonbinary people than cisgender youths (*z* = −4.37, *p* < .001). With respect to gender identity, post‐hoc pairwise Tukey tests found that gender‐diverse youths were more likely to be interested in nonbinary people than girls (*z* = 4.43, *p* < .001) and boys (*z* = −5.18, *p* < .001), but boys and girls did not differ (*z* = −2.23, *p* = .066).

###### VI.5.2.2.6 Relation Between Interest in Nonbinary People and Target of Attraction

As illustrated in the center panel of Figure 14, *Target of Attraction* was related to interest in nonbinary people, Wald χ^2^(2) = 46.08, *p* < .001. Post‐hoc pairwise Tukey comparisons showed that youths interested in *both boys and girls* were more likely to be interested in nonbinary people than those whose interest was in *only boys* (*z* = 6.75, *p* < .001) or *only girls* (*z* = 5.57, *p* < .001); youths interested in *only girls* were also more likely to be interested in nonbinary people than youths interested in *only boys* (*z* = −3.48, *p* = .002).

###### VI.5.2.2.7 Questioning

Participants were coded as *Yes* if they indicated that they were currently unsure or confused about any aspect of their sexuality. Lack of interest in boys or girls was not seen as evidence of *Questioning* (i.e., simply saying “I'm not into guys or girls” did not indicate questioning, to help differentiate this categorization from asexuality or aromanticism). Full descriptive results are presented in Table 28.

**TABLE 28:** Most Recently Expressed Report of Questioning Among Youths

Identity at Visit	Mean Age at Visit (Years)	*N*	*N* (% of Total)	
			*Questioning*	Not *Questioning*
Binary trans boy	16.1	79	15 (19.0%)	64 (81.0%)
Binary trans girl	15.4	144	19 (13.2%)	125 (86.8%)
Cisgender boy	15.6	145	9 (6.2%)	136 (93.8%)
Cisgender girl	15.5	181	36 (19.9%)	145 (80.1%)
Gender diverse, AFAB	15.8	30	5 (16.7%)	25 (83.3%)
Gender diverse, AMAB	15.4	15	3 (20.0%)	12 (80.0%)

###### VI.5.2.2.8 Relation Between Questioning and Gender/Modality

There was no significant relation between *Questioning* and gender modality (χ^2^(2) = 0.59, *p* = .746) or gender identity (χ^2^(2) = 4.64, *p* = .098).

###### VI.5.2.2.9 Relation Between Questioning and Target of Attraction

As illustrated in the rightmost panel of Figure 14, *Target of Attraction* was related to questioning one's sexuality (χ^2^(2) = 27.18, *p* < .001). Post‐hoc pairwise Tukey comparisons showed that youths whose interest was in *both boys and girls* were more likely to be questioning than those whose interest was in *only boys* (*z* = 3.33, *p* = .003) or *only girls* (*z* = 4.29, *p* < .001). Youths interested in *only girls* did not differ from youths interested in *only boys* (*z* = 1.05, *p* = .549).

###### VI.5.2.2.10 Interim Summary: Asexuality/Aromanticism, Interest in Nonbinary People, and Questioning

Gender‐diverse youths showed the highest identification as *Asexual*—higher than boys and girls (who did not differ from each other) and binary transgender and cisgender youths (who did not differ from each other). We found no significant relation between *Asexual* and *Target of Attraction*, perhaps because of small sample sizes. Gender‐diverse youths showed more *Interest in Nonbinary People* than binary transgender youths, who in turned showed more *Interest in Nonbinary People* than cisgender youths; gender‐diverse youths also were more interested in nonbinary people than boys or girls (who did not differ from each other). Finally, likelihood of questioning one's sexual orientation did not differ as a function of gender modality (binary transgender, cisgender, or gender diverse) or identity (boy, girl, or gender diverse), though youths whose *Target of Attraction* was *both boys and girls* were more likely to be *Questioning* relative to those who were interested in *only boys* or *only girls*.

### VI.6 Research Question 2: What is the Relation Between Earliest Gender Development Measures and Current Sexual Orientation?

Our questions of interest in this section concern whether youths' or parents' earliest reports on three gender development measures—(a) their gender identity on a continuous spectrum (*Continuum*), (b) their gender‐typed preferences for peers, toys, and clothing (*Preference*), and (c) their parents' reports of their gendered preferences and identity (*Parent Questionnaire*, Johnson et al., 2004)—predict youths' current attraction. More information about these measures can be found in the Supporting Information for Chapters III (*Continuum*) and V (*Preference* and *Parent Questionnaire*).

#### VI.6.1 Analytic Strategy and Participant Inclusion

For each of the three gender development measures we use in this section, we ask two questions, in two separate models for each gender development measure: (1) Among current boys, are those who gave more feminine scores on the childhood measures more likely to be gay or bisexual today? (2) Among current girls, are those who gave more masculine scores on the childhood measures more likely to be lesbian or bisexual today?

In addition, for both current boys and current girls, we ask whether the relation between earliest scores and current *Target of Attraction* depends on *current modality* (i.e., whether the youth is transgender or cisgender). Thus, for each gender development measure, we test whether earliest scores are associated with current *Target of Attraction*, *current modality* (binary transgender or cisgender), and the interaction between these two factors. If youths (or parents, in the case of the *Parent Questionnaire*) completed a given gender development measure multiple times, we used the earliest report of the measure.

##### VI.6.1.1 Statistical Analysis

**Figure 15:**
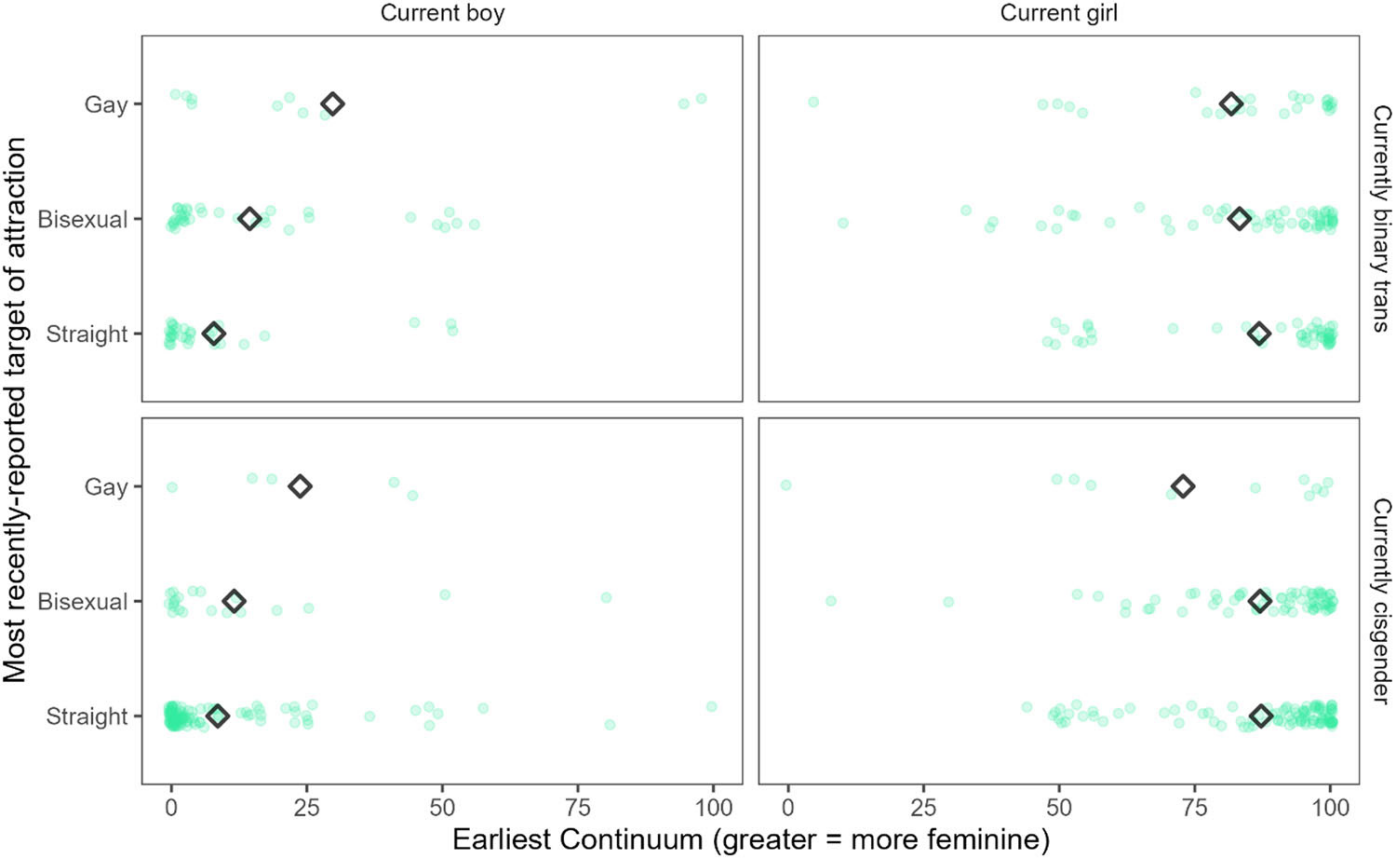
Earliest continuum scores broken down by target of attraction, current gender, and current modality. *Note*: Includes only youths who are currently boys or girls (not gender diverse); “Gay” as it applies to current girls is equivalent to “Lesbian.” Black diamonds represent group means. Points are jittered for visual clarity.

**Figure 16:**
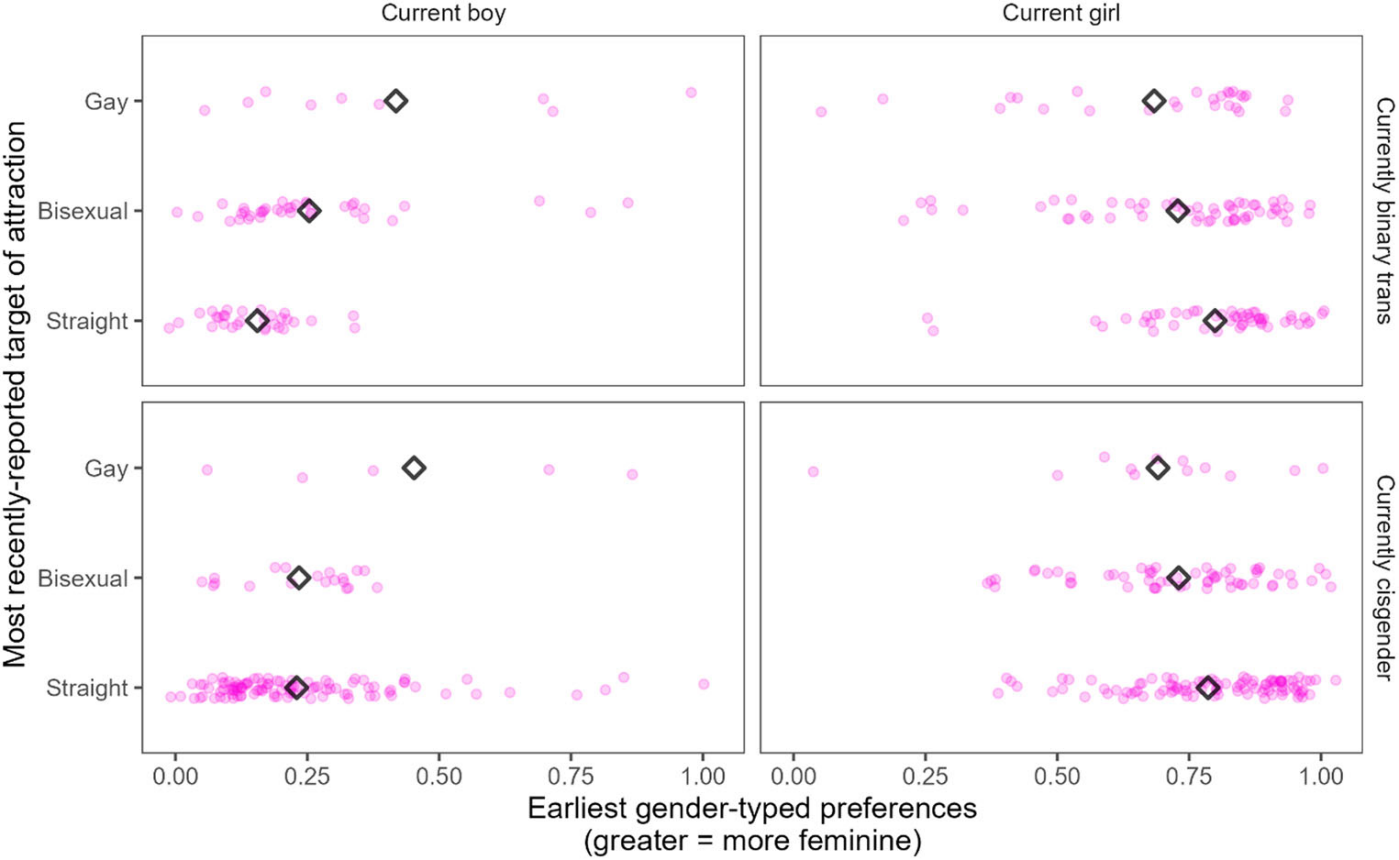
Earliest preference scores broken down by target of attraction, current gender, and current modality. *Note*: Includes only youths who were most recently boys or girls (not gender diverse); “Gay” as it applies to current girls is equivalent to “Lesbian.” Black diamonds represent group means. Points are jittered for visual clarity.

**Figure 17:**
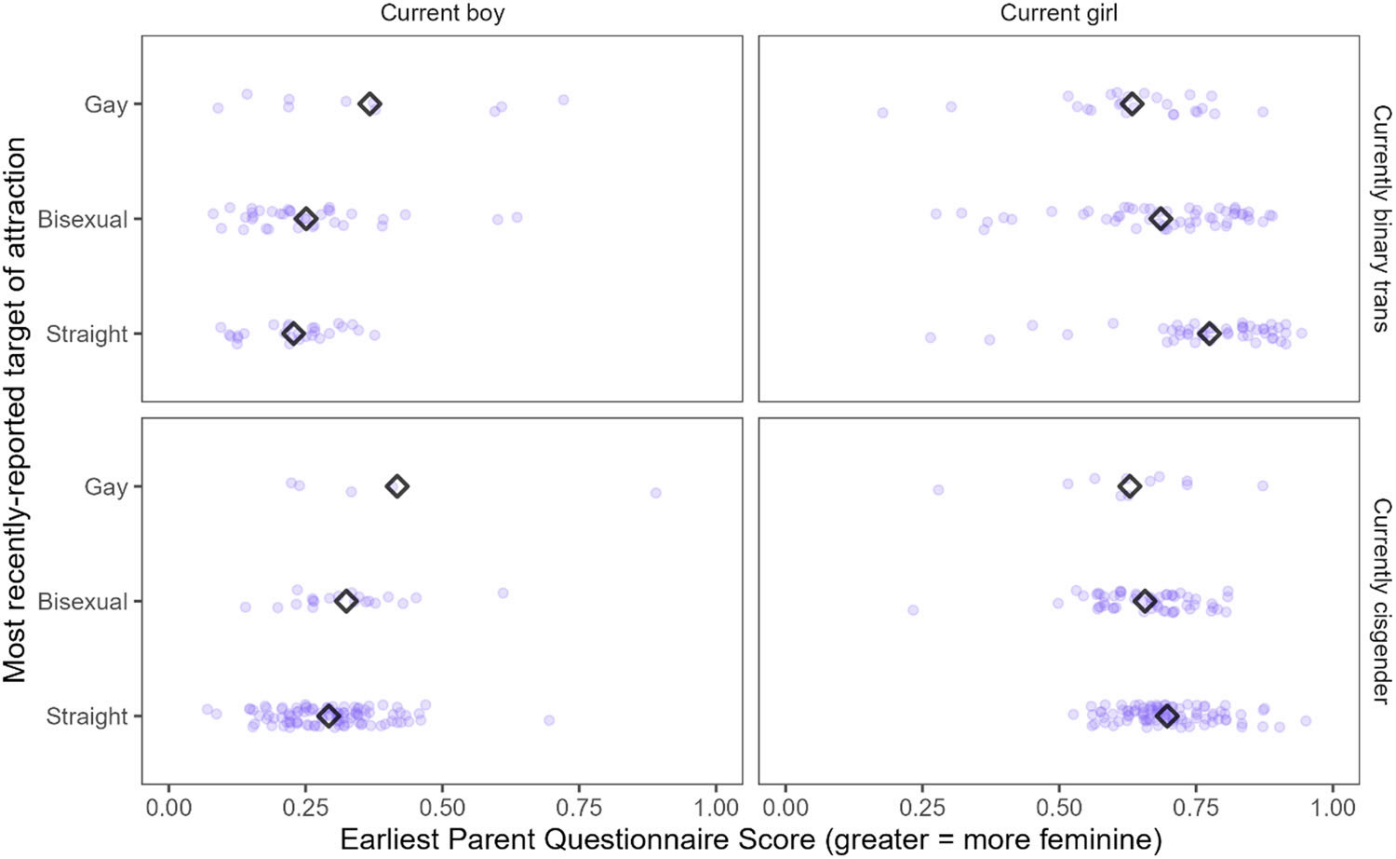
Earliest parent questionnaire scores broken down by target of attraction, current gender, and current modality. *Note*: Includes only youths who were most recently boys or girls (not gender diverse); “Gay” as it applies to current girls is equivalent to “Lesbian.” Black diamonds represent group means. Points are jittered for visual clarity.

To maintain consistency with other analyses in this monograph, we do not run models with cells that have 10 or fewer participants. This issue arose in all analyses in this section that were focused on boys because there were very few currently cisgender boys who indicated interest in *only boys*. To deal with this issue in these boy‐specific analyses, we further collapsed the 3‐level *Target of Attraction* variable (interest in *only boys*, *both boys and girls*, and *only girls*) into a 2‐level variable: *queer* (i.e., interest in *only boys* or *both boys and girls*) and *straight* (interest in *only girls*). Thus, all of the boy‐specific analyses in this section tested whether earliest predictor scores are associated with two 2‐level contrast‐coded factors (current attraction: *queer* or *straight*, coded −0.5 and 0.5, respectively; *current modality*: transgender or cisgender, coded −0.5 and 0.5, respectively) and their interaction—though in visualizations (Figures 15, 16, and 17), we show queer boys' scores broken down into the subcategories of *gay* and *bisexual*. For boy‐specific analyses, we performed linear regressions on these contrast‐coded predictors, and we report regression coefficients and their significance. Conversely, because they did not encounter issues with small *N*'s, girl‐specific analyses included models with the original three‐level categorical factor of current *Target of Attraction* (*only boys, both boys and girls*, or *only girls*), a two‐level factor of *current modality* (transgender or cisgender), and their interaction.

For all following models, we first attempted to include a random intercept for each family to account for sibling pairs in the dataset. If these models obtained singular fits or did not converge, we fit simple linear models instead. In all girl‐specific models that include the 3‐level categorical factor of *Target of Attraction*, we conduct type‐III analysis of variance with sum‐contrast‐coded predictors and evaluate significance of Wald χ^2^ or *F* statistics in models with and without random‐effects specifications, respectively. In the case of significant χ^2^ or omnibus *F* statistics, we follow up with post‐hoc Tukey adjusted pairwise comparisons on estimated marginal means. A summary of significant results from these models is presented in Table 31 later this chapter.

#### VI.6.2 Relation Between Earliest *Continuum* and Later *Target of Attraction*

A brief description of *Continuum* can be found in Chapter III, and more detailed information about the measure can be found in the Supporting Information for Chapter III. For our purposes here, the key point for readers to know is that youths indicated their identity on a continuous scale from 0 (feeling totally like a boy) to 100 (feeling totally like a girl). Youths in these analyses had a mean age of 10.6 years at earliest *Continuum* report and 15.3 years at most recent *Target of Attraction* report, allowing for an average prediction span of 4.7 years.

##### VI.6.2.1 Current Boys and Continuum

Among current boys (*N* = 208; see Figure 15 and Table 29), we found a significant effect of *Target of Attraction* such that *straight* boys showed significantly more masculine earliest *Continuum* scores than *queer* boys (*b* = −7.74, *p* = .011); there was no significant effect of *current modality* (i.e., cisgender and transgender boys did not differ in their scores; *b* = 1.56, *p* = .604) and there was no significant interaction between *current modality* and *Target of Attraction* (*b* = 4.55, *p* = .449).

**TABLE 29:** Means, Standard Deviations, and *N*'s of Current Boys by Modality and Most Recent Attraction

Modality	Attraction	Continuum			Preference			Parent Questionnaire		
		M	SD	*N*	M	SD	*N*	M	SD	*N*
Transgender	Queer	17.9	24	45	0.29	0.23	46	0.28	0.15	46
	Straight	7.8	14.6	31	0.16	0.08	30	0.23	0.08	27
Cisgender	Queer	14	20.1	25	0.28	0.19	24	0.34	0.15	25
	Straight	8.6	16.7	107	0.23	0.18	104	0.29	0.09	99

*Note*: Lower values on all three measures indicate more stereotypically masculine responding.

##### VI.6.2.2 Current Girls and Continuum

Among current girls (*N *= 308, see Figure 15 and Table 30), *Target of Attraction* was significantly related to earliest *Continuum* score, *F*(2, 302) = 3.29, *p* = .039; post‐hoc Tukey tests showed that *straight* girls showed significantly more feminine scores than *lesbian* girls, *t*(302) = 2.57, *p* = .029, but no other pairwise comparisons were significant (all *p*'s > .102). There was no significant effect of *current modality, F*(1, 302) = 0.32, *p* = .572, and no significant interaction between *Target of Attraction* and *current modality, F*(2, 302) = 1.39, *p* = .251.

**TABLE 30:** Means, Standard Deviations, and *N*'s of Current Girls by Modality and Most Recent Attraction

Modality	Attraction	Continuum			Preference			Parent Questionnaire		
		*M*	*SD*	*N*	M	*SD*	*N*	*M*	*SD*	*N*
Transgender	Straight	86.9	18.4	49	0.8	0.16	50	0.77	0.14	48
	Bisexual	83.3	21.2	58	0.73	0.2	55	0.69	0.15	53
	Lesbian	81.7	23	26	0.68	0.23	26	0.63	0.15	25
Cisgender	Straight	87.3	16.3	98	0.79	0.15	98	0.7	0.08	96
	Bisexual	87	17.1	66	0.73	0.16	59	0.66	0.1	57
	Lesbian	72.9	31.3	11	0.69	0.25	12	0.63	0.15	11

**TABLE 31:** Summary of Relations Between Continuous Gender Development Measures in Childhood and Sexual Orientation in Adolescence

Measure	Brief Description of Measure	Result With Current Boys	Result With Current Girls
*Continuum*	Youth‐reported continuous gender identity on a 0–100 scale	Straight boys more masculine than queer boys; no effect of, or interaction with, modality	Straight girls more feminine than lesbian, no other pairwise significant; no effect of, or interaction with, modality
*Preference*	Youth‐reported gender‐typed preferences for peers, toys, and clothing	Straight boys more masculine than queer boys; no effect of, or interaction with, modality	Straight girls more feminine than both lesbian and bisexual girls, who did not differ; no effect of, or interaction with, modality
*Parent questionnaire*	Parent‐reported gender typicality	Straight boys more masculine than queer boys; transgender boys more masculine than cisgender boys; no interaction between sexual orientation and modality	Straight girls more feminine than both lesbian and bisexual girls, who did not differ; transgender more feminine than cisgender; no interaction between sexual orientation and modality

#### VI.6.3 Relation Between Earliest Gender‐Typed Preferences and Current Attraction

A full description of the *Preference* composite measure (assessing peer, toy, and clothing preferences) can be found in the Supporting Information for Chapter V, but for our purposes in this chapter, the key point for readers to know is that youths received a composite score ranging from 0 (most masculine preferences) to 1 (most feminine preferences). Youths in these analyses had a mean age of 8.2 years at earliest *Preference* report and 14.9 years at most recent *Target of Attraction* report, allowing for an average prediction span of 6.7 years.

##### VI.6.3.1 Current Boys and Preferences

Among current boys (*N* = 204, see Figure 16 and Table 29), we found a significant effect of *Target of Attraction* such that current boys who are *straight* showed significantly more masculine *Preference* scores than those who are *queer* (*b* = −.09, *p* = .003); there was no significant effect of *current modality* (*b* = −.03, *p* = .251) and there was no significant interaction between *current modality* and *Target of Attraction* (*b* = −.08, *p* = .176).

##### VI.6.3.2 Current Girls and Preferences

For current girls (*N* = 300, see Figure 16 and Table 30), a type‐III analysis of variance found that *Target of Attraction* was significantly related to earliest *Preference* score, *F*(2, 294) = 6.79, *p* < .001. Post‐hoc pairwise Tukey tests comparing levels of *Target of Attraction* found that *straight* girls showed more feminine *Preference* scores than those who were *bisexual, t*(294) = 2.84, *p* = .014 and *lesbian, t*(294) = 3.10, *p* = .006; *bisexual* and *lesbian* girls did not significantly differ, *t*(294) = 1.22, *p* = .440. There was no significant effect of *current modality, F*(1, 294) = 0.03, *p* = .953; there was also no interaction between the two predictors, *F*(2, 294) = 0.08, *p* = .925.

#### VI.6.4 Relation Between Earliest *Parent Questionnaire* and Current Attraction

A full description of the *Parent Questionnaire* can be found in the Supporting Information for Chapter V; for our purposes in this chapter, the key point for readers to know is that parents answered an 18‐item measure assessing the gender (non)conformity of their child's preferences, identity, and desire to be the other binary gender. Youths in these analyses had a mean age of 9.0 years at earliest *Parent Questionnaire* report and 15.0 years at most recent *Target of Attraction* report, allowing for an average prediction span of 6.0 years.

##### VI.6.4.1 Current Boys and Parent Questionnaire

Among current boys (*N *= 197, see Figure 17 and Table 29), we found a significant effect of *Target of Attraction* such that current boys who are *straight* showed significantly more masculine scores than those who are *queer* (*b* = −.04, *p* = .021); further, there was a significant effect of *current modality* such that transgender boys showed slightly more masculine scores than cisgender boys (*b* = .05, *p* = .002). There was no significant interaction between *current modality* and *Target of Attraction* (*b* = .03, *p* = .372).

##### VI.6.4.2 Current Girls and Parent Questionnaire

Among current girls (*N* = 290, see Figure 17 and Table 30), *Target of Attraction,* Wald χ^2^(2) = 32.89, *p* < .001 and *current modality,* Wald χ^2^(2) = 21.25, *p* < .001, were significantly related to earliest *Parent Questionnaire* scores. Post‐hoc Tukey‐adjusted pairwise comparisons on estimated marginal means of *Target of Attraction* groups showed that *straight* girls had significantly more feminine scores than *bisexual* girls (*t*(172) = 4.15, *p* < .001) (and *lesbian* girls (*t*(141) = 4.70, *p* < .001), though *bisexual* and *lesbian* girls did not differ, *t*(177) = 1.89, *p* = .144. Further, binary transgender girls had significantly more feminine scores than cisgender girls, *t*(85.8) = 2.82, *p* = .006. There was no significant interaction between *Target of Attraction* and *current modality,* Wald χ^2^(2) = 5.27, *p* = .072.

### VI.7 Research Question 3: Stability and Change in Attraction Over Time

For youths who gave more than one report of attraction, how much stability and change did they show across reports? To answer this question, we examined youths' responses on both face‐to‐face and online survey visits. We defined change as a sequence of two self‐reports: in the first, the youth must have expressed being interested in boys, girls, or both; in the second, they must have expressed a different *Target of Attraction* or stated that they were interested in neither boys nor girls. Thus, for example, a youth who expressed interest in girls at age 11 and then expressed they had no romantic or sexual interest at age 13 would qualify as a change for the above table. We did not, however, consider a youth who had expressed no interest at 11 and then interest in girls at 13 to have shown change for the following analyses. The reason for this asymmetry is that attraction develops over time and often emerges in adolescence. As reports of being interested in neither boys nor girls on childhood face‐to‐face visits were common,O and several young participants indicated (without being explicitly prompted to do so) that they were too young to have attraction, we decided that developing an interest (after having no such interest at age 9) is not what we typically mean when we say that someone's attraction changed.

**Figure 18:**
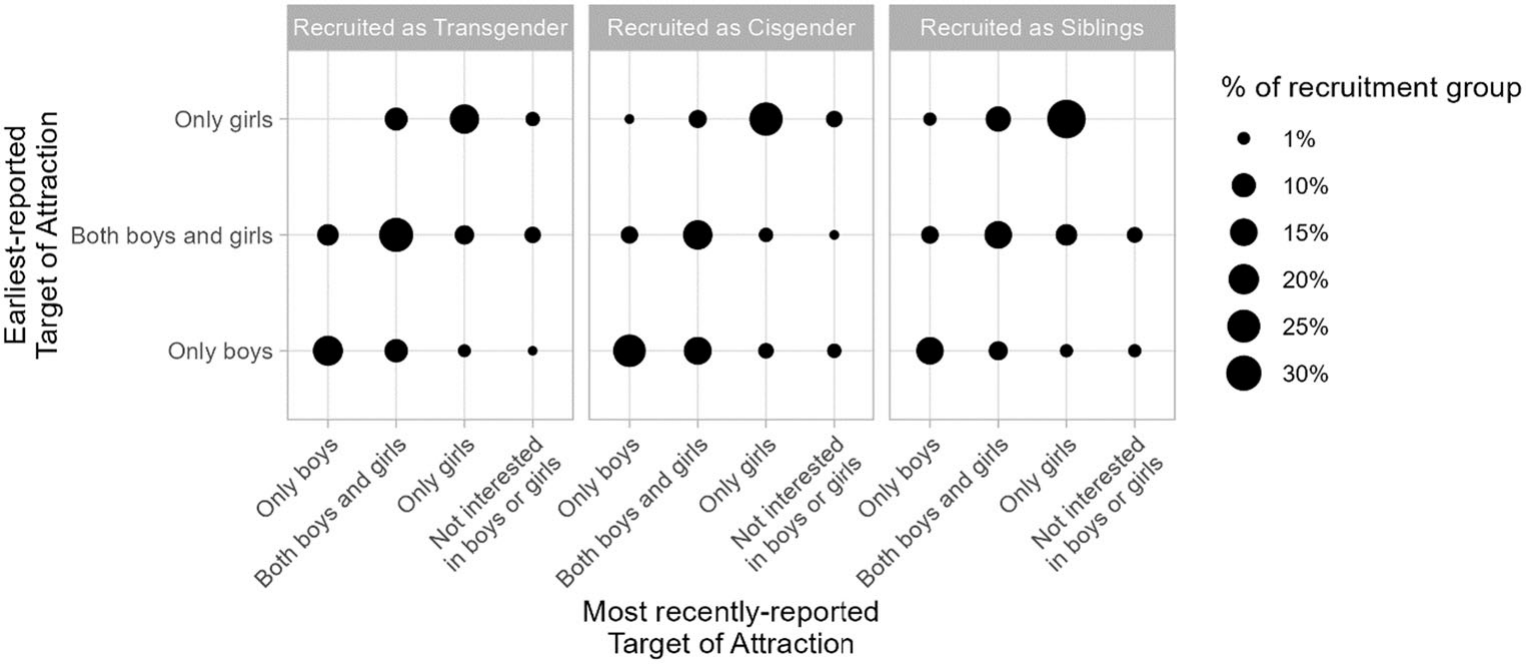
Relationship between earliest and most recent report of attraction.

Among the full sample of 912 youths, 460 (50.4%) have reported attraction enough times to be able to be included in analyses of stability and change. In general, a majority of these 460 participants showed stability (61.5%) but a substantial minority (38.5%) showed change. Overall levels of stability and change are summarized in Table 32. Frequencies of specific trajectories of change in *Target of Attraction* are shown in Table 33, which demonstrates that when change did occur, it mostly involved changing to or from an interest in *both boys and girls*. Longitudinal movement (earliest report to latest report) between different targets of attraction is visualized in Figure 18.

**TABLE 32:** Stability and Change in Attraction by Recruitment Group and Recruitment Gender

Recruitment Group	Recruitment Gender	Mean Age (years) at Earliest Attraction Report (SD)	Mean Years Between First and Latest Attraction Reports (SD)	*N*	% Who Change
Recruited as Transgender	Boy	13.0 (1.9)	3.4 (2)	67	34.3%
	Girl	12.7 (2)	2.9 (1.6)	120	45.0%
Recruited as Cisgender	Boy	13.5 (1.9)	3 (1.8)	65	23.1%
	Girl	12.6 (1.8)	3.1 (1.8)	121	45.5%
Recruited as Siblings	Boy	13.1 (2.1)	2.7 (1.8)	50	30.0%
	Girl	13.2 (2.1)	2.7 (2)	37	40.5%

**TABLE 33:** Frequencies of Visit‐to‐Visit Trajectories in Target of Attraction

Trajectory	Total	Recruited as Transgender	Recruited as Cisgender	Recruited as Siblings
*Only boys* to *Both boys and girls*	45	14	27	4
*Only girls* to *Both boys and girls*	31	14	7	10
*Both boys and girls* to *Only boys*	22	13	6	3
*Both boys and girls* to *Only girls*	17	8	3	6
*Both boys and girls* to *Neither boys nor girls*	7	4	1	2
*Only boys* to *Both boys and girls* to *Only boys*	7	4	2	1
*Both boys and girls* to *Only girls* to *Both boys and girls*	6	3	2	1
*Only boys* to *Neither boys nor girls*	5	1	3	1
*Only boys* to *Only girls*	5	0	4	1
*Only girls* to *Neither boys nor girls*	5	2	3	0
*Both boys and girls* to *Only boys* to *Both boys and girls*	4	1	3	0
*Only girls* to *Both boys and girls* to *Only girls*	4	1	3	0
*Only boys* to *Neither boys nor girls* to *Both boys and girls*	3	2	1	0
*Only girls* to *Both boys and girls* to *Neither boys nor girls*	3	1	2	0
*Only boys* to *Only girls* to *Both boys and girls*	2	1	1	0
*Only girls* to *Only boys*	2	0	1	1
*Both boys and girls* to *Neither boys nor girls* to *Only boys* to *Both boys and girls*	1	1	0	0
*Both boys and girls* to *Neither boys nor girls* to *Only girls*	1	1	0	0
*Both boys and girls* to *Only boys* to *Neither boys nor girls*	1	1	0	0
*Only boys* to *Both boys and girls* to *Only girls*	1	1	0	0
*Only boys* to *Neither boys nor girls* to *Both boys and girls* to *Only girls*	1	1	0	0
*Only boys* to *Neither boys nor girls* to *Only boys*	1	1	0	0
*Only boys* to *Only girls* to *Only boys*	1	0	1	0
*Only girls* to *Both boys and girls* to *Neither boys nor girls* to *Both boys and girls*	1	1	0	0
*Only girls* to *Both boys and girls* to *Only girls* to *Both boys and girls*	1	1	0	0

*Note*: Only youths who have shown change are represented here. This table does not include change from *Neither boys nor girls* if the youth had never expressed interest in boys or girls at any point prior.

Did youths differ in their reported levels of stability and change in sexual orientation based on their *recruitment group* (*Recruited as Transgender*, *Recruited as Cisgender*, and *Recruited as Siblings*)? We fit a logistic mixed‐effects regression model predicting whether a youth showed stability in their attraction from a youth's *recruitment group* and the amount of *time elapsed* (in years, mean‐centered) between their earliest and most recent reports of attraction, as well as a random intercept for each family to account for sibling pairings in the dataset. *Time elapsed* was included in this analysis, as in Chapter IV, because people with more time between reports had more time to change their identity. A Type II Wald χ^2^ test indicated that *recruitment group*, Wald χ^2^(2) = 0.90, *p* = .639, was not significantly related to showing stability or change in attraction, though there was a significant effect of *time elapsed*, Wald χ^2^(1) = 9.91, *p* = .002, such that participants with more time between reports were more likely to show change in attraction.

### VI.8 Discussion

This chapter examined sexual orientation among youths in the Trans Youth Project. We had three main aims: (1) to describe youths' sexual orientation; (2) to assess if childhood gender development measures—namely, gender‐typed preferences for toys, clothing, and peers, as well as gender identity on a continuous scale and parent‐reported gender typicality—were related to current sexual orientation; and (3) to describe levels of stability and change in youths' attraction. We describe findings regarding these three aims in turn.

When youths were younger (primarily under 12), roughly a third of youths who were binary transgender or cisgender, and half of youths who were gender diverse, reported having *not* had a crush on a boy or girl; binary transgender youths were more likely to express queer attraction than cisgender youths at these young ages as well. At youths' most recent visit as teenagers, we again found that more binary transgender youths expressed queer romantic attraction than cisgender youths. Binary transgender youths reported queer attraction at about double the rate of queer attraction among cisgender youths (60% vs. 33%). Girls were more likely than boys to be queer, and gender‐diverse youths were more likely than binary transgender and cisgender youths to be interested in both boys and girls.

These results corroborate several patterns in recent research on youth sexual orientation. First, more youth in the United States are identifying as queer (Jones, 2024, 2025; Mpofu et al., 2023; Conron et al., 2020). Second, transgender and gender‐diverse youth appear to identify as queer at higher rates than cisgender youth (e.g., Suarez et al., 2024; Gower et al., 2022; Szoko et al., 2023; Boskey & Ganor, 2022; Price‐Feeney et al., 2020; Bosse & Chiodo, 2016). Our sample does not show any evidence that early identifying transgender children overwhelmingly show exclusive interest in other people of their same assigned sex (e.g., many transgender girls in our sample were interested in girls, rather than only being interested in boys). This was also true if we looked only at transgender girls—only 32.0% of youths recruited as girls in the *Recruited as Transgender* group showed exclusive interest in boys (see Supporting Information for Chapter VI for details). This finding contradicts older theories about the sexuality of transgender women who come out early in development as transgender (e.g., Blanchard, 1988) which posited that young people who are assigned male but identify as girls early in life will overwhelmingly be attracted only to males later in life.

Our results echo other research suggesting that people with nonexclusive attraction (e.g., bisexual or pansexual) represent the largest subgroup of queer identities among young people (e.g., Jones, 2024, 2025; Mpofu et al., 2023; Phillips et al., 2024) and queer people in general (Diamond, 2014). Past work (usually, but not always, with individuals with binary gender identities) has shown that being attracted to people of multiple genders is more common than experiencing exclusively gay or lesbian attraction, even though people often (inaccurately) think the opposite is true. Our results are in line with this claim—more youths were bisexual than were gay or lesbian, and this was particularly true of girls, mirroring some past work suggesting that (cisgender) girls and women are more likely to experience non‐exclusive patterns of attraction (Chandra et al., 2013; Dickson et al., 2003; Dickson et al., 2013; Mosher et al., 2005; Savin‐Williams et al., 2012).

From older youths' online survey responses, we also evaluated whether they were asexual or aromantic, romantically interested in nonbinary people, and questioning their sexuality. Gender‐diverse youths were more likely to be asexual or aromantic and to be romantically interested in nonbinary people than binary transgender or cisgender boys and girls. Girls were more romantically interested in nonbinary people than boys. Additionally, youths who expressed interest in both boys and girls were more interested in nonbinary people than youths who were romantically interested in either boys or girls (but not both). There were no differences in the likelihood of expressing questioning among different groups based on gender modality (i.e., transgender vs. cisgender vs. gender diverse) or identity (i.e., boys vs. girls vs. gender diverse), but youths who expressed attraction to both boys and girls were more likely to say they were questioning than youths who expressed exclusive attraction to either boys or girls.

Our second major question in this chapter was whether measures of childhood gender development were related to current sexual orientation. We used three childhood gender development measures: *Continuum* (continuous gender identity on a 0–100 scale), *Preference* (a composite score on tasks assessing gender‐typed preferences for peers, toys, and clothing), and *Parent Questionnaire* (a parent‐report instrument assessing masculinity and femininity of children's behavior, activity preferences, and attitudes about their gender). Analyses with these measures found that all three were related to current sexual orientation in youths who are currently boys or girls; this was no more or less the case in youths who are today binary transgender versus cisgender. These results point to the same conclusion as past work showing that childhood gender‐typed preferences and identity are related to later sexual orientation (e.g., Li et al., 2017; Steensma et al., 2013b); we expand upon that work by demonstrating that gender modality did not moderate this effect. However, one important distinction between past work and our own is that nearly all of the participants in past work identified as cisgender at the later time point. In our study, we show that even among transgender adolescents, the general association holds between gender‐typed preferences and identity in childhood and later sexual orientation.

We note that several characteristics of the current study warrant interpreting these data with some caution. Our relatively small sample sizes (especially when considering all the combinations of gender identity, gender modality, and current attraction that we were trying to analyze) may have led to imprecise model estimates and insufficient power. We tried to minimize this concern by combining gay and bisexual boys into a single group for analyses dealing with the relation between childhood gender development and current sexual orientation; however, this adjustment slightly changes the research questions we can answer. Furthermore, many participants have not provided enough data to be included in these analyses (meaning that those who have may not be representative of the entire sample). We also were not able to thoroughly examine whether childhood gender development measures predicted current attraction to youths who are currently gender diverse, given the sample size and interpretation limitations (though it was clear from the data we could examine that they were overwhelmingly interested in *both boys and girls*). In sum, our results show that early childhood gender development measures may hold some predictive signal of later sexual orientation in both cisgender and transgender youth.

Finally, in examining stability and change in sexual orientation, we found that while the majority of youths who could show change did *not* do so (61.5%), a substantial minority did (3.5%). This finding supports literature that has suggested that fluctuation in sexual orientation over time is not rare and that—perhaps by definition—this is more common among queer people (Savin‐Williams & Ream, 2007; Savin‐Williams et al., 2012; Ott et al., 2011; Dickson et al., 2003; Dickson et al., 2013). Moreover, participants in different *recruitment groups* (*Recruited as Transgender, Recruited as Cisgender*, and *Recruited as Siblings*) were no more or less likely to show change.

### VI.9 Summary

Among TYP participants who have provided data about their sexual orientation as teenagers, a large percentage (60% of transgender adolescents, 33% of cisgender adolescents) reported some variety of queer attraction. These results accord with recent data suggesting a major generational shift in the United States in the number of queer young people in the United States and past work suggesting that transgender youths are more likely to be queer than cisgender youths. By far the largest subgroup of queer participants in the TYP are interested in both boys and girls, rather than being exclusively gay or lesbian. We find no support for older theories which speculated that early‐identifying transgender girls would be overwhelmingly attracted only to boys and men; only 32% of youths who started the study as transgender girls most recently expressed interest exclusively in boys. Among participants who have reported their sexual orientation multiple times, a large percentage—38%—have reported sexual orientation change, again refuting claims from several decades ago that sexual orientation is a static trait and corroborating research from the past several decades suggesting that sexual orientation change is common. Teenagers who were gender diverse (that is, neither boys nor girls) were more likely to be attracted to nonbinary people, more likely to be asexual, and more likely to be bisexual than those who are binary transgender or cisgender. Participants often identified with labels in much more flexible ways than past researchers considered possible (e.g., self‐identifying as asexual lesbian, straight but interested in nonbinary people, etc.), providing more evidence that teenagers in the present day are defying basic assumptions about sexual orientation—both those traditionally held in the research literature, and those held broadly in society.

## VII Key Findings, Strengths, Limitations, Future Directions, and Implications

VII.1

Since the 1950s, the question of how gender and sexual orientation develop has been at the forefront of our field. In the domain of gender development, renowned developmental psychologists such as Lawrence Kohlberg, Walter Mischel, Eleanor Maccoby, and Carol Jacklin, among many others, built a foundational literature describing how children (primarily in the U.S. and similar wealthy Western countries) come to understand their and other peoples' genders—and how gender comes to play a central role in children's self‐concepts, relationships, preferences, attitudes, and stereotypes. Undergirding this empirical effort was the general goal of understanding how children develop gender‐typed knowledge and traits with such striking regularity. In much of this work, researchers held the assumption that, barring extremely unusual circumstances, assigned males would come to think of themselves as boys and assigned females would come to think of themselves as girls.

Recent generational shifts have highlighted that the developmental story of gender may not be as straightforward or predictable as this classic literature suggested it was. Over the past decade across the United States, Canada, the United Kingdom, and Western Europe, larger numbers of youth have come out as transgender and nonbinary (Suarez et al., 2024; Brown, 2022; StatCan, 2022; McKechnie et al., 2023; Kidd et al., 2021; Minnesota Department of Education, 2022) and a subset of them—more than in past generations—have begun seeking gender‐affirming medical treatment from gender clinics (Bauer et al., 2021; Masala et al., 2024; Spack et al., 2012; L. Thompson et al., 2022; van der Loos et al., 2023; Vandendriessche, 2023). In other words, a consequential shift appears to be occurring in how young people experience their gender. As transgender and gender‐ diverse youth gain visibility—with their identities and rights becoming central to political agendas and heated scientific debates—it becomes increasingly critical that new psychological research helps the public understand how these youth's identities develop over time. Furthermore, as queer identities become far more common among today's young people, developmental scientists can add to our knowledge of how these youths understand their sexual orientations over time, and how this may or may not accord with years of past research on the topic.

In this monograph, we began to address some of these gaps in the literature by reporting on stability and change in gender identity and sexual orientation among over 900 North American youths who fell into three groups: (1) those who were supported by their families in a social gender transition by age 12 (*Recruited as Transgender*); (2) a group of unrelated cisgender youths who were age‐ and gender‐matched to the *Recruited as Transgender* group (*Recruited as Cisgender*); and (3) siblings of youths in the *Recruited as Transgender* group (*Recruited as Siblings*). We followed the youth in this sample from an average age of 8.1 years at their first visit with our research team to an average age of 14.3 years at their most recent visit. In this final chapter, we summarize the main empirical findings from previous chapters and discuss their implications for psychology, as well as for broader society. We also highlight strengths and limitations of our contribution and possible directions for future research.

### VII.2 Key Findings

#### VII.2.1 Relatively High Identification as Transgender, Gender Diverse, and/or Queer Compared to Historical Estimates

A major finding in this work is that youth reported relatively high levels of gender‐diverse gender identities (e.g., nonbinary) and queer sexual orientation compared to what one might expect based on literature from two or more decades before, likely reflecting a major cultural shift in American and Canadian youth's thinking about gender. In our sample, a particularly large number of youths identified as gender diverse in all *recruitment groups*: 8.5% in the *Recruited as Transgender* group, 6% in the *Recruited as Cisgender* group, and 11.9% in the *Recruited as Siblings* group. The prevalence of gender‐diverse identity in all three groups, as well as the relatively high prevalence of binary transgender identity in the *Recruited as Cisgender* (3.9%) and *Recruited as Siblings* (3.4%) groups, indicates that our participants are thinking about gender more flexibly than youth from previous generations, both in the sense that they view gender as more malleable across time and that they identify with options other than “boy” or “girl” more often.

It is not possible to know precisely how representative our cisgender comparison groups are of broader U.S. and Canadian youth. Nonetheless, the patterns observed are consistent with other empirical research from the past 10 years suggesting that a generational change in how youth think about gender is occurring. Just 5 to 10 years ago, being a transgender child or teenager was considered rare (1% or less of the population; Meerwijk & Selvius, 2017; Zucker, 2017). The Williams Institute released estimates in 2017 and 2022 of the percentage of United States youth ages 13–17 that were transgender; the 2022 estimate (1.4%) was nearly double that of 2017 [0.7% (Herman et al., 2017, 2022)]. Data from the 2023 Youth Risk Behavior Survey, administered by the Center for Disease Control, found that 3.3% of high‐school students (roughly ages 14–18) were transgender and an additional 2.2% were questioning whether they might be (Suarez et al., 2024). Pew Research Center estimates in 2021 suggested that the number of adults ages 18–29 who know a trans or nonbinary person had risen to 51% (9 points higher than their 2017 estimate), and in 2022 estimated that 5% of young adults in the same age range were transgender or nonbinary (Minkin & Brown, 2021; Brown, 2022). An analysis of data from the United States Census Bureau's Household Pulse Survey reported that 7% of Generation Z adults in 2024 (i.e., those in their late teens or early‐to‐mid‐20s) identified as nonbinary (Twenge, 2024). Finally, more geographically localized estimates from school districts in Minnesota and Pennsylvania, and from a sample of 18–22‐year‐old youth in the Seattle area (where most of our *Recruited as Cisgender* group was recruited), suggest even higher rates of transgender or gender‐diverse identity, around 10–15% (Minnesota Department of Education, 2022; Kidd et al., 2021; King et al., 2024). This emerging research suggests that the 9.8% and 15.3% transgender or gender‐diverse identification rates in the *Recruited as Cisgender* and *Recruited as Siblings* groups may exceed some national estimates, but are not extreme outliers and likely reflect contemporary rates in our participants' geographic regions. At the least, our results are in line with the temporal trend of increasing transgender and gender‐diverse identification appreciable in recent literature.

We also found that many participants, in all *recruitment groups*, were queer (i.e., not straight). At most recent report, 60% of binary transgender and 33% of cisgender youths who had contributed data on their sexual orientation were gay, lesbian, or bisexual. While estimating the prevalence of queer identity among the general population is a challenging endeavor that is the subject of its own extensive literature, we can confidently say that these rates are higher than both historical and contemporary estimates of the adult queer population in the United States and Canada, which are typically between 2% and 10% (Jones, 2024, 2025; Gates, 2014; Flores & Conron, 2023). They are more in line, however, with recent data about youth's sexual orientation identities, which have shown a steady increase in the past 15 years in queer identification. For example, data from the Youth Risk Behavior Survey in 2021 showed that over 20% of respondents (U.S. high‐school students, approximately ages 14–18) did not identify as heterosexual, as opposed to slightly over 10% in the same survey from 2015 (Mpofu et al., 2023). Similarly, Gallup polls in 2023 and 2024 found that over 20.0% of Gen‐Z adults (born between 1997 and 2005) were LGBTQ+ (Jones, 2024, 2025). Some individual research studies with large samples, particularly in urban areas, are finding even higher rates, more comparable to those in the present study (e.g., King et al., 2024).

A particularly notable trend in our findings regarding gender and sexual orientation is that a large number of youths in our sample reported identities that are not on either end of a binary spectrum. Across all three *recruitment groups*, 37.1% of youths who gave a report of their sexuality in an online survey identified as bisexual, pansexual, or queer, far outnumbering youths who expressed being gay or lesbian. This phenomenon accords with prior population‐representative studies of sexuality which have repeatedly shown that among people who are not straight, nonexclusive attraction (such as bisexual, pansexual or queer) is more common than exclusive same‐gender attraction, despite common misconceptions that the opposite is true (Diamond, 2014).

Perhaps more surprising with regard to past literature is the relatively high prevalence of gender‐diverse identity (most often nonbinary) in our sample: across all three *recruitment groups*, 8.3% of youths who have contributed longitudinal data identified as gender diverse at their most recent report (6.2% according to parent report). While this is certainly higher than we would have observed even 5 or 10 years ago, it tracks with emerging data that show increasing numbers of youths expressing nonbinary identities (e.g., Twenge, 2024). The idea that youth can identify as between or beyond the binary identities of “boy” or “girl” is not new. Psychologists have argued that gender should be conceptualized and measured as a multidimensional, spectrum‐like concept for decades (e.g., Bem, 1974; Egan & Perry, 2001; Hyde et al., 2019; Martin et al., 2017, Tate et al., 2014) precisely because people can and do subjectively view masculinity, femininity, manhood, and womanhood as characteristics that one can possess in varying amounts. What appears to be unique to recent cohorts of youth (including those in our sample) is that this variation is much more likely to manifest in youth adopting a new category label for themselves, whereas this would have been exceedingly rare even 15 years ago, at least in the United States and Canada. In fact, even as we were setting up the study in 2013, there was no mention of nonbinary children (using that label or a related one) in our conversation with families, and we did not meet individuals using pronouns like they/them (a much more common occurrence today).

We also found a notable interaction between gender and sexuality such that gender‐diverse teens were more likely to be bisexual, pansexual, or queer than binary transgender or cisgender teens; they were also more likely to be romantically interested in nonbinary people. These findings accord with past research showing that nonbinary youth are relatively more likely than binary transgender or cisgender people to experience nonexclusive attraction, and relatively less likely to describe themselves as heterosexual (Szoko et al., 2023; Boskey & Ganor, 2022). Our results further suggest that being gender diverse may, for at least some youth, represent a general openness when it comes to gender categories, sexual orientation, and attraction. For these individuals, gender and sexual orientation may not be as separable as they are for others; this is an intriguing direction for a follow‐up study of gender‐diverse youth.

#### VII.2.2 Stability Was the Most Common Trajectory

Even though we found higher rates of transgender and gender‐diverse identity than would be suggested by research from the 2010s or before, it is still the case that stability in gender identity was the most common trajectory among youths in our sample. Of youths who have contributed longitudinal self‐report data, 81.6% of the *Recruited as Transgender* group, 86.0% of the *Recruited as Cisgender* group, and 80.8% of the *Recruited as Siblings* group have not reported any gender change across approximately 6 years of participating (these numbers are even higher according to parents' reports: 88.5%, 91.6%, and 86.5%, respectively). Overall, 87.9% of youths in all three groups are currently the same gender that they were at the beginning of the study (91.0% according to parent report). While parents reported slightly more stability than youths, several key patterns held regardless of whether youths' or parents' reports were examined: (1) there was no significant difference in levels of stability and change over time shown by youths in the three *recruitment groups*; (2) there was no clear period of development in which changes were particularly likely to occur; (3) when youths did show change, it most commonly involved a change to or from a gender‐diverse identity, as opposed to changing from one binary gender to the other.

These results have important implications for both early‐identifying, socially transitioned transgender youth, as well as those who are cisgender in childhood. Our data suggest that most socially transitioned transgender youth will persist in their gender identity into adolescence and that they are not more likely than cisgender youth to show fluctuations in their gender identity throughout childhood and adolescence (though some do). This is a different conclusion than was reached in past research with clinical samples of gender‐nonconforming children, most of whom “desisted” and did not end up identifying as transgender (or the equivalent term at the time) as adolescents or young adults (Green, 1987; Zucker & Bradley, 1995; Drummond et al., 2008; Wallien & Cohen‐Kettenis, 2008; Steensma et al., 2013a; Singh et al., 2021).

There are many reasons why the socially transitioned children in our sample may have persisted in their transgender identities at much higher rates than clinical samples in past work. First, these youths were supported by their families in a transition. Even if some parents may have initially reacted negatively to their child's insistence that they were a different gender, they eventually came to support a social transition. These youth have also grown up in an era in which social transitions were significantly more accepted and visible than when past work was conducted. This level of acceptance may have allowed them to feel comfortable in their genders without as much shame about, or pressure to change, their identities as they might have in previous generations. This sample also had earlier and greater access to gender‐affirming medical care, which may have helped them feel more comfortable in their developing bodies. It is notable that the transgender youth in this study, at least through the time period of the current work, show comparable mental health to the cisgender youth in this sample (Wittlin et al., 2025).

Youths in our sample may also have shown stronger gender nonconformity on average than youths in clinical samples (Olson, 2016), many of whom did not actually insist that they *were* the other binary gender (Zucker & Bradley, 1995), which was common among youths in our sample. Our research team's past work with a different subset of youths suggested that social transition is itself associated with greater gender nonconformity in childhood—this gives more reason to believe that the current *Recruited as Transgender* group might have shown levels of pronounced gender nonconformity from early ages that were uncommon in clinical samples. Given past work linking greater gender nonconformity to persistence of transgender identity (Singh et al., 2021; Steensma et al., 2011; Wallien & Cohen‐Kettenis, 2008), the possibility that gender nonconformity was stronger among youths in our sample might account for the difference in trajectories.

It is also the case that many of the youths in the current study are still adolescents and may potentially change genders in the future; some clinical samples were followed for longer periods or followed youth to older ages than we have (e.g., Green, 1987; Wallien & Cohen‐Kettenis, 2008; Steensma et al., 2013a; Singh et al., 2021; Drummond et al., 2008). We will note that among youths in the *Recruited as Transgender* group whose most recent visit was at age 18 or older, 85% were still identifying as the *recruitment gender*—a similar percentage to the 88% shown in the *Recruited as Transgender* group overall, suggesting that the younger age of our participants relative to past work's samples is likely not driving the discrepancy. Additionally, there is some emerging evidence that a subset of youths in past clinic‐based studies who initially detransitioned later returned to the clinics in adulthood, retransitioning to live as transgender (Steensma & Cohen‐Kettenis, 2015; A. de Vries, personal communication, 2024). We cannot know exactly which, if any, of these reasons (or, likely, combination of reasons) account for why persistence in transgender identity has been so much more common in this sample relative to past work with clinical samples.

We also found that the vast majority of youths in the *Recruited as Cisgender* and *Recruited as Siblings* groups have reported no change in their gender identity. However, between 10% and 20% of these youths *did* express gender change. While this estimate is not significantly different from the estimate for the *Recruited as Transgender* group, it may be surprising to readers for the opposite reason, in that it may seem higher than expected. This finding contrasts with how gender identity has traditionally been conceived in the gender development literature: that is, a stable trait that people acquire an understanding of at around age 3 (Thompson, 1975; Slaby & Frey, 1975). In fact, influential theories of gender development in the cognitive developmental (i.e., Piagetian) tradition dating back to the 1960s argued that children who say it is possible for a person's gender to change have not yet developed the full cognitive maturity necessary to (in the views of past work, correctly) reason that gender is an unchangeable trait (Kohlberg, 1966). In other words, thinking that gender could possibly change was a sign that some more cognitive development needed to occur in order to show adult‐like reasoning. At the same time, concurrent research on persistent gender nonconformity (at least in assigned males) was typically conducted in clinical settings. This work sometimes conceived of a children's indication that gender could change or that their gender did not align with their sex as indication of a developmental delay (Zucker et al., 1999), providing further indication that deviation from one's assigned sex at birth was viewed as a rare aberration from a more correct or developmentally appropriate view of gender and its stability across the lifespan. The fact that a sizable minority of our initially cisgender participants have shown gender change suggests that, contrary to the assumptions that have dominated much gender development research since the 1950s, today's youth can and do change how they think about their gender category over time—an outcome considered so rare and counter‐normative in past work that researchers and society essentially took for granted that it would not occur.

Our data are thus consistent with two core phenomena: most young people show stability in their gender identity over time, *and* it is not exceedingly rare for contemporary youth to experience gender change, particularly to nonbinary or gender‐diverse identities. Put differently, stability in gender identity and identification with one's birth sex are the modal pathways among youth who are identified as cisgender in childhood, but there is variation in how much and how consistently youth identify with gender categories over time. These findings indicate that the cultural forces—whatever they may be—that have set the stage for more youth to express gender fluidity and flexibility are not unique to early identifying transgender children.

#### VII.2.3 Some Evidence that Childhood Preferences and Continuous Identity Predict Later Categorical Identity

We examined whether three gender development measures that youths (or their parents) completed were related to their later (current) gender identity and sexual orientation. These measures included their gender identity on a continuous 0–100 scale (*Continuum*); gender‐typed preferences for peers, toys, and clothing (*Preference*); and a parent‐report instrument of gendered behavior, preferences, and identity used in past work with clinical samples of gender‐nonconforming children (*Parent Questionnaire*; Johnson et al., 2004). We asked if youths whose genders changed from their early identity showed more gender‐atypical responses on these measures (which were given on average 5.4 years prior to their most recent report of their gender) than those whose genders remained the same later on. We found tentative evidence of this effect with the *Preference* and (to a lesser extent) *Continuum* childhood measures, but not the *Parent Questionnaire*. Results from the *Preference* measure suggested that a child who, for example, started and ended the study as a boy tended to have more masculine preferences and a more masculine identity score in childhood than a child who started the study as a boy and today lives as a nonbinary person or girl. This was also the case for the *Continuum* measure, but only in youths who were boys in childhood; youths who were girls in childhood did not show this effect.

We performed a similar series of analyses to examine the relation between early gender development and current sexual orientation. To do so, we looked at the earliest *Preference*, *Continuum*, and *Parent Questionnaire* scores among the subset of youth who are currently boys or girls. Among boys, those who are currently straight had more masculine early *Preference*, *Continuum*, and *Parent Questionnaire* scores than those who were bisexual or gay. Among girls, those who are currently straight had more feminine earliest *Preference* and *Parent Questionnaire* scores than those who were bisexual or lesbian.

Taken together, we sometimes, but not always, found evidence linking childhood gender development to current identity and sexual orientation. Results were always in the expected direction given past literature (Wallien & Cohen‐Kettenis, 2008; Steensma et al., 2013b; Zucker & Bradley, 1995; Drummond et al., 2008; Rae et al., 2019; Singh et al., 2021; Bailey & Zucker, 1995; Li et al., 2017). However, we urge some caution in interpreting our results given the very small number of youths with particular trajectories: our estimates of early scores for (for example) cisgender gay boys or youth who have made a binary transition during the study period are highly imprecise because there were only a few youths who showed these outcomes in our sample. Nonetheless, our data suggest the presence of two important phenomena with regard to predictors of gender identity and sexual orientation later in development. First, identity and gender‐typed preferences did show some (albeit small) predictive signal for later identity and identity change. Second, within youth who currently report any given gender identity or sexual orientation, there is substantial variability in the preferences and continuous identity they may have expressed earlier in development.

#### VII.2.4 Relation of Work to Broader Theories of Childhood Gender Diversity

The data we report in this monograph allow us to reflect on some bigger, broader questions about childhood gender diversity. One central theoretical question in the developmental and clinical literature concerns what causes gender diversity in youth. Theories often posit a range of social, biological, and environmental factors. For example, Zucker et al. (2012) suggested that young children may have a biological predisposition toward gender nonconformity and that parents' tolerance or encouragement of gender nonconformity may facilitate gender nonconformity. Research on the biological basis of gender identity is far from conclusive, with some evidence suggesting that factors such as hormones (e.g., Dessens et al., 2005; Hines, 2009) or genes (Iervolino et al., 2005) may play a role, at least in some cases. We have no data in the present study to directly speak to the role of biological factors in the emergence of gender diversity as biological measures have not been collected. The rate of gender diversity in the *Recruited as Siblings* group was numerically higher than it was in the *Recruited as Cisgender* group, but we did not find significant group differences, so we cannot draw a clear conclusion about whether genetics played a role. Furthermore, youths in the *Recruited as Siblings* group share a social environment with their transgender siblings, so even if we had observed a significant difference, attributing it to biological factors would be difficult.

Another common idea in the literature is that parents in some way contribute to or facilitate gender nonconformity in their children (e.g., Green, 1987; Zucker & Bradley, 1995). We are skeptical of this interpretation of our data for a few reasons. First, we see that most of the *Recruited as Siblings* youths—who grew up in the same families as the *Recruited as Transgender* youths—are cisgender. Presumably, if parents were encouraging gender nonconformity, in general, we might see a higher rate of transgender identity among the siblings. Furthermore, as reported in past research with this sample (Gülgöz et al., 2019; Supplement), we had coders naïve to participants' gender rate photographs of our transgender participants from their first years of life—an age at which parents (rather than children themselves) select outfit choices and bedroom decorations. If parents were raising the later‐identifying transgender children in hopes of having transgender children, or with an eye toward gender nonconformity, we would expect to see counter‐stereotypic clothing and bedroom decor. In contrast, these photos indicated that the transgender youths were initially wearing clothes and living in bedrooms that aligned with sex‐based stereotypes (e.g., a transgender girl was brought home from the hospital to a room that was stereotypically masculine and dressed in masculine clothing) in their first year of life.

In discussions with parents—during early study visits—they often told us that their child's gender nonconformity emerged early and that it came as a surprise. Some families initially recalled reacting negatively and trying to stop it. Other families ignored it. Still others openly allowed it or embraced it as a response to society's rigid gender stereotypes. Almost none of the families reported thinking their child was transgender right away. Far more thought perhaps their child was going to be gay or was simply a bit more masculine or feminine than the other children in their family or in the neighborhood. Many showed us photos or videos of their very young children turning household supplies—towels, string—into stereotypically gendered accessories (e.g., long hair, necklaces).

These stories and accompanying materials suggest to us that most of these youth had an internal experience of gender quite early. Our read of the literature—which accords with our participants' lived experiences—is that gender itself is culturally defined and delineated; there is nothing inherently “feminine” about a particular hair length or the presence of a necklace. Like all children, the children in our sample took in early cultural ideas of gender and how it is instantiated. Youths in the *Recruited as Transgender* group seem to have interpreted something in their internal experience as translating within this cultural context to a particular gender identity (and we will note that this may also be the process of gender identification for cisgender youth too—we just do not tend to think of it that way). While our study cannot lay out a definitive explanation of how this happens, we hope our data can contribute to the broader discussion of childhood gender diversity and to future theoretical advancement in this area.

### VII.3 Strengths and Limitations

This work has several notable strengths and limitations. We discuss these below as they relate to four aspects of the work: (1) characteristics of our sample of youths and parents; (2) participation over time, retention, attrition, and missing data; (3) our assessment of gender and sexual orientation; and (4) decisions related to analytic strategies and open science practices.

#### VII.3.1 Characteristics of Our Sample

As the first longitudinal study on socially transitioned transgender youth, the Trans Youth Project sample carries many strengths. First, it is a relatively large sample. Few if any studies have attempted, let alone successfully followed, so many youths who were “gender nonconforming” (from the perspective of their sex assigned at birth) in childhood through adolescence. None have provided as extensive documentation of their gender development during that time.

Second, the inclusion of the *Recruited as Cisgender* and *Recruited as Siblings* groups as contemporaneous comparisons to the *Recruited as Transgender* group significantly increases the informativeness of the current work by allowing us to situate results about the *Recruited as Transgender* group in the context of what is happening at this unique historical moment in the United States and Canada. For example, between 15% and 20% of early‐identifying transgender youths in our sample have shown gender change—but is this more or less than what we should expect? Our inclusion of the *Recruited as Cisgender* and *Siblings* groups—groups with many similar demographic characteristics such as primarily coming from politically liberal, highly educated households—grants us a better understanding of what the early‐identifying transgender youths' outcomes mean. In this particular case, they point to the intriguing conclusion that socially transitioned children show strikingly similar trajectories, rates of change, and current identities as their cisgender counterparts.

Another strength is that we included data from both youth and parents. The fact that all major results relating to levels of stability and change in the youth's identities (e.g., stability over time as the most common trajectory, lack of significant differences between groups, most changes involving change to or from gender‐ diverse identity) replicate in results from parent and youth reporters gives us confidence that our results are not, in large part, due to reporter effects. Inclusion of parents also allowed us to test a secondary empirical question—the extent to which youth and parents report the same gender identities for the youth. We found that in over 90% of cases in which youth and parents simultaneously provided reports of the youth's identity, their reports agreed. We consider this a very high percentage, considering that we initially coded gender using a fairly complex 5‐category coding scheme (boy, boy‐expansive, gender diverse, girl‐expansive, girl) that allowed for significant nuance in reporting. We also found that in cases when two parents provided reports at the same time point, they agreed on their child's gender identity an overwhelming majority of the time (>97%).

In addition to these strengths, our study and sample also carry several limitations. Relative to the demographics of the United States and Canada overall, all three of our *recruitment groups* were disproportionately White, non‐Hispanic (69% of our sample), and families from middle‐to‐upper SES were overrepresented (71% of our sample had a household income of greater than $75,000, a value that is near the current median income in the U.S.). Parents are disproportionately highly educated (83% of youths in our sample had a parent in the household with a college degree) and politically liberal (see Chapter II for details). We do not know how our results would or would not generalize to a more racially diverse, lower‐SES, or politically conservative sample. Some data from the past decade have suggested that youth of color and lower‐SES youth may be as likely or more likely to identify as transgender in adolescence as white, upper‐SES youth (Kidd et al., 2021; Kahn et al., 2024; Minnesota Department of Education, 2022; Day et al., 2018), but it is not clear whether youth of color and lower‐SES youth undergo childhood social transitions, have access to the same types of resources (e.g., gender affirming care), or access to the same levels of support (e.g., from family members, therapists, physicians, etc.) as the youths in our study do. As a result, we cannot be certain if our sample was or was not representative of the youth who were socially transitioning between 2013 and 2017 or if a more diverse sample of youth would show similar trajectories to the ones we observed here.

In addition, the *recruitment groups* are not perfectly matched demographically. While all three groups are disproportionately white (and multiracial), from upper‐SES backgrounds, and with politically liberal parents, the *Recruited as Cisgender* group tended to be slightly higher‐income, slightly more politically conservative, and more concentrated in the Seattle metropolitan area than the *Recruited as Transgender* and Siblings groups. That these families come primarily from the Seattle region may explain their higher incomes and their self‐reports of less “liberal” attitudes. People in Seattle have high incomes relative to national averages (Balk, 2024b) and the city is very liberal (Tausanovitch & Warshaw, 2014; Balk, 2024a; Bowman, 2020). This could potentially skew *Recruited as Cisgender* parents' reports of their political leanings if they are comparing themselves to the people they know, rather than the country as a whole. So conversely, parents in the *Recruited as Transgender* and *Recruited as Siblings* groups tend to live in more politically diverse locations in which their relatively more liberal attitudes might be more salient.

Both the *Recruited as Cisgender* and *Recruited as Siblings* groups have advantages and disadvantages in terms of their role as a comparison group to the *Recruited as Transgender* group. On the one hand, the *Recruited as Siblings* group is a better match demographically (and genetically) to the *Recruited as Transgender* group. They also have a transgender youth living in their household, and this could plausibly impact their own trajectories by making them more likely to consider or reflect upon their own gender fluidity and/or nonconformity. On the other hand, the *Recruited as Cisgender* group probably better approximates the average U.S. or Canadian youth's exposure to conversations about, and examples of, gender diversity and change over time (though the *Recruited as Cisgender* group is likely also subject to some degree of self‐selection bias, given that parents have agreed to have their children's development be tracked in a longitudinal study about gender identity that included transgender youth, signaling some level of acceptance of the topic).

Our sample notably does not contain certain groups of youth whose trajectories could be informative. For example, we did not sample a group of youth who experience gender dysphoria but who are not able to transition for some reason (e.g., their families are not supportive). Recruiting this group is extremely challenging because, in our experience, parents who are not supportive of their children's gender identities are typically not eager to participate in a long‐term study tracking their children's identities. As a result, we do not know whether the high levels of stability we found in our *Recruited as Transgender* group would also apply to youth who feel like they are transgender or gender diverse early in development but do not undergo a social transition (or who do not have support for their identities at that time). We also do not have data from a large group of gender‐ diverse children *before* their transition, so we are limited in our ability to assess whether gender nonconformity prior to social transition is related to stability and/or change in gender identity later in development. However, other work from our research team with a small sample of youth who had not socially transitioned when they began participating in our research has found that gender nonconformity in childhood does predict later social transition (Rae et al., 2019), and we found some evidence pointing to a similar phenomenon in our *Recruited as Cisgender* and *Siblings* samples in Chapter V.

#### VII.3.2 Retention, Attrition, Missing Data, and Participation Over Time

Retention thus far in the Trans Youth Project has been high relative for field standards in developmental psychology. Of the 912 youths profiled in this study, we have had research participation from either parents and/or the youth themselves from 811 (88.9%) between January 2020 and February 2024, despite nearly all youth joining the study between 2013 and 2017. Participation has been highest in the *Recruited as Transgender* group, with 94.6% showing some participation since 2020 (the equivalent figures are 82.5% and 91.7% for the *Recruited as Cisgender* and Siblings, respectively). For 74.3% of our participants, we had two visits that were at least 5 years apart, and for 26.0% of our participants, we had two visits at least 8 years apart (in the *Recruited as Transgender* group, whom the project was primarily designed to study, these figures are 88.6% and 35.0%, respectively).

As we have presented findings from our sample over the years, we have often been asked about our high rates of retention in the study. While we cannot be certain what to attribute these high rates to, our sense is that having met all of the *Recruited as Transgender* (and *Recruited as Siblings*) families in person, often at their homes or in their communities, played a key role. Our team spent quality time with families, getting to know them during these visits. The same researchers often returned year after year, creating a sense of connection. Between tasks or while waiting for a family member to complete the study, parents often told us stories about the youth, furthering our rapport and understanding of these families' experiences. Furthermore, we have remained in contact with families through repeated invitations to participate and interim updates (e.g., newsletters documenting new team members, etc., which also go to *Recruited as Cisgender* families). Many families have expressed pride in being a part of this study—including those in the *Recruited as Cisgender* group.

A further strength relating to rate of participation in our sample is that we have had youths and parents participate several times throughout development, rather than only at the beginning and end of the study, as in most work on gender‐ nonconforming youth performed in clinical settings. This repeated participation throughout the study period has allowed us to describe participants' unique trajectories in a greater level of detail than we would be able to if we only examined one timepoint in childhood and one later timepoint in adolescence or adulthood.

However, attrition rates have not been zero nor are they uniform across groups. This leaves open the possibility that participants who have not continued in the study are qualitatively different from those who have, which (if true) would affect the *Recruited as Cisgender* group the most out of the three groups. Household income, household education, youth race/ethnicity (comparing White, Non‐Hispanic youth to all other youth), age at earliest visit, and sex assigned at birth (see Table 2, Chapter II) were not significantly associated with whether a youth (or their parent) had participated in a visit since January 2020 (*p*'s > .25; as expected, participant group was significantly associated, Wald χ^2^(2) > 100, *p* < .001, see Table 3, Chapter II for by‐group rates of activity and attrition). Though we cannot be certain that another (potentially unmeasured) variable distinguishes youth who have and have not participated since 2020, straightforward demographic factors do not appear to explain participation. Throughout the monograph, we have attempted to be as transparent as possible about what slice of the full sample has been included in each analysis, and full gender trajectories as reported by youth and parents to the research team are illustrated for all 912 youths in the study in Figures S3–S8 in the Supporting Information for Chapter IV.

For some analyses, we observed sufficiently little data loss that we can fill in the gaps left by participant attrition and get a sense of how results would have turned out if we make certain assumptions about those who dropped out. For example, Table 12 in Chapter IV shows that according to parent report, the three *recruitment groups* showed similarly high rates of gender stability: 88.5%, 91.6%, and 86.5% in the *Recruited as Transgender*, *Cisgender*, and *Siblings* groups, respectively. However, these figures do not include youths for whom we do not have a codable follow‐up report of their gender from a parent. If we assume (probably unrealistically) that all youths for whom we do not have a follow‐up have shown gender stability, these percentages would be 90.4%, 92.3%, and 87.2%, for the *Recruited as Transgender*, *Cisgender*, and *Siblings* groups, respectively. If we make the opposite (also unrealistic) assumption that all those for whom we do not have data have *not* been stable, these percentages would be 85.2%, 84.1%, and 82.6%. This rough imputation suggests that while differential attrition introduces some uncertainty in rates of parent‐report stability and change in the youth's gender, we can be reasonably confident that stability is by far the modal pathway in all three groups, with over 80% of youth showing stability regardless of assumptions made about those who have stopped participating.

Conversely, there are other research questions we address throughout the monograph for which a large portion of the sample (>30%) had not provided sufficient data to be included in the analysis (e.g., those testing the relation between *Preference/Continuum/Parent Questionnaire* and later gender identity or sexual orientation in Chapters V and VI). For these analyses, it is much harder to derive a reasonable range of estimated effects that we would expect given various assumptions about participants who have not contributed enough data to be included. For this reason, along with the fact that none of our analyses were preregistered (which we discuss later in this chapter), we encourage readers to interpret these results cautiously. However, we note that all effects we found when assessing the relationship between *Preference, Continuum*, or *Parent Questionnaire* and current identity were in the direction predicted by prior literature (i.e., when gender nonconformity earlier in childhood or adolescence was related to current identity, it was such that more gender nonconformity tended to be slightly related to later gender change or later queer sexual orientation).

#### VII.3.3 Assessment of Gender Identity and Sexual Orientation

Our assessment of gender identity and sexual orientation has been methodologically heterogeneous and has changed substantially throughout the course of the study. This can be seen as both a strength and a limitation. It is a strength because it has allowed us to flexibly adapt the wording in our measures to reflect new terminology and conceptualization of gender and sexuality as it evolved during the study period, as well as the increasing maturity of our participants. It is a weakness primarily because what appears to be a change in identity could be the result of the use of different response options or different questions, rather than an actual change in identity. A particularly pronounced version of this problem involved the fact that for younger children in the study, we often only assessed their gender with a measure (Measure 1, Table 6, Chapter III) that allowed youths to express that they were a boy, girl, or gender diverse (and did not include the option to indicate any other identities); then, later on in the course of the study, we introduced measures (primarily for older children and adolescents) in which youths could provide much more nuanced descriptions of their identities. It is therefore possible that a youth's identity reports in our study evolved over time simply because the measures they were given offered them increasing latitude to express specific identities as they got older.

As a result of so much variation, we may have overestimated the rates of identity change. However, there are some indications that our data are mostly robust to measure‐specific effects. In cases when youth answered two different gender identity measures at the same visit, their responses on both measures agreed with each other in 88% of cases. Furthermore, to counteract possible measure‐specific effects on our coding of youth's gender identity, we had human coders manually review all responses from visits in which there was a within‐visit discrepancy to come to a single determination of the youth's gender.

In coding youth's sexual orientations, we similarly employed a holistic approach in which coders viewed all relevant information from a particular visit to come to a decision, thereby reducing the possibility that any one measure, decontextualized from the rest of the youth's responses, could have skewed our assessment of their identity. In addition, the fact that interrater reliability was high across coding procedures, and the fact that key results hold across various alternative ways of coding the data presented in the Supporting Information (i.e., coding “girl‐expansive” and “boy‐expansive” as “gender diverse,” rather than as “boy” or as “girl”), give us confidence that our general results are robust to most question‐level effects.

Two other potential critiques of the validity of our assessments of gender and sexual orientation are worth addressing. The first, which is applicable to all youth in the study but primarily to those in the *Recruited as Cisgender* and *Siblings* groups, is that by asking children questions that included response options other than “boy” or “girl,” or by presenting youth with measures that implicitly or explicitly represented gender as a spectrum (rather than a binary), we introduced more flexible conceptions of gender to the youth that they then subsequently adopted for themselves (whereas they would not have done so had they never participated in the study). This issue points to a general tension inherent in trying to assess lay beliefs about gender (which, for many people, are strictly binary; Parker et al., 2022; PRRI, 2023) while also being inclusive of gender diversity.

Though we are unable to directly assess the extent to which participants' responses were or were not impacted in this way, we speculate that it is unlikely that exposure to our measures substantively shaped participants' identities over time. Because youth only participated for less than an hour once a year at most, we believe it is improbable that their experience participating in the study would exert a noticeable impact on their identities over and above whatever exposure to gender diversity they receive from peers, schoolmates, popular culture, and other sources. For example, a Pew Research Center study in 2021 indicated that more than 50% of young people in the United States personally know a transgender person (Minkin & Brown, 2021); we know for certain that this would be true of all youths in the *Recruited as Siblings* group and likely most in the *Recruited as Cisgender* group given that gender‐diverse identities appear to be common in young people in the Seattle area (King et al., 2024). Furthermore, representative data suggest that most young people already believe it is possible to identify as neither male nor female, or at least are already familiar with the idea (Geiger & Graf, 2019; Parker et al., 2022).

The second critique presents the possibility of a similar, but in some ways reverse, scenario in the *Recruited as Transgender* group: namely, that youths' transgender identities were cemented across time because they felt pressure to persist in their identity given their participation in an ongoing research study. While we again cannot completely rule out this possibility, we are skeptical of this interpretation. The research team has attempted to be as clear as possible with youths and families that we are interested in their trajectories regardless of whether they persist as transgender or not, and our team's publications have routinely stated the need to support and understand the experiences of early‐identifying transgender youths who retransition to be cisgender (e.g., Durwood et al., 2022; Olson et al., 2022; Olson et al., 2024). Furthermore, some youths in the *Recruited as Transgender* group *have* gone on to identify as cisgender and continued participating; even the small number of youths who have dropped out of the study have often done so with a note about the child's identity at that time allowing us to retain an understanding of their identity. In sum, we find it unlikely that participants' identities over time could have been shaped simply by virtue of participating in our study.

#### VII.3.4 Analytic Strategies and Open Science Practices

Within academic psychology, the past decade and a half has seen a dramatic shift in field‐wide norms relating to methodological rigor, transparency, data‐ and analytic code‐sharing practices, preregistration, and analytic reproducibility, all in service of making empirical research as robust and replicable as possible (e.g., Alessandroni & Byers‐Heinlein, 2022; Kidwell et al., 2016; Nosek et al., 2022; Santoro, 2022). This general goal guided several aspects of our work here. We attempted to adhere to a set of consistent principles across all analyses in the monograph, including attempting to fit all regression models with random effects terms to account for nonindependence introduced by sibling pairs in the data set, simplifying models when the number of observations in any given cell was fewer than 10, and reporting alternative results stemming from different methodological decisions in the Supporting Information.

However, we were not able to implement some best practices for open science in the current work. Preregistering analyses has become increasingly common practice in psychology in the past 15 years because (when performed as intended) it reduces the likelihood of selectively reporting significant results that one has obtained after trying many different versions of an analysis (Eich, 2014; Lakens et al., 2024). Critically, none of our analyses were preregistered; rather, all were exploratory. For many of our analyses, it would not have been possible to prespecify the analytic strategy. As an example, many of our analyses operate on gender‐at‐visit codes of boy, boy‐expansive, gender diverse, girl‐expansive, or girl. This coding scheme and the subsequent analyses using it could not have been preregistered because we settled on this coding scheme after looking at the data and getting a sense of the heterogeneity participants were expressing in their responses. We could not have known whether, for example, anyone would describe their gender as “demi‐girl” or their sexuality as “grey pansexual” or how exactly we would recode those unknowable answers without seeing the raw data. In other words, the analytic strategies we ended up employing throughout the monograph were designed to make sense of features of the data which we could not have known about before doing some preliminary analysis. This is a noteworthy limitation of exploratory work like ours, as it increases “researcher degrees of freedom” (i.e., arbitrary choices made in the research process that could inadvertently impact significance of results; Simmons et al., 2011) and reduces the reliability of effects. We attempted to counteract this limitation by (a) reporting alternative results stemming from different analytic decisions in the Supporting Information, (b) presenting figures in the Supporting Information showing the data in the rawest form possible (while also keeping participants' identities safe) so that readers can see results for themselves when possible, (c) reporting measures of reliability and validity such as interrater reliability when human‐coding was involved, and (d) taking as consistent of an analytic approach as we could across chapters while also balancing the need to choose analytic techniques that were appropriate for various idiosyncratic subsets of the data.

Sharing anonymized versions of data and analytic code has also become increasingly common in psychology because doing so allows other researchers to ensure that reported results are reproducible and maximizes the chance that inadvertent errors in a data processing or analysis pipeline are caught and corrected (Klein et al., 2018; Frank et al., 2025). Despite our team's general tendency to share our data (e.g., Wittlin et al., 2024; Olson et al., 2024; Durwood et al., 2023; Gülgöz et al., 2022), in the current work, we do not. The key reasons are that (a) our data cover highly personal subject matter, including identities and personal details which may or may not be known to others and (b) we collected data within families and thus have unique identifiers in the data which link family members to one another. This second feature is relatively uncommon in lab‐based experimental research in developmental psychology, but common in some areas of social and clinical psychology, particularly those that focus on close relationships like families, friendships, romantic partnerships, and therapy groups (Joel et al., 2018). In these cases, even if data are fully anonymized, the linked nature of the data makes it possible that a participant could, in theory, identify which response within a publicly available record is theirs and then use this information to deduce what a family member said. The problem is compounded in our case because parents and youth were often reporting highly sensitive information—sometimes about each other—that the other did not necessarily know (e.g., a youth could have told us they were LGBTQ before having disclosed this to a parent). Releasing an anonymized version of the data with youth and parents unlinked is a potential solution, but would be generally unhelpful here because so many of our analyses include terms that model nonindependence introduced by sibling and parent/child relationships and because certain combinations of ages or dates might allow results to be connected anyway.

For these reasons, we made the unusual decision (for our team) to not share data for this monograph. Because our data are not available for other researchers to examine, we took additional measures to mitigate the potential impact of this choice and reduce the likelihood of reporting errors. All analyses were conducted in a fully reproducible workflow, and all analytic code used in the data processing and analysis pipeline was subjected to code review by another research group member who had not originally written the script. Additionally, a completely anonymized version of our data analysis scripts is available at https://osf.io/rjfxd/. We hope that in the future, researchers who work with sensitive paired or linked data can devise creative ways to protect the privacy of their participants while also releasing data to the extent possible in the spirit of transparency and rigor.

### VII.4 Future Directions

While we believe the work reported here makes a significant contribution to the field's knowledge about how children and teenagers experience stability and change in their gender identity and sexual orientation over time, there are many intriguing, important, and urgent avenues for future research that could be conducted with this and other cohorts.

#### VII.4.1 Sample Expansion and Investigation of Intersectional Identity Development

First, there is a clear need to examine how gender development—in particular, developmental trajectories in how one thinks of oneself—intersects with other social identities that youth may hold (e.g., racial/ethnic, class, religious, etc.). In the past two decades, psychological research has increasingly emphasized the need to examine social identities from an intersectional perspective (e.g., Cole, 2009; Ghavami et al., 2016; Lei & Rhodes, 2021; Purdie‐Vaughns & Eibach, 2008; Santos & Toomey, 2018), thus highlighting the importance of how various facets of one's identity affect each other (e.g., how one's experience of their race is shaped by being a member of a certain gender category) and how the experiences of people at certain intersections (e.g., a Black, gay, trans youth) may have different experiences than someone who shares one or two of those intersecting categories, but differs on another (e.g., a Black, gay, cisgender youth). Examination of intersectionality is critical in several psychological domains, including person perception [i.e., prototypes of different racial groups tend to be gendered and vice versa in both U.S. children and adults (e.g., Ghavami & Peplau, 2013; Johnson et al., 2012; Lei et al., 2020, 2022; Perszyk et al., 2019; Sesko & Biernat, 2010)] and youth marginalization [i.e., Black and Latino sexual minority adolescents have sometimes been shown to differ in their physical and mental health outcomes compared to white sexual minority adolescents (Burns et al., 2015; Le Vasseur et al., 2013; Poteat et al., 2011; Ryan et al., 2009)].

While research in psychology has examined the impact of social identities like race on adolescents' experiences with gender (Buckley, 2018; Rogers et al., 2015; Skinner & McHale, 2018) and has specifically profiled the experiences of racial and ethnic minority transgender and gender‐diverse youth (Goldenberg et al., 2021; Grossman & d'Augelli, 2008; Reck, 2009; Singh, 2013; Vance et al., 2021), there is much we still do not know about how gender self‐categorization and its development over time are impacted by one's other social identities. Given past research demonstrating that children's and adolescents' person perception and physical/mental health outcomes are impacted by intersecting social identities, there are many important questions that we hope future research will explore. Is it the case, for example, that in racial/ethnic or religious contexts in which felt pressure to conform to gender norms is particularly strong, this might systematically impact the way gender‐diverse youths think about their gender identities over time? If a youth experiences multiple forms of marginalization as a result of holding several minority identities, is it possible that they would be less likely to explore alternative gender presentations due to resource constraints, stress, or other pressures—or more likely given that they are already seen by majority group members as “different”? If, in a youth's family and neighborhood context, gender fluidity is embraced and embodied by many people in the community, but in the school context, there are rigid views of identity, how might this impact the coming‐out process—both to oneself and to others? We are not able to answer these questions with our sample, but we believe they represent a crucial avenue for future researchers that is important to examine in its own right, beyond how answers to these questions bear on the generalizability of our work here.

A related issue is that the gender category labels available to someone are contingent on their social or national context. Participants in this monograph, like most existing work on gender development over time in gender‐diverse youth, are embedded in Western conceptions of gender that are dominant in the United States, western Europe, and Commonwealth countries, meaning that we are not able to contribute to the field's knowledge of how gender self‐categorization develops in any other cultural or national setting. In the past decade, an emerging research literature has begun to document the experiences of gender‐diverse youth (or, in some cases, their parents) in places such as South Africa (Pickstone‐Taylor et al., 2024), Argentina (Mulli et al., 2025), China (Peng et al., 2019), and Japan (Ishii, 2017); other work has described local variants of gender‐diverse identities in youths (or adults recalling their childhoods) in Samoa (Vasey & Bartlett, 2007), indigenous Zapotec areas of southern Mexico (Gómez Jiménez et al., 2020), and Afghanistan (Nordberg, 2015). Despite the growth of this work, there are still many open questions about how basic processes of self‐categorization over time, as we studied here, could be shaped by the category structure present in one's social context.

#### VII.4.2 Continuing Research on Gender‐Diverse Youth in Today's Harsh Sociopolitical Climate

It will be critical to continue studying gender identity over time—in this cohort and others—as the sociopolitical landscape changes. In the United States, the currently‐in‐power Republican party regularly engages in anti‐transgender rhetoric and has enacted legal changes meant to exclude transgender and gender‐diverse people from public life, including banning gender‐affirming medical care for minors, banning the use of federal facilities on the basis of gender identity (and instead requiring the use of sex‐based facilities), replacing gender with sex on passports, and forbidding the acknowledgement of a child's gender identity in school if it does not match their assigned sex. As we have argued here, many of the results we reported in this monograph are likely the products of a cultural landscape that has afforded many youths in our sample more latitude to express gender diversity and queer identity; it will thus be extremely important to examine whether the identities that youths express (publicly and privately) will shift in reaction to a sociopolitical climate that is openly hostile toward gender diversity more broadly. Collecting data from future samples of transgender and gender‐diverse youth who experience childhood and adolescence during the current administration will also be important for understanding the role of structural discrimination (and potentially, limitations on social and medical transition) on the development of gender identity.

#### VII.4.3 Future Research in the Trans Youth Project

The research team responsible for the current work plans to continue following this cohort throughout the next several years (assuming that remains legally and fiscally possible). We hope to continue examining how these youths' identities evolve over time, allowing for an even more direct comparison than we could offer here with well‐known prior literature featuring clinical samples of gender‐ nonconforming children (e.g., Steensma et al., 2013a; Zucker & Bradley, 1995) that sometimes reported on participants' outcomes as young adults (on average, our participants were 14.3 years old at their most recent visit). This will also allow us to assess whether youths in the *Recruited as Cisgender* group who are now transgender or gender diverse show similar or different levels of stability in their identities than we have seen in the early identifying *Recruited as Transgender* youths (who have shown stability in gender in the vast majority of cases). This may be an especially interesting and consequential question given the increasing societal scrutiny and criticism directed toward youth who were ostensibly gender‐conforming in childhood but who transitioned in adolescence (Edwards‐Leeper & Anderson, 2021; Gentleman, 2022; Littman, 2018; Sapir et al., 2024; Shrier, 2020; Terhune et al., 2022; Wadman, 2018). Additionally, as more of the youths in this cohort progress into later stages of adolescence, we will be able to report not only on their expressed sexual and romantic interests but also their experiences (e.g., dating, physical and emotional intimacy, etc.) and how these relate to their earlier reports of sexual orientation.

### VII.5 Conclusion

We have presented a detailed quantitative portrait of gender identity and sexual orientation over time in a sample of over 900 North American youths studied between 2013 and early 2024. Gender identity tended to be a stable trait across development for the vast majority of youths (>80.0%), including those who were supported in a social gender transition during childhood. These early‐identifying transgender youths were no more or less likely to show gender change than their siblings or an unrelated comparison group of youths who were recruited into the study as cisgender children. The gender change we did observe among both early‐identifying transgender and initially cisgender participants is suggestive of a potentially major shift among North American youth in thinking about gender as a concept that is less anchored on binary options and flexible across time. We also observed high rates (30.0%–60.0%) of queer romantic interest among adolescents who self‐report their sexual orientations. Change in romantic interest over time was present in approximately 35% of youths who have reported on sexual orientation longitudinally, and many youths of all genders expressed interest in both boys and girls; these results again point to a substantial shift toward flexibility in thinking about gender and sexuality among today's youth.

We conclude that two things are likely true about gender and sexual orientation in youth today: stability across development is the current modal pathway, regardless of whether one's gender aligns with one's assigned sex or not, *and* youth can and do change how they think about their identities, contrary to a major assumption present in decades of classic research in developmental psychology. We hope these results will not only refine our field's theories about how youth conceptualize the social identities of gender and sexual orientation but also inform broader societal understanding toward, and support of, gender diverse and sexual minority children and adolescents.

### VII.6 Statement on Open Science Practices

Analyses in this manuscript were not preregistered. Due to confidentiality concerns, data is not publicly available. Analytic code is available at https://osf.io/rjfxd/. All analyses were conducted in R version 4.4.1 (R Core Team, 2024) using the following packages: lme4, ggalluvial, funkyheatmap, car, fs, glue, emmeans, here, tidyverse, scales, psych, psychTools, knitr, and kableExtra (D. Bates et al., 2015; Brunson & Read, 2023; Cannoodt & Saelens, 2023; Fox & Weisberg, 2019; Hester et al., 2024; Hester & Bryan, 2024; Lenth, 2024; Müller & Bryan, 2020; Wickham et al., 2019, 2023; William Revelle, 2024a, 2024b; Xie, 2024; Zhu et al., 2024).

## Conflicts of Interest

The authors declare no conflicts of interest.

## Supporting information

Supporting Info.

## References

[mono12479-bib-0284] Achenbach, T. M. (1999). The child behavior checklist and related instruments. In M. E. Maruish (Ed.), The use of psychological testing for treatment planning and outcomes assessment (2nd ed., pp. 429–466). Lawrence Erlbaum Associates Publishers.

[mono12479-bib-0001] Ahlqvist, S. , Halim, M. L. , Greulich, F. K. , Lurye, L. E. , & Ruble, D. (2013). The potential benefits and risks of identifying as a tomboy: A social identity perspective. Self and Identity, 12(5), 563–581. 10.1080/15298868.2012.717709

[mono12479-bib-0002] Alessandroni, N. , & Byers‐Heinlein, K. (2022). Ten strategies to foster open science in psychology and beyond. Collabra: Psychology, 8(1), 57545. 10.1525/collabra.57545

[mono12479-bib-0003] American Psychiatric Association . (1980). Diagnostic and statistical manual of mental disorders: DSM‐3. (3rd ed.). American Psychiatric Association.

[mono12479-bib-0004] Ashley, F. (2022). The clinical irrelevance of “desistance” research for transgender and gender creative youth. Psychology of Sexual Orientation and Gender Diversity, 9(4), 387–397. 10.1037/sgd0000504

[mono12479-bib-0005] Bagley, C. , & Tremblay, P. (1998). On the prevalence of homosexuality and bisexuality, in a random community survey of 750 men aged 18 to 27. Journal of Homosexuality, 36(2), 1–18. 10.1300/J082v36n02_01 9736328

[mono12479-bib-0006] Bailey, J. M. (2003). The man who would be queen: The science of gender‐bending and transsexualism (pp. xiii, 233). Joseph Henry Press.

[mono12479-bib-0007] Bailey, J. M. , Bechtold, K. T. , & Berenbaum, S. A. (2002). Who are tomboys and why should we study them? Archives of Sexual Behavior, 31(4), 333–341. 10.1023/A:1016272209463 12187546

[mono12479-bib-0289] Bailey, J. M. , & Zucker, K. J. (1995). Childhood sex‐typed behavior and sexual orientation: A conceptual analysis and quantitative review. Developmental Psychology, 31(1), 43–55. 10.1037/0012-1649.31.1.43

[mono12479-bib-0008] Bakwin, H. (1960). Transvestism in children. The Journal of Pediatrics, 56(2), 294–298. 10.1016/S0022-3476(60)80128-X 13796068

[mono12479-bib-0009] Bakwin, H. (1968). Deviant gender‐role behavior in children: Relation to homosexuality. Pediatrics, 41(3), 620–629. 10.1542/peds.41.3.620 5641781

[mono12479-bib-0010] Balk, G. (2024a, September 5). Seattle keeps getting more liberal, survey data shows. *The Seattle Times*. https://www.seattletimes.com/seattle-news/data/seattle-keeps-getting-more-liberal-survey-data-shows/

[mono12479-bib-0011] Balk, G. (2024b, September 12). As Seattle household income hits all‐time high, a smaller WA city tops nation's list. *The Seattle Times*. https://www.seattletimes.com/seattle-news/data/seattle-median-household-income-hits-121000-census-data-shows/

[mono12479-bib-0012] Bates, D. , Mächler, M. , Bolker, B. , & Walker, S. (2015). Fitting linear mixed‐effects models using lme4. Journal of Statistical Software, 67(1), 1–48. 10.18637/jss.v067.i01

[mono12479-bib-0013] Bates, J. E. , Skilbeck, W. M. , Smith, K. V. R. , & Bentler, P. M. (1975). Intervention with families of gender‐disturbed boys. American Journal of Orthopsychiatry, 45(1), 150–157. 10.1111/j.1939-0025.1975.tb01176.x 1111297

[mono12479-bib-0014] Bauer, G. R. , Pacaud, D. , Couch, R. , Metzger, D. L. , Gale, L. , Gotovac, S. , Mokashi, A. , Feder, S. , Raiche, J. , Speechley, K. N. , Temple Newhook, J. , Ghosh, S. , Sansfaçon, A. P. , Susset, F. , Lawson, M. L. , & for the Trans Youth CAN! Research Team . (2021). Transgender Youth Referred to Clinics for Gender‐Affirming Medical Care in Canada. Pediatrics, 148(5), e2020047266. 10.1542/peds.2020-047266 34620727

[mono12479-bib-0015] Bazelon, E. (2022, June 15). The battle over gender therapy. *The New York Times Magazine*. https://www.nytimes.com/2022/06/15/magazine/gender-therapy.html

[mono12479-bib-0016] Beitler, P. J. , & Landis, J. R. (1985). A mixed‐effects model for categorical data. Biometrics, 41(4), 991–1000. 10.2307/2530970 3830263

[mono12479-bib-0017] Bem, S. L. (1974). The measurement of psychological androgyny. Journal of Consulting and Clinical Psychology, 42(2), 155–162. 10.1037/h0036215 4823550

[mono12479-bib-0018] Bem, S. L. (1981). Gender schema theory: A cognitive account of sex typing. Psychological Review, 88(4), 354–364. 10.1037/0033-295X.88.4.354

[mono12479-bib-0019] Bem, S. L. , & Lewis, S. A. (1975). Sex role adaptability: One consequence of psychological androgyny. Journal of Personality and Social Psychology, 31(4), 634–643. 10.1037/h0077098

[mono12479-bib-0020] Blanchard, R. (1988). Nonhomosexual gender dysphoria. Journal of Sex Research, 24(1), 188–193. 10.1080/00224498809551410 22375647

[mono12479-bib-0021] Blazina, C. , & Baronavski, C. (2022, September 15). How Americans view policy proposals on transgender and gender identity issues, and where such policies exist. *Pew Research Center*. https://www.pewresearch.org/short-reads/2022/09/15/how-americans-view-policy-proposals-on-transgender-and-gender-identity-issues-and-where-such-policies-exist/

[mono12479-bib-0022] Boskey, E. R. , & Ganor, O. (2022). Sexual orientation and attraction in a cohort of transmasculine adolescents and young adults. Transgender Health, 7(3), 270–275. 10.1089/trgh.2020.0190 36643063 PMC9829161

[mono12479-bib-0023] Bosse, J. D. , & Chiodo, L. (2016). It is complicated: Gender and sexual orientation identity in LGBTQ youth. Journal of Clinical Nursing, 25(23–24), 3665–3675. 10.1111/jocn.13419 27271008

[mono12479-bib-0024] Bowman, N. (2020, February 24). Report: Seattle 9th most liberal metro area in US. *MyNorthwest.Com*. https://mynorthwest.com/1728227/report-seattle-9th-most-liberal-metro-area-in-us/

[mono12479-bib-0025] Bradley, S. J. , & Zucker, K. J. (1990). Gender identity disorder and psychosexual problems in children and adolescents. The Canadian Journal of Psychiatry, 35(6), 477–486. 10.1177/070674379003500603 2207982

[mono12479-bib-0026] Brown, A. (2022, June 7). About 5% of young adults in the U.S. say their gender is different from their sex assigned at birth. *Pew Research Center*. https://www.pewresearch.org/short-reads/2022/06/07/about-5-of-young-adults-in-the-u-s-say-their-gender-is-different-from-their-sex-assigned-at-birth/

[mono12479-bib-0027] Brunson, J. C. , & Read, Q. D. (2023). Ggalluvial: Alluvial Plots in “ggplot2”. http://corybrunson.github.io/ggalluvial/

[mono12479-bib-0028] Buckley, T. R. (2018). Black adolescent males: Intersections among their gender role identity and racial identity and associations with self‐concept (global and school). Child Development, 89(4), e311–e322. 10.1111/cdev.12950 28898392 PMC5847408

[mono12479-bib-0029] Bungener, S. L. , Steensma, T. D. , Cohen‐Kettenis, P. T. , & de Vries, A. L. C. (2017). Sexual and romantic experiences of transgender youth before gender‐affirmative treatment. Pediatrics, 139(3), e20162283. 10.1542/peds.2016-2283 28242863

[mono12479-bib-0030] Burns, A. , Farrell, M. , & Brown, J. C. (1990). Clinical features of patients attending a gender‐identity clinic. The British Journal of Psychiatry, 157(2), 265–268. 10.1192/bjp.157.2.265 2224378

[mono12479-bib-0031] Burns, M. N. , Ryan, D. T. , Garofalo, R. , Newcomb, M. E. , & Mustanski, B. (2015). Mental health disorders in young urban sexual minority men. Journal of Adolescent Health, 56(1), 52–58. 10.1016/j.jadohealth.2014.07.018 PMC4275373 25294230

[mono12479-bib-0032] Cannoodt, R. , & Saelens, W. (2023). Funkyheatmap: Generating funky heatmaps for data frames. Manuscript submitted for publication.

[mono12479-bib-0033] Carver, P. R. , Yunger, J. L. , & Perry, D. G. (2003). Gender identity and adjustment in middle childhood. Sex Roles, 49(3), 95–109. 10.1023/A:1024423012063

[mono12479-bib-0034] Chandra, A. , Copen, C. E. , & Mosher, W. D. (2013). Sexual behavior, sexual attraction, and sexual identity in the United States: Data from the 2006–2010 National Survey of Family Growth. In A. K. Baumle (Ed.), International Handbook on the Demography of Sexuality (pp. 45–66). Springer Netherlands. 10.1007/978-94-007-5512-3_4

[mono12479-bib-0035] Coates, S. , & Person, E. S. (1985). Extreme boyhood femininity: isolated behavior or pervasive disorder? Journal of the American Academy of Child Psychiatry, 24(6), 702–709. 10.1016/S0002-7138(10)60113-6 4067139

[mono12479-bib-0036] Cole, E. R. (2009). Intersectionality and research in psychology. American Psychologist, 64(3), 170–180. 10.1037/a0014564 19348518

[mono12479-bib-0273] Conron, K. J. (2020). LGBT youth population in the United States. Williams Institute. https://williamsinstitute.law.ucla.edu/publications/lgbt-youth-pop-us/

[mono12479-bib-0037] Corby, B. C. , Hodges, E. V. E. , & Perry, D. G. (2007). Gender identity and adjustment in black, Hispanic, and white preadolescents. Developmental Psychology, 43(1), 261–266. 10.1037/0012-1649.43.1.261 17201524

[mono12479-bib-0038] Davenport, C. W. (1986). A follow‐up study of 10 feminine boys. Archives of Sexual Behavior, 15(6), 511–517. 10.1007/BF01542316 3800642

[mono12479-bib-0039] Day, J. K. , Perez‐Brumer, A. , & Russell, S. T. (2018). Safe Schools? Transgender youth's school experiences and perceptions of school climate. Journal of Youth and Adolescence, 47(8), 1731–1742. 10.1007/s10964-018-0866-x 29858740 PMC7153781

[mono12479-bib-0040] deMayo, B. , Kahn‐Samuelson, S. , & Olson, K. R. (2022). Endorsement of gender stereotypes in gender diverse and cisgender adolescents and their parents. PLOS ONE, 17(6), e0269784. 10.1371/journal.pone.0269784 35700172 PMC9197027

[mono12479-bib-0041] deMayo, B. E. , Jordan, A. E. , & Olson, K. R. (2022). Gender development in gender diverse children. Annual Review of Developmental Psychology, 4, 207–229. 10.1146/annurev-devpsych-121020-034014 PMC10457095 37638126

[mono12479-bib-0042] Dessens, A. B. , Slijper, F. M. E. , & Drop, S. L. S. (2005). Gender dysphoria and gender change in chromosomal females with congenital adrenal hyperplasia. Archives of Sexual Behavior, 34(4), 389–397. 10.1007/s10508-005-4338-5 16010462

[mono12479-bib-0043] Diamond, L. M. (2003). Was it a phase? Young women's relinquishment of lesbian/bisexual identities over a 5‐year period. Journal of Personality and Social Psychology, 84(2), 352–364. 10.1037/0022-3514.84.2.352 12585809

[mono12479-bib-0290] Diamond, L. M. (2014). Gender and same‐sex sexuality. In D. L. Tolman , L. M. Diamond , J. A. Bauermeister , W. H. George , J. G. Pfaus , & L. M. Ward (Eds.), *APA handbook of sexuality and psychology*: Person‐based approaches (Vol. 1, pp. 629–652). American Psychological Association.

[mono12479-bib-0044] Diamond, L. M. (2016). Sexual fluidity in male and females. Current Sexual Health Reports, 8(4), 249–256. 10.1007/s11930-016-0092-z

[mono12479-bib-0045] Dickson, N. , Paul, C. , & Herbison, P. (2003). Same‐sex attraction in a birth cohort: Prevalence and persistence in early adulthood. Social Science & Medicine, 56(8), 1607–1615. 10.1016/S0277-9536(02)00161-2 12639578

[mono12479-bib-0046] Dickson, N. , van Roode, T. , Cameron, C. , & Paul, C. (2013). Stability and change in same‐sex attraction, experience, and identity by sex and age in a New Zealand birth cohort. Archives of Sexual Behavior, 42(5), 753–763. 10.1007/s10508-012-0063-z 23430085

[mono12479-bib-0047] Doorn, C. D. , Poortinga, J. , & Verschoor, A. M. (1994). Cross‐gender identity in transvestites and male transsexuals. Archives of Sexual Behavior, 23(2), 185–201. 10.1007/BF01542098 8018022

[mono12479-bib-0277] Drummond, K. D. , Bradley, S. J. , Peterson‐Badali, M. , & Zucker, K. J. (2008). A follow‐up study of girls with gender identity disorder. Developmental Psychology, 44(1), 34–45. 10.1037/0012-1649.44.1.34 18194003

[mono12479-bib-0279] Durwood, L. , Gallagher, N. M. , Sifre, R. , & Olson, K. R. (2023). A study of parent‐reported internalizing symptoms in transgender youths before and after childhood social transitions. Clinical Psychological Science, 12(5), 984–996. 10.1177/21677026231208086 39474313 PMC11521117

[mono12479-bib-0278] Durwood, L. , Kuvalanka, K. A. , Kahn‐Samuelson, S. , Jordan, A. E. , Rubin, J. D. , Schnelzer, P. , Devor, A. H. , & Olson, K. R. (2022). Retransitioning: The experiences of youth who socially transition genders more than once. International Journal of Transgender Health, 23(4), 409–427. 10.1080/26895269.2022.2085224 36324883 PMC9621273

[mono12479-bib-0048] Edwards‐Leeper, L. , & Anderson, E. (2021, November 24). Perspective | The mental health establishment is failing trans kids. *Washington Post*. https://www.washingtonpost.com/outlook/2021/11/24/trans-kids-therapy-psychologist/

[mono12479-bib-0049] Egan, S. K. , & Perry, D. G. (2001). Gender identity: A multidimensional analysis with implications for psychosocial adjustment. Developmental Psychology, 37(4), 451–463. 10.1037/0012-1649.37.4.451 11444482

[mono12479-bib-0050] Eich, E. (2014). Business not as usual. Psychological Science, 25(1), 3–6. 10.1177/0956797613512465 24285431

[mono12479-bib-0051] Eisenberg, M. E. , Gower, A. L. , McMorris, B. J. , Rider, G. N. , Shea, G. , & Coleman, E. (2017). Risk and protective factors in the lives of transgender/gender nonconforming adolescents. Journal of Adolescent Health, 61(4), 521–526. 10.1016/j.jadohealth.2017.04.014 PMC5626022 28736148

[mono12479-bib-0052] Etaugh, C. , Grinnell, K. , & Etaugh, A. (1989). Development of gender labeling: Effect of age of pictured children. Sex Roles, 21(11), 769–773. 10.1007/BF00289807

[mono12479-bib-0053] Fagot, B. I. (1985). Changes in thinking about early sex role development. Developmental Review, 5(1), 83–98. 10.1016/0273-2297(85)90031-0

[mono12479-bib-0054] Fagot, B. I. , & Leinbach, M. D. (1993). Gender‐role development in young children: From discrimination to labeling. Developmental Review, 13(2), 205–224. 10.1006/drev.1993.1009

[mono12479-bib-0055] Fagot, B. I. , Leinbach, M. D. , & Hagan, R. (1986). Gender labeling and the adoption of sex‐typed behaviors. Developmental Psychology, 22(4), 440–443. 10.1037/0012-1649.22.4.440

[mono12479-bib-0291] Flores, A. R. & Conron, K. J. (2023). Adult LGBT population in the United States. Williams Institute. https://williamsinstitute.law.ucla.edu/publications/adult-lgbt-pop-us/

[mono12479-bib-0056] Flores, A. R. , Herman, J. L. , Gates, G. J. , & Brown, T. N. T. (2016, June). How many adults identify as transgender in the United States? *Williams Institute*. https://williamsinstitute.law.ucla.edu/wp-content/uploads/Trans-Adults-US-Aug-2016.pdf

[mono12479-bib-0057] Fox, J. , & Weisberg, S. (2019). An R companion to applied regression (3rd ed.). Sage. https://www.john-fox.ca/Companion/

[mono12479-bib-0058] Frank, M. C. , Braginsky, M. , Cachia, J. , Coles, N. A. , Hardwicke, T. E. , Hawkins, R. D. , Mathur, M. B. , & Williams, R. 2025. Experimentology: An open science approach to experimental psychology methods. Stanford University. 10.25936/3JP6-5M50

[mono12479-bib-0059] Friend, M. R. , Schiddel, L. , Klein, B. , & Dunaeff, D. (1954). Observations on the development of transvestitism in boys. American Journal of Orthopsychiatry, 24(3), 563–575. 10.1111/j.1939-0025.1954.tb06128.x 13180676

[mono12479-bib-0060] Gallagher, N. M. , Foster‐Hanson, E. , & Olson, K. R. (2025). Gender categorization and memory in transgender and cisgender people. Journal of Experimental Social Psychology, 116, 104691. 10.1016/j.jesp.2024.104691 40046167 PMC11879278

[mono12479-bib-0061] Gallup . (2024). *LGBTQ+ rights*. Gallup. https://news.gallup.com/poll/1651/Gay-Lesbian-Rights.aspx

[mono12479-bib-0062] Gates, G. J. (2014). LGBT demographics: Comparisons among population‐based surveys. *Williams Institute*. https://escholarship.org/uc/item/0Kr784fx

[mono12479-bib-0063] Geiger, A. W. , & Graf, N. (2019, September 5). About one‐in‐five U.S. adults know someone who goes by a gender‐neutral pronoun. *Pew Research Center*. https://www.pewresearch.org/short-reads/2019/09/05/gender-neutral-pronouns/

[mono12479-bib-0064] Gentleman, A. (2022, November 24). ‘An explosion’: What is behind the rise in girls questioning their gender identity? *The Guardian*. https://www.theguardian.com/society/2022/nov/24/an-explosion-what-is-behind-the-rise-in-girls-questioning-their-gender-identity

[mono12479-bib-0065] Ghavami, N. , & Peplau, L. A. (2013). An intersectional analysis of gender and ethnic stereotypes: Testing three hypotheses. Psychology of Women Quarterly, 37(1), 113–127. 10.1177/0361684312464203

[mono12479-bib-0066] Ghavami, N. , Katsiaficas, D. , & Rogers, L. O. (2016). Chapter Two—Toward an Intersectional Approach in Developmental Science: The Role of Race, Gender, Sexual Orientation, and Immigrant Status. In S. S. Horn , M. D. Ruck , & L. S. Liben (Eds.), Advances in Child Development and Behavior (Vol. 50, pp. 31–73). JAI. 10.1016/bs.acdb.2015.12.001 26956069

[mono12479-bib-0067] Ghorayshi, A. (2022, May 4). Few transgender children change their minds after 5 years, study finds. *The New York Times*. https://www.nytimes.com/2022/05/04/health/transgender-children-identity.html

[mono12479-bib-0068] Gill‐Peterson, J. (2018). Histories of the transgender child. University of Minnesota Press. 10.5749/j.ctv75d87g

[mono12479-bib-0069] GLAAD . (2015, September 16). Number of Americans who report knowing a transgender person doubles in seven years, according to new GLAAD survey. *GLAAD*. https://glaad.org/releases/number-americans-who-report-knowing-transgender-person-doubles-seven-years-according-new/

[mono12479-bib-0070] GLAAD . (2023, April 19). The New York Times' inaccurate coverage of transgender people is being weaponized against the transgender community | GLAAD. *GLAAD*. https://glaad.org/new-york-times-inaccurate-coverage-transgender-people-being-weaponized-against-transgender/

[mono12479-bib-0071] Goldenberg, T. , Gamarel, K. E. , Reisner, S. L. , Jadwin‐Cakmak, L. , & Harper, G. W. (2021). Gender affirmation as a source of resilience for addressing stigmatizing healthcare experiences of transgender youth of color. Annals of Behavioral Medicine, 55(12), 1168–1183. 10.1093/abm/kaab011 33761531 PMC8601040

[mono12479-bib-0072] Golombok, S. , Rust, J. , Zervoulis, K. , Croudace, T. , Golding, J. , & Hines, M. (2008). Developmental trajectories of sex‐typed behavior in boys and girls: A longitudinal general population study of children aged 2.5–8 years. Child Development, 79(5), 1583–1593. 10.1111/j.1467-8624.2008.01207.x 18826544

[mono12479-bib-0073] Gómez Jiménez, F. R. , Court, L. , & Vasey, P. L. (2020). A retrospective study of childhood sex‐typed behavior in Istmo Zapotec men, women, and muxes. Archives of Sexual Behavior, 49(2), 467–477. 10.1007/s10508-019-01544-6 31529223

[mono12479-bib-0074] Gonsiorek, J. C. , Sell, R. L. , & Weinrich, J. D. (1995). Definition and measurement of sexual orientation. Suicide and Life‐Threatening Behavior, 25(s1), 40–51. 10.1111/j.1943-278X.1995.tb00489.x 8553428

[mono12479-bib-0075] Gower, A. L. , Rider, G. N. , Brown, C. , & Eisenberg, M. E. (2022). Diverse sexual and gender identity, bullying, and depression among adolescents. Pediatrics, 149(4), e2021053000. 10.1542/peds.2021-053000 35307739 PMC9647869

[mono12479-bib-0076] Green, R. (1974). Sexual identity conflict in children and adults. Basic Books.

[mono12479-bib-0077] Green, R. (1987). The “sissy boy syndrome” and the development of homosexuality. Yale University Press.

[mono12479-bib-0078] Green, R. (2017). To transition or not to transition? That is the question. Current Sexual Health Reports, 9(2), 79–83. 10.1007/s11930-017-0106-5

[mono12479-bib-0079] Green, R. , & Money, J. (1960). Incongruous gender role: Nongenital manifestations in prepubertal boys. The Journal of Nervous and Mental Disease, 131(2), 160. 13708206

[mono12479-bib-0080] Green, R. , & Money, J. (1961). Effeminacy in prepubertal boys: Summary of eleven cases and recommendations for case management. Pediatrics, 27(2), 286–291. 10.1542/peds.27.2.286 13708205

[mono12479-bib-0081] Green, R. , Williams, K. , & Goodman, M. (1982). Ninety‐nine “tomboys” and “non‐tomboys”: Behavioral contrasts and demographic similarities. Archives of Sexual Behavior, 11(3), 247–266. 10.1007/BF01544993 7138299

[mono12479-bib-0082] Greenberg, D. , Najle, M. , Jackson, N. , Bola, O. , & Jones, R. P. (2019, June 11). America's Growing Support for Transgender Rights. *PRRI*. https://www.prri.org/research/americas-growing-support-for-transgender-rights/

[mono12479-bib-0083] Grossman, A. H. , & D'augelli, A. R. (2006). Transgender youth: Invisible and vulnerable. Journal of Homosexuality, 51(1), 111–128. 10.1300/J082v51n01_06 16893828

[mono12479-bib-0084] Gubbala, S. , Poushter, J. , & Huang, C. (2023, November 27). How people around the world view same‐sex marriage. *Pew Research Center*. https://www.pewresearch.org/short-reads/2023/11/27/how-people-around-the-world-view-same-sex-marriage/

[mono12479-bib-0085] Gülgöz, S. , Edwards, D. L. , & Olson, K. R. (2022). Between a boy and a girl: Measuring gender identity on a continuum. Social Development, 31(3), 916–929. 10.1111/sode.12587 37637193 PMC10457018

[mono12479-bib-0086] Gülgöz, S. , Glazier, J. J. , Enright, E. A. , Alonso, D. J. , Durwood, L. J. , Fast, A. A. , Lowe, R. , Ji, C. , Heer, J. , Martin, C. L. , & Olson, K. R. (2019). Similarity in transgender and cisgender children's gender development. Proceedings of the National Academy of Sciences, 116(49), 24480–24485. 10.1073/pnas.1909367116 PMC6900519 31740598

[mono12479-bib-0087] Guss, C. , Shumer, D. , & Katz‐Wise, S. L. (2015). Transgender and gender nonconforming adolescent care: Psychosocial and medical considerations. Current Opinion in Pediatrics, 27(4), 421. 10.1097/MOP.0000000000000240 26087416 PMC4522917

[mono12479-bib-0088] Halim, M. L. , Dalmut, E. , Greulich, F. K. , Ahlqvist, S. , Lurye, L. E. , & Ruble, D. N. (2011). The role of athletics in the self‐esteem of tomboys. Child Development Research, 2011, 1–9.

[mono12479-bib-0089] Halim, M. L. , & Ruble, D. (2010). Gender identity and stereotyping in early and middle childhood. In J. C. Chrisler & D. R. McCreary (Eds.), Handbook of Gender Research in Psychology: Volume 1: Gender Research in General and Experimental Psychology (pp. 495–525). Springer. 10.1007/978-1-4419-1465-1_24

[mono12479-bib-0090] Halim, M. L. , Ruble, D. , Tamis‐LeMonda, C. , & Shrout, P. E. (2013). Rigidity in gender‐typed behaviors in early childhood: A longitudinal study of ethnic minority children. Child Development, 84(4), 1269–1284. 10.1111/cdev.12057 23432471 PMC4754127

[mono12479-bib-0091] Hennessy‐Fiske, M. (2023, December 6). ‘Detransitioners’ wield influence in shaping conservative transgender laws. *Washington Post*. https://www.washingtonpost.com/nation/2023/12/06/detransitioners-transgender-care-laws/

[mono12479-bib-0092] Herdt, G. , & McClintock, M. (2000). The magical age of 10. Archives of Sexual Behavior, 29(6), 587–606. 10.1023/A:1002006521067 11100264

[mono12479-bib-0093] Herman, J. L. , Flores, A. R. , Brown, T. N. T. , Wilson, B. D. M. , & Conron, K. J. (2017, January). Age of Individuals Who Identify as Transgender in the United States. *Williams Institerute*. https://williamsinstitute.law.ucla.edu/wp-content/uploads/Age-Trans-Individuals-Jan-2017.pdf

[mono12479-bib-0094] Herman, J. L. , Flores, A. R. , & O'Neill, K. K. (2022, June). *How Many Adults and Youth Identify as Transgender in the United States?* Williams Institute. https://williamsinstitute.law.ucla.edu/publications/trans-adults-united-states/

[mono12479-bib-0095] Hester, J. , & Bryan, J. (2024). Glue: Interpreted String Literals. *glue*. https://glue.tidyverse.org/

[mono12479-bib-0096] Hester, J. , Wickham, H. , & Csárdi, G. (2024). Fs: Cross‐Platform File System Operations Based on “libuv”. *fs*. https://fs.r-lib.org

[mono12479-bib-0097] Hines, M. (2009). Gonadal hormones and sexual differentiation of human brain and behavior. In R. T. Rubin & D. W. Pfaff (Eds.), Hormone/behavior relations of clinical importance: Endocrine systems interacting with brain and behavior (pp. 207–247). Elsevier Academic Press.

[mono12479-bib-0098] Hyde, J. S. , Bigler, R. S. , Joel, D. , Tate, C. C. , & van Anders, S. M. (2019). The future of sex and gender in psychology: Five challenges to the gender binary. American Psychologist, 74(2), 171–193. 10.1037/amp0000307 30024214

[mono12479-bib-0099] Iervolino, A. C. , Hines, M. , Golombok, S. E. , Rust, J. , & Plomin, R. (2005). Genetic and environmental influences on sex‐typed behavior during the preschool years. Child Development, 76(4), 826–840. 10.1111/j.1467-8624.2005.00880.x 16026499

[mono12479-bib-0100] Ipsos . (2018, January 29). Global attitudes toward transgender people | Ipsos. *Ipsos*. https://www.ipsos.com/en-us/news-polls/global-attitudes-toward-transgender-people

[mono12479-bib-0101] Ishii, Y. (2017). Rebuilding relationships in a transgender family: The stories of parents of Japanese transgender children. Journal of GLBT Family Studies, 14(3), 213–237. 10.1080/1550428X.2017.1326015

[mono12479-bib-0102] Johnson, K. L. , Freeman, J. B. , & Pauker, K. (2012). Race is gendered: How covarying phenotypes and stereotypes bias sex categorization. Journal of Personality and Social Psychology, 102(1), 116–131. 10.1037/a0025335 21875229

[mono12479-bib-0103] Johnson, L. L. , Bradley, S. J. , Birkenfeld‐Adams, A. S. , Kuksis, M. A. R. , Maing, D. M. , Mitchell, J. N. , & Zucker, K. J. (2004). A parent‐report gender identity questionnaire for children. Archives of Sexual Behavior, 33(2), 105–116. 10.1023/B:ASEB.0000014325.68094.f3 15146143

[mono12479-bib-0105] Jones, J. M. (2024, March 13). LGBTQ+ Identification in U.S. Now at 7.6%. *Gallup*. https://news.gallup.com/poll/611864/lgbtq-identification.aspx

[mono12479-bib-0104] Jones, J. M. (2025, February 20). LGBTQ+ Identification in U.S. Rises to 9.3%. *Gallup*. https://news.gallup.com/poll/656708/lgbtq-identification-rises.aspx

[mono12479-bib-0106] Kahn, N. F. , Sequeira, G. M. , Asante, P. G. , Kidd, K. M. , Coker, T. R. , Christakis, D. A. , Karrington, B. , Aye, T. , Conard, L. A. E. , Dowshen, N. , Kazak, A. E. , Nahata, L. , Nokoff, N. J. , Voss, R. V. , & Richardson, L. P. (2024). Estimating transgender and gender‐diverse youth populations in health systems and survey data. Pediatrics, 153(6), e2023065197. 10.1542/peds.2023-065197 38752289

[mono12479-bib-0107] Katz‐Wise, S. L. (2015). Sexual fluidity in young adult women and men: Associations with sexual orientation and sexual identity development. Psychology & Sexuality, 6(2), 189–208. 10.1080/19419899.2013.876445

[mono12479-bib-0108] Katz‐Wise, S. L. , & Todd, K. P. (2022). The current state of sexual fluidity research. Current Opinion in Psychology, 48, 101497. 10.1016/j.copsyc.2022.101497 36401908 PMC10289116

[mono12479-bib-0109] Katz‐Wise, S. L. , Perry, N. S. , Nelson, K. M. , Gordon, A. R. , & Ybarra, M. L. (2023a). Sexual fluidity in identity and behavior among cisgender youth: findings from the longitudinal growing up with media study. The Journal of Pediatrics, 257, 113355. 10.1016/j.jpeds.2023.01.020 36822509 PMC10293031

[mono12479-bib-0110] Katz‐Wise, S. L. , Ranker, L. R. , Gordon, A. R. , Xuan, Z. , & Nelson, K. (2023b). Sociodemographic patterns in retrospective sexual orientation identity and attraction change in the sexual orientation fluidity in youth study. Journal of Adolescent Health, 72(3), 437–443. 10.1016/j.jadohealth.2022.10.015 PMC10289118 36528519

[mono12479-bib-0111] Katz‐Wise, S. L. , Ranker, L. R. , Kraus, A. D. , Wang, Y.‐C. , Xuan, Z. , Green, J. G. , & Holt, M. (2024). Fluidity in gender identity and sexual orientation identity in transgender and nonbinary youth. The Journal of Sex Research, 61(9), 1367–1376. 10.1080/00224499.2023.2244926 37585555

[mono12479-bib-0112] Katz‐Wise, S. L. , Reisner, S. L. , Hughto, J. W. , & St. Amand, C. (2016). Differences in sexual orientation diversity and sexual fluidity in attractions among gender minority adults in Massachusetts. The Journal of Sex Research, 53(1), 74–84. 10.1080/00224499.2014.1003028 26156113 PMC4685005

[mono12479-bib-0113] Katz‐Wise, S. L. , Rosario, M. , Calzo, J. P. , Scherer, E. A. , Sarda, V. , & Austin, S. B. (2017). Endorsement and timing of sexual orientation developmental milestones among sexual minority young adults in the growing up today study. The Journal of Sex Research, 54(2), 172–185. 10.1080/00224499.2016.1170757 27148762 PMC5607625

[mono12479-bib-0114] Keo‐Meier, C. , & Ehrensaft, D. (Eds.). (2018). Introduction to the gender affirmative model. In The gender affirmative model: An interdisciplinary approach to supporting transgender and gender expansive children (pp. 3–19). American Psychological Association. 10.1037/0000095-001

[mono12479-bib-0115] van Kesteren, P. J. , Gooren, L. J. , & Megens, J. A. (1996). An epidemiological and demographic study of transsexuals in the Netherlands. Archives of Sexual Behavior, 25(6), 589–600. 10.1007/BF02437841 8931882

[mono12479-bib-0116] Kidd, K. M. , Sequeira, G. M. , Douglas, C. , Paglisotti, T. , Inwards‐Breland, D. J. , Miller, E. , & Coulter, R. W. S. (2021). Prevalence of gender‐diverse youth in an urban school district. Pediatrics, 147(6), e2020049823. 10.1542/peds.2020-049823 34006616 PMC8168604

[mono12479-bib-0117] Kidwell, M. C. , Lazarević, L. B. , Baranski, E. , Hardwicke, T. E. , Piechowski, S. , Falkenberg, L.‐S. , Kennett, C. , Slowik, A. , Sonnleitner, C. , Hess‐Holden, C. , Errington, T. M. , Fiedler, S. , & Nosek, B. A. (2016). Badges to acknowledge open practices: A simple, low‐cost, effective method for increasing transparency. PLOS Biology, 14(5), e1002456. 10.1371/journal.pbio.1002456 27171007 PMC4865119

[mono12479-bib-0118] King, K. M. , Feil, M. C. , Gomez Juarez, N. , Moss, D. , Halvorson, M. A. , Dora, J. , Upton, N. F. , Bryson, M. A. , Seldin, K. , Shoda, Y. , Lee, C. M. , & Smith, G. T. (2024). Negative urgency as a state‐level process. Journal of Personality, 93(3), 529–552. https://doi.org/1.1111/jopy.129610. 10.1111/jopy.12961 39015055 PMC11739430

[mono12479-bib-0119] Kinsey Institute . (2011). Diversity of sexual orientation. *Kinsey Institute at Indiana University*. https://kinseyinstitute.org/research/publications/historical-report-diversity-of-sexual-orientation.php

[mono12479-bib-0120] Klein, O. , Hardwicke, T. E. , Aust, F. , Breuer, J. , Danielsson, H. , Mohr, A. H. , Ijzerman, H. , Nilsonne, G. , Vanpaemel, W. , & Frank, M. C. (2018). A practical guide for transparency in psychological science. Collabra: Psychology, 4(1), 20. 10.1525/collabra.158

[mono12479-bib-0122] Kohlberg, L. (1966). A cognitive‐developmental analysis of children's sex‐role concepts and attitudes. In E. Maccoby (Ed.), The Development of Sex Differences (pp. 82–173). Stanford University Press.

[mono12479-bib-0123] Kosky, R. J. (1987). Gender‐disordered children: Does inpatient treatment help? Medical Journal of Australia, 146(11), 565–569. 10.5694/j.1326-5377.1987.tb120415.x 3614045

[mono12479-bib-0124] Kuper, L. E. , Lindley, L. , & Lopez, X. (2019). Exploring the gender development histories of children and adolescents presenting for gender affirming medical care. Clinical Practice in Pediatric Psychology, 7(3), 217–228. 10.1037/cpp0000290

[mono12479-bib-0125] Kuvalanka, K. A. , Weiner, J. L. , & Mahan, D. (2014). Child, family, and community transformations: findings from interviews with mothers of transgender girls. Journal of GLBT Family Studies, 10(4), 354–379. https://www.tandfonline.com/doi/abs/10.1080/1550428X.2013.834529

[mono12479-bib-0126] Lakens, D. , Mesquida, C. , Rasti, S. , & Ditroilo, M. (2024). The benefits of preregistration and Registered Reports. Evidence‐Based Toxicology, 2(1), 2376046. 10.1080/2833373X.2024.2376046

[mono12479-bib-0128] Lawrence, A. A. (2010). Sexual orientation versus age of onset as bases for typologies (subtypes) for gender identity disorder in adolescents and adults. Archives of Sexual Behavior, 39(2), 514–545. 10.1007/s10508-009-9594-3 20140487

[mono12479-bib-0129] Lebovitz, P. S. (1972). Feminine behavior in boys: Aspects of its outcome. American Journal of Psychiatry, 128(10), 1283–1289. 10.1176/ajp.128.10.1283 4111167

[mono12479-bib-0292] Lei, R. F. , Leshin, R. A. , Moty, K. , Foster‐Hanson, E. , & Rhodes, M. (2022). How race and gender shape the development of social prototypes in the United States. Journal of Experimental Psychology: General, 151(8), 1956–1971. 10.1037/xge0001164 34941345 PMC9413299

[mono12479-bib-0131] Lei, R. F. , Leshin, R. A. , & Rhodes, M. (2020). The development of intersectional social prototypes. Psychological Science, 31(8), 911–926. 10.1177/0956797620920360 32501742 PMC7492722

[mono12479-bib-0130] Lei, R. F. , & Rhodes, M. (2021). Why developmental research on social categorization needs intersectionality. Child Development Perspectives, 15(3), 143–147. 10.1111/cdep.12421

[mono12479-bib-0132] Leinbach, M. D. , & Fagot, B. I. (1986). Acquisition of gender labels: A test for toddlers. Sex Roles, 15(11), 655–666. 10.1007/BF00288221

[mono12479-bib-0133] Leinbach, M. D. , & Fagot, B. I. (1993). Categorical habituation to male and female faces: Gender schematic processing in infancy. Infant Behavior and Development, 16(3), 317–332. 10.1016/0163-6383(93)80038-A

[mono12479-bib-0134] Lenth, R. V. (2024). Emmeans: Estimated marginal means, aka least‐squares means. R package version. https://rvlenth.github.io/emmeans/

[mono12479-bib-0135] LeVasseur, M. T. , Kelvin, E. A. , & Grosskopf, N. A. (2013). Intersecting identities and the association between bullying and suicide attempt among New York City youths: Results from the 2009 New York City youth risk behavior survey. American Journal of Public Health, 103(6), 1082–1089. 10.2105/AJPH.2012.300994 23597376 PMC3698714

[mono12479-bib-0136] Li, G. , Kung, K. T. F. , & Hines, M. (2017). Childhood gender‐typed behavior and adolescent sexual orientation: A longitudinal population‐based study. Developmental Psychology, 53(4), 764–777. 10.1037/dev0000281 28221049

[mono12479-bib-0137] Littman, L. (2018). Parent reports of adolescents and young adults perceived to show signs of a rapid onset of gender dysphoria. PLOS ONE, 13(8), e0202330. 10.1371/journal.pone.0202330 30114286 PMC6095578

[mono12479-bib-0138] van der Loos, M. A. T. C. , Klink, D. T. , Hannema, S. E. , Bruinsma, S. , Steensma, T. D. , Kreukels, B. P. C. , Cohen‐Kettenis, P. T. , de Vries, A. L. C. , den Heijer, M. , & Wiepjes, C. M. (2023). Children and adolescents in the Amsterdam Cohort of Gender Dysphoria: Trends in diagnostic‐ and treatment trajectories during the first 20 years of the Dutch Protocol. The Journal of Sexual Medicine, 20(3), 398–409. 10.1093/jsxmed/qdac029 36763938

[mono12479-bib-0139] Luke, S. G. (2017). Evaluating significance in linear mixed‐effects models in R. Behavior Research Methods, 49(4), 1494–1502. 10.3758/s13428-016-0809-y 27620283

[mono12479-bib-0140] Maccoby, E. E. , & Jacklin, C. N. (1987). Gender segregation in childhood. In H. W. Reese (Ed.), Advances in child development and behavior (Vol. 20, pp. 239–287). JAI. 10.1016/S0065-2407(08)60404-8 3630812

[mono12479-bib-0141] Maguen, S. , Floyd, F. J. , Bakeman, R. , & Armistead, L. (2002). Developmental milestones and disclosure of sexual orientation among gay, lesbian, and bisexual youths. Journal of Applied Developmental Psychology, 23(2), 219–233. 10.1016/S0193-3973(02)00105-3

[mono12479-bib-0142] Martin, C. L. , & Dinella, L. M. (2012). Congruence between gender stereotypes and activity preference in self‐identified tomboys and non‐tomboys. Archives of Sexual Behavior, 41(3), 599–610. 10.1007/s10508-011-9786-5 21755383

[mono12479-bib-0143] Martin, C. L. , & Fabes, R. A. (2001). The stability and consequences of young children's same‐sex peer interactions. Developmental Psychology, 37(3), 431–446. 10.1037/0012-1649.37.3.431 11370917

[mono12479-bib-0144] Martin, C. L. , Andrews, N. C. Z. , England, D. E. , Zosuls, K. , & Ruble, D. N. (2017). A dual identity approach for conceptualizing and measuring children's gender identity. Child Development, 88(1), 167–182. 10.1111/cdev.12568 27246654

[mono12479-bib-0145] Masala, B. , Love, A. , Carmichael, P. , & Masic, U. (2024). Demographics of referrals to a specialist gender identity service in the UK between 2017 and 2020. Clinical Child Psychology and Psychiatry, 29(2), 624–636. 10.1177/13591045231202372 37698232

[mono12479-bib-0146] McKechnie, D. G. J. , O'Nions, E. , Bailey, J. , Hobbs, L. , Gillespie, F. , & Petersen, I. (2023). Transgender identity in young people and adults recorded in UK primary care electronic patient records: Retrospective, dynamic, cohort study. BMJ Medicine, 2(1), e000499. 10.1136/bmjmed-2023-000499 38034075 PMC10685922

[mono12479-bib-0147] Meadow, T. (2011). ‘Deep down where the music plays’: How parents account for childhood gender variance. Sexualities, 14(6), 725–747. 10.1177/1363460711420463

[mono12479-bib-0148] Meerwijk, E. L. , & Sevelius, J. M. (2017). Transgender population size in the United States: A meta‐regression of population‐based probability samples. American Journal of Public Health, 107(2), e1–e8. 10.2105/AJPH.2016.303578 PMC5227946 28075632

[mono12479-bib-0149] Miller, C. C. , & Paris, F. (2025, February 20). Nearly one in 10 U.S. adults identifies as L.G.B.T.Q., survey finds. *The New York Times*. https://www.nytimes.com/2025/02/20/upshot/lgbtq-survey-results.html

[mono12479-bib-0150] Minkin, R. , & Brown, A. (2021, July 27). Rising shares of U.S. adults know someone who is transgender or goes by gender‐neutral pronouns. *Pew Research Center*. https://www.pewresearch.org/short-reads/2021/07/27/rising-shares-of-u-s-adults-know-someone-who-is-transgender-or-goes-by-gender-neutral-pronouns/

[mono12479-bib-0151] Minnesota Department of Education . (2022). Minnesota student survey reports 2013–2022. *Minnesota Department of Education Data Center*. https://public.education.mn.gov/MDEAnalytics/DataTopic.jsp?TOPICID=242

[mono12479-bib-0152] Mintel . (2020, December 10). 46% of Americans view gender as a spectrum. *Mintel*. https://www.mintel.com/press-centre/gender-spectrum-mainstreams-46-of-americans-view-gender-as-a-spectrum/

[mono12479-bib-0286] Mock, S. E. , & Eibach, R. P. (2012). Stability and change in sexual orientation identity over a 10‐year period in adulthood. Archives of Sexual Behavior, 41(3), 641–648. 10.1007/s10508-011-9761-1 21584828

[mono12479-bib-0153] Money, J. , & Russo, A. J. (1979). Homosexual outcome of discordant gender identity/role in childhood: Longitudinal follow‐up1. Journal of Pediatric Psychology, 4(1), 29–41. 10.1093/jpepsy/4.1.29

[mono12479-bib-0154] Mosher, W. D. , Chandra, A. , & Jones, J. (2005). Sexual behavior and selected health measures: Men and women 15–44 years of age, United States, 2002. *U.S. Department of Health and Human Services, Centers for Disease Control and Prevention, National Center for Health Statistics*.

[mono12479-bib-0274] Mpofu, J. J. , Underwood, J. M. , Thornton, J. E. , Brener, N. D. , Rico, A. , Kilmer, G. , Harris, W. A. , Leon‐Nguyen, M. , Chyen, D. , Lim, C. , Mbaka, C. K. , Smith‐Grant, J. , Whittle, L. , Jones, S. E. , Krause, K. H. , Li, J. , Shanklin, S. L. , McKinnon, I. , Arrey, L. , … Roberts, A. M. (2023). Overview and methods for the youth risk behavior surveillance system — United States, 2021. MMWR Supplements, 72(1), 1–12. 10.15585/mmwr.su7201a1 37104281 PMC10156160

[mono12479-bib-0155] Müller, K. , & Bryan, J. (2020). *here: A Simpler Way to Find Your Files* (Version 1.0.1) [Computer software]. https://cran.r-project.org/web/packages/here/index.html

[mono12479-bib-0156] Mulli, V. , Zabalza, M. , Eymann, A. , Alonso, G. , Bellomo, M. M. , Bertini, M. C. , Kuspiel, M. F. , Ormaechea, M. N. , Catsicaris, C. , & Busaniche, J. (2025). Características de niños, niñas y adolescentes trans y no binarios atendidos en un hospital de tercer nivel. Archivos Argentinos de Pediatría, 123(1), e202410359. https://www.sap.org.ar/docs/publicaciones/archivosarg/2025/v123n1a15.pdf 39270068 10.5546/aap.2024-10359.eng

[mono12479-bib-0157] Mustanski, B. , Kuper, L. , & Greene, G. J. (2014). Development of sexual orientation and identity. In D. L. Tolman , L. M. Diamond , J. A. Bauermeister , W. H. George , J. G. Pfaus , & L. M. Ward (Eds.), APA handbook of sexuality and psychology, Vol. 1: Person‐based approaches (pp. 597–628). American Psychological Association. 10.1037/14193-019

[mono12479-bib-0158] Nieder, T. O. , Herff, M. , Cerwenka, S. , Preuss, W. F. , Cohen‐Kettenis, P. T. , De Cuypere, G. , Hebold Haraldsen, I. R. , & Richter‐Appelt, H. (2011). Age of onset and sexual orientation in transsexual males and females. The Journal of Sexual Medicine, 8(3), 783–791. 10.1111/j.1743-6109.2010.02142.x 21143416

[mono12479-bib-0159] NORC . (2022). Key trends. *GSS Data Explorer*. https://gssdataexplorer.norc.org/trends

[mono12479-bib-0160] Nordberg, J. (2015). The underground girls of Kabul: In search of a hidden resistance in Afghanistan. Crown.

[mono12479-bib-0161] Nosek, B. A. , Hardwicke, T. E. , Moshontz, H. , Allard, A. , Corker, K. S. , Dreber, A. , Fidler, F. , Hilgard, J. , Struhl, M. K. , Nuijten, M. B. , Rohrer, J. M. , Romero, F. , Scheel, A. M. , Scherer, L. D. , Schönbrodt, F. D. , & Vazire, S. (2022). Replicability, robustness, and reproducibility in psychological science. Annual Review of Psychology, 73, 719–748. 10.1146/annurev-psych-020821-114157 34665669

[mono12479-bib-0162] Olson, K. R. (2016). Prepubescent transgender children: What we do and do not know. Journal of the American Academy of Child & Adolescent Psychiatry, 55(3), 155–156. 10.1016/j.jaac.2015.11.015 26903246

[mono12479-bib-0163] Olson, J. , Schrager, S. M. , Belzer, M. , Simons, L. K. , & Clark, L. F. (2015). Baseline physiologic and psychosocial characteristics of transgender youth seeking care for Gender Dysphoria. Journal of Adolescent Health, 57(4), 374–380. 10.1016/j.jadohealth.2015.04.027 PMC5033041 26208863

[mono12479-bib-0164] Olson, K. R. , Durwood, L. , Horton, R. , Gallagher, N. M. , & Devor, A. (2022). Gender identity 5 years after social transition. Pediatrics, 150(2), e2021056082. 10.1542/peds.2021-056082 35505568 PMC9936352

[mono12479-bib-0165] Olson, K. R. , Raber, G. F. , & Gallagher, N. M. (2024). Levels of satisfaction and regret with gender‐affirming medical care in adolescence. JAMA Pediatrics, 178(12), 1354–1361. 10.1001/jamapediatrics.2024.4527 39432272 PMC11581734

[mono12479-bib-0166] Ott, M. Q. , Corliss, H. L. , Wypij, D. , Rosario, M. , & Austin, S. B. (2011). Stability and change in self‐reported sexual orientation identity in young people: Application of mobility metrics. Archives of Sexual Behavior, 40(3), 519–532. 10.1007/s10508-010-9691-3 21125325 PMC3081371

[mono12479-bib-0167] Parker, K. , Horowitz, J. M. , & Brown, A. (2022, June 28). Americans' complex views on gender identity and transgender issues. *Pew Research Center*. https://www.pewresearch.org/social-trends/2022/06/28/americans-complex-views-on-gender-identity-and-transgender-issues/

[mono12479-bib-0168] Parks, C. (2024, November 5). How a grandma became the focus of a ‘misinformed’ Trump ad on trans athletes. *Washington Post*. https://www.washingtonpost.com/nation/2024/11/05/election-trans-sports-trump-campaign/

[mono12479-bib-0169] Paul, P. (2024, February 2). As kids, they thought they were trans. They no longer do. *The New York Times*. https://www.nytimes.com/2024/02/02/opinion/transgender-children-gender-dysphoria.html

[mono12479-bib-0170] PBS . (2024, November 2). Why anti‐transgender political ads are dominating the airwaves this election. *PBS News*. https://www.pbs.org/newshour/show/why-anti-transgender-political-ads-are-dominating-the-airwaves-this-election

[mono12479-bib-0171] Peng, K. , Zhu, X. , Gillespie, A. , Wang, Y. , Gao, Y. , Xin, Y. , Qi, J. , Ou, J. , Zhong, S. , Zhao, L. , Liu, J. , Wang, C. , & Chen, R. (2019). Self‐reported rates of abuse, neglect, and bullying experienced by transgender and gender‐nonbinary adolescents in China. JAMA Network Open, 2(9), e1911058. 10.1001/jamanetworkopen.2019.11058 31490542 PMC6735403

[mono12479-bib-0172] Perszyk, D. R. , Lei, R. F. , Bodenhausen, G. V. , Richeson, J. A. , & Waxman, S. R. (2019). Bias at the intersection of race and gender: Evidence from preschool‐aged children. Developmental Science, 22(3), e12788. 10.1111/desc.12788 30675747

[mono12479-bib-0173] Phillips, G. L. , Curtis, M. G. , Kelsey, S. W. , Floresca, Y. B. , Davoudpour, S. , Quiballo, K. , & Beach, L. B. (2024). Changes to sexual identity response options in the youth risk behavior survey. JAMA Pediatrics, 178(5), 506–508. 10.1001/jamapediatrics.2024.0024 38436940 PMC10912997

[mono12479-bib-0174] Pickstone‐Taylor, S. D. , Davids, E. L. , de Bever, G. N. , & de Vries, P. J. (2024). Demographic and mental health profile of youth in a gender service: An African case series. South African Journal of Psychiatry, 30(1), 2160. 10.4102/sajpsychiatry.v30i0.2160 PMC11079370 38726329

[mono12479-bib-0175] Plumb, P. , & Cowan, G. (1984). A developmental study of destereotyping and androgynous activity preferences of tomboys, nontomboys, and males. Sex Roles, 10(9), 703–712. 10.1007/BF00287381

[mono12479-bib-0176] Poteat, V. P. , Mereish, E. H. , DiGiovanni, C. D. , & Koenig, B. W. (2011). The effects of general and homophobic victimization on adolescents' psychosocial and educational concerns: The importance of intersecting identities and parent support. Journal of Counseling Psychology, 58(4), 597–609. 10.1037/a0025095 21859187

[mono12479-bib-0177] Price‐Feeney, M. , Green, A. E. , & Dorison, S. (2020). Understanding the mental health of transgender and nonbinary youth. Journal of Adolescent Health, 66(6), 684–690. 10.1016/j.jadohealth.2019.11.314 31992489

[mono12479-bib-0178] PRRI . (2023, June 8). The politics of gender, pronouns, and public education. *PRRI*. https://www.prri.org/research/the-politics-of-gender-pronouns-and-public-education/

[mono12479-bib-0179] Puckett, J. A. , Tornello, S. , Mustanski, B. , & Newcomb, M. E. (2022). Gender variations, generational effects, and mental health of transgender people in relation to timing and status of gender identity milestones. Psychology of Sexual Orientation and Gender Diversity, 9(2), 165–178. 10.1037/sgd0000391 35983565 PMC9380989

[mono12479-bib-0180] Pullen Sansfaçon, A. , Gravel, É. , Gelly, M. , Planchat, T. , Paradis, A. , & Medico, D. (2024). A retrospective analysis of the gender trajectories of youth who have discontinued a transition. International Journal of Transgender Health, 25(1), 74–89. 10.1080/26895269.2023.2279272 38328586 PMC10846427

[mono12479-bib-0181] Purdie‐Vaughns, V. , & Eibach, R. P. (2008). Intersectional Invisibility: The distinctive advantages and disadvantages of multiple subordinate‐group identities. Sex Roles, 59(5), 377–391. 10.1007/s11199-008-9424-4

[mono12479-bib-0182] Quinn, P. C. , Yahr, J. , Kuhn, A. , Slater, A. M. , & Pascalis, O. (2002). Representation of the gender of human faces by infants: A preference for female. Perception, 31(9), 1109–1121. 10.1068/p3331 12375875

[mono12479-bib-0183] R Core Team . (2024). R: A Language and Environment for Statistical Computing. *R Foundation for Statistical Computing*. https://www.R-project.org/

[mono12479-bib-0184] Rae, J. R. , Gülgöz, S. , Durwood, L. , DeMeules, M. , Lowe, R. , Lindquist, G. , & Olson, K. R. (2019). Predicting early‐childhood gender transitions. Psychological Science, 30(5), 669–681. 10.1177/0956797619830649 30925121 PMC6512159

[mono12479-bib-0185] Reck, J. (2009). Homeless gay and transgender youth of color in San Francisco: “No one likes street kids”—Even in the Castro. Journal of LGBT Youth, 6(2–3), 223–242. 10.1080/19361650903013519

[mono12479-bib-0186] Rekers, G. A. , Bentler, P. M. , Rosen, A. C. , & Lovaas, O. I. (1977). Child gender disturbances: A clinical rationale for intervention. Psychotherapy: Theory, Research & Practice, 14(1), 2–11. 10.1037/h0087487

[mono12479-bib-0187] Rekers, G. A. , Yates, C. E. , Willis, T. J. , Rosen, A. C. , & Taubman, M. (1976). Childhood gender identity change: Operant control over sex‐typed play and mannerisms. Journal of Behavior Therapy and Experimental Psychiatry, 7(1), 51–57. 10.1016/0005-7916(76)90043-4

[mono12479-bib-0188] Rider, G. N. , Vencill, J. A. , Berg, D. R. , Becker‐Warner, R. , Candelario‐Pérez, L. , & Spencer, K. G. (2020). The gender affirmative lifespan approach (GALA): A framework for competent clinical care with nonbinary clients. In J. Motmans , T. O. Nieder , & W. Bouman (Eds.), Non‐binary and genderqueer genders. Routledge. 10.1080/15532739.2018.1485069 PMC6831004 32999613

[mono12479-bib-0189] Rogers, L. O. , Scott, M. A. , & Way, N. (2015). Racial and gender identity among Black adolescent males: An intersectionality perspective. Child Development, 86(2), 407–424. 10.1111/cdev.12303 25363136

[mono12479-bib-0190] Rosario, M. , Meyer‐Bahlburg, H. F. L. , Hunter, J. , Exner, T. M. , Gwadz, M. , & Keller, A. M. (1996). The psychosexual development of urban lesbian, gay, and bisexual youths. The Journal of Sex Research, 33(2), 113–126. 10.1080/00224499609551823

[mono12479-bib-0191] Rosario, M. , Schrimshaw, E. W. , Hunter, J. , & Braun, L. (2006). Sexual identity development among lesbian, gay, and bisexual youths: Consistency and change over time. The Journal of Sex Research, 43(1), 46–58. 10.1080/00224490609552298 16817067 PMC3215279

[mono12479-bib-0192] Ryan, C. , Huebner, D. , Diaz, R. M. , & Sanchez, J. (2009). Family rejection as a predictor of negative health outcomes in White and Latino Lesbian, Gay, and Bisexual young adults. Pediatrics, 123(1), 346–352. 10.1542/peds.2007-3524 19117902

[mono12479-bib-0193] Salk, R. H. , Thoma, B. C. , & Choukas‐Bradley, S. (2020). The gender minority youth study: Overview of methods and social media recruitment of a nationwide sample of U.S. cisgender and transgender adolescents. Archives of Sexual Behavior, 49(7), 2601–2610. 10.1007/s10508-020-01695-x 32306108 PMC7865131

[mono12479-bib-0194] Sandfort, T. G. M. (2005). Sexual orientation and gender: Stereotypes and beyond. Archives of Sexual Behavior, 34(6), 595–611. 10.1007/s10508-005-7907-8 16362245

[mono12479-bib-0195] Santoro, H. (2022, January 1). Open science is surging. *American Psychological Association*. https://www.apa.org/monitor/2022/01/special-open-science

[mono12479-bib-0196] Santos, C. E. , & Toomey, R. B. (2018). Integrating an intersectionality lens in theory and research in developmental science. New Directions for Child and Adolescent Development, 2018(161), 7–15. 10.1002/cad.20245 29969178

[mono12479-bib-0197] Sapir, L. , Littman, L. , & Biggs, M. (2024). The U.S. Transgender Survey of 2015 supports rapid‐onset gender dysphoria: Revisiting the “Age of Realization and Disclosure of Gender Identity Among Transgender Adults.” Archives of Sexual Behavior, 53(3), 863–868. 10.1007/s10508-023-02754-9 38110845 PMC10920421

[mono12479-bib-0198] Savin‐Williams, R. C. (1995). An exploratory study of pubertal maturation timing and self‐esteem among gay and bisexual male youths. Developmental Psychology, 31(1), 56–64. 10.1037/0012-1649.31.1.56

[mono12479-bib-0199] Savin‐Williams, R. C. (2006). Who's gay? Does it matter? Current Directions in Psychological Science, 15(1), 40–44. 10.1111/j.0963-7214.2006.00403.x PMC2435044 18568084

[mono12479-bib-0200] Savin‐Williams, R. C. , & Ream, G. L. (2007). Prevalence and stability of sexual orientation components during adolescence and young adulthood. Archives of Sexual Behavior, 36(3), 385–394. 10.1007/s10508-006-9088-5 17195103

[mono12479-bib-0201] Savin‐Williams, R. C. , Joyner, K. , & Rieger, G. (2012). Prevalence and stability of self‐reported sexual orientation identity during young adulthood. Archives of Sexual Behavior, 41(1), 103–110. 10.1007/s10508-012-9913-y 22302504

[mono12479-bib-0202] Schick, V. , Calabrese, S. K. , & Herbenick, D. (2014). Survey methods in sexuality research. In D. L. Tolman , L. M. Diamond , J. A. Bauermeister , W. H. George , J. G. Pfaus , & L. M. Ward (Eds.), APA handbook of sexuality and psychology, Vol. 1: Person‐based approaches (pp. 81–98). American Psychological Association. 10.1037/14193-004

[mono12479-bib-0287] Serano, J. (2020). Autogynephilia: A scientific review, feminist analysis, and alternative ‘embodiment fantasies’ model. Sociological Review, 68(4), 763–778. 10.1177/0038026120934690

[mono12479-bib-0203] Serbin, L. A. , Powlishta, K. K. , Gulko, J. , Martin, C. L. , & Lockheed, M. E. (1993). The development of sex typing in middle childhood. Monographs of the Society for Research in Child Development, 58(2), i–95. 10.2307/1166118 8474512

[mono12479-bib-0204] Sesko, A. K. , & Biernat, M. (2010). Prototypes of race and gender: The invisibility of Black women. Journal of Experimental Social Psychology, 46(2), 356–360. 10.1016/j.jesp.2009.10.016

[mono12479-bib-0205] Shrier, A. (2020). Irreversible damage: The transgender craze seducing our daughters. Simon and Schuster.

[mono12479-bib-0206] Signorella, M. L. , Bigler, R. S. , & Liben, L. S. (1993). Developmental differences in children′s gender schemata about others: A meta‐analytic review. Developmental Review, 13(2), 147–183. 10.1006/drev.1993.1007

[mono12479-bib-0207] Simmons, J. P. , Nelson, L. D. , & Simonsohn, U. (2011). False‐positive psychology: Undisclosed flexibility in data collection and analysis allows presenting anything as significant. Psychological Science, 22(11), 1359–1366. 10.1177/0956797611417632 22006061

[mono12479-bib-0208] Singh, A. A. (2013). Transgender youth of color and resilience: Negotiating oppression and finding support. Sex Roles, 68(11), 690–702. 10.1007/s11199-012-0149-z

[mono12479-bib-0209] Singh, D. , Bradley, S. J. , & Zucker, K. J. (2021). A follow‐up study of boys with gender identity disorder. Frontiers in Psychiatry, 12, 632784. https://www.frontiersin.org/articles/10.3389/fpsyt.2021.632784 33854450 10.3389/fpsyt.2021.632784 PMC8039393

[mono12479-bib-0210] Skinner, O. D. , & McHale, S. M. (2018). The development and correlates of gender role orientations in African‐American youth. Child Development, 89(5), 1704–1719. 10.1111/cdev.12828 28474457

[mono12479-bib-0211] Slaby, R. G. , & Frey, K. S. (1975). Development of gender constancy and selective attention to same‐sex models. Child Development, 46(4), 849–856. 10.2307/1128389 1201664

[mono12479-bib-0212] Spack, N. P. , Edwards‐Leeper, L. , Feldman, H. A. , Leibowitz, S. , Mandel, F. , Diamond, D. A. , & Vance, S. R. (2012). Children and adolescents with gender identity disorder referred to a pediatric medical center. Pediatrics, 129(3), 418–425. 10.1542/peds.2011-0907 22351896

[mono12479-bib-0213] Spence, J. T. (1993). Gender‐related traits and gender ideology: Evidence for a multifactorial theory. Journal of Personality and Social Psychology, 64(4), 624–635. 10.1037/0022-3514.64.4.624 8473979

[mono12479-bib-0214] Spence, J. T. , & Helmreich, R. L. (1979). Masculinity and Femininity: Their Psychological Dimensions, Correlates, and Antecedents. University of Texas Press.

[mono12479-bib-0215] StatCan . (2022, April 27). Canada is the first country to provide census data on transgender and non‐binary people. *Statistics Canada—Statistique Canada*. https://www150.statcan.gc.ca/n1/daily-quotidien/220427/dq220427b-eng.htm

[mono12479-bib-0216] Steensma, T. D. , & Cohen‐Kettenis, P. T. (2011). Gender transitioning before puberty? Archives of Sexual Behavior, 40(4), 649–650. 10.1007/s10508-011-9752-2 21373942

[mono12479-bib-0217] Steensma, T. D. , & Cohen‐Kettenis, P. T. (2015). More than two developmental pathways in children with gender dysphoria? Journal of the American Academy of Child & Adolescent Psychiatry, 54(2), 147–148. 10.1016/j.jaac.2014.10.016 25617255

[mono12479-bib-0218] Steensma, T. D. , Biemond, R. , de Boer, F. , & Cohen‐Kettenis, P. T. (2011). Desisting and persisting gender dysphoria after childhood: A qualitative follow‐up study. Clinical Child Psychology and Psychiatry, 16(4), 499–516. 10.1177/1359104510378303 21216800

[mono12479-bib-0271] Steensma, T. D. , McGuire, J. K. , Kreukels, B. P. C. , Beekman, A. J. , & Cohen‐Kettenis, P. T. (2013a). Factors associated with desistence and persistence of childhood gender dysphoria: A quantitative follow‐up study. Journal of the American Academy of Child & Adolescent Psychiatry, 52(6), 582–590. 10.1016/j.jaac.2013.03.016 23702447

[mono12479-bib-0272] Steensma, T. D. , van der Ende, J. , Verhulst, F. C. , & Cohen‐Kettenis, P. T. (2013b). Gender variance in childhood and sexual orientation in adulthood: A prospective study. Journal of Sexual Medicine, 10(11), 2723–2733. 10.1111/j.1743-6109.2012.02701.x 22458332

[mono12479-bib-0221] Stewart, J. L. , Spivey, L. A. , Widman, L. , Choukas‐Bradley, S. , & Prinstein, M. J. (2019). Developmental patterns of sexual identity, romantic attraction, and sexual behavior among adolescents over three years. Journal of Adolescence, 77, 90–97. 10.1016/j.adolescence.2019.10.006 31693971 PMC6885553

[mono12479-bib-0222] Stoller, R. J. (1967). “It's Only a Phase”: Femininity in boys. JAMA, 201(5), 314–315. 10.1001/jama.1967.03130050048014 6071749

[mono12479-bib-0223] Stoller, R. J. (1968). A further contribution to the study of gender identity. The International Journal of Psycho‐Analysis, 49, 364–369. 5698204

[mono12479-bib-0224] Suarez, N. A. , Trujillo, L. , McKinnon, I. I. , Mack, K. A. , Lyons, B. , Robin, L. , Carman‐McClanahan, M. , Pampati, S. , Cezair, K. L. R. , & Ethier, K. A. (2024). Disparities in school connectedness, unstable housing, experiences of violence, mental health, and suicidal thoughts and behaviors among transgender and cisgender high school students—Youth risk behavior survey, United States, 2023. *MMWR Supplements*. https://www.cdc.gov/mmwr/volumes/73/su/su7304a6.htm 10.15585/mmwr.su7304a6 PMC11559675 39378210

[mono12479-bib-0276] Szoko, N. , Sequeira, G. M. , Coulter, R. W. S. , Kobey, J. , Ridenour, E. , Burnett, O. , & Kidd, K. M. (2023). Sexual Orientation Among Gender Diverse Youth. Journal of Adolescent Health, 72(1), 153–155. 10.1016/j.jadohealth.2022.08.016 PMC10748722 36216680

[mono12479-bib-0225] Tannehill, B. (2017, January 1). The end of the desistance myth. *Huffington Post*. https://www.huffpost.com/entry/the-end-of-the-desistance_b_8903690

[mono12479-bib-0293] Tate, C. C. , Youssef, C. P. , & Bettergarcia, J. N. (2014). Integrating the study of transgender spectrum and cisgender experiences of self‐categorization from a personality perspective. Review of General Psychology, 18(4), 302–312. 10.1037/gpr0000019

[mono12479-bib-0226] Tausanovitch, C. , & Warshaw, C. (2014). Representation in municipal government. American Political Science Review, 108(3), 605–641. 10.1017/S0003055414000318

[mono12479-bib-0227] Temple Newhook, J. , Pyne, J. , Winters, K. , Feder, S. , Holmes, C. , Tosh, J. , Sinnott, M.‐L. , Jamieson, A. , & Pickett, S. (2018). A critical commentary on follow‐up studies and “desistance” theories about transgender and gender‐nonconforming children. International Journal of Transgenderism, 19(2), 212–224. 10.1080/15532739.2018.1456390

[mono12479-bib-0228] Terhune, C. , Respaut, R. , & Conlin, M. (2022, October 6). As children line up at gender clinics, families confront many unknowns. *Reuters*. https://www.reuters.com/investigates/special-report/usa-transyouth-care/

[mono12479-bib-0229] The Trevor Project . (2021). The Trevor Project National Survey. *The Trevor Project*. https://www.TheTrevorProject.org/survey-2021/

[mono12479-bib-0230] The White House . (2025a, January 21). Defending women from gender ideology extremism and restoring biological truth to the Federal Government. *The White House*. https://www.whitehouse.gov/presidential-actions/2025/01/defending-women-from-gender-ideology-extremism-and-restoring-biological-truth-to-the-federal-government/

[mono12479-bib-0231] The White House . (2025b, January 28). Protecting children from chemical and surgical mutilation. *The White House*. https://www.whitehouse.gov/presidential-actions/2025/01/protecting-children-from-chemical-and-surgical-mutilation/

[mono12479-bib-0232] The White House . (2025c, January 29). Ending radical indoctrination in K‐12 schooling. *The White House*. https://www.whitehouse.gov/presidential-actions/2025/01/ending-radical-indoctrination-in-k-12-schooling/

[mono12479-bib-0233] The White House . (2025d, February 5). Keeping men out of women's sports. *The White House*. https://www.whitehouse.gov/presidential-actions/2025/02/keeping-men-out-of-womens-sports/

[mono12479-bib-0234] Thompson, S. K. (1975). Gender labels and early sex role development. Child Development, 46(2), 339–347. 10.2307/1128126 1183267

[mono12479-bib-0235] Thompson, L. , Sarovic, D. , Wilson, P. , Sämfjord, A. , & Gillberg, C. (2022). A PRISMA systematic review of adolescent gender dysphoria literature: 1) Epidemiology. PLOS Global Public Health, 2(3), e0000245. 10.1371/journal.pgph.0000245 36962334 PMC10021877

[mono12479-bib-0236] Tobin, D. D. , Menon, M. , Menon, M. , Spatta, B. C. , Hodges, E. V. E. , & Perry, D. G. (2010). The intrapsychics of gender: A model of self‐socialization. Psychological Review, 117(2), 601–622. 10.1037/a0018936 20438239

[mono12479-bib-0237] Trans Legislation Tracker . (2024). 2024 Anti‐trans bills. *Trans Legislation Tracker*. https://translegislation.com

[mono12479-bib-0238] Trautner, H. M. , Ruble, D. N. , Cyphers, L. , Kirsten, B. , Behrendt, R. , & Hartmann, P. (2005). Rigidity and flexibility of gender stereotypes in childhood: Developmental or differential? Infant and Child Development, 14(4), 365–381. 10.1002/icd.399

[mono12479-bib-0239] Twenge, J. M. (2023a). Generations: The real differences between Gen Z, Millennials, Gen X, Boomers, and silents—and what they mean for America's future. Simon and Schuster.

[mono12479-bib-0240] Twenge, J. M. (2023b, May 1). How Gen Z changed its views on gender. *TIME*. https://time.com/6275663/generation-z-gender-identity/

[mono12479-bib-0241] Twenge, J. M. (2024, October 20). The surprising number of young adults who identify as nonbinary. *Generation Tech*. https://www.generationtechblog.com/p/the-surprising-number-of-young-adults

[mono12479-bib-0242] Twenge, J. M. , Wells, B. E. , Le, J. , & Rider, G. N. (2024). Increases in self‐identifying as transgender among US adults, 2014–2022. Sexuality Research and Social Policy, 22, 755–773. 10.1007/s13178-024-01001-7

[mono12479-bib-0243] Vance, Jr., S. R. , Boyer, C. B. , Glidden, D. V. , & Sevelius, J. (2021). Mental health and psychosocial risk and protective factors among black and latinx transgender youth compared with peers. JAMA Network Open, 4(3), e213256. 10.1001/jamanetworkopen.2021.3256 33769506 PMC7998078

[mono12479-bib-0244] Vandendriessche, L. (2023, March 29). *“Pano” onderzoekt medische behandeling van trans tieners: Grote stijging aanmeldingen, maar ook discussie over juiste aanpak*. VRT NWS. https://www.vrt.be/vrtnws/nl/2023/03/26/trans-tieners/

[mono12479-bib-0245] Vasey, P. L. , & Bartlett, N. H. (2007). What can the Samoan “Fa'afafine” teach us about the western concept of gender identity disorder in childhood? Perspectives in Biology and Medicine, 50(4), 481–490. https://muse.jhu.edu/pub/1/article/222247 17951883 10.1353/pbm.2007.0056

[mono12479-bib-0285] Verhulst, F. C. , van der Ende, J. , & Koot, H. M. (1996). Handleiding voor de CBCL/4‐18. Sophia Kinderziekenhuis/Academisch Ziekenhuis/Erasmus Universiteit.

[mono12479-bib-0246] Vilain, E. , & Bailey, J. M. (2015, May 21). What should you do if your son says he's a girl? *Los Angeles Times*. https://www.latimes.com/opinion/op-ed/la-oe-vilain-transgender-parents-20150521-story.html

[mono12479-bib-0247] Wadman, M. (2018). ‘Rapid onset’ of transgender identity ignites storm. Science, 361(6406), 958–959. 10.1126/science.361.6406.958 30190384

[mono12479-bib-0248] Wallien, M. S. C. , & Cohen‐Kettenis, P. T. (2008). Psychosexual outcome of gender‐dysphoric children. Journal of the American Academy of Child & Adolescent Psychiatry, 47(12), 1413–1423. 10.1097/CHI.0b013e31818956b9 18981931

[mono12479-bib-0249] Weinraub, M. , Clemens, L. P. , Sockloff, A. , Ethridge, T. , Gracely, E. , & Myers, B. (1984). The Development of sex role stereotypes in the third year: Relationships to gender labeling, gender identity, sex‐types toy preference, and family characteristics. Child Development, 55(4), 1493–1503. 10.2307/1130019 6488962

[mono12479-bib-0250] Wickham, H. , Averick, M. , Bryan, J. , Chang, W. , McGowan, L. D. , François, R. , Grolemund, G. , Hayes, A. , Henry, L. , Hester, J. , Kuhn, M. , Pedersen, T. L. , Miller, E. , Bache, S. M. , Müller, K. , Ooms, J. , Robinson, D. , Seidel, D. P. , Spinu, V. , Yutani, H. (2019). Welcome to the tidyverse. Journal of Open Source Software, 4(43), 1686. 10.21105/joss.01686

[mono12479-bib-0251] Wickham, H. , Pedersen, T. L. , & Seidel, D. (2023). scales: Scale Functions for Visualization. *R Pack version*. https://scales.r-lib.org

[mono12479-bib-0252] William Revelle . (2024a). psych: Procedures for Psychological, Psychometric, and Personality Research. *Northwestern University*. https://CRAN.R-project.org/package=psych

[mono12479-bib-0253] William Revelle . (2024b). psychTools: Tools to Accompany the “psych” Package for Psychological Research. *Northwestern University*. https://CRAN.R-project.org/package=psychTools

[mono12479-bib-0288] Winters, K. (2008). Autogynephilia: The infallible derogatory hypothesis, part 1. GID Reform Weblog by Kelley Winters. https://gidreform.wordpress.com/2008/11/10/autogynephilia-the-infallible-derogatory-hypothesis-part-1/

[mono12479-bib-0254] Winters, K. (2019). The “80% desistance” dictum: Is it science? In A. I. Lev & A. R. Gottlieb (Eds.), Families in transition: Parenting gender diverse children, adolescents, and young adults (pp. 88–101). Harrington Park Press.

[mono12479-bib-0255] Winters, K. , Temple Newhook, J. , Pyne, J. , Feder, S. , Jamieson, A. , Holmes, C. , Sinnott, M.‐L. , Pickett, S. , & Tosh, J. (2018). Learning to listen to trans and gender diverse children: A Response to Zucker (2018) and Steensma and Cohen‐Kettenis (2018). International Journal of Transgenderism, 19(2), 246–250. 10.1080/15532739.2018.1471767

[mono12479-bib-0219] Wittlin N. M. , Gallagher N. M. , Atwood S. , & Olson K. R. (2025). Mental health during medical transition in a us and Canadian sample of early socially transitioned transgender youth. Journal of Adolescent Health, 76(2), 228–237. https://www.sciencedirect.com/science/article/pii/S1054139X24005196 10.1016/j.jadohealth.2024.10.023 PMC11738661 39520461

[mono12479-bib-0256] Wittlin, N. M. , Gallagher, N. M. , & Olson, K. R. (2024). Gender identity importance in cisgender and gender diverse adolescents in the US and Canada. *British Journal of Developmental Psychology*. 10.1111/bjdp.12485 PMC11461692 38591552

[mono12479-bib-0257] Xie, Y. (2024). *knitr: A general‐purpose package for dynamic report generation in R*. https://yihui.org/knitr/

[mono12479-bib-0258] Ybarra, M. L. , Price‐Feeney, M. , & Mitchell, K. J. (2019). A cross‐sectional study examining the (in)congruency of sexual identity, sexual behavior, and romantic attraction among adolescents in the US. The Journal of Pediatrics, 214, 201–208. 10.1016/j.jpeds.2019.06.046 31402142 PMC7202349

[mono12479-bib-0259] Yunger, J. L. , Carver, P. R. , & Perry, D. G. (2004). Does gender identity influence children's psychological well‐being? Developmental Psychology, 40(4), 572–582. 10.1037/0012-1649.40.4.572 15238044

[mono12479-bib-0260] Zhu, H. , Travison, T. , Tsai, T. , Beasley, W. , Xie, Y. , Yu, G. , Laurent, S. , Shepherd, R. , Sidi, Y. , Salzer, B. , Gui, G. , Fan, Y. , Murdoch, D. , Arel‐Bundock, V. , & Evans, B. (2024). *kableExtra: Construct Complex Table with “kable” and Pipe Syntax* (Version 1.4.0) [Computer software]. https://cran.r-project.org/web/packages/kableExtra/index.html

[mono12479-bib-0261] Zucker, K. J. (2005). Gender identity disorder in children and adolescents. Annual Review of Clinical Psychology, 1(1), 467–492. 10.1146/annurev.clinpsy.1.102803.144050 17716096

[mono12479-bib-0262] Zucker, K. J. (2014). Gender dysphoria. In M. Lewis & K. D. Rudolph (Eds.), Handbook of Developmental Psychopathology (pp. 683–702). Springer US. 10.1007/978-1-4614-9608-3_35

[mono12479-bib-0263] Zucker, K. J. (2017). Epidemiology of gender dysphoria and transgender identity. Sexual Health, 14(5), 404–411. 10.1071/SH17067 28838353

[mono12479-bib-0264] Zucker, K. J. (2018). The myth of persistence: Response to “A critical commentary on follow‐up studies and ‘desistance’ theories about transgender and gender non‐conforming children” by Temple Newhook et al. (2018). International Journal of Transgenderism, 19(2), 231–245. https://www.tandfonline.com/doi/abs/10.1080/15532739.2018.1468293

[mono12479-bib-0265] Zucker, K. J. , & Bradley, S. J. (1995). Gender identity disorder and psychosexual problems in children and adolescents. Guilford Press. 10.1177/070674379003500603 2207982

[mono12479-bib-0266] Zucker, K. J. , Bradley, S. J. , Doering, R. W. , & Lozinski, J. A. (1985). Sex‐typed behavior in cross‐gender‐identified children: Stability and change at a one‐year follow‐up. Journal of the American Academy of Child Psychiatry, 24(6), 710–719. 10.1016/S0002-7138(10)60114-8 4067140

[mono12479-bib-0267] Zucker, K. J. , Bradley, S. J. , Kuksis, M. , Pecore, K. , Birkenfeld‐Adams, A. , Doering, R. W. , Mitchell, J. N. , & Wild, J. (1999). Gender constancy judgments in children with gender identity disorder: Evidence for a developmental lag. Archives of Sexual Behavior, 28(6), 475–502. 10.1023/A:1018713115866 10650437

[mono12479-bib-0282] Zucker, K. J. , Bradley, S. J. , Sullivan, C. B. L. , Kuksis, M. , Birkenfeld‐Adams, A. , & Mitchell, J. N. (1993). A gender identity interview for children. Journal of Personality Assessment, 61(3), 443–456. 10.1207/s15327752jpa6103_2 8295110

[mono12479-bib-0280] Zucker, K. J. , Doering, R. W. , Bradley, S. J. , & Finegan, J.‐A. K. (1982). Sex‐typed play in gender‐disturbed children: A comparison to sibling and psychiatric controls. Archives of Sexual Behavior, 11(4), 309–321. 10.1007/bf01541592 7149966

[mono12479-bib-0281] Zucker, K. J. , Finegan, J.‐A. K. , Doering, R. W. , & Bradley, S. J. (1983). Human figure drawings of gender‐problem children: A comparison to sibling, psychiatric, and normal controls. Journal of Abnormal Child Psychology, 11(2), 287–298. 10.1007/bf00912092 6352776

[mono12479-bib-0283] Zucker, K. J. , Mitchell, J. N. , Bradley, S. J. , Tkachuk, J. , Cantor, J. M. , & Allin, S. M. (2006). The recalled childhood gender identity/gender role questionnaire: Psychometric properties. Sex Roles, 54(7–8), 469–483. 10.1007/s11199-006-9019-x

[mono12479-bib-0268] Zucker, K. J. , Wood, H. , Singh, D. , & Bradley, S. J. (2012). A developmental, biopsychosocial model for the treatment of children with gender identity disorder. Journal of Homosexuality, 59(3), 369–397. 10.1080/00918369.2012.653309 22455326

[mono12479-bib-0269] Zuger, B. (1966). Effeminate behavior present in boys from early childhood: I. The clinical syndrome and follow‐up studies. The Journal of Pediatrics, 69(6), 1098–1107. 10.1016/S0022-3476(66)80301-3 5953837

[mono12479-bib-0270] Zuger, B. , & Taylor, P. (1969). Effeminate behavior present in boys from early in childhood: II. Comparison with similar symptoms in non‐effeminate boys. Pediatrics, 44(3), 375–380. 10.1542/peds.44.3.375 5809894

