# Supplementary material for: Stability and Change in Gender Identity and Sexual Orientation Across Childhood and Adolescence
Source: Monogr Soc Res Child Dev. 2025 Jul 15;90(1-3):7–172. doi: 10.1111/mono.12479 (PMC12260785; doi:10.1111/mono.12479)
Supplement: Supplementary file 1 — Supporting Info. [file MONO-90-7-s001.docx]

**Supporting Information for:**

**Stability and Change in Gender Identity and Sexual Orientation Across Childhood and Adolescence**

**Supporting Information for Chapter II:**

**Trans Youth Project Participants and General Design**

In the sections below, we provide information related to how each of the parent-reported variables contained in Chapter II were derived.

**Overview of Parent-reported Variables**

The parent-reported variables described in Chapter II (e.g., demographic measures, variables related to children’s social transitions) vary both in *how many times* parents responded to the measure and in *which parent(s)* responded to it. As a result of this variation, our process for cleaning these measures and deriving variables for use in Chapter II vary somewhat across the measure in question. For a subset of measures, we use lab-standardized versions of each variable, which allows for consistency with other manuscripts using the TYP dataset. These lab-coded variables reflect the *earliest* parent report received for each measure, irrespective of which parent reported it; in instances in which we have more than one parent report from the same (earliest) visit, we use data from the parent who was in greater contact with the research team as of that visit. For another subset of measures, we include reports from the "primary parent”—defined as the parent who provided the most responses over the course of the child’s participation in the study—unless they did not respond to the measure in question, in which case we include data from secondary parents. If primary parents (or secondary parents, in their absence) provided an answer to the latter set of measures across multiple visits, we use the answer provided the first time they were asked.

**Lab-standardized Variables**

The following measures from Chapter II reflect lab-standardized versions: youth sex assigned at birth; youth race-ethnicity, as originally reported by parents; parent race-ethnicity; household income; educational attainment; political ideology; geographic location; primary parent gender; age of social transition (for *Recruited as Transgender* youth); age of starting and stopping blockers (for *Recruited as Transgender* youth); and age of starting and stopping gender affirming hormones (for *Recruited as Transgender* youth).

***Variables Reported by Primary Parent (or, in Absence of Data, Secondary Parent)***

The following measures from Chapter II contain data from the primary parent when it is available and the secondary parent when it is not (note that all of these measures are reported only for *Recruited as Transgender* youth): age of youths’ first “cross-gender” behavior, age at which parents learned that their children did not identify with the gender associated with their sex assigned at birth, and parents’ perceptions of social support for their children’s gender identity/expression.

**Further Information about “Original” 316 *Recruited as Cisgender* Participants**

As described in Chapter II, some participants in the *Recruited as Cisgender* group (N = 316) can be conceptualized as “original” matched comparison participants to the 317 *Recruited as Transgender* youths, while a smaller subset (N = 61) can be conceptualized as replacements for the original matched comparison group and/or their initial replacements. Here, we report demographic information and gender and sexual orientation outcomes for the 316 “original” matched comparison participants.

***Demographic Information and Study Participation Characteristics of “Original” 316 Recruited as Cisgender Participants***

**Table S1**

*Demographic Information of Youths and Parents, Including Column with 316 “Original” Recruited as Cisgender Participants*

|  | Recruited as Transgender | Recruited as Siblings | Recruited as Cisgender (all) | Recruited as Cisgender (orig) |
| --- | --- | --- | --- | --- |
| Youth assigned sex at birth: N (%) |  |  |  |  |
| Male | 208 (66%) | 125 (57%) | 128 (34%) | 109 (34%) |
| Female | 109 (34%) | 93 (43%) | 249 (66%) | 207 (66%) |
| Youth age as of latest visit: M (SD, range) | 15.10 (*SD* = 3.04, *range:* 4-21) | 13.99 (*SD* = 3.88, *range:* 5 – 22) | 13.78 (*SD*=3.60, *range:* 4 – 22) | 13.99 (*SD*=3.69, *range:* 4 – 22) |
| Youth race-ethnicity (parent-reported): N (%) |  |  |  |  |
| White, non-Hispanic | 219 (69%) | 151 (69%) | 255 (68%) | 224 (71%) |
| White, Hispanic | 25 (8%) | 19 (9%) | 20 (5%) | 19 (6%) |
| Multiracial, non-Hispanic | 41 (13%) | 24 (11%) | 70 (19%) | 54 (17%) |
| Asian, non-Hispanic | 9 (3%) | 4 (2%) | 13 (3%) | 9 (3%) |
| Black/African, non-Hispanic | 5 (2%) | 4 (2%) | 4 (1%) | 3 (1%) |
| Other | 14 (4%) | 8 (4%) | 7 (2%) | 4 (1%) |
| Not reported | 4 (1%) | 8 (4%) | 8 (2%) | 3 (1%) |
| Primary parent race-ethnicity: N (%) |  |  |  |  |
| White, non-Hispanic | 258 (81%) | 178 (82%) | 274 (73%) | 239 (76%) |
| White, Hispanic | 13 (4%) | 7 (3%) | 6 (2%) | 4 (1%) |
| Multiracial, non-Hispanic | 14 (4%) | 10 (5%) | 25 (7%) | 17 (5%) |
| Asian, non-Hispanic | 5 (2%) | 4 (2%) | 26 (7%) | 17 (5%) |
| Other | 7 (2%) | 4 (2%) | 7 (2%) | 6 (2%) |
| Not reported | 20 (6%) | 15 (7%) | 39 (10%) | 33 (10%) |
| Household annual income: N (%) |  |  |  |  |
| Less than $25,000 | 11 (3%) | 9 (4%) | 6 (2%) | 5 (2%) |
| $25,001 - $50,000 | 33 (10%) | 21 (10%) | 17 (5%) | 14 (4%) |
| $50,001 - $75,000 | 64 (20%) | 39 (18%) | 43 (11%) | 39 (12%) |
| $75,001 - $125,000 | 98 (31%) | 72 (33%) | 118 (31%) | 105 (33%) |
| More than $150,000 | 111 (35%) | 77 (35%) | 192 (51%) | 153 (48%) |
| Not Reported | 0 (0%) | 0 (0%) | 1 (0%) | 0 (0%) |
| Household educational attainment: N (%) |  |  |  |  |
| High school diploma | 8 (3%) | 6 (3%) | 8 (2%) | 8 (3%) |
| Some college/Associate’s degree | 43 (14%) | 26 (12%) | 32 (8%) | 28 (9%) |
| College/Bachelor’s degree | 92 (29%) | 60 (28%) | 167 (44%) | 140 (44%) |
| Advanced degree (MA, MD, PhD, etc.) | 165 (52%) | 119 (55%) | 156 (41%) | 129 (41%) |
| Other | 5 (2%) | 4 (2%) | 2 (1%) | 1 (0%) |
| Not reported | 4 (1%) | 3 (1%) | 12 (3%) | 10 (3%) |
| Household political ideology (1-7 scale): N (%) |  |  |  |  |
| Liberal (1-2) | 274 (86%) | 184 (84%) | 239 (63%) | 202 (64%) |
| Moderate (3-5) | 40 (13%) | 32 (15%) | 122 (32%) | 104 (33%) |
| Conservative (6-7) | 2 (1%) | 2 (1%) | 12 (3%) | 10 (3%) |
| Other | 1 (0%) | 0 (0%) | 2 (1%) | 0 (0%) |
| Not reported | 0 (0%) | 0 (0%) | 2 (1%) | 0 (0%) |
| Geographic location (at recruitment): N (%) |  |  |  |  |
| US: Northeast | 37 (12%) | 22 (10%) | 13 (3%) | 0 (0%) |
| US: Midwest | 65 (21%) | 50 (23%) | 0 (0%) | 0 (0%) |
| US: South | 57 (18%) | 39 (18%) | 0 (0%) | 0 (0%) |
| US: West | 152 (48%) | 102 (47%) | 364 (97%) | 316 (100%) |
| Canada | 6 (2%) | 5 (2%) | 0 (0%) | 0 (0%) |
| Primary parent gender: N (%) |  |  |  |  |
| Man | 25 (8%) | 21 (10%) | 34 (9%) | 29 (9%) |
| Woman | 276 (87%) | 187 (86%) | 329 (87%) | 276 (87%) |
| Nonbinary or other | 16 (5%) | 10 (5%) | 10 (3%) | 8 (3%) |
| Not reported | 0 (0%) | 0 (0%) | 4 (1%) | 3 (1%) |

**Table S2**

*Characteristics Regarding Recruitment and Participation of Each Group, , Including Column with 316 “Original” Recruited as Cisgender Participants*

|  | | Recruited as Transgender | Recruited as  Siblings | Recruited as  Cisgender (all) | Recruited as Cisgender (orig) |
| --- | --- | --- | --- | --- | --- |
| Total N (youths) | | 317 | 218 | 377 | 316 |
| Recruitment method | | Conferences, camps, gender clinics, word of mouth, online recruitment through lab website | Conferences, camps, gender clinics, word of mouth, online recruitment through lab website | Participant databases at the University of Washington and Princeton University | Participant databases at the University of Washington |
| Number of visits in study: M (SD, range) | | 5.88 (*SD* = 1.84, *range:* 1-9) | 4.64 (*SD* = 1.89, *range:* 1-9) | 4.62 (*SD* = 2.02, *range:* 1-9) | 4.99 (*SD* = 1.90, *range*: 1-9) |
| Youth age at first visit: M (SD, range) | | 8.07 (*SD*=2.36, *range:* 3-12) | 7.79 (*SD*=2.55, *range:* 3-14) | 8.36 (*SD*=2.37, *range:* 3-14) | 8.10 (*SD* = 2.35, *range*: 3-13) |
| Year of first participation | 2013-2017: N (%) | 317 (100.0%) | 201 (92.2%) | 319 (84.6%) | 307 (97.2%) |
|  | 2018-2023: N (%) | 0 (0.0%) | 17 (7.8%) | 58 (15.4%) | 9 (2.8%) |
| Average time (years) between initial and most recent visit: *M* (SD, range) | | 7.03 (*SD*=1.92, *range:* 0-10.25) | 6.20 (*SD*=2.42, *range:* 0-10.33) | 5.42 (*SD*=2.74, *range:* 0-9.92) | 5.89 (*SD*=2.55, *range:* 0-9.92) |
| Participants with a study visit since 2020: N (%) | | 300 (95%) | 200 (92%) | 311 (82%) | 261 (83%) |
| Attrition | Youth opted out of study: N (%) | 4 (1.3%) | 2 (0.9%) | 16 (4.2%) | 16 (5.1%) |
|  | Youth deceased: N (%) | 0 (0.0%) | 2 (0.9%) | 0 (0.0%) | 0 (0.0%) |
|  | Youth lost to contact: N (%) | 2 (0.6%) | 2 (0.9%) | 0 (0.0%) | 0 (0.0%) |

***Gender and Sexual Orientation Outcomes Among “Original” 316 Recruited as Cisgender Siblings***

Table S3 and Figure S1 show current gender identity outcomes among the “original” 316 *Recruited as Cisgender* participants; Table S4 shows current sexual orientation outcomes.

Outcomes were extremely similar between the “original” 316 *Recruited as Cisgender* participants and the entire *Recruited as Cisgender* group.

**Table S3**

*Current Youth-Reported Identities, With Original Recruited as Cisgender Participants*

| Recruitment group | Recruitment gender | Total N | N with codable follow-up (% of total) | N (% of youths with codable follow-ups) | | | Mean age at first visit (years) | Mean age at most recent codable report (years) |
| --- | --- | --- | --- | --- | --- | --- | --- | --- |
|  |  |  |  | Boy | Gender diverse | Girl |  |  |
| Recruited as Transgender | Boy | 109 | 98 (89.9%) | 87 (88.8%) | 7 (7.1%) | 4 (4.1%) | 8.6 | 15.6 |
|  | Girl | 208 | 185 (88.9%) | 7 (3.8%) | 17 (9.2%) | 161 (87.0%) | 7.6 | 14.5 |
| Recruited as Cisgender (Full Sample) | Boy | 128 | 97 (75.8%) | 88 (90.7%) | 2 (2.1%) | 7 (7.2%) | 9.0 | 15.1 |
|  | Girl | 249 | 188 (75.5%) | 4 (2.1%) | 15 (8.0%) | 169 (89.9%) | 8.2 | 14.5 |
| Recruited as Cisgender (Originals Only) | Boy | 109 | 87 (79.8%) | 79 (90.8%) | 2 (2.3%) | 6 (6.9%) | 8.9 | 15.3 |
|  | Girl | 207 | 159 (76.8%) | 4 (2.5%) | 14 (8.8%) | 141 (88.7%) | 7.9 | 14.6 |
| Recruited as Siblings | Boy | 125 | 99 (79.2%) | 90 (90.9%) | 5 (5.1%) | 4 (4.0%) | 7.5 | 13.7 |
|  | Girl | 93 | 78 (83.9%) | 2 (2.6%) | 16 (20.5%) | 60 (76.9%) | 7.7 | 13.8 |

**Figure S1**

*Most Recent Gender Identity According to Youth Report, Among Youth With At Least One Codable Follow-Up Report, Including “Original” 316 Recruited as Cisgender Participants*


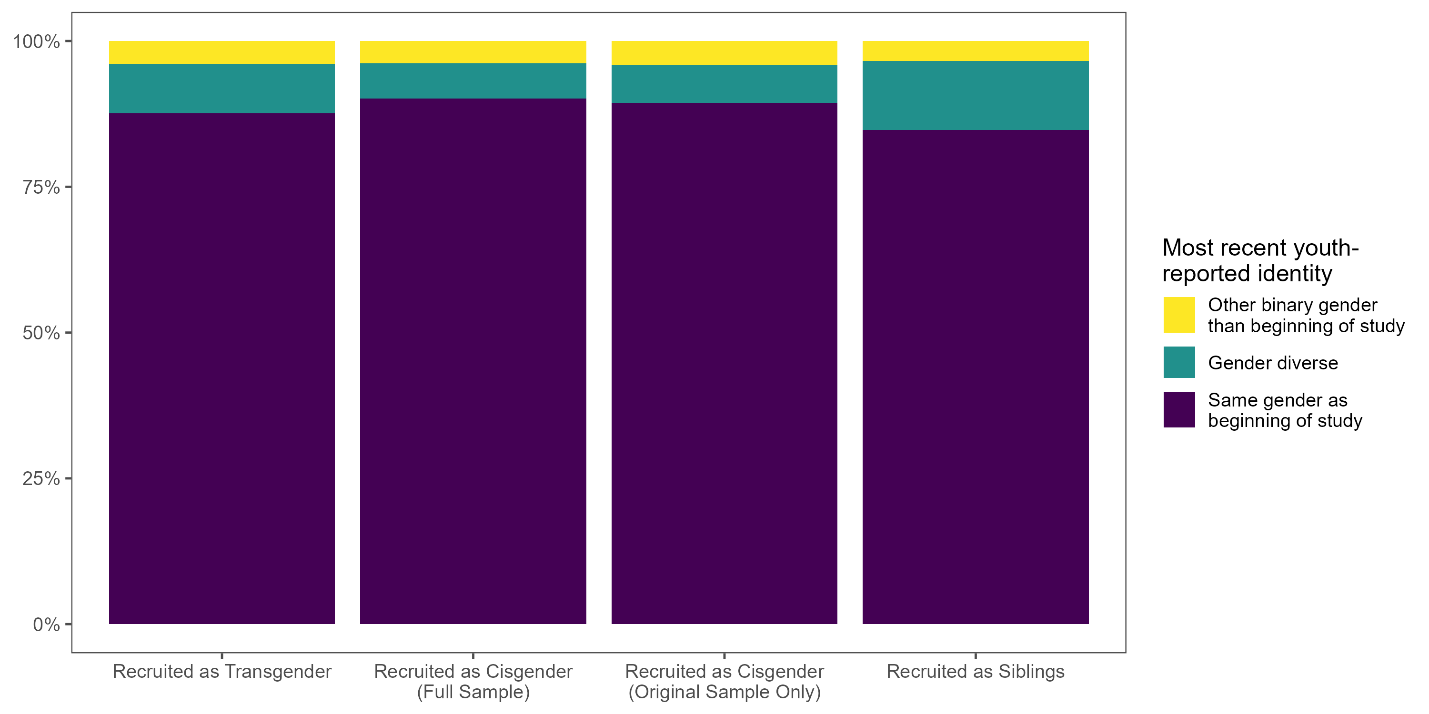


**Table S4**

*Target of Attraction as Indicated on Youths’ Most Recent Online Survey Visits, Including “Original” 316 Recruited as Cisgender Participants*

| Recruitment group | Recruitment Gender | N with report | Mean age (years) at visit | Youth expressing interest in: N (% of those with a report) | | | | | | N : No report on online survey |
| --- | --- | --- | --- | --- | --- | --- | --- | --- | --- | --- |
|  |  |  |  | Only boys | Mostly boys | Both boys and girls | Mostly girls | Only girls | No interest expressed |  |
| Recruited as Transgender | Boy | 84 | 16.2 | 7 (8.3%) | 9 (10.7%) | 25 (29.8%) | 9 (10.7%) | 30 (35.7%) | 4 (4.8%) | 25 |
|  | Girl | 150 | 15.3 | 48 (32.0%) | 10 (6.7%) | 38 (25.3%) | 11 (7.3%) | 27 (18.0%) | 16 (10.7%) | 58 |
| Recruited as Cisgender | Boy | 80 | 16 | 1 (1.2%) | 2 (2.5%) | 8 (10.0%) | 5 (6.2%) | 59 (73.8%) | 5 (6.2%) | 48 |
|  | Girl | 154 | 15.3 | 76 (49.4%) | 20 (13.0%) | 38 (24.7%) | 5 (3.2%) | 8 (5.2%) | 7 (4.5%) | 95 |
| Recruited as Cisgender - Original Sample | Boy | 72 | 16.2 | 1 (1.4%) | 2 (2.8%) | 7 (9.7%) | 5 (6.9%) | 53 (73.6%) | 4 (5.6%) | 37 |
|  | Girl | 129 | 15.5 | 60 (46.5%) | 17 (13.2%) | 33 (25.6%) | 5 (3.9%) | 8 (6.2%) | 6 (4.7%) | 78 |
| Recruited as Siblings | Boy | 74 | 15.3 | 2 (2.7%) | 1 (1.4%) | 10 (13.5%) | 7 (9.5%) | 52 (70.3%) | 2 (2.7%) | 51 |
|  | Girl | 54 | 15.8 | 21 (38.9%) | 2 (3.7%) | 18 (33.3%) | 3 (5.6%) | 6 (11.1%) | 4 (7.4%) | 39 |

**Supporting Information for Chapter III:**

**Assessing Youth- and Parent-Reported Gender at Each Visit**

**Dropped Gender Identity Measure (Superseded Version of Measure 1)**

The research team initially included a different version of Measure 1, in which youth were asked “what do you feel like you are on the inside?” with six response options presented simultaneously: boy, girl, neither, both, it changes over time, and I don’t know. The simultaneous presentation of all six possible answers is the central difference between this measure and Measure 1. The measure was replaced because team members reported that many youth – especially young children – were overwhelmed by having that many options to consider at once. The team felt this encouraged youth to pick answers from later in the list (i.e., gender diverse answers) simply because they had forgotten the initial answers (i.e., binary answers).

Since there are 511 visits which did have this measure, we were able to follow up on this analytically, looking at responses to both the dropped measure and Measure 1 among youth ages 3-14 (the age range of youth who had ever completed the dropped measure). We classified each response as binary (boy, girl) or gender diverse (neither, both, it changes over time, and I don’t know). We then used a logistic regression to test whether participant age, the measure used, and the interaction of these factors predicted the likelihood of making a binary response (vs. a gender diverse response; we first attempted this model including a random intercept for participant to account for repeated participation – as that model did not converge, we report the results of a non-nested analysis). The effect of age on binary (vs. gender diverse) responding differed significantly by measure (OR = 1.21, CI = [1.08 – 1.35], *p* = 0.001), such that age predicted more binary responding on the dropped measure (OR = 1.16, CI = [1.06 – 1.28], *p* = 0.002) but not on Measure 1 (OR = 0.96, CI = [0.91 – 1.02], = 0.179).

**Table S5**

*Age-Specific Proportion of Binary Responses to Dropped Measure and Measure 1*

| Measure | 3-5 Year Olds | 6-8 Year Olds | 9-11 Year Olds | 12-14 Year Olds |
| --- | --- | --- | --- | --- |
| Dropped Measure | 78.35%  N_visits_ = 97 | 74.36%  N_visits_ = 195 | 85.38%  N_visits_ = 171 | 95.83%  N_visits_ = 48 |
| Measure 1 | 87.96%  N_visits_ = 108 | 88.92%  N_visits_ = 370 | 91.23%  N_visits_ = 593 | 86.85%  N_visits_ = 631 |

**Detailed Information on Participant Responses to Individual Gender Measures**

***Youth-Report Measures***

Here, we report descriptive statistics for youths’ responses to Measures 1-6 (Chapter III).

**Table S6**

*Breakdown of Responses on Measure 1 (Three-Item Multiple Choice)*

| Participant group | Visits included | Total N | Mean age (years) | N (%) | | | | | |
| --- | --- | --- | --- | --- | --- | --- | --- | --- | --- |
|  |  |  |  | Boy | Girl | Gender diverse answers | | | |
|  |  |  |  |  |  | Both | Neither | Changes | IDK |
| Recruited as Transgender | Participants’ first time completing measure | 301 visits from 301 participants | 10.1 | 99  (32.9%) | 168 (55.8%) | 13 (4.3%) | 6  (2.0%) | 4  (1.3%) | 11 (3.7%) |
|  | All follow-up visits | 609 visits from 254 participants | 13.6 | 168 (27.6%) | 354 (58.1%) | 15 (2.5%) | 15 (2.5%) | 29 (4.8%) | 28 (4.6%) |
| Recruited as Cisgender | Participants’ first time completing measure | 330 visits from 330 participants | 10 | 108 (32.7%) | 198 (60.0%) | 6  (1.8%) | 2  (0.6%) | 6  (1.8%) | 10 (3.0%) |
|  | All follow-up visits | 577 visits from 257 participants | 13.8 | 157 (27.2%) | 361 (62.6%) | 4  (0.7%) | 16 (2.8%) | 21 (3.6%) | 18 (3.1%) |
| Recruited as Siblings | Participants’ first time completing measure | 200 visits from 200 participants | 9.6 | 104 (52.0%) | 75  (37.5%) | 8  (4.0%) | 3  (1.5%) | 5  (2.5%) | 5  (2.5%) |
|  | All follow-up visits | 285 visits from 147 participants | 13.3 | 147 (51.6%) | 101 (35.4%) | 4  (1.4%) | 10 (3.5%) | 7  (2.5%) | 16 (5.6%) |

**Table S7**

*Youth Responses on Measure 2 (Six-Option Multiple Choice Item Assessing Gender Identity)*

| Participant group | Total N | Mean age (years) | N (%) | | | | | |
| --- | --- | --- | --- | --- | --- | --- | --- | --- |
|  |  |  | Boy | Girl | Gender diverse answers | | | Prefer other term |
|  |  |  |  |  | Gender fluid | Nonbinary | Agender |  |
| Recruited as Transgender | 59 | 14.5 | 28 (47.5%) | 27 (45.8%) | 0 (0%) | 2 (3.4%) | 0 (0%) | 2 (3.4%) |
| Recruited as Cisgender | 52 | 14.5 | 24 (46.2%) | 25 (48.1%) | 0 (0%) | 0 (0%) | 2 (3.8%) | 1 (1.9%) |

*Note.* Participants in the *Recruited as Siblings* group did not receive this measure.

**Table S8**

*Youth Responses on Measure 3 (Seven-Option Multiple Choice Item Assessing Gender Identity, Focusing on Modality)*

| Participant group ^a^ | Total N | Mean age (years) | N (%) | | | | | | |
| --- | --- | --- | --- | --- | --- | --- | --- | --- | --- |
|  |  |  | Transgender (Did not receive a code) | Cisgender (Coded as gender associated with birth sex)^b^ | Gender diverse answers | | | | Prefer other term |
|  |  |  |  |  | Nonbinary | Agender | Gender nonconforming | Gender Fluid |  |
| Recruited as Transgender | 56 | 14.5 | 48 (85.7%) | 3 (5.4%) | 1 (1.8%) | 0 (0%) | 0 (0%) | 1 (1.8%) | 3 (5.4%) |
| Recruited as Cisgender | 44 | 14.5 | 1 (2.3%) | 37 (84.1%) | 0 (0%) | 2 (4.5%) | 0 (0%) | 0 (0%) | 4 (9.1%) |

*Notes.*

a. Participants in the *Recruited as Siblings* group did not receive this measure.

b. Three participants in the *Recruited as Transgender* group selected “Cisgender”, creating a within-visit conflict with their responses on Measure 2 (meaning that qualitative coders reviewed all three of these visits). On all three of these visits, coders determined that the youths were actually binary transgender, and that their selection of “Cisgender” on Measure 3 did not accord with our intended meaning of the term.

**Table S9**

*Percentage of Youths Who Selected Various Options on Measure 4 (Yes/No Questions)*

| Participant group | Visits included | Total N | Mean age (years) |  |  | | N (%) | | | | |
| --- | --- | --- | --- | --- | --- | --- | --- | --- | --- | --- | --- |
|  |  |  |  | Boy | Girl | Boy-Expansive | Girl-Expansive | Gender diverse answers | | | Response could not be coded |
|  |  |  |  |  |  |  |  | Nonbinary | Girl and  Boy | Girl and  Boy and  Nonbinary |  |
| Recruited as Transgender | Participants’ first time completing measure | 265 visits from 265 participants | 13.8 | 82 (30.9%) | 140 (52.8%) | 5 (1.9%) | 5 (1.9%) | 18 (6.8%) | 2 (0.8%) | 4 (1.5%) | 9 (3.4%) |
|  | All follow-up visits | 291 visits from 193 participants | 15.4 | 76 (26.1%) | 173 (59.5%) | 5 (1.7%) | 11 (3.8%) | 16 (5.5%) | 1 (0.3%) | 2 (0.7%) | 7 (2.4%) |
| Recruited as Cisgender | Participants’ first time completing measure | 264 visits from 264 participants | 13.7 | 80 (30.3%) | 152 (57.6%) | 0 (0.0%) | 6 (2.3%) | 15 (5.7%) | 3 (1.1%) | 0 (0.0%) | 8 (3.0%) |
|  | All follow-up visits | 288 visits from 190 participants | 15.4 | 79 (27.4%) | 172 (59.7%) | 3 (1.0%) | 5 (1.7%) | 14 (4.9%) | 1 (0.3%) | 3 (1.0%) | 11 (3.8%) |
| Recruited as Siblings | Participants’ first time completing measure | 154 visits from 154 participants | 13.5 | 79 (51.3%) | 51 (33.1%) | 1 (0.6%) | 3 (1.9%) | 10 (6.5%) | 0 (0.0%) | 3 (1.9%) | 7 (4.5%) |
|  | All follow-up visits | 117 visits from 83 participants | 15.4 | 68 (58.1%) | 36 (30.8%) | 0 (0.0%) | 1 (0.9%) | 8 (6.8%) | 0 (0.0%) | 0 (0.0%) | 4 (3.4%) |

**Table S10**

*Summary of Youth Responses on Measure 5 (Select-all-that-Apply Question)*

| Participant group | Visits included | Total N | Mean age (years) | N (%) | | | | | | | | |
| --- | --- | --- | --- | --- | --- | --- | --- | --- | --- | --- | --- | --- |
|  |  |  |  | Agender | Boy/Man | Cisgender | Gender nonconforming | Genderfluid | Girl/woman | Nonbinary | Transgender | Prefer other term |
| Recruited as Transgender | Participants’ first time completing measure | 235 visits from 235 participants | 14.6 | 3 (1.3%) | 73 (31.1%) | 6 (2.6%) | 20 (8.5%) | 17 (7.2%) | 113 (48.1%) | 28 (11.9%) | 206 (87.7%) | 17 (7.2%) |
|  | All follow-up visits | 222 visits from 152 participants | 16.2 | 7 (3.2%) | 62 (27.9%) | 7 (3.2%) | 21 (9.5%) | 15 (6.8%) | 128 (57.7%) | 33 (14.9%) | 186 (83.8%) | 13 (5.9%) |
| Recruited as Cisgender | Participants’ first time completing measure | 233 visits from 233 participants | 14.5 | 6 (2.6%) | 72 (30.9%) | 134 (57.5%) | 18 (7.7%) | 12 (5.2%) | 125 (53.6%) | 16 (6.9%) | 14 (6.0%) | 5 (2.1%) |
|  | All follow-up visits | 226 visits from 151 participants | 16.2 | 8 (3.5%) | 61 (27.0%) | 136 (60.2%) | 31 (13.7%) | 9 (4.0%) | 134 (59.3%) | 20 (8.8%) | 22 (9.7%) | 7 (3.1%) |
| Recruited as Siblings | Participants’ first time completing measure | 128 visits from 128 participants | 14.6 | 6 (4.7%) | 63 (49.2%) | 79 (61.7%) | 8 (6.2%) | 12 (9.4%) | 36 (28.1%) | 13 (10.2%) | 8 (6.2%) | 2 (1.6%) |
|  | All follow-up visits | 108 visits from 78 participants | 15.8 | 2 (1.9%) | 59 (54.6%) | 73 (67.6%) | 13 (12.0%) | 4 (3.7%) | 30 (27.8%) | 8 (7.4%) | 8 (7.4%) | 3 (2.8%) |

**Table S11**

*Recoded Responses to Youth Responses on Measure 5 (Select-all-that-Apply Question)*

| Participant group | Visits included | Total N | Mean age (years) | N (%) | | | | | |
| --- | --- | --- | --- | --- | --- | --- | --- | --- | --- |
|  |  |  |  | Boy | Girl | Boy-expansive | Girl-expansive | Gender Diverse | Response could not be coded |
| Recruited as Transgender | Participants’ first time completing measure | 235 visits from 235 participants | 14.6 | 67 (28.5%) | 101 (43.0%) | 8 (3.4%) | 14 (6.0%) | 18 (7.7%) | 27 (11.5%) |
|  | All follow-up visits | 222 visits from 152 participants | 16.2 | 53 (23.9%) | 114 (51.4%) | 6 (2.7%) | 13 (5.9%) | 19 (8.6%) | 17 (7.7%) |
| Recruited as Cisgender | Participants’ first time completing measure | 233 visits from 233 participants | 14.5 | 69 (29.6%) | 114 (48.9%) | 3 (1.3%) | 11 (4.7%) | 21 (9.0%) | 15 (6.4%) |
|  | All follow-up visits | 226 visits from 151 participants | 16.2 | 55 (24.3%) | 118 (52.2%) | 4 (1.8%) | 15 (6.6%) | 22 (9.7%) | 12 (5.3%) |
| Recruited as Siblings | Participants’ first time completing measure | 128 visits from 128 participants | 14.6 | 61 (47.7%) | 30 (23.4%) | 0 (0.0%) | 5 (3.9%) | 17 (13.3%) | 15 (11.7%) |
|  | All follow-up visits | 108 visits from 78 participants | 15.8 | 53 (49.1%) | 26 (24.1%) | 4 (3.7%) | 2 (1.9%) | 10 (9.3%) | 13 (12.0%) |

**Table S12**

*Coded Participant Responses on Measure 6 (Open-Ended Description of Gender)*

| Participant group | Visits included | Total N | Mean age (years) | N (%) | | | | | |
| --- | --- | --- | --- | --- | --- | --- | --- | --- | --- |
|  |  |  |  | Boy | Boy-expansive | Gender diverse | Girl-expansive | Girl | Uncodable response |
| Recruited as Transgender | Participants’ first time completing measure | 228 visits from 228 participants | 14.7 | 64 (28.1%) | 8 (3.5%) | 20 (8.8%) | 10 (4.4%) | 102 (44.7%) | 24 (10.5%) |
|  | All follow-up visits | 200 visits from 137 participants | 16.2 | 46 (23.0%) | 7 (3.5%) | 18 (9.0%) | 8 (4.0%) | 96 (48.0%) | 25 (12.5%) |
| Recruited as Cisgender | Participants’ first time completing measure | 224 visits from 224 participants | 14.5 | 67 (29.9%) | 0 (0%) | 12 (5.4%) | 6 (2.7%) | 111 (49.6%) | 28 (12.5%) |
|  | All follow-up visits | 207 visits from 142 participants | 16.2 | 48 (23.2%) | 4 (1.9%) | 12 (5.8%) | 9 (4.3%) | 112 (54.1%) | 22 (10.6%) |
| Recruited as Siblings | Participants’ first time completing measure | 123 visits from 123 participants | 14.7 | 56 (45.5%) | 2 (1.6%) | 12 (9.8%) | 4 (3.3%) | 30 (24.4%) | 19 (15.4%) |
|  | All follow-up visits | 96 visits from 72 participants | 15.9 | 38 (39.6%) | 4 (4.2%) | 4 (4.2%) | 2 (2.1%) | 29 (30.2%) | 19 (19.8%) |

***Parent-Report Measures***

Here, we report descriptive statistics for parents’ responses to Measures 7-11.

**Table S13**

*Parents’ Coded Responses to Measure 7 (Three-Option Parent-Report of Youth Gender)*

| Participant group | Visits included | Total N | Youth mean age (years) | N (%) | | | | |
| --- | --- | --- | --- | --- | --- | --- | --- | --- |
|  |  |  |  | Boy | Girl | Girl-expansive | Gender Diverse | Response could not be coded |
| Recruited as Transgender | Participants’ first time completing measure | 316 visits from 316 participants | 8.1 | 109 (34.5%) | 203  (64.2%) | 2  (0.6%) | 2  (0.6%) | 0  (0.0%) |
|  | All follow-up visits | 594 visits from 270 participants | 10.2 | 179 (30.1%) | 392  (66.0%) | 1  (0.2%) | 20  (3.4%) | 2  (0.3%) |
| Recruited as Cisgender | Participants’ first time completing measure | 374 visits from 374 participants | 8.4 | 127 (34.0%) | 247  (66.0%) | 0  (0.0%) | 0  (0.0%) | 0  (0.0%) |
|  | All follow-up visits | 524 visits from 279 participants | 10.3 | 154 (29.4%) | 359  (68.5%) | 1  (0.2%) | 10  (1.9%) | 0  (0.0%) |
| Recruited as Siblings | Participants’ first time completing measure | 217 visits from 217 participants | 7.8 | 124 (57.1%) | 92  (42.4%) | 0  (0.0%) | 1  (0.5%) | 0  (0.0%) |
|  | All follow-up visits | 313 visits from 176 participants | 10 | 176 (56.2%) | 122  (39.0%) | 2  (0.6%) | 11  (3.5%) | 2  (0.6%) |

*Note.* No responses were coded as boy-expansive on this measure.

**Table S14**

*Parents’ Coded Responses on Measure 8 (Six-Option Multiple Choice Item Assessing Youth Gender Identity)*

| Participant group | Visits included | Total N | Mean age (years) | N (%) | | | | | |
| --- | --- | --- | --- | --- | --- | --- | --- | --- | --- |
|  |  |  |  | Boy | Girl | Boy-expansive | Girl-expansive | Gender Diverse | Response could not be coded |
| Recruited as Transgender | Participants’ first time completing measure | 264 visits from 264 participants | 14.1 | 87 (33.0%) | 155 (58.7%) | 2 (0.8%) | 3 (1.1%) | 17 (6.4%) | 0 (0.0%) |
|  | All follow-up visits | 354 visits from 190 participants | 16.4 | 115 (32.5%) | 210 (59.3%) | 1 (0.3%) | 2 (0.6%) | 23 (6.5%) | 3 (0.8%) |
| Recruited as Cisgender | Participants’ first time completing measure | 255 visits from 255 participants | 14 | 85 (33.3%) | 156 (61.2%) | 0 (0.0%) | 3 (1.2%) | 9 (3.5%) | 2 (0.8%) |
|  | All follow-up visits | 315 visits from 177 participants | 16.4 | 112 (35.6%) | 178 (56.5%) | 0 (0.0%) | 1 (0.3%) | 23 (7.3%) | 1 (0.3%) |
| Recruited as Siblings | Participants’ first time completing measure | 150 visits from 150 participants | 14.8 | 87 (58.0%) | 42 (28.0%) | 1 (0.7%) | 1 (0.7%) | 17 (11.3%) | 2 (1.3%) |
|  | All follow-up visits | 149 visits from 94 participants | 16.3 | 80 (53.7%) | 49 (32.9%) | 1 (0.7%) | 0 (0.0%) | 17 (11.4%) | 2 (1.3%) |

**Table S15**

*Parents’ Coded Responses on Measure 9 (Seven-Option Multiple Choice Item Assessing Youth Gender Identity)*

| Participant group | Visits included | N | Mean age (years) | N (%) | | | | | |
| --- | --- | --- | --- | --- | --- | --- | --- | --- | --- |
|  |  |  |  | Boy | Girl | Boy-expansive | Girl-expansive | Gender Diverse | Response could not be coded |
| Recruited as Transgender | Participants’ first time completing measure | 264 visits from 264 participants | 14.1 | 5 (1.9%) | 5 (1.9%) | 1 (0.4%) | 1 (0.4%) | 12 (4.5%) | 240 (90.9%) |
|  | All follow-up visits | 354 visits from 190 participants | 16.4 | 12 (3.4%) | 15 (4.2%) | 0 (0.0%) | 0 (0.0%) | 22 (6.2%) | 305 (86.2%) |
| Recruited as Cisgender | Participants’ first time completing measure | 253 visits from 253 participants | 14.1 | 78 (30.8%) | 138 (54.5%) | 0 (0.0%) | 1 (0.4%) | 17 (6.7%) | 19 (7.5%) |
|  | All follow-up visits | 299 visits from 169 participants | 16.4 | 98 (32.8%) | 153 (51.2%) | 0 (0.0%) | 0 (0.0%) | 23 (7.7%) | 25 (8.4%) |
| Recruited as Siblings | Participants’ first time completing measure | 150 visits from 150 participants | 14.8 | 86 (57.3%) | 41 (27.3%) | 0 (0.0%) | 1 (0.7%) | 18 (12.0%) | 4 (2.7%) |
|  | All follow-up visits | 148 visits from 94 participants | 16.3 | 79 (53.4%) | 48 (32.4%) | 0 (0.0%) | 0 (0.0%) | 17 (11.5%) | 4 (2.7%) |

**Table S16**

*Parents’ Responses to Measure 10: Select-All-That-Apply Question With 12 Options After Recoding*

| Participant group^a^ | Total N | Mean age (years) | N (%) | | | |
| --- | --- | --- | --- | --- | --- | --- |
|  |  |  | Boy | Girl | Gender Diverse | Response could not be coded |
| Recruited as Transgender | 121 | 8.6 | 42 (34.7%) | 70 (57.9%) | 6 (5.0%) | 3 (2.5%) |
| Recruited as Siblings | 57 | 8.5 | 35 (61.4%) | 20 (35.1%) | 0 (0.0%) | 2 (3.5%) |

*Note.* This measure was not asked of parents in the *Recruited as Cisgender* group.

**Table S17**

*Parents’ Coded Responses to Measure 11: Open-Ended Description of Gender*

| Participant group | N | Mean age (years) | N (%) | | | | | |
| --- | --- | --- | --- | --- | --- | --- | --- | --- |
|  |  |  | Boy | Girl | Boy-expansive | Girl-expansive | Gender Diverse | Response could not be coded |
| Recruited as Cisgender | 225 | 9.3 | 64 (28.4%) | 144 (64.0%) | 0  (0.0%) | 1  (0.4%) | 1  (0.4%) | 15  (6.7%) |
| Recruited as Siblings | 109 | 9.4 | 63 (57.8%) | 39  (35.8%) | 1  (0.9%) | 0  (0.0%) | 1  (0.9%) | 5  (4.6%) |

*Note*. Only one participant in the *Recruited as Transgender* group had a parent complete this measure; their response was coded as gender diverse.

**Further Detail on *Continuum*** **Measure**

When this measure was asked in person, participants made a mark on a piece of paper to indicate where they fell on the continuum. Two members of the research team measured this physical distance, converting it to a measurement between (most masculine) 0 and 100 (most feminine). If the two measurements differed by more than 10, a third measurement was made, and the two most similar measurements were used. When this measure was asked digitally, a slider question was used and the participant could move it wherever they wished on a digital line.

**Table S18**

*Scores on Continuum*

| Participant group | Recruitment gender | Visits included | N | Mean age (years) | Mean *Continuum* score (SD) |
| --- | --- | --- | --- | --- | --- |
| Recruited as Transgender | Boy | Participants’ first time completing measure | 102 visits from 102 participants | 11 | 9.1 (13.9) |
|  |  | All follow-up visits | 209 visits from 88 participants | 14.5 | 15.5 (22.5) |
|  | Girl | Participants’ first time completing measure | 193 visits from 193 participants | 10.1 | 83.5 (18.8) |
|  |  | All follow-up visits | 401 visits from 163 participants | 13.6 | 85 (20.1) |
| Recruited as Cisgender | Boy | Participants’ first time completing measure | 112 visits from 112 participants | 10.8 | 11.7 (17.1) |
|  |  | All follow-up visits | 188 visits from 87 participants | 14.5 | 16.2 (27.2) |
|  | Girl | Participants’ first time completing measure | 215 visits from 215 participants | 10.1 | 85.2 (18.3) |
|  |  | All follow-up visits | 399 visits from 171 participants | 13.7 | 86 (20.4) |
| Recruited as Siblings | Boy | Participants’ first time completing measure | 107 visits from 107 participants | 10.2 | 10.3 (18) |
|  |  | All follow-up visits | 140 visits from 73 participants | 14 | 10.8 (18.1) |
|  | Girl | Participants’ first time completing measure | 83 visits from 83 participants | 10.3 | 85.1 (17.7) |
|  |  | All follow-up visits | 113 visits from 61 participants | 12.9 | 79.6 (25.9) |

*Note*. A score of 0 indicates feeling totally like a boy, and 100 indicates feeling totally like a girl.

**Primary and Secondary Parent Agreement**

In the main text of the monograph, we rely on primary parents’ responses. Here, we report the extent to which those responses agree (or disagree) on each parent-report gender measure in instances in which we have simultaneous responses from multiple parents,

***Measure 7: Three-Option Parent-Report.*** In the 731 cases in which both primary and secondary parents answered this measure about the same youth, parents’ responses received the same five-category code 718 times (98.1%).

***Measure 8: Six-Option Parent-Report.*** In the 40 cases in which both primary and secondary parents answered this measure about the same youth, parents’ responses received the same five-category code all 40 times.

***Measure 9: Seven-Option Parent-Report Multiple Choice with Focus on Modality.*** In the 39 cases in which both primary and secondary parents answered this measure about the same youth, parents’ responses received the same five-category 33 times (84.6%); all 6 disagreements involved one or the other parent providing a response that could not be coded.

***Measure 10: Select-all-that-apply parent-report question with 12 options.*** In visits in which both primary and secondary parents responded to Measure 10 contemporaneously about the same youth (N = 61), their responses were coded as the same category in 54 cases (88.5%); 2 out of the 7 discrepancies between primary and secondary parents involved one or the other parent providing a response that could not be coded.

***Measure 11: Open-ended parent-report.*** In visits in which primary and secondary parents responded to Measure 11 contemporaneously about the same youth (N = 60), their responses were coded as the same category in 52 cases (86.7%); 6 out of the 8 discrepancies between primary and secondary parents involved one or the other parent providing a response that could not be coded.

***Overall Gender-at-Visit Codes.*** In visits in which primary and secondary parents gave reports of the youth’s gender-at-visit that were assigned a code in the 5-category scheme (N = 818), their codes were the same in 801 (97.9%) of cases.

**Supporting Information for Chapter IV:**

**Stability and Change in Gender Identity Over Time**

**Measure-Level Stability and Change**

Here, we describe measure-level stability, or the extent to which respondents gave the same response on a particular measure if they answered it at multiple visits. We focus on their first and latest visit at which they responded to a given measure (in cases with more than 2 responses) to provide the longest-term estimate.

***Youth Self-Report***

**Categorical Measures of Identity.** Table S19 shows, for each of the four categorical measures of gender identity that were asked of youth longitudinally, how often a youth’s response on that measure was assigned the same five-category code on their earliest and most recent reports in which they completed the measure (see Chapter III and Supporting Information for details on the measures and the five-category coding scheme). Measures 2 and 3 are not shown in Table S19 because no youth participant completed these measures more than once. Two notable trends emerge in the data. First, across all measures, stability between first and latest visit is the most common pattern for youth in each of the three groups. Second, we see no significant differences between groups on any of these measures in the percentage who remained stable vs. not (see chi-square analyses in the rightmost column of Table S19).

**Table S19**

*Measure-Level Stability on Youth-Report Gender Measures Between First and Most Recent Visit*

| Measure | Recruited as Transgender | | | Recruited as Cisgender | | | Recruited as Siblings | | | Group differences |
| --- | --- | --- | --- | --- | --- | --- | --- | --- | --- | --- |
|  | Percentage stable | Mean years elapsed (SD) | N | Percentage stable | Mean years elapsed (SD) | N | Percentage stable | Mean years elapsed (SD) | N |  |
| Measure 1: Three-option multiple-choice | 82.7% | 5.2 (1.9) | 254 | 82.9% | 4.8 (2) | 257 | 79.6% | 4.7 (2) | 147 | χ^2^(2) = 0.79, *p* = .67 |
| Measure 4: Yes-or-no questions | 88.2% | 1.8 (0.5) | 186 | 89.0% | 1.8 (0.5) | 182 | 93.6% | 1.6 (0.6) | 78 | χ^2^(2) = 1.78, *p* = .41 |
| Measure 5: Select-all-that-apply question | 82.0% | 1.6 (0.5) | 128 | 80.7% | 1.6 (0.5) | 140 | 86.6% | 1.4 (0.5) | 67 | χ^2^(2) = 1.09, *p* = .58 |
| Measure 6: Open-ended description of gender | 90.7% | 1.6 (0.5) | 118 | 86.7% | 1.6 (0.5) | 120 | 88.9% | 1.4 (0.6) | 54 | χ^2^(2) = 0.96, *p* = .62 |

***Continuum* Measure.** On the *Continuum* measure, youth were asked to indicate how they felt on a continuous 0-100 scale, with 0 representing feeling totally like a boy, and 100 representing feeling totally like a girl (see Chapter III and its Supporting Information for details about this measure). On average, youth were 10.1 years old at their earliest report of *Continuum*, and 4.6 years had elapsed between youths’ earliest and most recent reports. To compare groups in their levels of stability and change on *Continuum*, we fit a linear mixed effects model predicting participants’ absolute value of change between their earliest and most recent reports of *Continuum* from fixed effects of (a) *recruitment group* and (b) a continuous control variable of months between earliest and most recent reports on which *Continuum* was reported; we also included a random intercept for each family to account for nonindependence introduced by multiple siblings from the same family in the dataset. We predicted absolute-value change so that participants who changed to feel more girl-like would not offset participants who changed to feel more boy-like. The aforementioned model showed that the three *recruitment groups* did not differ in their absolute-value change on the continuum measure, Wald χ^2^(2) = 0.48, *p* = .789; however, time elapsed between earliest and most recent continuum report was a small but significant predictor of absolute-value change (Wald χ^2^(2) = 5.67, *p* = .017), such that each additional year between a youth’s earliest and most recent continuum report was associated with an additional 1.04 points of change (out of a possible 100).

**Figure S2**

*Density Curves of Per-Participant Absolute Value Change on Continuum, Earliest to Latest*


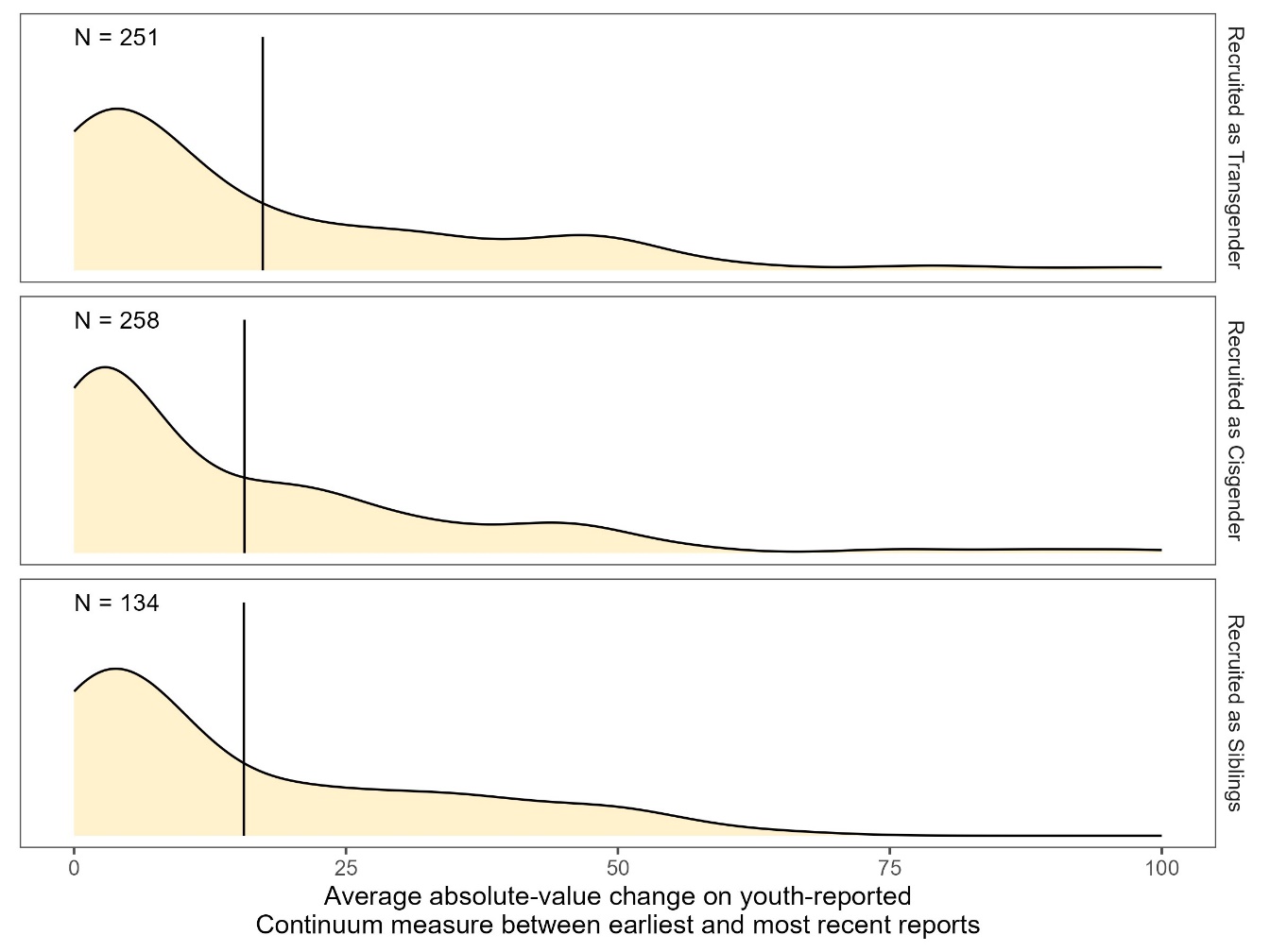


*Note*. Solid vertical lines represent group means; each youth participant who gave more than one response on *Continuum* is represented once in the above figure.

***Primary Parent Report***

***Categorical Measures of Identity.*** Table S20 shows, for each categorical measure about youths’ gender identities that was asked of parents longitudinally, how often a primary parent’s response on that measure was assigned the same five-category code on the earliest and most recent reports in which they completed the measure (See Chapter III for details on the measures and the five-category coding scheme). As with the youth-reported measures, primary parents gave responses that were assigned the same code on their first and latest instances completing the measures in a majority of cases. Further, parents from the three *recruitment group*s did not show differing levels of stability and/or change on Measure 7 and Measure 8 (see “Group differences” column in Table S20). On Measure 9, primary parents in the *Recruited as Transgender* group showed relatively more longitudinal change in part due to the relatively small number in that group who gave more than one codable response to the measure across visits. Because we excluded “uncodable” responses from our calculations of stability and change in Table 20, relatively few parent participants in the *Recruited as Transgender* group are included in the table (n = 19; most gave an uncodable response of simply “Transgender” on Measure 9); therefore, this significant difference should be interpreted with caution.

**Table S20**

*Measure-Level Stability on Youth-Report Gender Measures Between First and Latest Visit*

| Measure | Recruited as Transgender | | | Recruited as Cisgender | | | Recruited as Siblings | | | Group differences |
| --- | --- | --- | --- | --- | --- | --- | --- | --- | --- | --- |
|  | % Stable | Mean years elapsed (SD) | N | % Stable | Mean years elapsed (SD) | N | % Stable | Mean years elapsed (SD) | N |  |
| Measure 7: Three-item multiple choice | 93.0% | 4.1 (1.7) | 270 | 96.4% | 3.7 (1.8) | 279 | 94.3% | 3.6 (1.6) | 176 | χ^2^(2) = 3.26, *p* = 0.2 |
| Measure 8: Six-option multiple choice | 92.1% | 2.4 (1.2) | 189 | 92.0% | 2.3 (1.2) | 176 | 94.6% | 1.7 (0.5) | 93 | χ^2^(2) = 0.71, *p* = 0.7 |
| Measure 9: Seven-option multiple choice with focus on modality | 73.7% | 2 (0.9) | 19 | 94.2% | 2.2 (1.2) | 156 | 93.5% | 1.7 (0.5) | 92 | Fisher's exact test, *p* = 0.02 |

**Alternative Analyses on Youth-Reported Stability and Change**

***Defining Change Relative to Recruitment Gender, Coding Expansive Categories as Gender Diverse***

Table S21 shows percentages of youth in each recruitment group who showed stability or change in their gender identity if change is defined relative to the youths’ *recruitment gender* and the expansive categories are recoded as gender diverse.

**Table S21**

*Proportion of Youth Who Have Shown Gender Change Relative to Recruitment Gender at First Visit, expansive Categories Coded as Gender Diverse*

| Recruitment group | Total N | N (% of total) | | N (% of youth with codable follow-up) | | | Mean age at first visit (years) | Mean age at most recent codable report of gender (years) |
| --- | --- | --- | --- | --- | --- | --- | --- | --- |
|  |  | Missing codable follow-up | Has codable follow-up | Stable | Has shown one change | Has shown more than one change |  |  |
| Recruited as Transgender | 317 | 34 (10.7%) | 283 (89.3%) | 211 (74.6%) | 45 (15.9%) | 27 (9.5%) | 8 | 15.3 |
| Recruited as Cisgender | 377 | 92 (24.4%) | 285 (75.6%) | 234 (82.1%) | 28 (9.8%) | 23 (8.1%) | 8.4 | 15.2 |
| Recruited as Siblings | 218 | 41 (18.8%) | 177 (81.2%) | 142 (80.2%) | 26 (14.7%) | 9 (5.1%) | 7.8 | 14.4 |

***Defining Change Relative to Youths’ Earliest Self-Described Gender, Coding Expansive Categories as Binary***

Table S22 shows percentages of youth in each recruitment group who showed stability or change in their gender identity if change is defined relative to the youths’ *earliest self-described gender* and the expansive categories are recoded as binary. In this operationalization of change, we only consider a youth to have changed if (a) they gave 2 or more codable reports of their gender identity across time and (b) they indicated more than one gender identity across time on these reports.

**Table S22**

*Proportion of Youth Who Have Shown Gender Change Relative to Own Earliest Self-Described Gender, expansive Categories Coded as Binary*

| Recruitment group | Total N | N (% of total) | | N (% of youth with 2 or more codable self-reports of gender) | | | Mean age at first codable report | Mean years between first and latest codable report |
| --- | --- | --- | --- | --- | --- | --- | --- | --- |
|  |  | Missing codable follow-up | Has codable follow-up | Stable | Has shown one change | Has shown more than one change |  |  |
| Recruited as Transgender | 317 | 54 (17.0%) | 263 (83.0%) | 210 (79.8%) | 36 (13.7%) | 17 (6.5%) | 9.6 | 15.0 |
| Recruited as Cisgender | 377 | 114 (30.2%) | 263 (69.8%) | 215 (81.7%) | 34 (12.9%) | 14 (5.3%) | 9.9 | 14.8 |
| Recruited as Siblings | 218 | 71 (32.6%) | 147 (67.4%) | 116 (78.9%) | 24 (16.3%) | 7 (4.8%) | 9.2 | 13.9 |

***Defining Change Relative to Youths’ Earliest Self-Described Gender, Coding Expansive Categories as Gender Diverse***

Table S23 shows percentages of youth in each recruitment group who showed stability or change in their gender identity if change is defined relative to the youths’ *earliest self-described gender* and the expansive categories are recoded as gender diverse.

**Table S23**

*Proportion of Youth Who Have Shown Gender Change Relative to Own Earliest Self-Described Gender, expansive Categories Coded as Gender Diverse*

| Recruitment group | Total N | N (% of total) | | N (% of youth with 2 or more codable self-reports of gender) | | | Mean age at first codable report | Mean years between first and latest codable report |
| --- | --- | --- | --- | --- | --- | --- | --- | --- |
|  |  | Missing codable follow-up | Has codable follow-up | Stable | Has shown one change | Has shown more than one change |  |  |
| Recruited as Transgender | 317 | 54 (17.0%) | 263 (83.0%) | 194 (73.8%) | 47 (17.9%) | 22 (8.4%) | 9.6 | 5.4 |
| Recruited as Cisgender | 377 | 114 (30.2%) | 263 (69.8%) | 207 (78.7%) | 39 (14.8%) | 17 (6.5%) | 9.9 | 4.9 |
| Recruited as Siblings | 218 | 71 (32.6%) | 147 (67.4%) | 117 (79.6%) | 23 (15.6%) | 7 (4.8%) | 9.2 | 4.7 |

***Summary of Alternative Inferential Tests of Whether Youth-Reported Stability Differs by Recruitment Group***

Table S24 summarizes results from all four alternative versions of the inferential test of whether youth-reported stability and change differs by *recruitment group*.

**Table S24**

*Alternative Models: Does Youth-Reported Stability Differ by Recruitment Group?*

| Coding of Expansive Gender Categories | Change Coded Relative to: | Effect of *Recruitment Group* (*Recruited as Transgender*, *Recruited as Cisgender*, *Recruited as Siblings*) | | Effect of *Time Elapsed* | | Discrepancies with Main Text |
| --- | --- | --- | --- | --- | --- | --- |
|  |  | Result | Interpretation | Result | Interpretation |  |
| *Expansive* recoded to *Boy* or *Girl* | Recruitment Gender | Wald χ^2^(2) = 2.37, *p* = .305 | No difference | Wald χ^2^(1) = 5.09, *p* = .024 | More time in study associated with more gender change | N/A |
|  | Self-report | Wald χ^2^(2) = 2.60, *p* = .272 |  | Wald χ^2^(1) = 10.19, *p* = .001 |  | None |
| *Expansive* recoded to *Gender Diverse* | Recruitment Gender | Wald χ^2^(2) = 0.69, *p* = .707 |  | Wald χ^2^(1) = 10.52, *p* = .001 |  |  |
|  | Self-report | Wald χ^2^(2) = 1.22, *p* = .542 |  | Wald χ^2^(1) = 11.33, *p <* .001 |  |  |

**Alternative Analyses on Parent-Reported Stability and Change**

***Defining Change Relative To Recruitment Gender, Coding Expansive Categories as Gender Diverse***

Table S25 shows percentages of youth in each *recruitment group* who showed stability or change in their gender identity according to their parents, if expansive categories are recoded as gender diverse.

**Table S25**

*Proportion of Youth Who Have Shown Gender Change Relative to Recruitment Gender at First Visit According to Primary Parent Report, expansive Categories Coded as Gender Diverse*

| Recruitment group | Total N | N (% of total) | | N (% of youth with codable follow-up) | | | Mean age at first visit (years) | Mean years between beginning participation and latest codable report |
| --- | --- | --- | --- | --- | --- | --- | --- | --- |
|  |  | Missing codable follow-up | Has codable follow-up | Stable | Has shown one change | Has shown more than one change |  |  |
| Recruited as Transgender | 317 | 12 (3.8%) | 305 (96.2%) | 261 (85.6%) | 23 (7.5%) | 21 (6.9%) | 8 | 15.1 |
| Recruited as Cisgender | 377 | 30 (8.0%) | 347 (92.0%) | 309 (89.0%) | 20 (5.8%) | 18 (5.2%) | 8.3 | 14.1 |
| Recruited as Siblings | 218 | 10 (4.6%) | 208 (95.4%) | 179 (86.1%) | 22 (10.6%) | 7 (3.4%) | 7.8 | 14.1 |

***Summary of Alternative Inferential Tests of Whether Parent-Reported Stability Differs by Recruitment Group***

Table S26 summarizes results the two alternative versions of the inferential test of whether parent-reported stability and change differs by *recruitment group*.

**Table S26**

*Alternative Models: Does Parent-Reported Stability Differ by Recruitment Group?*

| Coding of Expansive Gender Categories | Effect of *Recruitment Group* (*Recruited as Transgender*, *Recruited as Cisgender*, *Recruited as Siblings*) | | Effect of *Time Elapsed* | | Discrepancies with Main Text |
| --- | --- | --- | --- | --- | --- |
|  | Result | Interpretation | Result | Interpretation |  |
| *Expansive* recoded to *Boy* or *Girl* | Wald χ^2^(2) = 5.50, *p* = .064 | No difference | Wald χ^2^(1) = 3.92, *p* = .048 | More time in study associated with more gender change | N/A |
| *Expansive* recoded to *Gender Diverse* | Wald χ^2^(2) = 1.16, *p* = .559 |  | Wald χ^2^(1) = 3.85, *p* = .050 |  | None |

**Comparing Youth- and Primary Parent-Reported Stability and Change When Expansive Categories Are Coded as Gender Diverse**

In the main manuscript, we tested whether youth and primary parents differed in their reports of stability and change if responses in the expansive categories were recoded as binary responses and found that youth reported more change than their parents (McNemar’s χ^2^(1) = 25.35, *p* < .001). If expansive categories are recoded as gender diverse, we get the same result: youth reported more change than their parents, McNemar’s χ^2^(1) = 35.13, *p* < .001.

**When in Development Does Change Occur? (Expansive Categories Coded as Gender Diverse)**

To investigate whether changes in gender identity – and specifically a person’s first change in identity from their *recruitment gender* – tend to occur at particular ages, we calculated the percentage of follow-up visits at each age in which participants reported change from their *recruitment gender* for the first time. As opposed to the main text, in which expansive categories are considered binary identities, here we show results coding expansive categories as gender diverse. These percentages are shown in Tables S27 (youth report) and S28 (parent report). Note that once a given participant had a first change, their later data were removed from these calculations because they could not, by definition, show a first change after that point. These tables indicate that changes occurred throughout development in all three participant groups.

**Table S27**

*Percent Of Visits at Different Ages in Which Youth Self-Reported First Gender Change, Expansive Categories Coded as Gender Diverse*

| Age (years) | % of visits in age range reporting first gender change (N) | | | |
| --- | --- | --- | --- | --- |
|  | Recruited as Transgender | Recruited as Cisgender | Recruited as Siblings | Total |
| 6-8 | 12.3% (9) | 7.0% (4) | 8.0% (4) | 9.4% (17) |
| 9-11 | 12.5% (24) | 3.6% (6) | 12.7% (8) | 9.0% (38) |
| 12-14 | 11.6% (26) | 10.1% (25) | 9.9% (12) | 10.6% (63) |
| 15-17 | 5.0% (9) | 8.0% (13) | 7.9% (6) | 6.7% (28) |
| 18+ | 6.4% (3) | 5.6% (3) | 8.0% (2) | 6.3% (8) |

*Note.* Figures are only shown for cells in which there were 10 or more total follow-up visits on which youth could have reported first gender change; as a result, ages 3-5 are not shown.

**Table S28**

*Percent Of Visits at Different Ages in Which Parents Reported First Change in Their Child’s Gender, Expansive Categories Coded as Gender Diverse*

| Age (years) | % of visits in age range reporting first gender change (N) | | | |
| --- | --- | --- | --- | --- |
|  | Recruited as Transgender | Recruited as Cisgender | Recruited as Siblings | Total |
| 3-5 | 10.7% (3) | 0.0% (0) | 3.2% (1) | 4.2% (4) |
| 6-8 | 4.3% (6) | 0.0% (0) | 2.3% (3) | 1.9% (9) |
| 9-11 | 3.0% (9) | 2.0% (7) | 4.2% (7) | 2.8% (23) |
| 12-14 | 5.0% (18) | 4.9% (19) | 4.4% (8) | 4.8% (45) |
| 15-17 | 3.0% (7) | 5.1% (10) | 7.8% (10) | 4.8% (27) |
| 18+ | 1.3% (1) | 2.8% (2) | 0.0% (0) | 1.6% (3) |

*Note.* Figures are only shown for cells in which there were 10 or more total follow-up visits on which parents could have reported first gender change for their children.

**Youth’s Current Identities**

***Current Youth-Reported Identities, Expansive Categories Coded as Gender Diverse***

Table S29 shows current identities (expansive categories coded as gender diverse, instead of as boy or girl as in the main text) according to the youth themselves, among the 745 youth (81.7% of the total sample) who have given a codable report of their gender at a follow-up visit.

**Table S29**

*Current Identities, Per Youth Report (Expansive Categories Coded as Gender Diverse)*

| Recruitment group | Recruitment gender | Total N | N with codable follow-up (% of total) | N (% of youth with codable follow-ups) | | | Mean age at first visit (years) | Mean age at latest report (years) |
| --- | --- | --- | --- | --- | --- | --- | --- | --- |
|  |  |  |  | Boy | Gender diverse | Girl |  |  |
| Recruited as Transgender | Boy | 109 | 98 (89.9%) | 80 (81.6%) | 15 (15.3%) | 3 (3.1%) | 8.6 | 15.6 |
|  | Girl | 208 | 185 (88.9%) | 7 (3.8%) | 28 (15.1%) | 150 (81.1%) | 7.6 | 14.5 |
| Recruited as Cisgender | Boy | 128 | 97 (75.8%) | 87 (89.7%) | 4 (4.1%) | 6 (6.2%) | 9.0 | 15.1 |
|  | Girl | 249 | 188 (75.5%) | 2 (1.1%) | 23 (12.2%) | 163 (86.7%) | 8.2 | 14.5 |
| Recruited as Siblings | Boy | 125 | 99 (79.2%) | 89 (89.9%) | 8 (8.1%) | 2 (2.0%) | 7.5 | 13.7 |
|  | Girl | 93 | 78 (83.9%) | 1 (1.3%) | 18 (23.1%) | 59 (75.6%) | 7.7 | 13.8 |

***Summary of Alternative Inferential Tests on Youth-Reported Current Identity by Recruitment Group***

Table S30 summarizes results from the two alternative versions of the inferential test of whether youths in different *recruitment groups* differed in their likelihood of currently being their *recruitment gender*.

**Table S30**

*Alternative Models: Were Youths in Different Recruitment Groups More or Less Likely to Report Currently Being Their Recruitment Gender?*

| Coding of Expansive Gender Categories | Effect of *Recruitment Group* (*Recruited as Transgender*, *Recruited as Cisgender*, *Recruited as Siblings*) | | Effect of *Time Elapsed* | | Discrepancies with Main Text |
| --- | --- | --- | --- | --- | --- |
|  | Result | Interpretation | Result | Interpretation |  |
| *Expansive* recoded to *Boy* or *Girl* | Wald χ^2^(2) = 3.96, *p* = .138 | No difference | Wald χ^2^(1) = 1.25, *p* = .263 | No effect of *Time Elapsed* | N/A |
| *Expansive* recoded to *Boy* or *Girl* | Wald χ^2^(2) = 0.03, *p* = .984 |  | Wald χ^2^(1) = 8.40, *p* = .004 | More time in study associated with more gender change | Significant effect of *Time Elapsed* |

***Current Parent-Reported Identities, Expansive Categories Coded as Gender Diverse***

Table S31 shows current youth identities (expansive categories coded as gender diverse, instead of binary categories as in the main text) according to parents among the 860 youth (94.3% of the total sample) who have given a codable report of their child’s gender at a follow-up visit.

**Table S31**

*Current Youth Identity, Per Parent Report, Expansive Categories Coded as Gender Diverse*

| Recruitment group | Recruitment gender | Total N | N with codable follow-up (% of total) | N (% of youth with codable follow-ups) | | | Mean age at first visit (years) | Mean age at latest report (years) |
| --- | --- | --- | --- | --- | --- | --- | --- | --- |
|  |  |  |  | Boy | Gender diverse | Girl |  |  |
| Recruited as Transgender | Boy | 109 | 106 (97.2%) | 91 (85.8%) | 13 (12.3%) | 2 (1.9%) | 8.6 | 15.6 |
|  | Girl | 208 | 199 (95.7%) | 5 (2.5%) | 11 (5.5%) | 183 (92.0%) | 7.7 | 14.8 |
| Recruited as Cisgender | Boy | 128 | 117 (91.4%) | 107 (91.5%) | 3 (2.6%) | 7 (6.0%) | 8.9 | 14.8 |
|  | Girl | 249 | 230 (92.4%) | 4 (1.7%) | 13 (5.7%) | 213 (92.6%) | 8.0 | 13.8 |
| Recruited as Siblings | Boy | 125 | 120 (96.0%) | 112 (93.3%) | 6 (5.0%) | 2 (1.7%) | 7.7 | 14.2 |
|  | Girl | 93 | 88 (94.6%) | 0 (0%) | 16 (18.2%) | 72 (81.8%) | 7.9 | 14 |

***Summary of Alternative Inferential Tests on Parent-Reported Current Identity by Recruitment Group***

Table S32 summarizes results from the two alternative versions of the inferential test of whether parents of youths in different *recruitment groups* differed in their likelihood of reporting that their child is currently identifying as their *recruitment gender*.

**Table S32**

*Alternative Models: Are Youths in Different Recruitment Groups More or Less Likely to Currently Identify as Their Recruitment Gender, According to Parents?*

| Coding of Expansive Gender Categories | Effect of *Recruitment Group* (*Recruited as Transgender*, *Recruited as Cisgender*, *Recruited as Siblings*) | | Effect of *Time Elapsed* | | Discrepancies with Main Text |
| --- | --- | --- | --- | --- | --- |
|  | Result | Interpretation | Result | Interpretation |  |
| *Expansive* recoded to *Boy* or *Girl* | Wald χ^2^(2) = 2.87, *p* = .238 | No difference | Wald χ^2^(1) = 3.08, *p* = .079 | No effect of *Time Elapsed* | N/A |
| *Expansive* recoded to *Boy* or *Girl* | Wald χ^2^(2) = 3.56, *p* = .169 |  | Wald χ^2^(1) = 2.81, *p* = .094 |  | None |

**Comparing Youth’s Current Identity by Reporter (Youth vs. Parent), Expansive Categories Coded as Gender Diverse**

Did youth and parents systematically differ in their reports of the youth’s identity at the youth’s most recent visit? Of the 745 youth who have ever given a codable self-report of their gender at a follow-up visit, 691 (92.8%) also had a parent give a codable report of the youth’s gender at the same timepoint. Among this subgroup with overlapping participation, Table S33 shows contingency tables of youth’s and parents’ reports of the youth’s identities (expansive categories coded as gender diverse) in *Recruited as Transgender* group (N = 257), *Recruited as Cisgender* group (N = 277), and *Recruited as Siblings* group (N = 156). By far the modal pattern in all three groups was that youth and parents agreed on the youth’s gender identity.

**Table S33**

*Contingency Tables of Youth- and Parent-Reported Current Youth Identity*

|  | Youth Report | Parent Report | | | % Agreement |
| --- | --- | --- | --- | --- | --- |
|  |  | Boy | GD | Girl |  |
| Recruited as Transgender | Boy | 80 | 5 | 0 | 94.6% |
|  | GD | 0 | 11 | 8 |  |
|  | Girl | 0 | 1 | 152 |  |
| Recruited as Cisgender | Boy | 90 | 1 | 0 | 96.4% |
|  | GD | 1 | 9 | 7 |  |
|  | Girl | 0 | 1 | 169 |  |
| Recruited as Siblings | Boy | 81 | 1 | 0 | 94.2% |
|  | GD | 2 | 10 | 3 |  |
|  | Girl | 1 | 2 | 56 |  |

*Note.* Only includes youth who had codable parent- and youth-reports of gender at most recent visit.

**Figures Showing Gender Trajectories for Each Individual Participant: Youth-Report**

Figures S3, S4, and S5 visually depict the self-reported trajectories of all youth in the study, each of whom is represented by one horizontal line. Note that for this set of visualizations, each group is represented in a separate figure (i.e., Figure S3 for *Recruited as Transgender*, Figure S4 for *Recruited as Cisgender*, and Figure S5 for *Recruited as Siblings*), and each figure is organized by youths’ *recruitment gender* and whether their identity was stable or ever changed over the course of the study. The length of each line indicates the amount of time they have been in the study (so far), and where the line falls along the x-axis indicates their ages during the course of the study. Colors correspond to their self-reported gender identity at visits at which they produced codable responses. Grey lines indicate periods following visits in which youths did not give a codable report of gender.

**Figure S3.** *Longitudinal Gender Identity in the Recruited as Transgender Group, Ordered by Recruitment Gender and Stability vs. Change, According to Youth Self-Report*


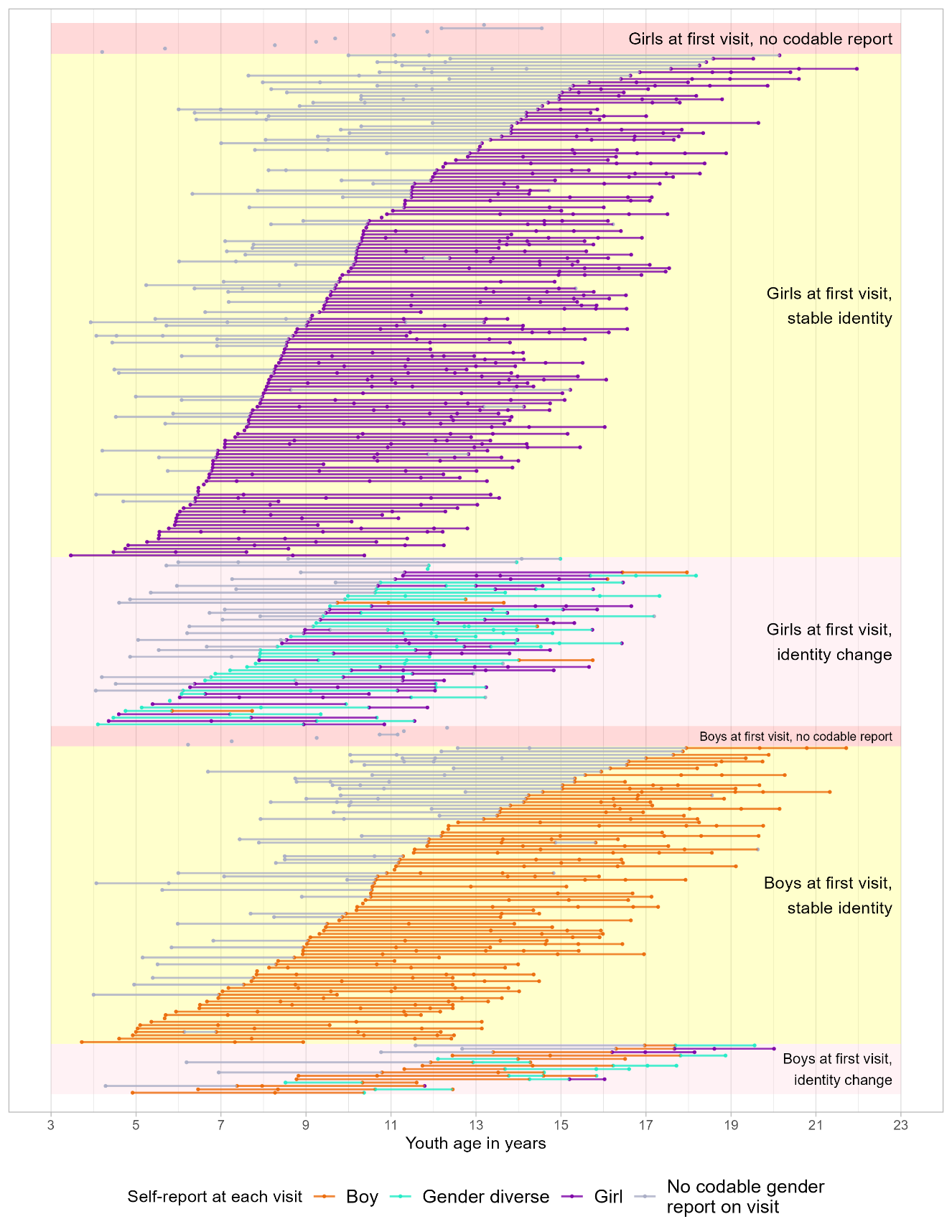


*Note.* Random noise between 0 and 12 months was added to or subtracted from youths’ ages to protect participant privacy. The sequence of presented visits was not altered.

**Figure S4.** *Longitudinal Gender Identity in the Recruited as Cisgender Group, Ordered by Recruitment Gender and Stability vs. Change, According to Youth Self-Report*


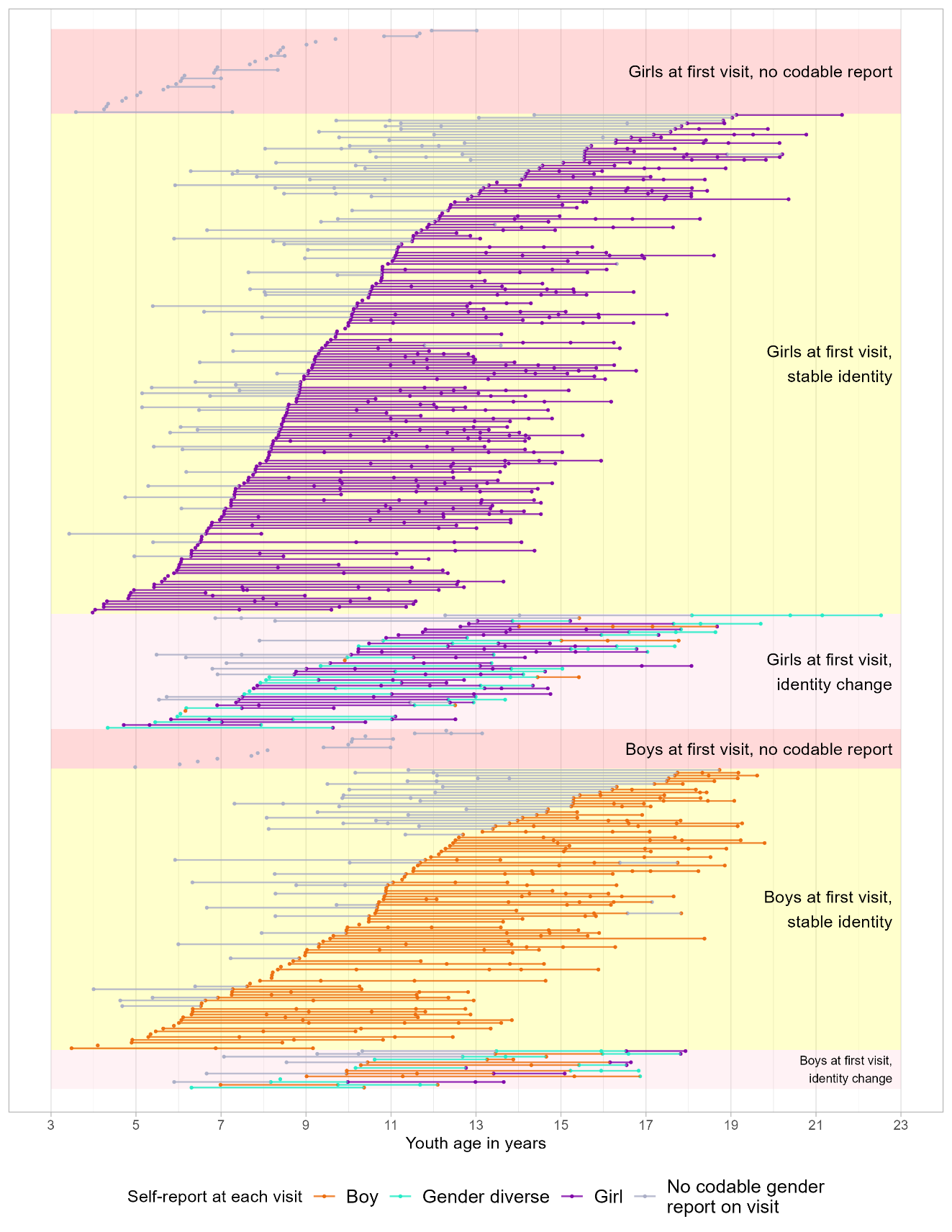


*Note.* Random noise between 0 and 12 months was added to or subtracted from youths’ ages to protect participant privacy. The sequence of presented visits was not altered.

**Figure S5.** *Longitudinal Gender Identity in the Recruited as Siblings Group, Ordered by Recruitment Gender and Stability vs. Change, According to Youth Self-Report*


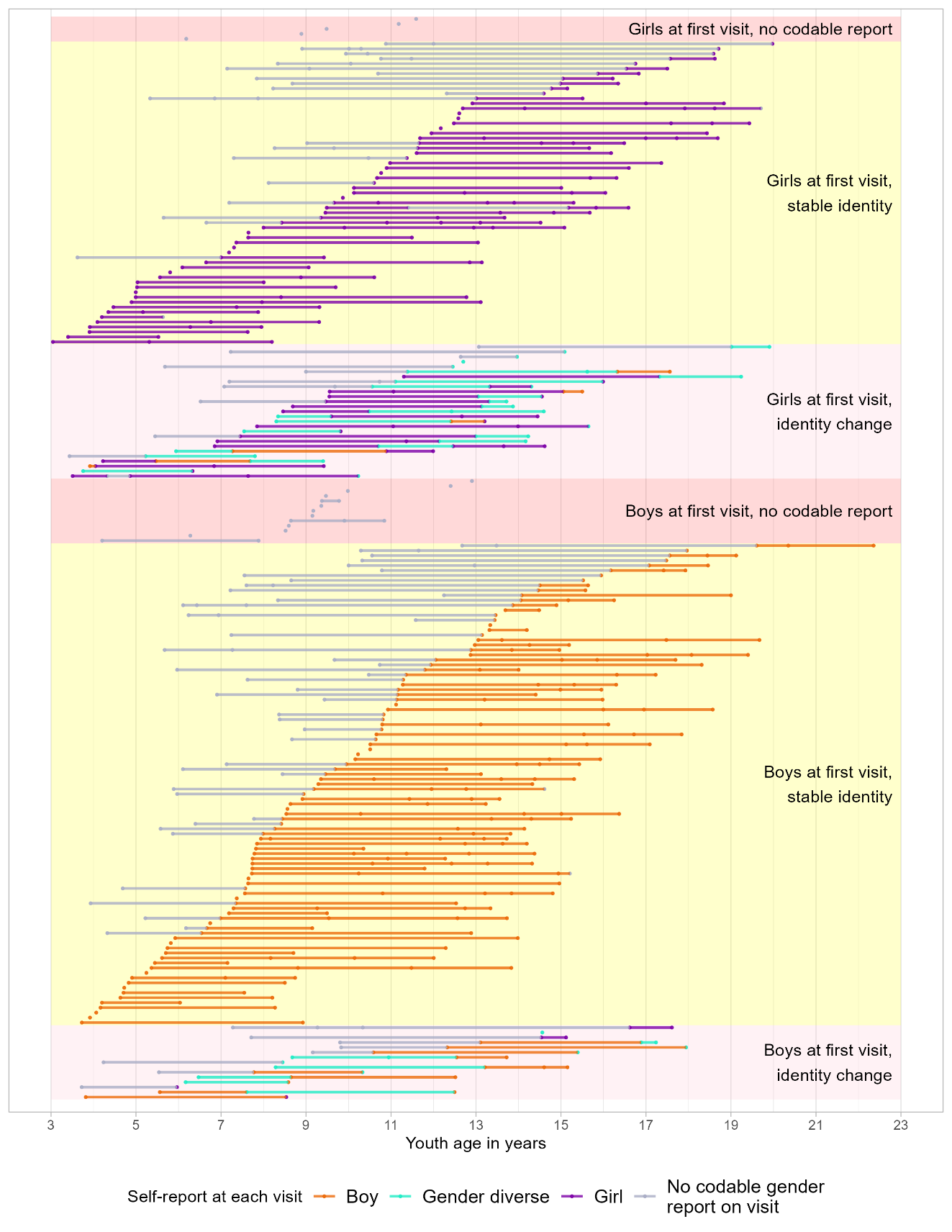


*Note.* Random noise between 0 and 12 months was added to or subtracted from youths’ ages to protect participant privacy. The sequence of presented visits was not altered.

**Figures Showing Gender Trajectories for Each Individual Participant: Parent-Report**

Figures S6, S7, and S8 visually depict the parent-reported trajectories of all youths in the study, each of whom is represented by one horizontal line. The format is identical to that of Figures S3-S5 above.

**Figure S6.** *Longitudinal Gender Identity in the Recruited as Transgender Group According to Primary Parent Report*


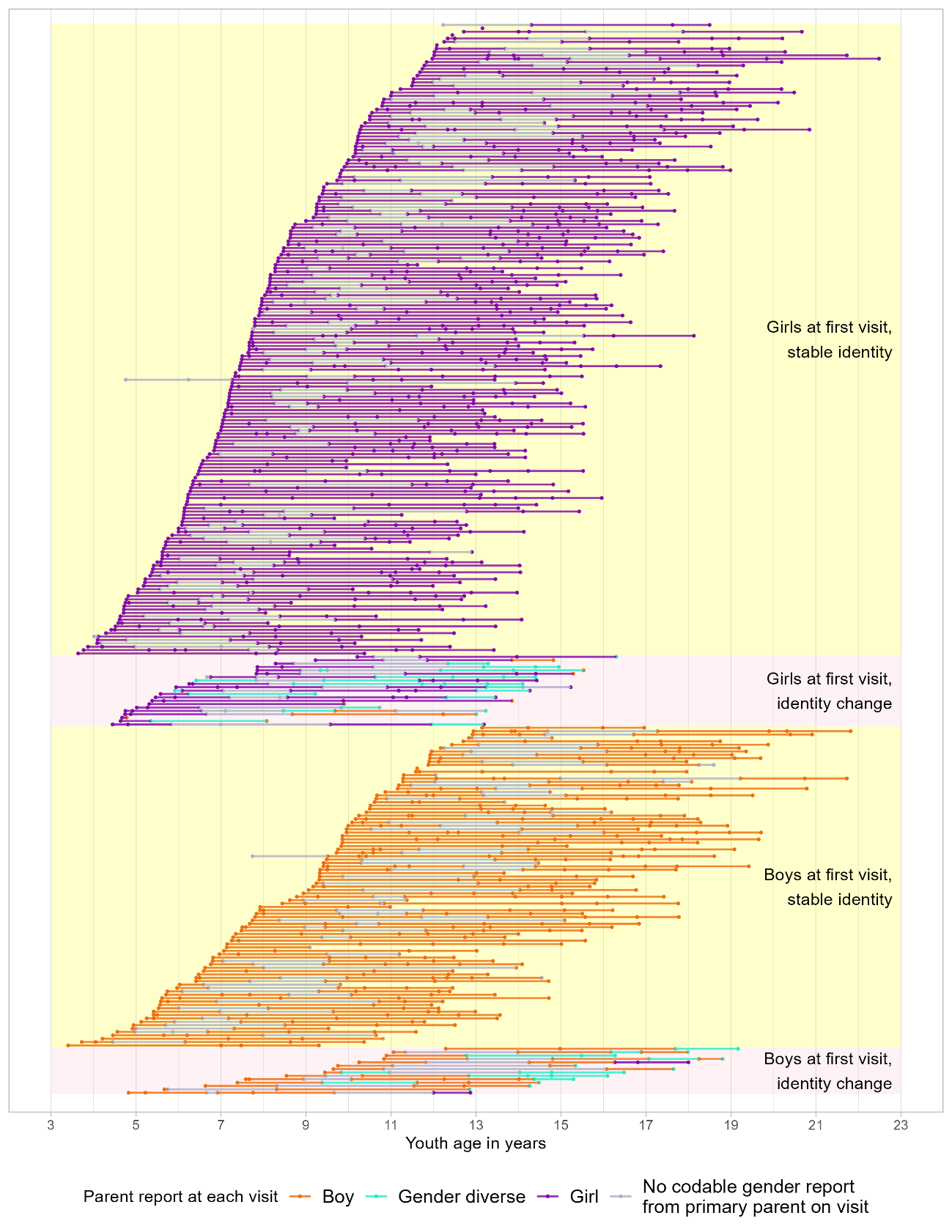


*Note.* Random noise between 0 and 12 months was added to or subtracted from youths’ ages to protect participant privacy. The sequence of presented visits was not altered.

**Figure S7.** *Longitudinal Gender Identity in the Recruited as Cisgender Group According to Primary Parent Report*


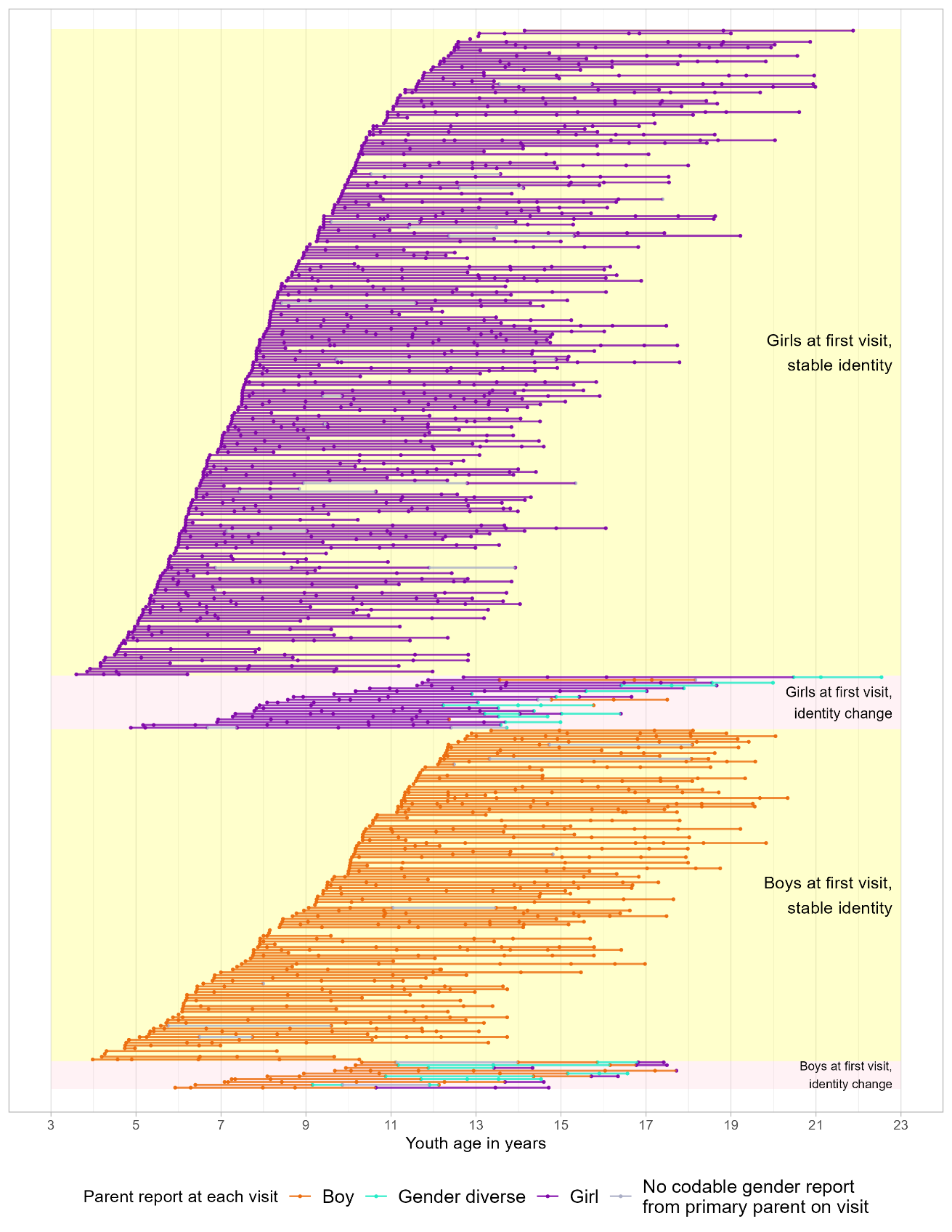


*Note.* Random noise between 0 and 12 months was added to or subtracted from youths’ ages to protect participant privacy. The sequence of presented visits was not altered.

**Figure S8.** *Longitudinal Gender Identity in the Recruited as Siblings Group According to Primary Parent Report*


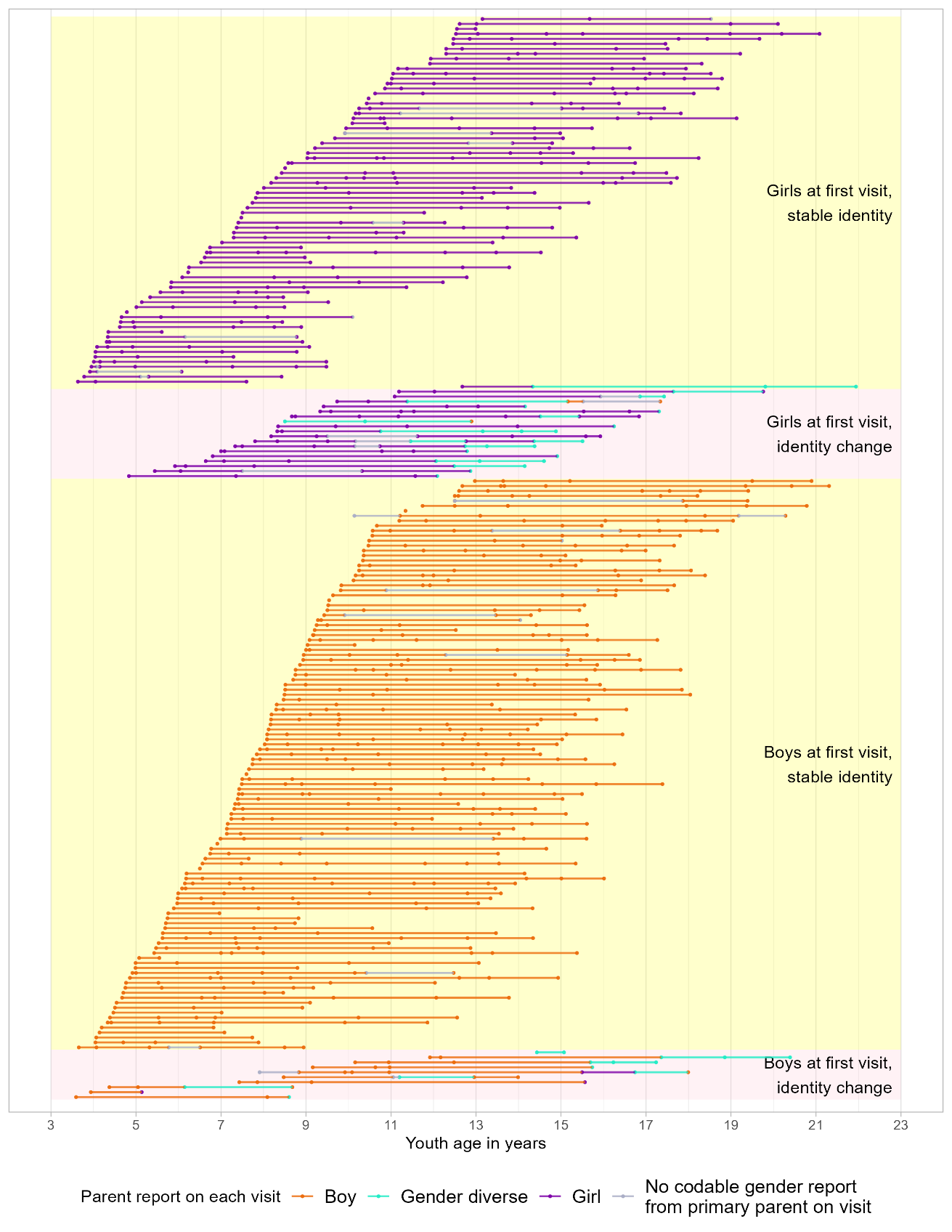


*Note.* Random noise between 0 and 12 months was added to or subtracted from youths’ ages to protect participant privacy. The sequence of presented visits was not altered.

**Supporting Information for Chapter V:**

**Associations Between Childhood Gender Development and Current Gender Identity**

**Additional Information on *Preference* and *Parent Questionnaire* Measures**

***Preference Measure***

In Chapters 5 and 6, we use youths’ gender-typed preferences for toys, clothing, and peers as predictors of most recent gender or sexual orientation. These preferences have been assessed throughout the Trans Youth Project, typically for children under 12, though in infrequent cases, youths aged 12 or 13 received these measures. Results from the original cohort of youths on these measures at their initial visit were reported in Gülgöz et al. (2019).

***Toy Preferences.*** Youths saw four trials in which they were presented with images of five toys ranging from stereotypically masculine to stereotypically feminine and selected which toy from each set they would like to play with. Perceived masculinity and femininity of the toys was determined by pilot testing with a separate group of cisgender children, and toys shown to youths ages 3-7 were different from those shown to youths ages 8-11 to ensure that children saw developmentally appropriate toys. We updated the exact stimuli used in 2021 as the toys in the original study were nearly a decade old, but the format, age ranges, and procedure was the same. Choosing the most feminine toy on a given trial was assigned a score of 1, the moderately feminine toy 0.75, the neutral toy 0.5, the moderately masculine toy 0.25, and the most masculine toy 0; children’s scores on all trials they answered were averaged. Youths had to have answered at least two of the four trials to be counted as having completed the measure (α = 0.77).

***Clothing Preferences.*** Similar to the toy measure, youths saw four trials in which they were presented with images of five outfits that ranged from stereotypically masculine to stereotypically feminine. Norming and scoring of the stimuli, differentiation of stimuli between younger and older children, updating stimuli, and inclusion criteria were all the same as for the toy measure (α = 0.91).

***Peer Preferences.*** Youths saw a series of six trials in which they were shown a boy and a girl and asked to choose who they would like to be friends with the most; youths also saw two filler trials (one with two boys and the other with two girls) that were not used towards scoring youths’ peer gender preferences. To be counted as having answered the peer preference measure, youths had to have answered half (i.e., three or more) of the six boy-girl trials. We derived a score between 0 (always selected boys) and 1 (always selected girls) by calculating the proportion of times they selected the girl.

***Preference Composite Score.*** To derive a single composite preference score (summarized in Table S34), we took the average of youths’ toy, clothing, and peer preference scores (all of which were between 0, representing most masculine, to 1, representing most feminine; α = 0.77).

**Table S34**

*Preference Composite Scores*

| Participant group | Designated gender | Visits included | N | Mean age (years) | Mean Preference Composite score (SD) |
| --- | --- | --- | --- | --- | --- |
| Recruited as Transgender | Boy | Participants’ first time completing measure | 94 visits from 94 participants | 8.3 | 0.19 (0.1) |
|  |  | All follow-up visits | 75 visits from 51 participants | 10 | 0.26 (0.12) |
|  | Girl | Participants’ first time completing measure | 189 visits from 189 participants | 7.6 | 0.77 (0.16) |
|  |  | All follow-up visits | 193 visits from 127 participants | 9.9 | 0.62 (0.18) |
| Recruited as Cisgender | Boy | Participants’ first time completing measure | 106 visits from 106 participants | 8.6 | 0.23 (0.13) |
|  |  | All follow-up visits | 60 visits from 43 participants | 10.1 | 0.22 (0.12) |
|  | Girl | Participants’ first time completing measure | 226 visits from 226 participants | 7.8 | 0.78 (0.15) |
|  |  | All follow-up visits | 170 visits from 120 participants | 10 | 0.73 (0.15) |
| Recruited as Siblings | Boy | Participants’ first time completing measure | 110 visits from 110 participants | 7.6 | 0.24 (0.18) |
|  |  | All follow-up visits | 62 visits from 55 participants | 9.4 | 0.23 (0.12) |
|  | Girl | Participants’ first time completing measure | 82 visits from 82 participants | 7.6 | 0.76 (0.16) |
|  |  | All follow-up visits | 58 visits from 43 participants | 8.9 | 0.69 (0.2) |

*Note.* Scores range from 0 (most masculine) to 1 (most feminine)

***Parent Questionnaire Measure***

In Chapters 5 and 6, we use parents’ reports of youths’ gender-typed preferences and behavior as predictors of most recent gender or sexual identity. We assessed this with a 16-item version of the parent-report Gender Identity Questionnaire for Children (Johnson et al., 2004), which we referred to as *Parent Questionnaire* in the manuscript. Parents were asked about their child’s gender-typed preferences (e.g., for girls or boys as playmates, for masculine or feminine toys; 12 items) and their child’s identity statements (e.g., “I am a boy”; 2 boy-or-girl-specific items in the original measure, 4 items in our measure). Each item has five response options from maximally girl-typical (e.g., the child’s favorite playmates are always girls) to maximally boy-typical (e.g., the child’s favorite playmates are always boys). Some items also have a response indicating the question doesn’t apply (e.g., that the child never plays “house”). We coded these items from 0 (maximally masculine) to 1 (maximally feminine).

The measure also includes two items about a child’s physical comfort with their body (e.g., liking one’s private parts). For *Recruited as Cisgender* and *Recruited as Siblings* youths, being comfortable with one’s body is considered gender-typical of one’s gender group, while discomfort with one’s body is considered gender-atypical (i.e., a cisgender girl who talks about liking her private parts every day is coded a 1 for maximally feminine; a cisgender boy who did the same would be coded a 0 for maximally masculine). This is reversed for *Recruited as Transgender* youths (i.e., a transgender girl who never talks about liking her private parts is coded a 1 for maximally feminine, a transgender boy who did the same would be coded a 0 for maximally masculine). Every time a parent answered this measure, they replied to at least half of the items, and so we include them all (96% answered all 18 questions). We use the mean of responses across all included items (α = 0.93). Table S35 shows scores on this measure broken down by participant group. As can be seen in the table, boys tend to have very low scores and girls tend to have very high scores, providing face validity.

**Table S35**

*Mean scores on Parent-Report Gender Identity Questionnaire for Children*

| Participant group | Designated gender | Visits included | N | Mean age (years) | Mean score (SD) |
| --- | --- | --- | --- | --- | --- |
| Recruited as Transgender | Boy | Participants’ first time completing measure | 106 visits from 106 participants | 9.3 | 0.2 (0.1) |
|  |  | All follow-up visits | 116 visits from 79 participants | 9.9 | 0.2 (0.1) |
|  | Girl | Participants’ first time completing measure | 205 visits from 205 participants | 8.4 | 0.7 (0.1) |
|  |  | All follow-up visits | 286 visits from 166 participants | 9.5 | 0.7 (0.1) |
| Recruited as Cisgender | Boy | Participants’ first time completing measure | 121 visits from 121 participants | 9.2 | 0.3 (0.1) |
|  |  | All follow-up visits | 119 visits from 79 participants | 10.0 | 0.3 (0.1) |
|  | Girl | Participants’ first time completing measure | 233 visits from 233 participants | 8.3 | 0.7 (0.1) |
|  |  | All follow-up visits | 288 visits from 165 participants | 9.2 | 0.7 (0.1) |
| Recruited as Siblings | Boy | Participants’ first time completing measure | 119 visits from 119 participants | 8.3 | 0.3 (0.1) |
|  |  | All follow-up visits | 111 visits from 75 participants | 9.3 | 0.3 (0.1) |
|  | Girl | Participants’ first time completing measure | 88 visits from 88 participants | 8.3 | 0.7 (0.1) |
|  |  | All follow-up visits | 81 visits from 52 participants | 9.2 | 0.7 (0.1) |

*Note.* Scores range from 0 (most masculine) to 1 (most feminine).

***Agreement Between Primary and Secondary Parents on Parent Questionnaire.*** In the 617 cases in which both primary and secondary parents answered *Parent Questionnaire* about the same youth, parents’ responses correlated highly (*r*(615) = 0.94, *p* < .001).

**Alternative Inferential Analyses**

Here, we present parallel results from Chapter V’s inferential tests if we recode the “boy-expansive” and “girl-expansive” categories to “gender diverse”, rather than “boy” and “girl” respectively (as they are in the main text). For all six inferential analyses in the main text, we include a table showing what results we observe when expansive categories are recoded as gender diverse, rather than as boy or girl. In each table, the critical comparison is whether youths who are currently identified as their *recruitment gender* show significantly different early scores on each gender development measure (*Continuum*, *Preference*, or *Parent Questionnaire*) than those who are currently a different gender from their *recruitment gender*.

**Question 1: *Continuum* and Its Relation to Current Gender Identity**

***Boys at Early Continuum***

**Table S36**

*Alternative Models: Early Continuum and Its Relation to Current Gender Identity (Boys at Early Continuum)*

| Coding of Expansive Gender Categories | Omnibus statistic | Pairwise Comparison Between Current Gender Diverse and Current Girls | | Pairwise Comparison Between Current Gender Diverse and Current Boys | | Pairwise Comparison Between Current Girls and Current Boys | | Discrepancies with Main Text |
| --- | --- | --- | --- | --- | --- | --- | --- | --- |
|  |  | Results | Interpretation | Results | Interpretation | Results | Interpretation |  |
| *Expansive* recoded to *Boy* or *Girl* | Wald χ^2^(2) = 10.44, *p* = .005 | *b* = -11.70, *p* = .094 | No difference | *b* = 1.60, *p* = .914 | No difference | *b* = 13.30, *p =* .004 | Current girls more feminine than current boys | N/A |
| *Expansive* recoded to *Gender Diverse* | Wald χ^2^(2) = 12.30, *p* = .005 | *b* = -6.42, *p* = .422 |  | *b* = 6.22, *p* = .106 |  | *b* = 12.64, *p =* .010 |  | None |

***Girls at Early Continuum***

**Table S37**

*Alternative Models: Early Continuum and Its Relation to Current Gender Identity (Girls at Early Continuum)*

| Coding of Expansive Gender Categories | Comparison of Current Girls and Current Boys/Gender Diverse | | Discrepancies with Main Text |
| --- | --- | --- | --- |
|  | Result | Interpretation |  |
| *Expansive* recoded to *Boy* or *Girl* | *b* = -3.99, *p =* .151 | No difference | N/A |
| *Expansive* recoded to *Gender Diverse* | *b* = -2.38, *p* = .318 |  | None |

**Question 2: *Preference* and Its Relation to Current Gender Identity**

***Boy at Early Preference***

**Table S38**

*Alternative Models: Early Preference and Its Relation to Current Gender Identity (Boys at Early Preference)*

| Coding of Expansive Gender Categories | Comparison of Boys and Current Girls/Gender Diverse | | Discrepancies with Main Text |
| --- | --- | --- | --- |
|  | Result | Interpretation |  |
| *Expansive* recoded to *Boy* or *Girl* | *b* = -0.09, *p =* .008 | Current boys more masculine than current girls/gender diverse | N/A |
| *Expansive* recoded to *Gender Diverse* | *b* = -0.10, *p* < .001 |  | None |

***Girls at Early Preference***

**Table S39**

*Alternative Models: Early Preference and Its Relation to Current Gender Identity (Girls at Early Preference)*

| Coding of Expansive Gender Categories | Comparison of Current Girls and Current Boys/Gender Diverse | | Discrepancies with Main Text |
| --- | --- | --- | --- |
|  | Result | Interpretation |  |
| *Expansive* recoded to *Boy* or *Girl* | *b* = 0.08, *p =* .006 | Current girls more feminine than current boys/gender diverse | N/A |
| *Expansive* recoded to *Gender Diverse* | *b* = 0.06, *p* = .016 |  | None |

**Question 3: *Parent Questionnaire* and Its Relation to Current Gender Identity**

***Boys at Early Parent Questionnaire***

**Table S40**

*Alternative Models: Early Parent Questionnaire and Its Relation to Current Gender Identity (Boys at Early Parent Questionnaire)*

| Coding of Expansive Gender Categories | Comparison of Boys and Current Girls/Gender Diverse | | Discrepancies with Main Text |
| --- | --- | --- | --- |
|  | Result | Interpretation |  |
| *Expansive* recoded to *Boy* or *Girl* | *b* = -0.04, *p =* .107 | No difference | N/A |
| *Expansive* recoded to *Gender Diverse* | *b* = -0.05, *b* = .015 | Current boys more masculine than current girls/gender diverse | Significant effect of current gender identity found |

***Girls at Early Parent Questionnaire***

**Table S41**

*Alternative Models: Early Parent Questionnaire and Its Relation to Current Gender Identity (Girls at Early Parent Questionnaire)*

| Coding of Expansive Gender Categories | Comparison of Current Girls and Current Boys/Gender Diverse | | Discrepancies with Main Text |
| --- | --- | --- | --- |
|  | Result | Interpretation |  |
| *Expansive* recoded to *Boy* or *Girl* | *b* = 0.03, *p =* .143 | No difference | N/A |
| *Expansive* recoded to *Gender Diverse* | *b* = 0.03, *p* = .056 |  | None |

**Supporting Information for Chapter VI:**

**Sexual Orientation Across Childhood and Adolescence**

**Chapter VI, Research Question 1: Overall Rates of Sexual Orientation Identification**

In this section, we have two main goals:

1. We present descriptive results (i.e., what percentage of youths identified with various sexual orientation identities) broken down by *recruitment group* and *recruitment gender*, rather than by current gender and current modality as in the main text.
2. We present alternative results on inferential models, illustrating whether results do or do not change if (a) expansive gender categories are recoded as gender diverse rather than boy or girl; and/or (b) *Target of Attraction* codes of *mostly boys* and *mostly girls* are recoded as *only boys* and *only girls* respectively (rather than being recoded as *both boys and girls*, as in the main text).

**Face-to-Face Visits with Younger Youth**

In face-to-face visits, we measured romantic interest by asking youth if they had or had not had a crush on a boy and/or girl (*Childhood Sexuality Measure*; see Table 22 in the main text Chapter VI for full wording). In the main text, Table 23 showed results broken down by youth’s identities at the time of report; here, Table S42 shows results broken down by *recruitment group* and *recruitment gender*.

**Table S42**

*Target of Attraction as Reported on Most Recent Response to Childhood Sexuality Measure, Broken Down by Recruitment Group and Recruitment Gender*

| Recruitment group | Recruitment gender | N | Mean age (years) at visit | Youth reporting a crush on: N(%) | | | | N : No report on Crush - Yes/No measure |
| --- | --- | --- | --- | --- | --- | --- | --- | --- |
|  |  |  |  | Only boys | Both boys and girls | Only girls | No interest expressed |  |
| Recruited as Transgender | Boy | 52 | 11.4 | 3 (5.8%) | 10 (19.2%) | 23 (44.2%) | 16 (30.8%) | 57 |
|  | Girl | 108 | 11.3 | 33 (30.6%) | 25 (23.1%) | 8 (7.4%) | 42 (38.9%) | 100 |
| Recruited as Cisgender | Boy | 52 | 11.3 | 0 (0%) | 2 (3.8%) | 31 (59.6%) | 19 (36.5%) | 76 |
|  | Girl | 110 | 11.2 | 61 (55.5%) | 14 (12.7%) | 0 (0%) | 35 (31.8%) | 139 |
| Recruited as Siblings | Boy | 32 | 11.2 | 0 (0%) | 1 (3.1%) | 23 (71.9%) | 8 (25.0%) | 93 |
|  | Girl | 30 | 11.3 | 12 (40.0%) | 10 (33.3%) | 0 (0%) | 8 (26.7%) | 63 |

*Note.* “N: No report on Crush – Yes/No measure” refers to number of participants who never gave a response to Crush – Yes/No measure at a visit in which they also gave a codable report of their gender.

We also tested whether, among youth with reports on these face-to-face visits, reports of *queer* (vs. *straight*) attraction differ by gender (boy vs. girl), modality (binary transgender vs. cisgender), and their interaction; Table S43 summarizes alternative results to this question depending on whether expansive gender categories are coded as boy/girl or gender diverse.

**Table S43**

*Alternative Results: Among Children Aged 9-14 Surveyed on Face-to-Face Visits, Do Reports of Queer (vs. Straight) Attraction Differ by Gender (Boy vs. Girl), Modality (Binary Transgender vs. Cisgender), and Their Interaction?*

| Coding of Expansive Gender Categories | Effect of *Gender* (Comparison between boys and girls) | | Effect of *Modality* (Comparison between binary transgender and cisgender) | | Interaction between *Gender and Modality* | | Discrepancies with Main Text |
| --- | --- | --- | --- | --- | --- | --- | --- |
|  | Result | Interpretation | Result | Interpretation | Result | Interpretation |  |
| *Expansive* recoded to *Boy* or *Girl* | *OR* = 0.35, *p* = .016 | Boys more likely to be *straight* than girls | *OR* = 6.12, *p* = .001 | Binary transgender more likely to be *queer* than cisgender | *OR* = 0.48, *p* = .380 | No interaction between *gender* and *modality* | N/A |
| *Expansive* recoded to *Gender Diverse* | *OR* = 0.32, *p* = .005 | Boys more likely to be *straight* than girls | *OR* = 4.87, *p* < .001 | Binary transgender more likely to be *queer* than cisgender | *OR* = 0.42, *p* = .273 | No interaction between *gender* and *modality* | Random intercept for family removed ^a^ |

*Note.* a. This model converged without a singular fit when including a random intercept for each family; however, implausible odds ratios (e.g., > 3000) indicated poor model fit; thus, this random intercept was removed. Significance of model predictors was the same with or without including random effects structure.

**Online Survey Visits with Older Youth**

***Target of Attraction (Towards Boys and Girls)***

**Descriptive Statistics of *Target of Attraction* Broken Down by *Recruitment Group* and *Recruitment Gender.*** Table S44 shows youths’ most recent *Target of Attraction* expressed in an online survey visit, broken down by *recruitment group* and *recruitment gender*.

**Table S44**

*Target of Attraction as Indicated on Youths’ Most Recent Online Survey Visits, Broken Down by Recruitment Group and Recruitment Gender*

| Recruitment group | Recruitment gender | N with report | Mean age (years) at visit | Youth expressing interest in: N (% of those with a report) | | | | | | N : No report on online survey |
| --- | --- | --- | --- | --- | --- | --- | --- | --- | --- | --- |
|  |  |  |  | Only boys | Mostly boys | Both boys and girls | Mostly girls | Only girls | No interest expressed |  |
| Recruited as Transgender | Boy | 84 | 16.2 | 7 (8.3%) | 9 (10.7%) | 25 (29.8%) | 9 (10.7%) | 30 (35.7%) | 4 (4.8%) | 25 |
|  | Girl | 150 | 15.3 | 48 (32.0%) | 10 (6.7%) | 38 (25.3%) | 11 (7.3%) | 27 (18.0%) | 16 (10.7%) | 58 |
| Recruited as Cisgender | Boy | 80 | 16 | 1 (1.2%) | 2 (2.5%) | 8 (10.0%) | 5 (6.2%) | 59 (73.8%) | 5 (6.2%) | 48 |
|  | Girl | 154 | 15.3 | 76 (49.4%) | 20 (13.0%) | 38 (24.7%) | 5 (3.2%) | 8 (5.2%) | 7 (4.5%) | 95 |
| Recruited as Siblings | Boy | 74 | 15.3 | 2 (2.7%) | 1 (1.4%) | 10 (13.5%) | 7 (9.5%) | 52 (70.3%) | 2 (2.7%) | 51 |
|  | Girl | 54 | 15.8 | 21 (38.9%) | 2 (3.7%) | 18 (33.3%) | 3 (5.6%) | 6 (11.1%) | 4 (7.4%) | 39 |

**Inferential Questions with *Target of Attraction* from Online Survey Visits.** We also ran a series of inferential tests involving *Target of Attraction* as reported in online survey visits. Here, we report alternative results obtained when making different variable-coding decisions (i.e., expansive categories recoded to gender diverse rather than boy/girl, “mostly” *Target of Attraction* categories coded as “only” categories, rather than *Both Boys and Girls*).

**Table S45**

*Alternative Results: Are Boys vs. Girls, or Transgender vs. Cisgender Youths, More Likely to Report Queer Attraction?*

| Coding of: | | Effect of *Gender* (Comparison between boys and girls) | | Effect of *Modality* (Comparison between binary transgender and cisgender) | | Interaction between *Gender and Modality* | | Discrepancies with Main Text |
| --- | --- | --- | --- | --- | --- | --- | --- | --- |
| Expansive gender categories | *“*Mostly*”* Target of Attraction categories | Result | Interpretation | Result | Interpretation | Result | Interpretation |  |
| *Expansive* recoded to *Boy* or *Girl* | *Mostly* recoded to *Both boys and girls* | *OR* = 0.52, *p* = .001 | Boys more likely than girls to be straight | *OR* = 4.13, *p* < .001 | Cisgender more likely than transgender to be straight | *OR* = 0.28, *p* = .003 | Difference between boys and girls was more pronounced in cisgender than transgender | N/A |
|  | *Mostly* recoded to *Only boys* or *Only girls* | *OR* = 0.51, *p* = .001 |  | *OR* = 4.53, *p* < .001 |  | *OR* = 0.34, *p* = .009 |  | Random intercept of family removed |
| *Expansive* recoded to *Gender Diverse* | *Mostly* recoded to *Both boys and girls* | *OR* = 0.53, *p* < .002 |  | *OR* = 3.67, *p* < .001 |  | *OR* = 0.31, *p* = .007 |  | None |
|  | *Mostly* recoded to *Only boys* or *Only girls* | *OR* = 0.52, *p* = .002 |  | *OR* = 4.04, *p* < .001 |  | *OR* = 0.35, *p* = .014 |  | Random intercept of family removed |

**Table S46**

*Alternative Results: Are Some Groups of Queer Youths (e.g., Boys, Trans Youths) More Likely to be Bisexual vs. Gay/Lesbian*?

| Coding of: | | Effect of *Gender* (Comparison between boys and girls) | | Effect of *Modality* (Comparison between binary transgender and cisgender) | | Interaction between *Gender and Modality* | | Discrepancies with Main Text |
| --- | --- | --- | --- | --- | --- | --- | --- | --- |
| Expansive gender categories | *“*Mostly*”* Target of Attraction categories | Result | Interpretation | Result | Interpretation | Result | Interpretation |  |
| *Expansive* recoded to *Boy* or *Girl* | *Mostly* recoded to *Both boys and girls* | *OR* = 1.22, *p* = .584 | No difference | *OR* = 0.63, *p* = .196 | No difference | *OR* = 0.48, *p* = .304 | No interaction | N/A |
|  | *Mostly* recoded to *Only boys* or *Only girls* | *OR* = 0.92, *p* = .796 |  | *OR* = 0.68, *p* = .245 |  | *OR* = 0.49, *p* = .286 |  | None |
| *Expansive* recoded to *Gender Diverse* | *Mostly* recoded to *Both boys and girls* | *OR* = 1.24, *p* = .581 |  | *OR* = 0.59, *p* = .180 |  | *OR* = 0.62, *p* = .539 |  |  |
|  | *Mostly* recoded to *Only boys* or *Only girls* | *OR* = 0.88, *p* = .733 |  | *OR* = 0.62, *p* = .182 |  | *OR* = 0.49, *p* = .318 |  |  |

**Table S47**

*Alternative Results: Do Binary Transgender, Cisgender, and Gender Diverse Youths Differ in Rates of Being Bisexual (Rather than Attracted to One Gender)?*

| Coding of: | | Omnibus Statistic | Pairwise comparison between Cisgender and Gender Diverse | | Pairwise comparison between Cisgender and Transgender | | Pairwise comparison between Gender Diverse and Transgender | | Discrepancies with Main Text |
| --- | --- | --- | --- | --- | --- | --- | --- | --- | --- |
| Expansive gender categories | *“*Mostly*”* Target of Attraction categories |  | Result | Interpretation | Result | Interpretation | Result | Interpretation |  |
| *Expansive* recoded to *Boy* or *Girl* | *Mostly* recoded to *Both boys and girls* | Wald χ^2^(2) = 36.47, *p* < .001 | *OR* = 0.04, *p* < .001 | Cisgender less likely than Gender Diverse to be *Bisexual* | *OR* =0 .43, *p* < .001 | Cisgender less likely than Transgender to be *Bisexual* | *OR* = 9.80, *p* < .001 | Gender Diverse more likely than Transgender to be *Bisexual* | N/A |
|  | *Mostly* recoded to *Only boys* or *Only girls* | Wald χ^2^(2) = 28.08, *p* < .001 | *OR* = 0.03, *p* < .001 |  | *OR* = 0.45, *p =* .003 |  | *OR* = 13.54, *p* < .001 |  | None |
| *Expansive* recoded to *Gender Diverse* | *Mostly* recoded to *Both boys and girls* | Wald χ^2^(2) = 40.98, *p* < .001 | *OR* = 0.10, *p* < .001 |  | *OR* = 0.44, *p* < .001 |  | *OR* = 4.55, *p* < .001 |  |  |
|  | *Mostly* recoded to *Only boys* or *Only girls* | Wald χ^2^(2) = 30.54, *p* < .001 | *OR* = 0.11, *p* < .001 |  | *OR* = 0.49, *p =* .009 |  | *OR* = 4.59, *p* < .001 |  |  |

***Asexuality/Aromanticism***

**Descriptive Statistics of *Asexual* Broken Down by *Recruitment Group* and *Recruitment Gender.*** Table S48 shows youths’ most recent code on *Asexual* expressed in an online survey visit, broken down by *recruitment group* and *recruitment gender*, rather than by current gender and current modality as in the main text.

**Table S48**

*Most Recent Youth Self-Identification as Asexual on Online Survey Visit, Broken Down by Recruitment Group and Recruitment Gender*

| Recruitment group | Recruitment gender | Youth without any report on asexuality: N | Youth with report on asexuality: N | Mean age at latest report of asexuality (years) | Not asexual or aromantic: N (%) | Asexual or aromantic: N (%) | *Target of Attraction* among youth coded as *Asexual*: N | | | | | |
| --- | --- | --- | --- | --- | --- | --- | --- | --- | --- | --- | --- | --- |
|  |  |  |  |  |  |  | Only boys | Mostly boys | Both boys and girls | Mostly girls | Only girls | Not interested in boys or girls |
| Recruited as Transgender | Boy | 26 | 83 | 16.2 | 74 (89.2%) | 9 (10.8%) | 0 | 1 | 4 | 0 | 1 | 3 |
|  | Girl | 62 | 146 | 15.3 | 130 (89.0%) | 16 (11.0%) | 1 | 0 | 1 | 2 | 1 | 11 |
| Recruited as Cisgender | Boy | 49 | 79 | 16 | 73 (92.4%) | 6 (7.6%) | 0 | 0 | 1 | 0 | 1 | 4 |
|  | Girl | 99 | 150 | 15.3 | 131 (87.3%) | 19 (12.7%) | 2 | 3 | 7 | 0 | 2 | 5 |
| Recruited as Siblings | Boy | 51 | 74 | 15.3 | 72 (97.3%) | 2 (2.7%) | 0 | 0 | 0 | 0 | 1 | 1 |
|  | Girl | 40 | 53 | 15.8 | 51 (96.2%) | 2 (3.8%) | 1 | 0 | 0 | 0 | 0 | 1 |

*Note*. Includes only youth who participated in online survey visits in which they could have been coded as *Asexual* (i.e., completed a relevant measure; N = 585).

**Inferential Questions with *Asexual.*** We also ran a series of inferential tests involving *Asexual.* Here, we report alternative results obtained under different variable coding decisions.

**Table S49**

*Alternative Results: Relation Between Asexuality and Gender Modality (Binary Transgender, Cisgender, or Gender Diverse)*

| Coding of Expansive Gender Categories | Omnibus Statistic | Pairwise comparison between Cisgender and Gender Diverse | | Pairwise comparison between Cisgender and Transgender | | Pairwise comparison between Gender Diverse and Transgender | | Discrepancies with Main Text |
| --- | --- | --- | --- | --- | --- | --- | --- | --- |
|  |  | Result | Interpretation | Result | Interpretation | Result | Interpretation |  |
| *Expansive* recoded to *Boy* or *Girl* | χ^2^(2) = 23.84, *p* < .001 | *OR* = 0.13, *p* < .001 | Cisgender less than Gender Diverse likely to be *Asexual* | *OR* = 0.50, *p =* .099 | No difference | *OR* = 4.02, *p =* .001 | Gender diverse more likely than Transgender to be *Asexual* | N/A |
| *Expansive* recoded to *Gender Diverse* | χ^2^(2) = 31.12, *p* < .001 | *OR* = 0.13, *p* < .001 |  | *OR* = 0.56, *p =* .269 |  | *OR* = 4.27, *p <* .001 |  | None |

**Table S50**

*Alternative Results: Relation Between Asexuality and Gender Identity (Boy, Girl, or Gender Diverse)*

| Coding of Expansive Gender Categories | Omnibus Statistic | Pairwise comparison between Boys and Gender Diverse | | Pairwise comparison between Boys and Girls | | Pairwise comparison between Gender Diverse and Girls | | Discrepancies with Main Text |
| --- | --- | --- | --- | --- | --- | --- | --- | --- |
|  |  | Result | Interpretation | Result | Interpretation | Result | Interpretation |  |
| *Expansive* recoded to *Boy* or *Girl* | χ^2^(2) = 23.06, *p* < .001 | *OR* = 0.15, *p* < .001 | Boys less likely than Gender Diverse likely to be *Asexual* | *OR* = 0.79, *p =* .769 | No difference | *OR* = 5.26, *p* < .001 | Gender diverse more likely than Girls to be *Asexual* | N/A |
| *Expansive* recoded to *Gender Diverse* | χ^2^(2) = 29.25, *p* < .001 | *OR* = 0.14, *p* < .001 |  | *OR* = 0.76, *p =* .76 |  | *OR* = 5.29, *p <* .001 |  | None |

**Table S51**

*Alternative Results: Relation Between Asexuality and Target of Attraction (Only Boys, Both Boys and Girls, and Only Girls)*

| Coding of *“Mostly”* Target of Attraction categories | Omnibus Statistic | Pairwise comparison between bisexual youths and those interested in *only boys* | | Pairwise comparison between bisexual youths and those interested in *only girls* | | Pairwise comparison between youths interested in *only boys* and *only girls* | | Discrepancies with Main Text |
| --- | --- | --- | --- | --- | --- | --- | --- | --- |
|  |  | Result | Interpretation | Result | Interpretation | Result | Interpretation |  |
| *Mostly* recoded to *Both boys and girls* | χ^2^(2) = 8.62, *p* = .013 | *OR* = 3.56, *p* = .061 | No difference | *OR* = 2.80, *p* = .082 | No difference | *OR* = 0.79, *p* = .927 | No difference | N/A |
| *Mostly* recoded to *Only boys* or *Only girls* | χ^2^(2) = 5.93, *p* = .051 | No follow-up tests conducted (omnibus not significant) | | | | | | Omnibus statistic not significant |

***Interest in Nonbinary People***

**Descriptive Statistics of *Interest in Nonbinary People* Broken Down by *Recruitment Group* and *Recruitment Gender.*** Table S42 shows youths’ most recent code on *Interest in Nonbinary People* expressed in an online survey visit, broken down by *recruitment group* and *recruitment gender*, rather than by current gender and current modality as in the main text.

**Table S52**

*Most Recently Expressed Interest or Non-Interest in Nonbinary People Among Youth*

| Recruitment group | Recruitment gender | Mean age at visit (years) | N with a report of interest in nonbinary | N (% of those with report) | | N: No report |
| --- | --- | --- | --- | --- | --- | --- |
|  |  |  |  | Interested in nonbinary | Not interested in nonbinary |  |
| Recruited as Transgender | Boy | 16.3 | 79 | 37 (46.8%) | 42 (53.2%) | 30 |
|  | Girl | 15.4 | 129 | 64 (49.6%) | 65 (50.4%) | 79 |
| Recruited as Cisgender | Boy | 16 | 75 | 20 (26.7%) | 55 (73.3%) | 53 |
|  | Girl | 15.4 | 142 | 50 (35.2%) | 92 (64.8%) | 107 |
| Recruited as Siblings | Boy | 15.4 | 67 | 24 (35.8%) | 43 (64.2%) | 58 |
|  | Girl | 16 | 49 | 27 (55.1%) | 22 (44.9%) | 44 |

**Inferential Questions with *Interest in Nonbinary People.*** We also ran a series of inferential tests involving *Interest in Nonbinary People.* Here, we report alternative results obtained under different variable coding decisions.

**Table S53**

*Alternative Results: Relation Between Interest in Nonbinary People and Gender Modality (Binary Transgender, Cisgender, or Gender Diverse)*

| Coding of Expansive Gender Categories | Omnibus Statistic | Pairwise comparison between Cisgender and Gender Diverse | | Pairwise comparison between Cisgender and Transgender | | Pairwise comparison between Gender Diverse and Transgender | | Discrepancies with Main Text |
| --- | --- | --- | --- | --- | --- | --- | --- | --- |
|  |  | Result | Interpretation | Result | Interpretation | Result | Interpretation |  |
| *Expansive* recoded to *Boy* or *Girl* | Wald χ^2^(2) = 23.84, *p* < .001 | *OR* = 0.07, *p* < .001 | Cisgender less likely than Gender Diverse to be *Interested in Nonbinary* | *OR* = 0.42, *p* < .001 | Cisgender less likely than Transgender to be *Interested in Nonbinary* | *OR* = 6.06, *p* < .001 | Gender Diverse more likely than Transgender to be *Interested in Nonbinary* | N/A |
| *Expansive* recoded to *Gender Diverse* | Wald χ^2^(2) = 53.41, *p* < .001 | *OR* = 0.06, *p* < .001 |  | *OR* = 0.44, *p* < .001 |  | *OR* = 7.93, *p <* .001 |  | None |

**Table S54**

*Alternative Results: Relation Between Interest in Nonbinary People and Gender Identity (Boy, Girl, or Gender Diverse)*

| Coding of Expansive Gender Categories | Omnibus Statistic | Pairwise comparison between Boys and Gender Diverse | | Pairwise comparison between Boys and Girls | | Pairwise comparison between Gender Diverse and Girls | | Discrepancies with Main Text |
| --- | --- | --- | --- | --- | --- | --- | --- | --- |
|  |  | Result | Interpretation | Result | Interpretation | Result | Interpretation |  |
| *Expansive* recoded to *Boy* or *Girl* | χ^2^(2) = 27.50, *p* < .001 | *OR* = 0.08, *p* < .001 | Boys less likely than Gender Diverse likely to be *Interested in Nonbinary* | *OR* = 0.64, *p =* .066 | No difference | *OR* = 8.42, *p* < .001 | Gender diverse more likely than Girls to be *Interested in Nonbinary* | N/A |
| *Expansive* recoded to *Gender Diverse* | χ^2^(2) = 45.30, *p* < .001 | *OR* = 0.06, *p* < .001 |  | *OR* = 0.71, *p =* .211 |  | *OR* = 11.22, *p <* .001 |  | None |

**Table S55**

*Alternative Results: Relation Between Interest in Nonbinary People and Target of Attraction (Only Boys, Both Boys and Girls, and Only Girls)*

| Coding of *“Mostly”* Target of Attraction categories | Omnibus Statistic | Pairwise comparison between bisexual youths and those interested in *only boys* | | Pairwise comparison between bisexual youths and those interested in *only girls* | | Pairwise comparison between youths interested in *only boys* and *only girls* | | Discrepancies with Main Text |
| --- | --- | --- | --- | --- | --- | --- | --- | --- |
|  |  | Result | Interpretation | Result | Interpretation | Result | Interpretation |  |
| *Mostly* recoded to *Both boys and girls* | χ^2^(2) = 46.08, *p* < .001 | *OR* = 33.77, *p* < .001 | Bisexual youths more likely than those interested in *only boys* to be *Interested in Nonbinary* | *OR* = 10.17, *p* < .001 | Bisexual youths more likely than those interested in *only girls* to be *Interested in Nonbinary* | *OR* = 0.30, *p* = .002 | Youths interested in *only boys* less likely than youths interested in *only girls* to be *Interested in Nonbinary* | N/A |
| *Mostly* recoded to *Only boys* or *Only girls* | χ^2^(2) = 53.29, *p <* .001 . | *OR* = 25.11, *p <* .001 |  | *OR* = 10.57, *p* < .001 |  | *OR* = 0.42, *p* = .002 |  | None |

***Questioning***

**Descriptive Statistics of *Questioning* Broken Down by *Recruitment Group* and *Recruitment Gender.*** Table S56 shows youths’ most recent code on *Questioning* expressed in an online survey visit, broken down by *recruitment group* and *recruitment gender*, rather than by current gender and current modality as in the main text.

**Table S56**

*Most Recently Expressed Report of Questioning Sexuality Among Youth, Broken Down by Recruitment Group and Recruitment Gender*

| Recruitment group | Recruitment gender | Mean age at visit (years) | N with report of *Questioning* | N (% of total with report) | | N: No report |
| --- | --- | --- | --- | --- | --- | --- |
|  |  |  |  | *Questioning* | Not *Questioning* |  |
| Recruited as Transgender | Boy | 16.2 | 83 | 14 (16.9%) | 69 (83.1%) | 26 |
|  | Girl | 15.3 | 150 | 21 (14.0%) | 129 (86.0%) | 58 |
| Recruited as Cisgender | Boy | 16 | 80 | 6 (7.5%) | 74 (92.5%) | 48 |
|  | Girl | 15.3 | 154 | 29 (18.8%) | 125 (81.2%) | 95 |
| Recruited as Siblings | Boy | 15.3 | 74 | 4 (5.4%) | 70 (94.6%) | 51 |
|  | Girl | 15.8 | 53 | 13 (24.5%) | 40 (75.5%) | 40 |

**Inferential Questions with *Interest in Nonbinary.*** We also ran a series of inferential tests involving *Asexual.* Here, we report alternative results obtained under different variable coding decisions.

**Table S57**

*Alternative Results: Relation Between Questioning and Gender Modality (Binary Transgender, Cisgender, or Gender Diverse)*

| Coding of Expansive Gender Categories | Omnibus Statistic | Interpretation | Discrepancies with Main Text |
| --- | --- | --- | --- |
| *Expansive* recoded to *Boy* or *Girl* | χ^2^(2) = 0.59, *p* = .746 | No effect of gender modality on *Questioning* | N/A |
| *Expansive* recoded to *Gender Diverse* | χ^2^(2) = 0.66, *p =* .718 |  | None |

**Table S58**

*Alternative Results: Relation Between Questioning and Gender Identity (Boy, Girl, or Gender Diverse)*

| Coding of Expansive Gender Categories | Omnibus Statistic | Interpretation | Discrepancies with Main Text |
| --- | --- | --- | --- |
| *Expansive* recoded to *Boy* or *Girl* | χ^2^(2) = 4.64, *p* = .098 | No effect of gender identity on *Questioning* | N/A |
| *Expansive* recoded to *Gender Diverse* | χ^2^(2) = 5.05, *p* < .080 |  | None |

**Table S59**

*Alternative Results: Relation Between Questioning and Target of Attraction (Only Boys, Both Boys and Girls, and Only Girls)*

| Coding of *“Mostly”* Target of Attraction categories | Omnibus Statistic | Pairwise comparison between bisexual youths and those interested in *only boys* | | Pairwise comparison between bisexual youths and those interested in *only girls* | | Pairwise comparison between youths interested in *only boys* and *only girls* | | Discrepancies with Main Text |
| --- | --- | --- | --- | --- | --- | --- | --- | --- |
|  |  | Result | Interpretation | Result | Interpretation | Result | Interpretation |  |
| *Mostly* recoded to *Both boys and girls* | χ^2^(2) = 27.18, *p* < .001 | *OR* = 3.03, *p =* .003 | Bisexual youths more likely than those interested in *only boys* to be *Questioning* | *OR* = 4.77, *p* < .001 | Bisexual youths more likely than those interested in *only girls* to be *Questioning* | *OR* = 1.58, *p* = .549 | No difference | N/A |
| *Mostly* recoded to *Only boys* or *Only girls* | χ^2^(2) = 10.53, *p <* .001 | *OR* = 1.44, *p =* .447 | No difference | *OR* = 2.84, *p =* .005 |  | *OR* = 1.89, *p* = .090 |  | Pairwise comparison between bisexual youths and those interested in *only boys* was not significant |

**Chapter VI, Question 2: Relation Between Earlier Gender Development Measures and Most Recent Sexual Orientation (“Mostly” Categories Recoded as “Only”)**

In the main text of Chapter VI, we conducted several analyses examining the relation between youths’ earliest responses on three gender development measures (*Continuum*, *Preference*, and *Parent Questionnaire*). Here, we report alternate results from these analyses if different variable coding decisions are made (i.e., expansive categories are recoded as gender diverse rather than boy/girl, “mostly” *Target of Attraction* categories recoded as “only” categories rather than *Both Boys and Girls*).

**Table S60**

*Alternative Results: Relation Between Earliest Continuum and Later Target of Attraction in Current Boys*

| Coding of: | | Effect of *Target of Attraction* (*straight* vs. *queer*) | | Effect of *Modality* (Comparison between binary transgender and cisgender) | | Interaction between *Target of Attraction and Modality* | | Discrepancies with Main Text |
| --- | --- | --- | --- | --- | --- | --- | --- | --- |
| Expansive gender categories | *“*Mostly*”* Target of Attraction categories | Result | Interpretation | Result | Interpretation | Result | Interpretation |  |
| *Expansive* recoded to *Boy* or *Girl* | *Mostly* recoded to *Both boys and girls* | *b* = -7.74, *p* = .011 | *Straight* more masculine than *queer* | *b* = -1.56, *p* = .604 | No difference | *b* = 4.56, *p* = .449 | No interaction | N/A |
|  | *Mostly* recoded to *Only boys* or *Only girls* | *b* = -10.18, *p* = .002 |  | *b* = -0.67, *p* = .836 |  | *b* = 1.10, *p* = .866 |  | None |
| *Expansive* recoded to *Gender Diverse* | *Mostly* recoded to *Both boys and girls* | *b* = -6.12, *p =* .041 |  | *b* = 0.01, *p =* .998 |  | *b* = 1.15, *p =* .797 |  |  |
|  | *Mostly* recoded to *Only boys* or *Only girls* | *b* = -8.11, *p* = .014 |  | *b* = 1.39, *p* = .672 |  | *b* = -3.29, *p* = .616 |  |  |

**Table S61**

*Alternative Results: Relation Between Earliest Continuum and Later Target of Attraction in Current Girls*

| Coding of: | | Effect of *Target of Attraction* (*straight* vs. *bisexual* vs. *lesbian*) | | | | | | | Effect of *modality* (*binary transgender* vs. cisgender) | | Interaction between *Target of Attraction* and *Modality* | | Discrepancies with Main text |
| --- | --- | --- | --- | --- | --- | --- | --- | --- | --- | --- | --- | --- | --- |
| Expansive gender categories | *“*Mostly*”* Target of Attraction categories | Omnibus statistic | Pairwise: *straight* vs. *bi* | | Pairwise: *straight* vs. *lesbian* | | Pairwise: *bi* vs. *lesbian* | | Omnibus statistic | Interpretation | Omnibus statistic | Interpretation |  |
|  |  |  | Result | Interpretation | Result | Interpretation | Result | Interpretation |  |  |  |  |  |
| *Expansive* recoded to *Boy* or *Girl* | *Mostly* recoded to *Both boys and girls* | *F*(2, 302) = .039 | *b* = 1.92, *p* = .702 | No difference | *b* = 9.77, *p* = .029 | *Straight* more feminine than *lesbian* | *b* = 7.86, *p* = .102 | No difference | *F*(1, 302) = 0.32, *p* = 0.572 | No difference | *F*(2, 302) = 1.39, *p* = .251 | No interaction | N/A |
|  | *Mostly* recoded to *Only boys* or *Only girls* | *F*(2, 302) = .016 | *b* = -0.92, *p* = .938 |  | *b* = 8.32, *p* = .022 |  | *b* = 9.23, *p* = .023 | *Bi* more feminine than *lesbian* | *F*(1, 302) = 0.23, *p* = .635 |  | *F*(2, 302) = 1.46, *p* = 0.233 |  | Difference found between *bi* and *lesbian* |
| *Expansive* recoded to *Gender Diverse* | *Mostly* recoded to *Both boys and girls* | *F*(2, 282) = .069 | No follow-up tests conducted (omnibus not significant) | | | | | | *F*(1, 282) = 0.88, *p* = 0.349 |  | *F*(2, 282) = 1.49, *p* = .227 |  | Omnibus of *Target of Attraction* not significant |
|  | *Mostly* recoded to *Only boys* or *Only girls* | *F*(2, 282) = .022 | *b* = -2.14, *p* = .707 | No difference | *b* = 7.95, *p* = .046 | *Straight* more feminine than *lesbian* | *b* = 10.08, *p* = .020 | *Bi* more feminine than *lesbian* | *F*(1, 282) = 1.28, *p* = .259 |  | *F*(2, 282) = 2.41, *p* = .091 |  | Difference found between *bi* and *lesbian* |

**Table S62**

*Alternative Results: Relation Between Earliest Preference and Later Target of Attraction in Current Boys*

| Coding of: | | Effect of *Target of Attraction* (*straight* vs. *queer*) | | Effect of *Modality* (Comparison between binary transgender and cisgender) | | Interaction between *Target of Attraction and Modality* | | Discrepancies with Main Text |
| --- | --- | --- | --- | --- | --- | --- | --- | --- |
| Expansive gender categories | *“*Mostly*”* Target of Attraction categories | Result | Interpretation | Result | Interpretation | Result | Interpretation |  |
| *Expansive* recoded to *Boy* or *Girl* | *Mostly* recoded to *Both boys and girls* | *b* = -0.09, *p* = .003 | *Straight* more masculine than *queer* | *b* = 0.03, *p* = .241 | No difference | *b* = 0.08, *p* = .176 | No interaction | N/A |
|  | *Mostly* recoded to *Only boys* or *Only girls* | *b* = -0.12, *p* < .001 |  | *b* = 0.04, *p* = .201 |  | *b* = 0.04, *p* = .505 |  | None |
| *Expansive* recoded to *Gender Diverse* | *Mostly* recoded to *Both boys and girls* | *b* = -0.07, *p =* .020 |  | *b* = 0.06, *p =* .051 |  | *b* = 0.03, *p =* .556 |  |  |
|  | *Mostly* recoded to *Only boys* or *Only girls* | *b* = -0.09, *p* = .003 |  | *b* = 0.07, *p* = .027 | Transgender more masculine than cisgender | *b* = -0.01, *p* = .812 |  | Difference found between transgender and cisgender |

**Table S63**

*Alternative Results: Relation Between Earliest Preference and Later Target of Attraction in Current Girls*

| Coding of: | | Effect of *Target of Attraction* (*straight* vs. *bisexual* vs. *lesbian*) | | | | | | | Effect of *modality* (*binary transgender* vs. cisgender) | | Interaction between *Target of Attraction* and *Modality* | | Discrepancies with Main text |
| --- | --- | --- | --- | --- | --- | --- | --- | --- | --- | --- | --- | --- | --- |
| Expansive gender categories | *“*Mostly*”* Target of Attraction categories | Omnibus statistic | Pairwise: *straight* vs. *bi* | | Pairwise: *straight* vs. *lesbian* | | Pairwise: *bi* vs. *lesbian* | | Omnibus statistic | Interpretation | Omnibus statistic | Interpretation |  |
|  |  |  | Result | Interpretation | Result | Interpretation | Result | Interpretation |  |  |  |  |  |
| *Expansive* recoded to *Boy* or *Girl* | *Mostly* recoded to *Both boys and girls* | *F*(2, 294) = 6.79, *p* = .001 | *b* = 0.06, *p* = .014 | *Straight* more feminine than *bi* | *b* = 0.11, *p* = .006 | *Straight* more feminine than *lesbian* | *b* = 0.04, *p* = .441 | No difference | *F*(1, 294) = 0.00, *p* = .953 | No difference | *F*(2, 294) = 0.08, *p* = .925 | No interaction | N/A |
|  | *Mostly* recoded to *Only boys* or *Only girls* | *F*(2, 294) = 6.09, *p* = .003 | *b* = 0.05, *p* = .090 | No difference | *b* = 0.10, *p* = .004 |  | *b* = 0.04, *p* = .398 |  | *F*(1, 294) = 0.00, *p* = .965 |  | *F*(2, 294) = 0.35, *p* = .706 |  | No difference found between s*traight* and *bi* |
| *Expansive* recoded to *Gender Diverse* | *Mostly* recoded to *Both boys and girls* | *F*(2, 273) = 5.61, *p* = .004 | *b* = 0.05, *p* = .039 | *Straight* more feminine than *bi* | *b* = 0.10, *p* = .013 |  | *b* = 0.05, *p* = .402 |  | *F*(1, 273) = 0.33, *p* = .565 |  | *F*(2, 273) = 0.08, *p* = .927 |  | None |
|  | *Mostly* recoded to *Only boys* or *Only girls* | *F*(2, 273) = 5.30, *p* = .005 | *b* = 0.05, *p* = .147 | No difference | *b* = 0.09, *p* = .008 |  | *b* = .05, *p* = .356 |  | *F*(1, 273) = 0.22, *p* = .641 |  | *F*(2, 273) = 0.01, *p* = .986 |  | No difference found between *straight* and *bi* |

**Table S64**

*Alternative Results: Relation Between Earliest Parent Questionnaire and Later Target of Attraction in Current Boys*

| Coding of: | | Effect of *Target of Attraction* (*straight* vs. *queer*) | | Effect of *Modality* (Comparison between binary transgender and cisgender) | | Interaction between *Target of Attraction and Modality* | | Discrepancies with Main Text |
| --- | --- | --- | --- | --- | --- | --- | --- | --- |
| Expansive gender categories | *“*Mostly*”* Target of Attraction categories | Result | Interpretation | Result | Interpretation | Result | Interpretation |  |
| *Expansive* recoded to *Boy* or *Girl* | *Mostly* recoded to *Both boys and girls* | *b* = -0.04, *p* = .021 | *Straight* more masculine than *queer* | *b* = 0.05, *p* = .002 | Transgender more masculine than cisgender | *b* = 0.03, *p* = .372 | No interaction | N/A |
|  | *Mostly* recoded to *Only boys* or *Only girls* | *b* = -0.04, *p* < .030 |  | *b* = 0.05, *p* = .005 |  | *b* = -0.00, *p* = .978 |  | None |
| *Expansive* recoded to *Gender Diverse* | *Mostly* recoded to *Both boys and girls* | *b* = -0.04, *p =* .041 |  | *b* = 0.07, *p* < .001 |  | *b* = 0.00, *p =* .921 |  |  |
|  | *Mostly* recoded to *Only boys* or *Only girls* | *b* = -0.03, *p* = .140 | No difference | *b* = 0.07, *p* = .027 |  | *b* = -0.02, *p* = .596 |  | No difference found between *straight* and *queer* |

**Table S65**

*Alternative Results: Relation Between Earliest Parent Questionnaire and Later Target of Attraction in Current Girls*

| Coding of: | | Effect of *Target of Attraction* (*straight* vs. *bisexual* vs. *lesbian*) | | | | | | | Effect of *modality* (comparison between binary transgender and cisgender) | | | Interaction of *Target of Attraction* and *modality* | | Discrepancies with Main Text |
| --- | --- | --- | --- | --- | --- | --- | --- | --- | --- | --- | --- | --- | --- | --- |
| Expansive gender categories | *“*Mostly*”* Target of Attraction categories | Omnibus statistic | Pairwise: *straight* vs. *bi* | | Pairwise: *straight* vs. *lesbian* | | Pairwise: *bi* vs. *lesbian* | | Omnibus statistic | Pairwise: binary transgender vs. cisgender | | Omnibus statistic | Interpretation |  |
|  |  |  | Result | Interpretation | Result | Interpretation | Result | Interpretation |  | Result | Interpretation |  |  |  |
| *Expansive* recoded to *Boy* or *Girl* | *Mostly* recoded to *Both boys and girls* | Wald χ^2^(2) = 32.89, *p* < .001 | *b* = 0.06, *p* < .001 | *Straight* more feminine than *bi* | *b* = 0.11, *p* < .001 | *Straight* more feminine than *lesbian* | *b* = 0.04, *p* = .144 | No difference | Wald χ^2^(1) = 8.41, *p* = .004 | *b* = 0.04, *p* = .006 | Transgender more feminine than cisgender | Wald χ^2^(2) = 5.27, *p* = .072 | No interaction | N/A |
|  | *Mostly* recoded to *Only boys* or *Only girls* | Wald χ^2^(2) = 25.78, *p* < .001 | Subsumed in interaction (see Interaction column, right-hand side of table) | | | | | | Wald χ^2^(1) = 26.29, *p* < .001 | Subsumed in interaction (see Interaction column, right-hand side of table) | | Wald χ^2^(2) = 7.08, *p* = .029 | Among girls who are *straight*, binary transgender girls are more feminine than cisgender girls, but this pattern does not hold for *bisexual* or *lesbian* girls | Interaction found between *Target of Attraction* and *modality* |
| *Expansive* recoded to *Gender Diverse* | *Mostly* recoded to *Both boys and girls* | Wald χ^2^(2) = 26.95, *p* < .001 | *b* = 0.05, *p* = .001 | *Straight* more feminine than *bi* | *b* = 0.10, *p* < .001 | *Straight* more feminine than *lesbian* | *b* = 0.05, *p* = .114 | No difference | Wald χ^2^(1) = 21.75, *p* < .001 | *b* = 0.04, *p* = .007 | Transgender more feminine than cisgender | Wald χ^2^(2) = 5.18, *p* = .075 | No interaction | None |
|  | *Mostly* recoded to *Only boys* or *Only girls* | Wald χ^2^(2) = 27.11, *p* < .001 | *b* = 0.03, *p* = .125 | No difference | *b* = 0.09, *p* < .001 |  | *b* = 0.05, *p* = .052 |  | Wald χ^2^(1) = 23.71, *p* < .001 | *b* = 0.04, *p* = .006 |  | Wald χ^2^(2) = 5.21, *p* = .074 |  | No pairwise difference found between s*traight* and *bi* |

**Chapter VI, Research Question 3: Stability and Change in Attraction Over Time**

***Overall Levels of Stability and Change, “Mostly” Categories Recoded as “Only” Categories***

In the main text (Table 25), we showed overall levels stability and change in sexual orientation (broken down by *recruitment gender* and *recruitment group*), if the “mostly” *Target of Attraction* categories are recoded as *both boys and girls*; Table S66 shows parallel results if these “mostly” categories are recoded as *only boys* or *only girls*.

**Table S66**

*Stability and Change in Romantic Interest by Recruitment Group and Recruitment Gender (“Mostly” Categories Recoded into “Only” Categories)*

| Recruitment group | Recruitment gender | Mean age (years) at earliest sexuality report (SD) | Mean years between first and latest sexuality reports (SD) | N | % who change |
| --- | --- | --- | --- | --- | --- |
| Recruited as Transgender | Boy | 13 (1.9) | 3.4 (2) | 67 | 41.8% |
|  | Girl | 12.7 (2) | 2.9 (1.6) | 120 | 45.0% |
| Recruited as Cisgender | Boy | 13.5 (1.9) | 3 (1.8) | 65 | 16.9% |
|  | Girl | 12.6 (1.8) | 3.1 (1.8) | 121 | 48.8% |
| Recruited as Siblings | Boy | 13.1 (2.1) | 2.7 (1.8) | 50 | 26.0% |
|  | Girl | 13.2 (2.1) | 2.7 (2) | 37 | 43.2% |

***Alternative Results on Inferential Test of Whether Recruitment Groups Differ in Sexual Orientation Stability and Change***

In the main text, we tested whether levels of stability and change in sexual orientation differed among youths in the three *recruitment groups* (*Recruited as Transgender*, *Recruited as Cisgender*, and *Recruited as Siblings*). Table S67 shows results from this inferential test depending on how “mostly” *Target of Attraction* categories are recoded (i.e., as *both boys and girls* as in the main text, or as *only boys* or *only girls*).

**Table S67**

*Alternative Results: Testing Differences in Stability and Change in Sexual Orientation Among Recruitment Groups*

| Coding of “Mostly” *Target of Attraction* categories | Effect of *Recruitment Group* (*Recruited as Transgender*, *Recruited as Cisgender*, or *Recruited as Siblings)* | | Effect of *time elapsed* (years) | | Discrepancies with Main Text |
| --- | --- | --- | --- | --- | --- |
|  | Omnibus Statistic | Interpretation | Statistic | Interpretation |  |
| *Mostly* recoded to *Both boys and girls* | Wald χ^2^(2) = 0.90, *p* = .639 | No difference | Wald χ^2^(2) = 9.91, *p* = .002 | More *time elapsed* associated with more change | N/A |
| *Mostly* recoded to *Only boys* or *Only girls* | χ^2^(2) = 2.79, *p* = .248 | No difference | χ^2^(2) = 3.65, *p* = .056 | No effect of *time elapsed* | No effect of *time elapsed* found; random intercept of family removed |

***Figures Illustrating Each Participant’s Sexual Orientation Trajectory***

Figures S9 – S11 show self-reported sexual orientation trajectories among youths who have ever given a report of their *Target of Attraction* (including on a face-to-face visit in childhood). Each line represents one participant in the study. The figures are organized by most recent *Target of Attraction* and whether the youth has or has not shown change in their *Target of Attraction.* Colors correspond to their self-reported gender identity at visits at which they provided reports of their *Target of Attraction*; grey lines indicate periods following visits in which youths did not give a report of *Target of Attraction*.

**Figure S9.** *Trajectories of Attraction, Recruited as Transgender Group*


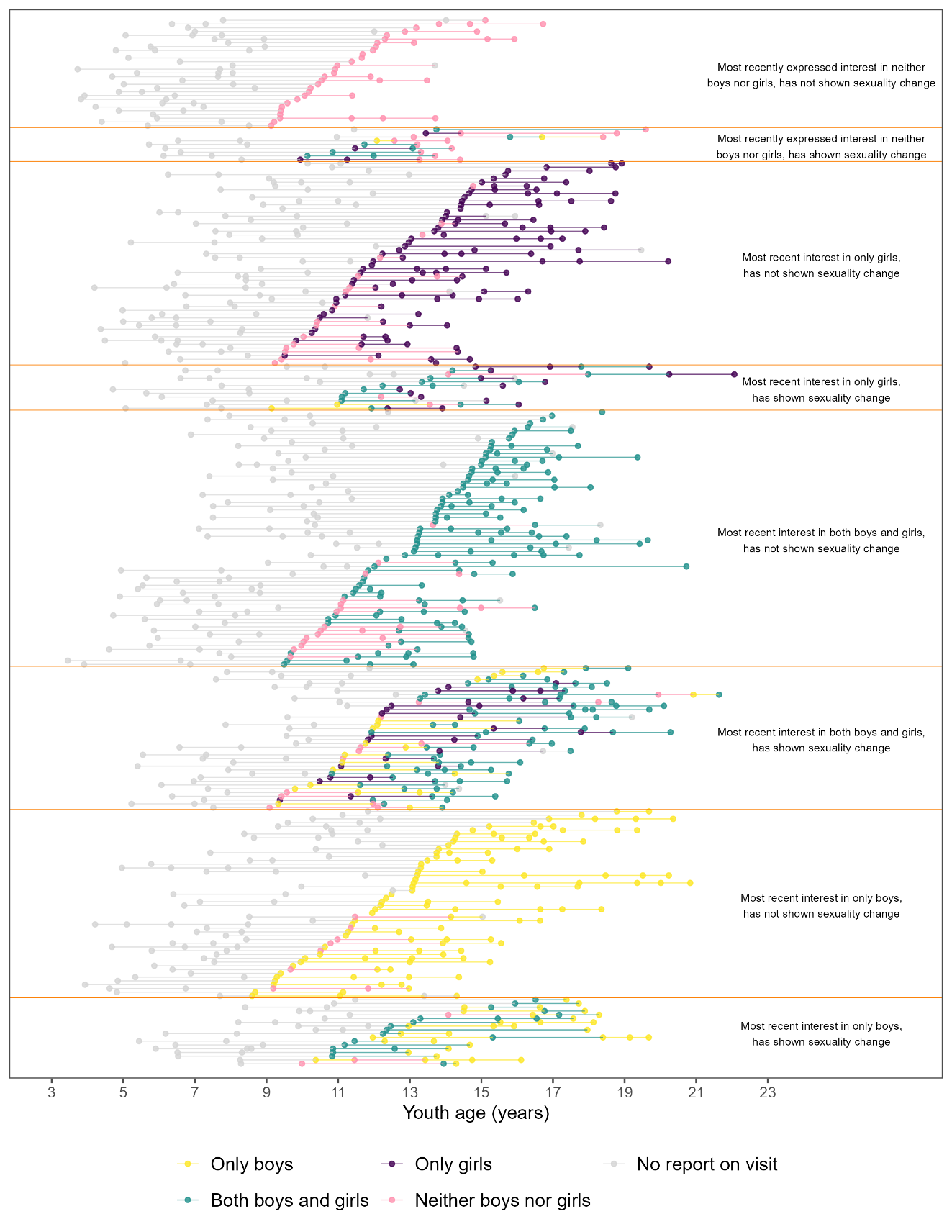


*Note.* Random noise between 0 and 12 months was added to or subtracted from youths’ ages to protect participant privacy. The sequence of presented visits was not altered.

**Figure S10.** *Trajectories of Attraction, Recruited as Cisgender Group*


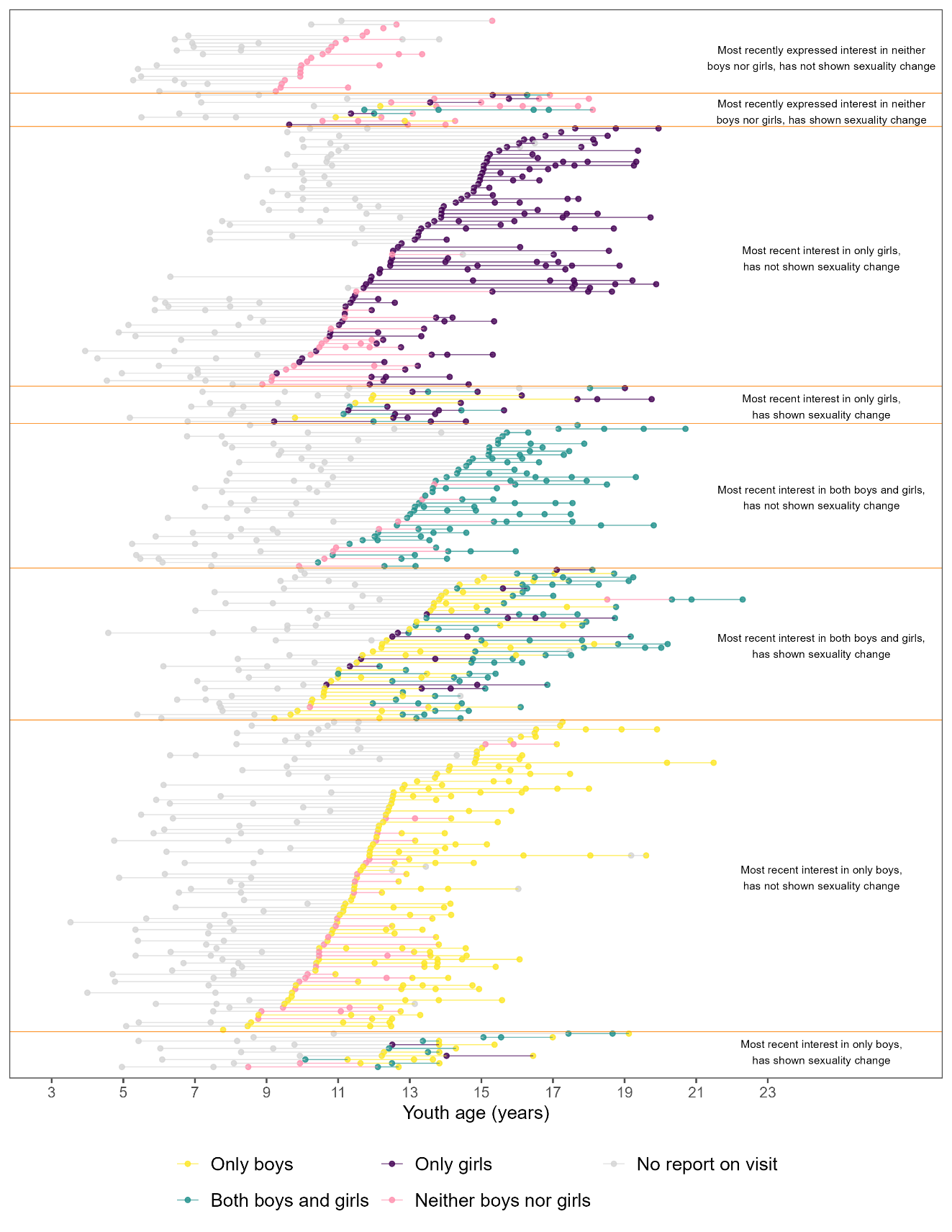


*Note.* Random noise between 0 and 12 months was added to or subtracted from youths’ ages to protect participant privacy. The sequence of presented visits was not altered.

**Figure S11.** *Trajectories of Attraction, Recruited as Siblings Group*


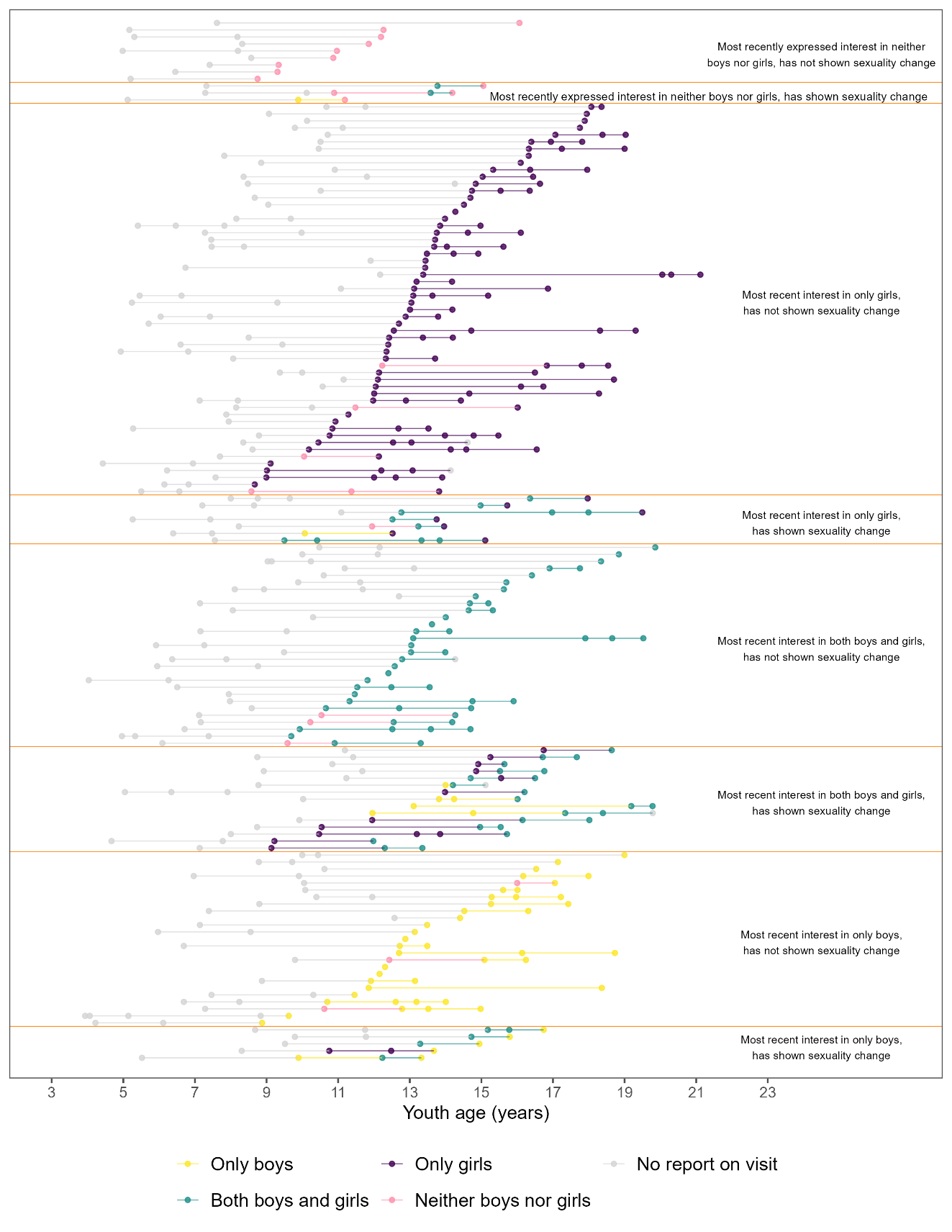


*Note.* Random noise between 0 and 12 months was added to or subtracted from youths’ ages to protect participant privacy. The sequence of presented visits was not altered.
